# Plasma proteomic signatures as predictors of dementia risk in individuals with sleep apnea: a cohort study

**DOI:** 10.1186/s40035-025-00485-6

**Published:** 2025-05-22

**Authors:** Yufei Liu, Pei-Yang Gao, Zhibo Wang, Ruiyang Li, Ke Meng, Weidong Le, Yi Tang

**Affiliations:** 1 Department of Neurology and Innovation Center for Neurological Disorders, Xuanwu Hospital, Capital Medical University, Beijing, 100053 China; 2 National Center for Neurological Disorders, Xuanwu Hospital, Capital Medical University, Beijing, 100053 China; 3 Key Laboratory of Liaoning Province for Research On the Pathogenic Mechanisms of Neurological Diseases, The First Affiliated Hospital, Dalian Medical University, Dalian, 116021 China; 4 Shanghai University of Medicine and Health Sciences Affiliated Zhoupu Hospital, Shanghai, 200000 China; 5 Neurodegenerative Laboratory of Ministry of Education of the People’s Republic of China, Beijing, 100053 China

## Main text

Dementia is a progressive neurodegenerative disorder that often begins with a preclinical, asymptomatic phase, making early diagnosis and intervention particularly difficult. Sleep apnea is characterized by chronic intermittent hypoxia and sleep fragmentation due to intermittent cessations of breathing during sleep, increased β-amyloid deposition, and hippocampal atrophy [1]. Sleep apnea affects one-seventh of adults globally [2], and is strongly associated with cognitive decline and dementia [3]. Guo et al. employed a data-driven proteomics approach in the largest prospective UK biobank cohort with long-term follow-up, identifying key plasma biomarkers such as glial fibrillary acidic protein (GFAP) for incident dementia [4]. Building on this foundation, we conducted this first large-scale prospective study using data from UK Biobank [5, 6], to assess plasma proteomic profiles in sleep apnea patients and identify biomarkers for dementia risk.

Sleep apnea cases were defined as individuals with an International Classification of Diseases Tenth Revision code G47.3 or self-reported diagnosis of sleep apnea. Dementia cases were identified through linked primary care, hospital, and death registry records. Ethical approval was obtained from the North West Multi-Centre Research Ethics Committee (reference: 11/NW/0382). All participants provided written consent to UK Biobank. To determine the contribution of sleep apnea to dementia risk, participants with baseline dementia, developing sleep apnea after baseline, or having snoring without sleep apnea (sleep apnea^−^/snoring^+^) were excluded from the study. As a result, 328,411 individuals were included in the study, including 324,977 with neither sleep apnea nor snoring (sleep apnea^−^/snoring^−^) and 3434 with sleep apnea (sleep apnea^+^) at baseline (Fig. S1). The participants with sleep apnea (mean age 57.84 years) were predominantly males (24.29% being female), and had higher body mass index (BMI) (mean 32.53 kg/m^2^) compared to sleep apnea^−^/snoring^−^ participants (mean age 56.40 years, 61.83% female, mean BMI 26.70 kg/m^2^) (Table S1). Follow-up time was calculated from baseline to dementia diagnosis, death, loss to follow-up, or censoring, whichever occurred first. To control for potential confounders, we applied multivariable regression with two adjustment models: Model 1 adjusting for age and sex; and Model 2 additionally adjusting for smoking status, alcohol consumption, BMI, education, ethnicity, and Townsend deprivation index. Sleep apnea was significantly associated with an increased risk of all-cause dementia (ACD), Alzheimer's disease (AD), and vascular dementia (VaD) in both models (Table S2). Sex-stratified analyses showed that sleep apnea was significantly associated with ACD in both males and females, while the association with AD was not significant in either sex. A significant association with VaD was observed only in the male subgroup (Table S3). BMI-stratified analyses showed that sleep apnea was significantly associated with ACD, AD, or VaD in participants with BMI > 25 kg/m^2^, but not with BMI ≤ 25 kg/m^2^ (Table S4).

**Fig. 1:**
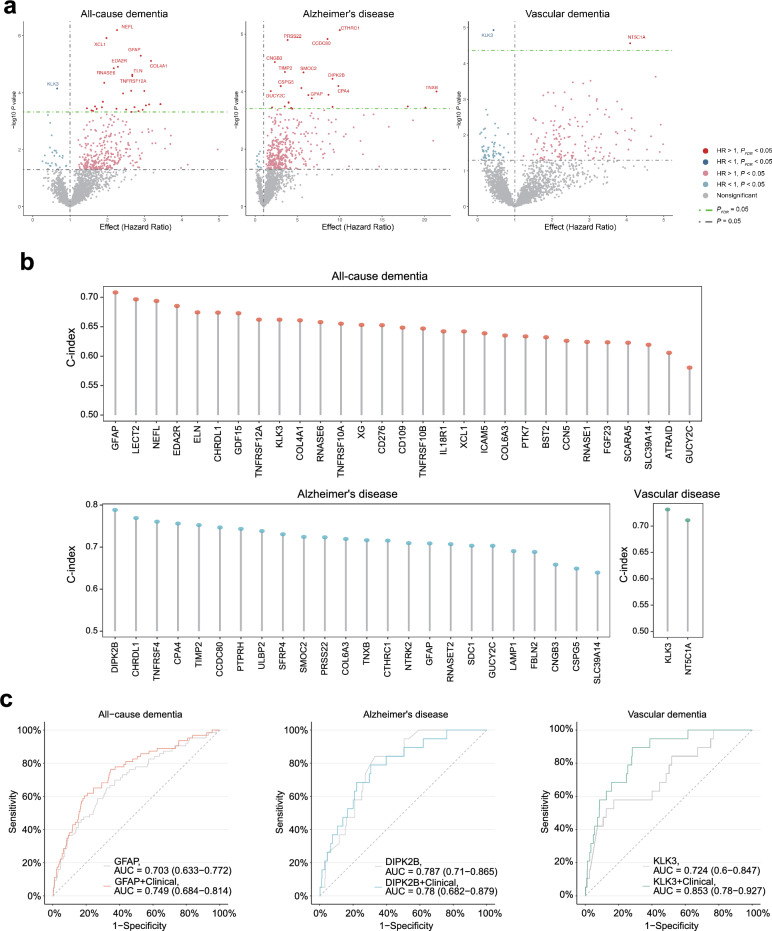
**a** Volcano plots display the hazard ratio and − log10 (*P*-value) for the association of 2920 plasma proteins with incident all-cause dementia, Alzheimer’s disease, and vascular dementia. Results are based on Cox proportional hazards models adjusted for age, sex, smoking status, alcohol use, body mass index, education, ethnicity, and the Townsend Deprivation Index. Proteins above the horizontal dotted green line are significantly associated with dementia after FDR correction (*P*_FDR_ = 0.05). **b** C-index of each protein indicating the predictive power. Proteins are ranked from left to right based on their relative importance in predicting dementia. The bar chart highlights the significance of proteins in forecasting future dementia. **c** Receiver operating curves show the performance of two models in predicting incident all-cause dementia, Alzheimer’s disease, and vascular dementia. The combined model includes demographic factors (age, sex, education, and body mass index). FDR, false discovery rate

For proteomic analyses, we included participants with sleep apnea and available proteomic data, yielding a final cohort of 1282 individuals, among whom 63 developed ACD, 19 AD, and 19 VaD, with a median follow-up of 13.58 years (Table S5, Fig. S1). Cox proportional hazards models, adjusted for Model 2, were employed to estimate the association between 2920 plasma proteins and the risk of ACD, AD, and VaD. To account for multiple testing, the Benjamini–Hochberg correction was applied to control the false discovery rate (FDR), with a significance threshold of *P* < 0.05 [7]. Finally, 29, 24, and 2 proteins were significantly associated with ACD, AD and VaD risk, respectively, in the sleep apnea population. Notably, kallikrein-related peptidase 3 (KLK3) was associated with reduced risk of ACD (HR = 0.66, 95% CI 0.53–0.81, *P* < 0.001, *P*_FDR_ = 0.021) and VaD (HR = 0.43, 95% CI 0.29–0.62, *P* < 0.001, *P*_FDR_ = 0.034) (Fig. 1a, Table S6).

Concordance index (C-index) values derived from Cox proportional hazards regression models were used to rank the dementia-associated proteins in the order of predictive performance in the sleep apnea population. The C-index measures the model's discrimination ability in the time-to-event context, providing an estimate of consistency between predicted dementia risk and actual outcomes. GFAP had the highest C-index value (0.708, 95% CI 0.641–0.775), highlighting its strong predictive ability for ACD. Divergent protein kinase domain 2B (DIPK2B) had the highest C-index for AD prediction (0.788, 95% CI 0.710–0.867). KLK3 had the highest C-index (0.732, 95% CI 0.608–0.856) for VaD (Fig. 1b, Table S7). Subgroup analysis in both sexes revealed significant associations of GFAP, DIPK2B and KLK3 with ACD, AD and VaD risks, respectively (Table S8).

Receiver operating characteristic curve analysis was further used to evaluate the predictive performance of each protein for future dementia outcomes. This analysis used the risk scores derived from our fitted Cox proportional hazards models and generated area under the curve (AUC) values that assess the ability of each protein to discriminate between individuals who would later develop dementia versus those who would not. GFAP showed moderate discrimination for ACD with an AUC of 0.703 (95% CI 0.633–0.772; sensitivity: 0.651; specificity: 0.679). When combined with clinical variables, the AUC increased to 0.749 (95% CI 0.684–0.814; sensitivity: 0.762; specificity: 0.655). For AD, DIPK2B had strong discrimination (AUC = 0.787, 95% CI 0.71–0.865; sensitivity: 0.842; specificity: 0.669), but inclusion of clinical variables did not significantly alter the predictive ability (AUC = 0.780, 95% CI 0.682–0.879; sensitivity: 0.789; specificity: 0.694). KLK3 showed moderate discriminative accuracy for VaD (AUC = 0.724, 95% CI 0.6–0.847; sensitivity: 0.579; specificity: 0.829), which improved significantly when combined with clinical variables (AUC = 0.853, 95% CI 0.78–0.927; sensitivity: 0.895; specificity: 0.718) (Fig. 1c). Subgroup analyses by sex confirmed these findings (Fig. S2).

Furthermore, Gene Ontology (GO) enrichment analysis was performed to identify key biological functions of proteins significantly related to dementia outcomes. GO analysis revealed significant enrichment in immune processes such as "regulation of cytokine production" and "T-helper 1 cell cytokine production" in ACD (Fig. S3a). In AD, enrichment in processes such as “vascular lumen'' and “vascular endothelial growth factor signaling pathway'' indicated vascular involvement (Fig. S3b). Due to the limited number of proteins, VaD pathway enrichment was not performed.

Additionally, we analyzed associations between proteins and dementia risk in sleep apnea^−^/snoring^−^ individuals. Our findings revealed that GFAP was significantly elevated in both sleep apnea and sleep apnea^−^/snoring^−^ groups, and showed strong associations with dementia risk, aligning with prior research indicating that GFAP is a general dementia biomarker [4] (Fig. S4a). In contrast, DIPK2B and KLK3 were significantly associated with dementia risk only in the sleep apnea group, but not in the sleep apnea^−^/snoring^−^ group. This suggests that DIPK2B and KLK3 may have a potentially sleep apnea-specific role in dementia risk (Fig. S4b, c).

Our findings support previous research linking sleep apnea to neurodegeneration, and identified plasma biomarkers [3]. GFAP, a well-established marker of astrocytic activation in response to neuroinflammation and amyloid pathology [4], was elevated in sleep apnea, suggesting hypoxia-associated astrocyte activation and neuroinflammation underlying associated cognitive impairment. We reported an association between DIPK2B and AD in sleep apnea, which warrants further mechanistic investigation. KLK3, traditionally recognized as a prostate-specific antigen, remains understudied in dementia. Our findings suggest a potential neuroprotective role of KLK3 in VaD, possibly through modulating the kallikrein-kinin system to promote angiogenesis and cerebral perfusion [8]. Moreover, kallikrein-related peptidases might influence pathways involved in amyloid and tau pathology, neuroinflammation, and synaptic dysfunction, which warrant further investigation [9].

Several limitations should be considered when interpreting our results. First, the recorded time of diagnosis may not reflect the actual disease onset, precluding proteomic assessments at disease initiation. To account for the potential impact of sleep apnea duration, we additionally adjusted for the interval between sleep apnea diagnosis and protein detection, and obtained similar findings (Table S9). Future datasets with synchronized proteomic sampling at disease onset are needed. Second, the causality and specificity of the identified plasma biomarkers remain unclear. The lack of longitudinal follow-up limits dynamic assessment of diagnoses and proteomic measurements. Moreover, the plasma biomarkers might not directly reflect neuropathological processes due to the blood–brain barrier. Longitudinal proteomic, neuroimaging, and cerebrospinal fluid analyses as well as experimental validation are needed. Third, there was a lack of UK Biobank data on sleep apnea subtypes and severity, limiting interpretation of subtypes and severity in relation to dementia. Finally, the limited number of dementia cases precludes independent validation, and the restriction to the white European cohort limits the generalizability of the finding to the overall population. Larger cohorts of multiple ethnicities are warranted for broader applicability.

In summary, sleep apnea is associated with increased dementia risk. GFAP, DIPK2B, and KLK3 are novel plasma predictors for dementia in cases with sleep apnea. Our findings offer new opportunities for intervention of cognitive decline in sleep apnea.

## Supplementary Information

Additional file 1. **Figure S1**. Study design and analysis workflow. **Figure S2**. Sex subgroup analysis of the predictive accuracy of KLK3 protein for incident dementia. **Figure S3**. GO pathway enrichment analysis. **Figure S4**. Associations between plasma proteins and dementia risk in individuals with neither sleep apnea nor snoring. **Table S1**. Baseline characteristics of the cohort investigating the relationship between sleep apnea and dementia risk. **Table S2**. Associations between sleep apnea and incident all-cause dementia, Alzheimer's disease, and vascular dementia. **Table S3**. Sex subgroup analysis of the associations between sleep apnea and incident dementia. **Table S4**. BMI subgroup analysis of the associations between sleep apnea and incident dementia. **Table S5**. Baseline characteristics of the proteomics study. **Table S6**. Associations between plasma proteins and dementia risk in sleep apnea patients. **Table S7**. C-index for plasma proteins. **Table S8**. Associations of plasma proteins with dementia risk among male and female sleep apnea patients. **Table S9**. Associations of plasma proteins with dementia risk with additional adjustment for diagnosis-todetection time interval.

## Data Availability

The data used in the present study are available from the UK Biobank with restrictions applied. Access to the UK Biobank data can be requested through a standard protocol (https://www.ukbiobank.ac.uk/register-apply/). The data were used under license and are thus not publicly available.

## Abbreviations

AD: Alzheimer's disease
ACD: All-cause dementia
AUC: Area under the curve
BMI: Body mass index
C-index: Concordance index
DIPK2B: Divergent protein kinase domain 2B
FDR: False discovery rate
GFAP: Glial fibrillary acidic protein
GO: Gene ontology
HR: Hazard ratio
KLK3: kallikrein-related peptidase 3
VaD: Vascular dementia
