# Supplementary material for: Plasma proteomic signatures as predictors of dementia risk in individuals with sleep apnea: a cohort study
Source: Transl Neurodegener. 2025 May 22;14:25. doi: 10.1186/s40035-025-00485-6 (PMC12096503; doi:10.1186/s40035-025-00485-6)
Supplement: Supplementary file 1 — Additional file 1. Figure S1. Study design and analysis workflow. Figure S2. Sex subgroup analysis of the predictive accuracy of KLK3 protein for incident dementia. Figure S3. GO pathway enrichment analysis. Figure S4. Associations between plasma proteins and dementia risk in individuals with neither sleep apnea nor snoring. Table S1. Baseline characteristics of the cohort investigating the relationship between sleep apnea and dementia risk. Table S2. Associations between sleep apnea and incident all-cause dementia, Alzheimer's disease, and vascular dementia. Table S3. Sex subgroup analysis of the associations between sleep apnea and incident dementia. Table S4. BMI subgroup analysis of the associations between sleep apnea and incident dementia. Table S5. Baseline characteristics of the proteomics study. Table S6. Associations between plasma proteins and dementia risk in sleep apnea patients. Table S7. C-index for plasma proteins. Table S8. Associations of plasma proteins with dementia risk among male and female sleep apnea patients. Table S9. Associations of plasma proteins with dementia risk with additional adjustment for diagnosis-todetection time interval. [file 40035_2025_485_MOESM1_ESM.pdf]

Additional file 1

Plasma Proteomic Signatures as Predictors of Dementia Risk in Individuals with Sleep Apnea: A cohort study

Figure S1. Study design and analysis workflow

Figure S2. Sex subgroup analysis of the predictive accuracy of KLK3 protein for incident dementia

Figure S3. GO pathway enrichment analysis

Figure S4. Associations between plasma proteins and dementia risk in individuals with neither sleep apnea nor snoring

Table S1. Baseline characteristics of the cohort investigating the relationship between sleep apnea and dementia risk

Table S2. Associations between sleep apnea and incident all-cause dementia, Alzheimer's disease, and vascular dementia

Table S3. Sex subgroup analysis of the associations between sleep apnea and incident dementia

Table S4. BMI subgroup analysis of the associations between sleep apnea and incident dementia

Table S5. Baseline characteristics of the proteomics study

Table S6. Associations between plasma proteins and dementia risk in sleep apnea patients

Table S7. C-index for plasma proteins

Table S8. Associations of plasma proteins with dementia risk among male and female sleep apnea patients

Table S9. Associations of plasma proteins with dementia risk with additional adjustment for diagnosis-to-detection time interval

Figure S1. Study design and analysis workflow

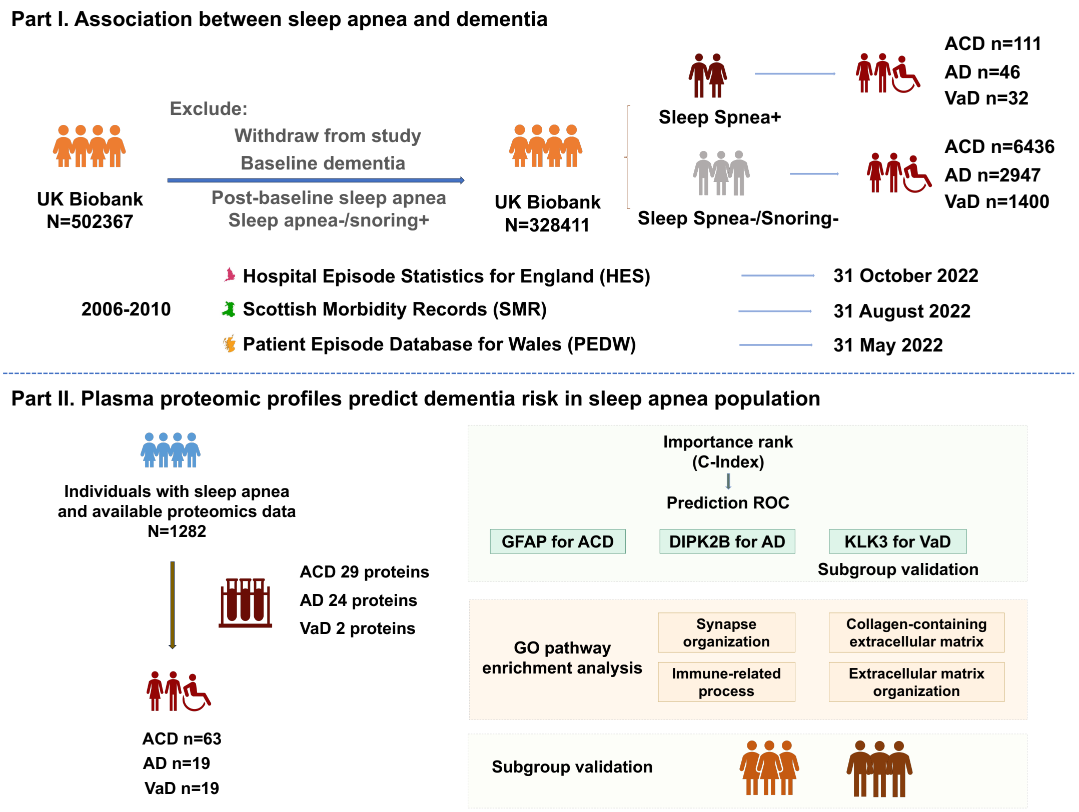

The flowchart outlines the two main components of the study. The first part excludes individuals who had withdrawn, had baseline dementia, had developed sleep apnea after baseline, or had snoring without sleep apnea, yielding a population-based primary analysis of 328,411 UK Biobank participants. This segment investigates the association between sleep apnea and dementia risk. The second part focuses on 1282 sleep apnea participants with available proteomic data. Proteins associated with ACD, AD, and VaD are identified, followed by C-index calculations, ranking of protein importance for dementia prediction, Gene Ontology (GO) pathway enrichment analysis, and subgroup validation. AD, Alzheimer's disease; ACD, All-cause dementia; VaD, Vascular dementia; GO, Gene Ontology.

Figure S2. Sex subgroup analysis of the predictive accuracy of KLK3 protein for incident dementia

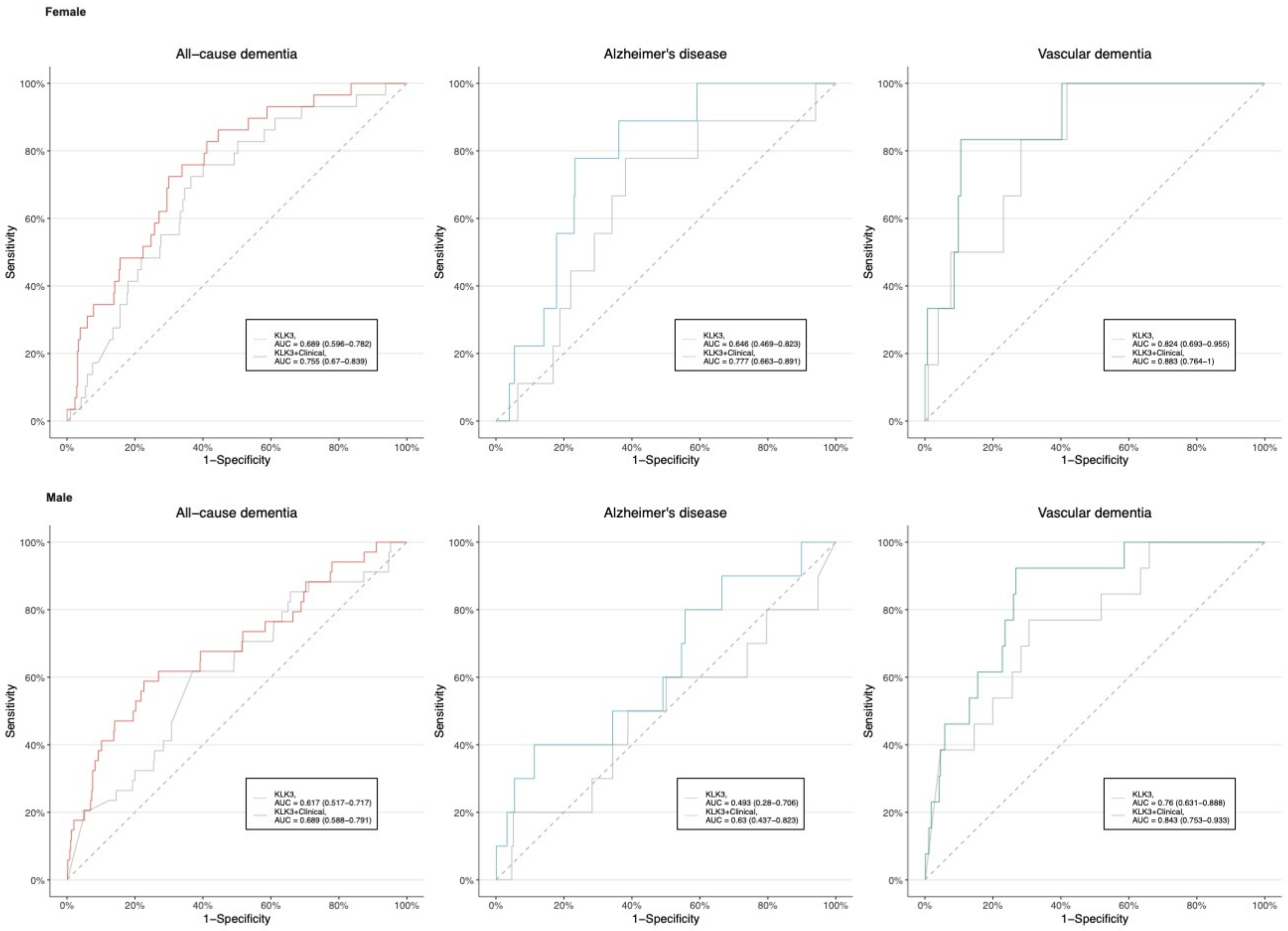

Abbreviation: AUC, Area Under the ROC Curve.

Figure S3. GO pathway enrichment analysis

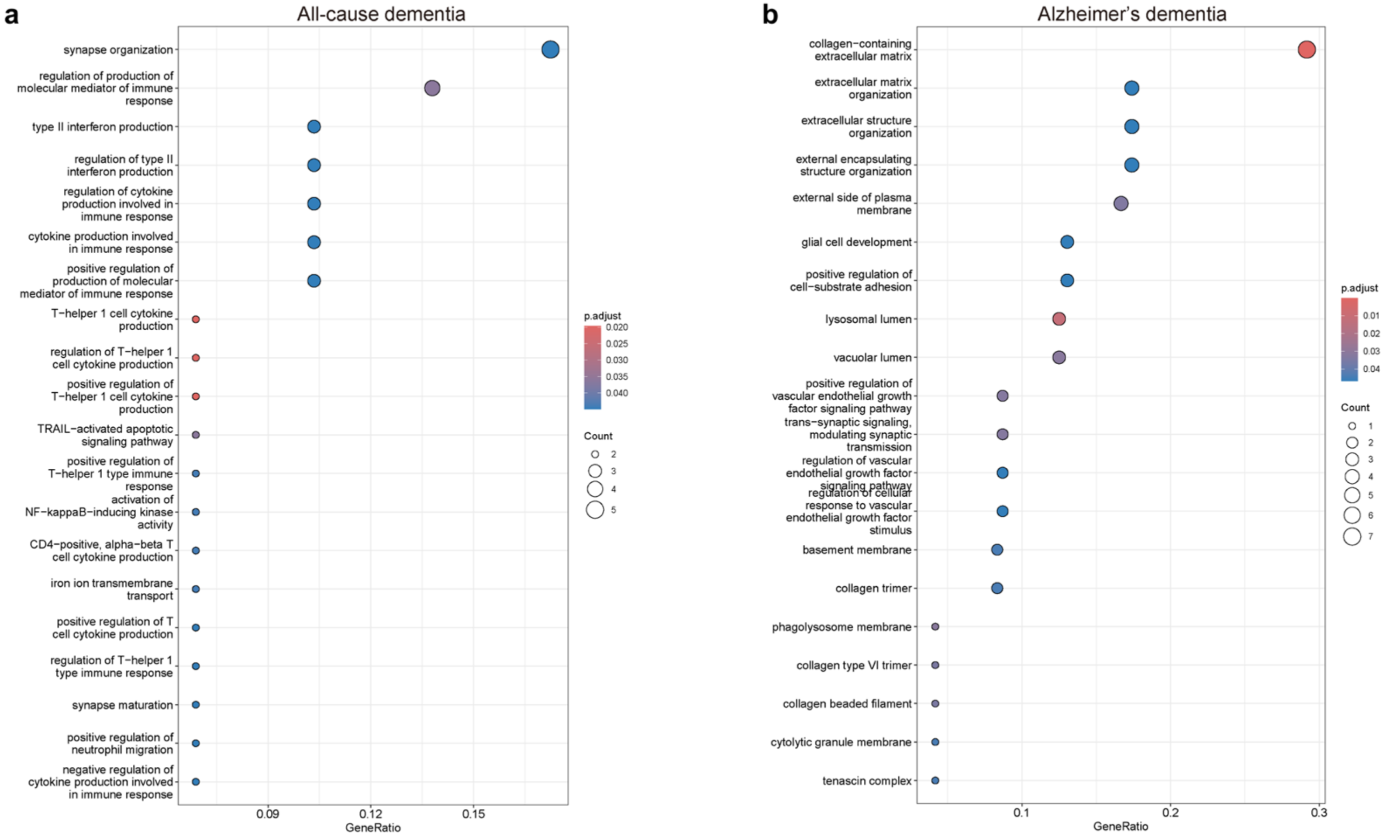

(a) All-cause dementia; (b) Alzheimer's disease. Significant proteins, identified after Bonferroni correction through Cox proportional hazards models, are shown. The x-axis represents the gene ratio, while the y-axis lists the enriched GO pathways. Circle size indicates the number of proteins per pathway, and color reflects the adjusted *p*-value (*p*.adjust) according to the key provided. Corrected *p*.adjust < 0.05 was considered significant. GO, Gene Ontology.

Figure S4. Associations between plasma proteins and dementia risk in individuals with neither sleep apnea nor snoring

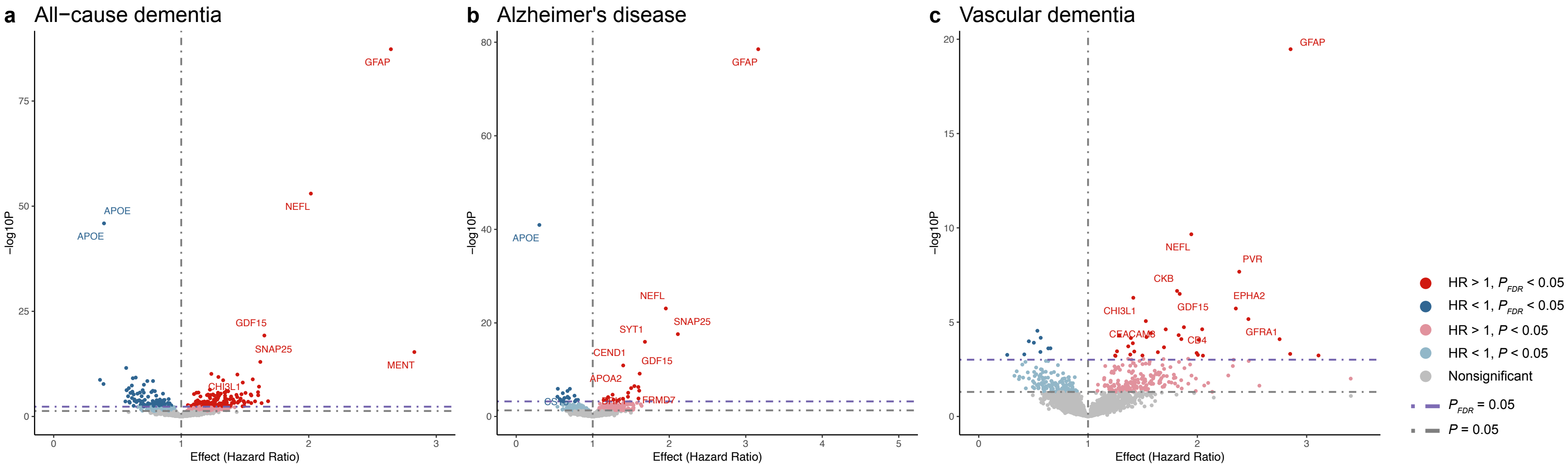

Cox model adjusted for age, sex, smoking status, alcohol consumption, body mass index, education level, ethnic, and Townsend deprivation index.

Table S1. Baseline characteristics of the cohort investigating the relationship between sleep apnea and dementia risk

	Total population	Neither sleep apnea nor snoring	Sleep apnea
No. of participants	328411	324977	3434
Age, years, mean (SD)	56.42 (8.25)	56.40 (8.26)	57.84 (7.40)
Sex, female, n (%)	201765 (61.44)	200931 (61.83)	834 (24.29)
Ethnicity, white, n (%)	308349 (93.89)	305156 (93.90)	3193 (92.98)
Body mass index, kg/m^2, mean (SD)	26.76 (4.55)	26.70 (4.48)	32.53 (6.57)
Education level, higher, n (%)	108737 (33.11)	107726 (33.15)	1011 (29.44)
Smoking status, n (%)			
Never	186279 (56.72)	184820 (56.87)	1459 (42.49)
Previous	107463 (32.72)	105984 (32.61)	1479 (43.07)
Current	32400 (9.87)	31932 (9.83)	468 (13.63)
Alcohol consumption, n (%)			
Never	16241 (4.95)	16083 (4.95)	158 (4.60)
Previous	12593 (3.83)	12383 (3.81)	210 (6.12)
Current	298106 (90.77)	295054 (90.79)	3052 (88.88)
Townsend deprivation index, mean (SD)	-1.23 (3.13)	-1.23 (3.12)	-0.69 (3.26)

Descriptive statistics were performed, with means and standard deviations provided for continuous variables and frequencies with percentages for categorical variables. Abbreviation: SD, standard deviation.

Table S2. Associations between sleep apnea and incident all-cause dementia, Alzheimer's disease, and vascular dementia

		Model 1		Model 2	
	**Case/Total population**	**HR (95%CI)**	***P***	**HR (95%CI)**	***P***
**ACD**					
Neither sleep apnea nor snoring	6436/324977	1 (Reference)		1 (Reference)	
Sleep apnea	111/3434	1.46 (1.21-1.77)	<0.001	1.40 (1.16-1.69)	<0.001
**AD**					
Neither sleep apnea nor snoring	2947/324977	1 (Reference)		1 (Reference)	
Sleep apnea	46/3434	1.42 (1.06-1.90)	0.019	1.46 (1.09-1.96)	0.012
**VaD**					
Neither sleep apnea nor snoring	1400/324977	1 (Reference)		1 (Reference)	
Sleep apnea	32/3434	1.80 (1.27-2.57)	0.001	1.52 (1.06-2.16)	0.022

Model 1 is adjusted for age and sex. Model 2 is adjusted for Model 1 plus smoking status, alcohol consumption, body mass index, education level, ethnicity, and Townsend deprivation index. ACD, All-cause dementia; AD, Alzheimer's disease; VaD, Vascular dementia.

Table S3. Sex subgroup analysis of the associations between sleep apnea and incident dementia

			Model 1		Model 2	
		**Case/Total population**	**HR (95%CI)**	***P***	**HR (95%CI)**	***P***
All-cause dementia						
Female	Neither sleep apnea nor snoring	3495/200931	1 (Reference)		1 (Reference)	
	Sleep apnea	25/834	1.73 (1.17-2.56)	0.006	1.58 (1.06-2.34)	0.024
Male	Neither sleep apnea nor snoring	2941/124046	1 (Reference)		1 (Reference)	
	Sleep apnea	86/2600	1.40 (1.13-1.73)	0.002	1.34 (1.08-1.67)	0.008
Alzheimer's disease						
Female	Neither sleep apnea nor snoring	1736/200931	1 (Reference)		1 (Reference)	
	Sleep apnea	12/834	1.67 (0.94-2.94)	0.078	1.66 (0.94-2.93)	0.082
Male	Neither sleep apnea nor snoring	1986/221014	1 (Reference)		1 (Reference)	
	Sleep apnea	90/7780	1.35 (0.96-1.89)	0.087	1.37 (0.97-1.93)	0.077
Vascular dementia						
Female	Neither sleep apnea nor snoring	681/200931	1 (Reference)		1 (Reference)	
	Sleep apnea	5/834	1.76 (0.73-4.25)	0.206	1.40 (0.58-3.40)	0.451
Male	Neither sleep apnea nor snoring	719/124046	1 (Reference)		1 (Reference)	
	Sleep apnea	27/2600	1.81 (1.23-2.65)	0.003	1.53 (1.03-2.26)	0.036

Model 1 adjusted for age and sex. Model 2 adjusted for model 1 plus smoking status, alcohol consumption, body mass index, education level, ethnic, and Townsend deprivation index.

Table S4. BMI subgroup analysis of the associations between sleep apnea and incident dementia

			Model 1		Model 2	
		**Case/Total population**	**HR (95%CI)**	***P***	**HR (95%CI)**	***P***
All-cause dementia						
BMI≤25 kg/m^2	Neither sleep apnea nor snoring	2165/126447	1 (Reference)		1 (Reference)	
	Sleep apnea	5/280	0.67 (0.28-1.62)	0.379	0.71 (0.30-1.71)	0.449
BMI>25 kg/m^2	Neither sleep apnea nor snoring	4271/198530	1 (Reference)		1 (Reference)	
	Sleep apnea	106/3154	1.55 (1.28-1.88)	<0.001	1.41 (1.16-1.72)	<0.001
Alzheimer's disease						
BMI≤25 kg/m^2	Neither sleep apnea nor snoring	1046/126447	1 (Reference)		1 (Reference)	
	Sleep apnea	3/280	0.88 (0.28-2.75)	0.83	0.95 (0.30-2.94)	0.924
BMI>25 kg/m^2	Neither sleep apnea nor snoring	1901/198530	1 (Reference)		1 (Reference)	
	Sleep apnea	43/3154	1.50 (1.10-2.03)	0.009	1.46 (1.08-1.99)	0.015
Vascular dementia						
BMI≤25 kg/m^2	Neither sleep apnea nor snoring	398/126447	1 (Reference)		1 (Reference)	
	Sleep apnea	1/280	0.68 (0.10-4.84)	0.7	0.74 (0.10-5.30)	0.768
BMI>25 kg/m^2	Neither sleep apnea nor snoring	1002/198530	1 (Reference)		1 (Reference)	
	Sleep apnea	31/3154	1.83 (1.28-2.62)	<0.001	1.51 (1.05-2.18)	0.026

Model 1 adjusted for age and sex. Model 2 adjusted for model 1 plus smoking status, alcohol consumption, body mass index, education level, ethnic, and Townsend deprivation index. BMI, body mass index.

Table S5. Baseline characteristics of the proteomics study

	Total population	Incident ACD	Incident AD	Incident VaD
No. of participants	1282	63	19	19
Age, years, mean (SD)	57.21 (7.83)	60.71 (7.54)	60.79 (8.00)	62.00 (7.22)
Sex, female, n (%)	413 (32.22)	29 (46.03)	9 (47.37)	6 (31.58)
Ethnicity, white, n (%)	1183 (92.28)	60 (95.24)	18 (94.74)	18 (94.74)
Body mass index, kg/m^2, mean (SD)	32.59 (6.56)	32.51 (6.13)	32.50 (8.10)	33.85 (3.81)
Education level, higher, n (%)	338 (26.37)	8 (12.70)	3 (15.79)	1 (5.26)
Smoking status, n (%)				
Never	594 (46.33)	31 (49.21)	11 (57.89)	5 (26.32)
Previous	518 (40.41)	25 (39.68)	6 (31.58)	11 (57.89)
Current	159 (12.40)	6 (9.52)	2 (10.53)	3 (15.79)
Alcohol consumption, n (%)				
Never	82 (6.40)	9 (14.29)	2 (10.53)	1 (5.26)
Previous	84 (6.55)	5 (7.94)	3 (15.79)	2 (10.53)
Current	1111 (86.66)	49 (77.78)	14 (73.68)	16 (84.21)
Townsend deprivation index, mean (SD)	-0.53 (3.35)	0.29 (3.21)	0.92 (3.46)	1.04 (3.13)

Descriptive statistics were conducted, with means and standard deviations for continuous variables and frequencies with percentages for categorical variables. SD, standard deviation; ACD, All-cause dementia; AD, Alzheimer's disease; VaD, Vascular dementia.

Table S6. Associations between plasma proteins and dementia risk in sleep apnea patients

	All-cause dementia (Case/total: 63/1282)			Alzheimer's disease (Case/total: 19/1282)			Vascular dementia (Case/total: 19/1282)		
**Proteins**	**HR (95% CI)**	***P* value**	**P_{FDR}**	**HR (95% CI)**	***P***	**P_{FDR}**	**HR (95% CI)**	***P***	**P_{FDR}**
A1BG	0.91 (0.24-3.48)	0.890	0.999	1.55 (0.12-19.81)	0.736	0.997	0.87 (0.07-10.68)	0.912	0.992
AAMDC	0.96 (0.59-1.56)	0.868	0.998	1.14 (0.48-2.69)	0.767	0.997	1.06 (0.45-2.49)	0.895	0.992
AARSD1	1.13 (0.78-1.65)	0.514	0.946	0.92 (0.47-1.81)	0.809	0.997	1.33 (0.71-2.48)	0.376	0.941
ABCA2	1.57 (0.78-3.20)	0.209	0.740	5.21 (1.93-14.12)	0.001	0.066	1.66 (0.43-6.36)	0.463	0.942
ABHD14B	0.90 (0.60-1.36)	0.622	0.966	0.99 (0.47-2.07)	0.972	0.997	1.13 (0.55-2.34)	0.742	0.983
ABL1	1.12 (0.82-1.52)	0.492	0.936	1.52 (0.88-2.61)	0.133	0.597	0.89 (0.48-1.67)	0.717	0.976
ABO	1.04 (0.87-1.24)	0.697	0.975	0.86 (0.61-1.21)	0.391	0.893	1.12 (0.80-1.57)	0.520	0.948
ABRAXAS2	0.87 (0.59-1.28)	0.478	0.928	1.25 (0.64-2.46)	0.509	0.942	0.65 (0.31-1.36)	0.255	0.901
ACAA1	1.10 (0.86-1.41)	0.432	0.906	1.14 (0.73-1.78)	0.556	0.962	1.27 (0.82-1.98)	0.291	0.915
ACADM	0.93 (0.61-1.42)	0.742	0.984	0.97 (0.46-2.05)	0.945	0.997	0.68 (0.27-1.69)	0.405	0.942
ACADSB	0.89 (0.65-1.22)	0.465	0.922	1.05 (0.60-1.82)	0.866	0.997	0.63 (0.34-1.17)	0.141	0.808
ACAN	0.98 (0.45-2.14)	0.959	0.999	1.80 (0.42-7.74)	0.430	0.908	0.86 (0.21-3.47)	0.827	0.991
ACE	1.04 (0.43-2.53)	0.931	0.999	6.00 (1.11-32.43)	0.037	0.333	1.65 (0.32-8.52)	0.551	0.955
ACE2	1.08 (0.77-1.52)	0.657	0.971	1.11 (0.60-2.05)	0.736	0.997	1.13 (0.61-2.11)	0.693	0.975
ACHE	1.06 (0.55-2.05)	0.866	0.998	1.19 (0.36-3.98)	0.773	0.997	1.39 (0.44-4.41)	0.572	0.960
ACOT13	0.96 (0.77-1.19)	0.688	0.975	0.97 (0.65-1.46)	0.900	0.997	0.73 (0.50-1.07)	0.111	0.763
ACOX1	0.71 (0.45-1.14)	0.160	0.680	0.69 (0.29-1.64)	0.400	0.901	0.72 (0.31-1.70)	0.457	0.942
ACP1	0.64 (0.35-1.16)	0.139	0.652	0.48 (0.16-1.49)	0.206	0.712	0.87 (0.31-2.45)	0.797	0.990
ACP5	1.23 (0.63-2.41)	0.543	0.948	0.83 (0.24-2.87)	0.771	0.997	0.64 (0.18-2.22)	0.479	0.944
ACP6	1.30 (0.92-1.86)	0.138	0.648	1.19 (0.64-2.20)	0.592	0.970	1.65 (0.87-3.12)	0.125	0.795
ACRBP	0.75 (0.40-1.40)	0.362	0.865	1.55 (0.58-4.16)	0.381	0.886	0.55 (0.16-1.87)	0.340	0.935

ACRV1	0.90 (0.70-1.16)	0.429	0.904	1.05 (0.67-1.65)	0.839	0.997	0.90 (0.58-1.38)	0.615	0.966
ACSL1	1.21 (0.63-2.33)	0.559	0.954	1.47 (0.56-3.85)	0.434	0.908	1.57 (0.69-3.57)	0.281	0.913
ACTA2	1.42 (1.05-1.93)	0.023	0.346	1.44 (0.84-2.49)	0.188	0.680	1.68 (1.01-2.80)	0.045	0.638
ACTN2	1.21 (0.89-1.65)	0.218	0.746	1.39 (0.80-2.43)	0.239	0.742	0.90 (0.46-1.75)	0.757	0.985
ACTN4	1.36 (0.84-2.22)	0.210	0.741	1.06 (0.36-3.18)	0.911	0.997	0.93 (0.25-3.43)	0.913	0.992
ACVRL1	2.24 (1.21-4.15)	0.010	0.238	2.96 (1.12-7.80)	0.028	0.295	2.54 (0.93-6.92)	0.069	0.718
ACY1	0.80 (0.57-1.12)	0.191	0.718	0.93 (0.52-1.67)	0.814	0.997	0.92 (0.51-1.66)	0.791	0.989
ACY3	0.95 (0.60-1.50)	0.827	0.995	1.32 (0.63-2.77)	0.457	0.923	1.42 (0.70-2.85)	0.331	0.934
ACYP1	0.80 (0.54-1.19)	0.273	0.799	1.03 (0.52-2.06)	0.927	0.997	0.86 (0.43-1.73)	0.674	0.975
ADA	1.52 (0.85-2.70)	0.156	0.676	1.79 (0.67-4.78)	0.248	0.753	3.06 (1.26-7.43)	0.013	0.437
ADA2	1.54 (0.98-2.42)	0.059	0.466	1.26 (0.55-2.89)	0.582	0.967	1.13 (0.48-2.64)	0.777	0.987
ADAM12	1.77 (1.03-3.05)	0.040	0.413	2.25 (0.99-5.11)	0.053	0.392	2.36 (1.06-5.27)	0.037	0.623
ADAM15	1.37 (0.69-2.73)	0.373	0.873	1.80 (0.54-5.98)	0.338	0.842	1.52 (0.43-5.29)	0.515	0.947
ADAM22	1.07 (0.60-1.91)	0.819	0.995	2.26 (0.79-6.45)	0.127	0.589	0.81 (0.29-2.28)	0.692	0.975
ADAM23	1.43 (0.73-2.79)	0.293	0.811	4.30 (1.17-15.77)	0.028	0.292	1.08 (0.33-3.51)	0.904	0.992
ADAM8	1.29 (0.60-2.79)	0.511	0.945	2.99 (0.78-11.49)	0.110	0.546	0.63 (0.15-2.73)	0.537	0.952
ADAM9	2.00 (0.96-4.15)	0.063	0.472	2.18 (0.62-7.66)	0.225	0.736	5.42 (2.00-14.72)	<0.001	0.180
ADAMTS1	1.89 (0.86-4.17)	0.113	0.602	2.31 (0.58-9.29)	0.236	0.742	2.57 (0.75-8.78)	0.133	0.796
ADAMTS13	1.44 (0.59-3.48)	0.421	0.899	0.68 (0.15-3.21)	0.629	0.984	1.37 (0.27-6.92)	0.700	0.975
ADAMTS15	2.41 (1.43-4.08)	<0.001	0.074	3.23 (1.24-8.42)	0.017	0.235	2.89 (1.12-7.45)	0.028	0.588
ADAMTS16	1.38 (0.86-2.22)	0.176	0.706	1.76 (1.06-2.91)	0.028	0.291	1.10 (0.28-4.26)	0.893	0.992
ADAMTS4	2.19 (1.09-4.40)	0.027	0.362	2.26 (0.70-7.32)	0.174	0.660	2.73 (0.92-8.07)	0.069	0.718
ADAMTS8	1.42 (0.88-2.28)	0.151	0.673	2.31 (0.95-5.61)	0.063	0.428	1.77 (0.72-4.36)	0.212	0.867
ADAMTSL2	1.46 (0.81-2.65)	0.211	0.741	4.10 (1.52-11.03)	0.005	0.146	0.93 (0.30-2.89)	0.905	0.992
ADAMTSL4	3.55 (1.37-9.20)	0.009	0.230	2.81 (0.47-16.68)	0.255	0.766	7.21 (1.27-41.06)	0.026	0.582

ADAMTSL5	0.76 (0.41-1.41)	0.378	0.875	0.93 (0.30-2.83)	0.898	0.997	0.78 (0.26-2.35)	0.661	0.975
ADCYAP1R1	0.99 (0.48-2.06)	0.979	0.999	0.97 (0.24-3.93)	0.962	0.997	1.77 (0.78-4.00)	0.173	0.853
ADD1	1.02 (0.72-1.45)	0.920	0.999	0.86 (0.45-1.64)	0.644	0.991	0.98 (0.52-1.86)	0.946	0.992
ADGRB3	2.02 (1.12-3.64)	0.019	0.311	1.35 (0.46-3.97)	0.582	0.967	2.66 (0.92-7.68)	0.071	0.718
ADGRD1	1.16 (0.61-2.20)	0.658	0.971	3.52 (1.17-10.59)	0.025	0.279	1.50 (0.48-4.73)	0.485	0.944
ADGRE1	1.50 (0.99-2.28)	0.057	0.461	2.15 (1.07-4.31)	0.031	0.308	1.27 (0.56-2.87)	0.569	0.960
ADGRE2	1.65 (0.82-3.33)	0.161	0.681	2.98 (0.90-9.88)	0.075	0.465	1.49 (0.40-5.57)	0.553	0.955
ADGRE5	1.89 (1.01-3.51)	0.045	0.426	2.26 (0.79-6.48)	0.129	0.590	1.42 (0.44-4.60)	0.557	0.955
ADGRF5	0.70 (0.32-1.55)	0.383	0.876	2.28 (0.54-9.53)	0.260	0.772	0.20 (0.04-0.93)	0.040	0.630
ADGRG1	1.16 (0.94-1.43)	0.179	0.706	1.24 (0.85-1.81)	0.261	0.772	0.90 (0.58-1.39)	0.620	0.967
ADGRG2	0.68 (0.32-1.45)	0.322	0.838	2.83 (0.65-12.35)	0.166	0.649	0.41 (0.11-1.52)	0.184	0.861
ADGRV1	0.94 (0.63-1.40)	0.769	0.988	0.92 (0.45-1.88)	0.824	0.997	0.51 (0.21-1.23)	0.135	0.796
ADH1B	0.78 (0.58-1.06)	0.111	0.598	0.84 (0.50-1.42)	0.514	0.945	0.63 (0.35-1.15)	0.133	0.796
ADH4	0.72 (0.56-0.94)	0.017	0.298	0.78 (0.49-1.25)	0.299	0.806	0.63 (0.39-1.03)	0.066	0.717
ADIPOQ	1.22 (0.88-1.69)	0.225	0.757	1.64 (0.89-3.04)	0.115	0.563	2.00 (1.06-3.77)	0.032	0.610
ADM	2.56 (1.18-5.58)	0.018	0.303	2.39 (0.59-9.66)	0.221	0.729	5.60 (1.27-24.72)	0.023	0.553
ADRA2A	1.20 (0.84-1.70)	0.310	0.826	1.04 (0.51-2.13)	0.910	0.997	0.97 (0.44-2.12)	0.939	0.992
AFAP1	1.10 (0.81-1.50)	0.545	0.948	0.72 (0.38-1.36)	0.306	0.816	1.29 (0.76-2.19)	0.349	0.938
AFM	0.67 (0.20-2.28)	0.524	0.948	3.36 (0.35-31.77)	0.291	0.803	1.53 (0.17-14.05)	0.709	0.976
AFP	1.00 (0.70-1.43)	0.997	0.999	0.64 (0.33-1.22)	0.176	0.662	1.90 (1.00-3.62)	0.050	0.670
AGBL2	1.12 (0.85-1.48)	0.415	0.894	1.07 (0.64-1.80)	0.798	0.997	1.42 (0.86-2.34)	0.173	0.853
AGER	1.44 (0.87-2.38)	0.153	0.673	3.07 (1.28-7.36)	0.012	0.206	1.09 (0.44-2.67)	0.859	0.992
AGR2	1.22 (0.98-1.52)	0.079	0.514	1.10 (0.73-1.64)	0.648	0.992	1.36 (0.90-2.06)	0.145	0.808
AGR3	1.41 (0.99-2.01)	0.058	0.463	1.13 (0.58-2.22)	0.719	0.995	2.07 (1.17-3.66)	0.013	0.437
AGRN	1.70 (1.06-2.73)	0.027	0.362	2.32 (1.14-4.73)	0.021	0.257	2.14 (1.04-4.43)	0.040	0.629

AGRP	1.36 (0.92-2.02)	0.123	0.620	1.24 (0.60-2.58)	0.565	0.963	1.64 (0.78-3.46)	0.193	0.865
AGT	1.48 (0.59-3.74)	0.405	0.894	2.54 (0.71-9.10)	0.152	0.626	1.30 (0.18-9.61)	0.798	0.990
AGXT	0.86 (0.65-1.13)	0.279	0.804	0.82 (0.48-1.39)	0.464	0.926	0.96 (0.57-1.59)	0.864	0.992
AHCY	1.05 (0.75-1.46)	0.793	0.994	1.08 (0.59-1.98)	0.805	0.997	0.99 (0.53-1.83)	0.967	0.994
AHNAK	0.73 (0.32-1.67)	0.452	0.916	1.97 (0.58-6.68)	0.278	0.788	0.75 (0.17-3.30)	0.700	0.975
AHNAK2	1.18 (0.47-2.99)	0.726	0.977	3.00 (0.80-11.24)	0.102	0.527	2.06 (0.45-9.39)	0.352	0.938
AHSA1	0.73 (0.47-1.12)	0.152	0.673	1.22 (0.71-2.11)	0.465	0.926	0.46 (0.18-1.18)	0.105	0.763
AHSG	1.24 (0.47-3.28)	0.666	0.971	4.40 (0.75-25.78)	0.101	0.525	2.24 (0.40-12.62)	0.361	0.939
AHSP	1.13 (0.88-1.44)	0.342	0.854	0.87 (0.55-1.36)	0.528	0.948	1.20 (0.76-1.89)	0.432	0.942
AIDA	1.41 (1.12-1.78)	0.004	0.141	1.60 (1.13-2.25)	0.007	0.164	1.44 (0.97-2.13)	0.071	0.718
AIF1	1.05 (0.68-1.61)	0.835	0.997	1.29 (0.62-2.70)	0.500	0.941	0.90 (0.38-2.12)	0.811	0.991
AIF1L	1.25 (0.76-2.05)	0.378	0.875	0.99 (0.37-2.65)	0.986	0.998	2.03 (0.97-4.25)	0.061	0.698
AIFM1	0.97 (0.78-1.20)	0.755	0.986	0.98 (0.66-1.45)	0.908	0.997	0.91 (0.60-1.36)	0.633	0.973
AK1	1.17 (0.85-1.63)	0.332	0.847	1.01 (0.55-1.85)	0.971	0.997	1.25 (0.72-2.17)	0.430	0.942
AK2	1.06 (0.84-1.33)	0.643	0.971	1.01 (0.66-1.55)	0.950	0.997	0.82 (0.53-1.28)	0.392	0.942
AKAP12	1.23 (0.34-4.48)	0.748	0.984	6.08 (0.82-45.19)	0.078	0.471	1.44 (0.13-16.52)	0.771	0.987
AKR1B1	0.94 (0.69-1.30)	0.726	0.977	0.92 (0.51-1.67)	0.790	0.997	0.81 (0.44-1.52)	0.514	0.947
AKR1B10	1.02 (0.70-1.47)	0.933	0.999	0.73 (0.34-1.58)	0.421	0.905	1.06 (0.56-2.04)	0.851	0.992
AKR1C4	1.14 (0.80-1.61)	0.466	0.922	0.96 (0.52-1.78)	0.900	0.997	1.47 (0.91-2.36)	0.117	0.780
AKR7L	0.91 (0.63-1.32)	0.627	0.966	0.95 (0.49-1.82)	0.866	0.997	0.61 (0.29-1.30)	0.202	0.867
AKT1S1	1.12 (0.79-1.57)	0.521	0.947	0.95 (0.51-1.75)	0.858	0.997	1.48 (0.79-2.79)	0.220	0.869
AKT2	0.92 (0.75-1.13)	0.433	0.906	1.10 (0.77-1.59)	0.602	0.973	0.72 (0.49-1.04)	0.082	0.743
AKT3	0.81 (0.54-1.21)	0.299	0.817	0.66 (0.31-1.39)	0.276	0.788	0.80 (0.37-1.72)	0.563	0.957
ALCAM	2.19 (0.81-5.92)	0.123	0.621	3.03 (0.51-18.10)	0.224	0.736	1.08 (0.16-7.23)	0.934	0.992
ALDH1A1	0.97 (0.67-1.39)	0.849	0.998	0.65 (0.32-1.32)	0.238	0.742	1.16 (0.62-2.18)	0.640	0.973

ALDH2	0.85 (0.55-1.32)	0.480	0.930	0.35 (0.10-1.19)	0.093	0.510	1.10 (0.58-2.11)	0.770	0.987
ALDH3A1	1.14 (0.86-1.50)	0.369	0.871	1.00 (0.57-1.76)	0.994	0.998	1.31 (0.80-2.15)	0.276	0.910
ALDH5A1	0.72 (0.48-1.09)	0.121	0.619	0.84 (0.41-1.72)	0.637	0.987	0.68 (0.30-1.53)	0.353	0.938
ALMS1	1.15 (0.74-1.77)	0.537	0.948	1.46 (0.71-3.01)	0.307	0.816	0.68 (0.25-1.87)	0.455	0.942
ALPI	1.10 (0.90-1.34)	0.370	0.871	1.05 (0.73-1.53)	0.781	0.997	1.04 (0.71-1.51)	0.854	0.992
ALPP	1.01 (0.82-1.25)	0.905	0.999	0.68 (0.47-0.99)	0.043	0.362	1.59 (1.10-2.31)	0.014	0.451
AMBN	1.49 (0.61-3.66)	0.379	0.875	3.76 (1.35-10.42)	0.011	0.199	0.63 (0.11-3.66)	0.608	0.966
AMBP	2.01 (0.76-5.35)	0.161	0.680	1.88 (0.34-10.50)	0.472	0.932	3.48 (0.64-18.97)	0.149	0.813
AMDHD2	1.12 (0.41-3.09)	0.827	0.995	4.50 (0.68-29.93)	0.120	0.573	0.20 (0.04-1.09)	0.063	0.704
AMFR	0.79 (0.53-1.16)	0.230	0.762	1.08 (0.55-2.11)	0.830	0.997	0.61 (0.27-1.37)	0.227	0.878
AMIGO1	1.07 (0.62-1.86)	0.810	0.995	1.35 (0.53-3.43)	0.524	0.948	0.79 (0.24-2.56)	0.695	0.975
AMIGO2	1.71 (0.71-4.15)	0.233	0.763	2.06 (0.44-9.75)	0.360	0.862	1.34 (0.26-7.01)	0.731	0.978
AMN	1.35 (0.86-2.11)	0.193	0.719	1.08 (0.46-2.52)	0.861	0.997	0.95 (0.41-2.21)	0.912	0.992
AMOT	1.71 (1.01-2.90)	0.048	0.430	1.19 (0.41-3.49)	0.750	0.997	2.90 (1.23-6.86)	0.015	0.480
AMOTL2	1.23 (0.89-1.70)	0.208	0.740	1.02 (0.52-2.00)	0.956	0.997	1.68 (0.99-2.87)	0.057	0.698
AMPD3	1.17 (0.84-1.62)	0.348	0.856	0.94 (0.52-1.68)	0.831	0.997	1.31 (0.75-2.30)	0.340	0.935
AMY1A_AMY1B_AMY1C	0.41 (0.25-0.68)	<0.001	0.055	0.47 (0.19-1.15)	0.097	0.520	0.80 (0.33-1.96)	0.621	0.967
AMY2A	0.51 (0.33-0.80)	0.003	0.132	0.66 (0.30-1.44)	0.292	0.803	0.69 (0.31-1.50)	0.342	0.936
AMY2B	0.58 (0.37-0.90)	0.016	0.294	0.63 (0.29-1.36)	0.242	0.744	0.72 (0.33-1.56)	0.407	0.942
ANG	1.15 (0.61-2.16)	0.659	0.971	1.03 (0.32-3.34)	0.961	0.997	1.14 (0.35-3.67)	0.826	0.991
ANGPT1	0.91 (0.69-1.19)	0.474	0.925	0.87 (0.53-1.42)	0.566	0.963	0.88 (0.54-1.45)	0.619	0.967
ANGPT2	1.66 (1.00-2.77)	0.050	0.436	2.45 (1.03-5.87)	0.044	0.363	2.08 (0.85-5.12)	0.110	0.763
ANGPTL1	1.18 (0.57-2.47)	0.654	0.971	2.95 (0.83-10.52)	0.095	0.517	2.00 (0.51-7.86)	0.320	0.926
ANGPTL2	1.63 (0.96-2.76)	0.070	0.496	2.80 (1.10-7.15)	0.031	0.310	1.56 (0.59-4.14)	0.368	0.939
ANGPTL3	1.01 (0.56-1.84)	0.975	0.999	1.84 (0.63-5.40)	0.266	0.774	0.63 (0.21-1.85)	0.397	0.942

ANGPTL4	1.32 (0.84-2.08)	0.233	0.763	1.19 (0.52-2.74)	0.686	0.993	2.00 (0.88-4.57)	0.100	0.762
ANGPTL7	1.72 (1.00-2.93)	0.049	0.430	2.20 (0.86-5.67)	0.102	0.527	2.54 (1.04-6.21)	0.041	0.630
ANK2	1.11 (0.90-1.39)	0.330	0.846	0.76 (0.42-1.36)	0.357	0.862	1.32 (0.93-1.88)	0.121	0.785
ANKMY2	0.94 (0.69-1.28)	0.697	0.975	1.27 (0.73-2.18)	0.395	0.897	0.66 (0.35-1.23)	0.188	0.862
ANKRA2	0.96 (0.75-1.22)	0.736	0.981	1.17 (0.78-1.75)	0.439	0.912	0.71 (0.42-1.22)	0.219	0.869
ANKRD54	1.41 (1.09-1.82)	0.009	0.220	0.96 (0.51-1.82)	0.903	0.997	1.22 (0.75-1.97)	0.421	0.942
ANP32C	0.62 (0.24-1.58)	0.312	0.829	0.59 (0.10-3.50)	0.562	0.962	0.39 (0.05-2.97)	0.366	0.939
ANPEP	1.32 (0.66-2.65)	0.431	0.904	2.34 (0.81-6.77)	0.116	0.563	0.42 (0.08-2.10)	0.292	0.915
ANXA1	0.97 (0.52-1.80)	0.913	0.999	1.30 (0.51-3.32)	0.586	0.967	0.91 (0.32-2.57)	0.861	0.992
ANXA10	0.97 (0.74-1.27)	0.836	0.997	1.02 (0.62-1.67)	0.953	0.997	1.19 (0.75-1.88)	0.464	0.942
ANXA11	0.95 (0.68-1.34)	0.780	0.992	0.93 (0.50-1.71)	0.811	0.997	0.79 (0.44-1.42)	0.432	0.942
ANXA2	1.08 (0.75-1.55)	0.677	0.974	1.49 (0.88-2.52)	0.136	0.600	0.69 (0.35-1.38)	0.293	0.915
ANXA3	0.99 (0.71-1.38)	0.948	0.999	0.97 (0.52-1.81)	0.932	0.997	0.83 (0.44-1.56)	0.557	0.955
ANXA4	0.89 (0.64-1.25)	0.514	0.946	1.08 (0.59-1.98)	0.802	0.997	0.62 (0.32-1.20)	0.153	0.816
ANXA5	2.54 (1.26-5.14)	0.009	0.231	2.16 (0.59-7.93)	0.245	0.749	4.05 (1.19-13.80)	0.025	0.582
AOC1	0.72 (0.46-1.14)	0.160	0.680	0.89 (0.42-1.92)	0.776	0.997	0.57 (0.22-1.47)	0.244	0.893
AOC3	1.49 (0.73-3.01)	0.270	0.799	4.73 (1.26-17.81)	0.022	0.261	0.95 (0.25-3.51)	0.933	0.992
AP1G2	1.18 (0.88-1.58)	0.272	0.799	1.71 (1.07-2.73)	0.026	0.281	0.96 (0.54-1.70)	0.894	0.992
AP2B1	0.76 (0.41-1.40)	0.380	0.875	1.01 (0.36-2.87)	0.980	0.998	0.41 (0.12-1.43)	0.161	0.838
AP3B1	1.06 (0.77-1.46)	0.728	0.978	1.42 (0.80-2.52)	0.225	0.736	0.98 (0.53-1.81)	0.945	0.992
AP3S2	0.83 (0.48-1.42)	0.493	0.936	1.16 (0.46-2.89)	0.758	0.997	0.59 (0.20-1.76)	0.344	0.936
APBB1IP	1.40 (0.89-2.18)	0.142	0.660	1.33 (0.57-3.10)	0.509	0.942	1.34 (0.59-3.03)	0.488	0.946
APCS	0.65 (0.31-1.35)	0.247	0.771	0.76 (0.20-2.94)	0.694	0.994	0.33 (0.10-1.11)	0.074	0.718
APEX1	1.05 (0.71-1.57)	0.794	0.994	1.04 (0.50-2.16)	0.924	0.997	0.71 (0.33-1.53)	0.380	0.942
APLP1	0.99 (0.65-1.50)	0.965	0.999	1.15 (0.53-2.48)	0.727	0.996	1.02 (0.48-2.18)	0.952	0.992

APOA1	0.69 (0.24-2.01)	0.495	0.938	1.18 (0.21-6.57)	0.847	0.997	0.97 (0.16-5.97)	0.976	0.994
APOA2	1.80 (1.12-2.88)	0.015	0.293	2.00 (0.85-4.70)	0.111	0.550	1.73 (0.76-3.92)	0.191	0.863
APOA4	0.93 (0.43-2.02)	0.860	0.998	2.09 (0.69-6.35)	0.192	0.690	1.31 (0.35-4.88)	0.692	0.975
APOB	2.06 (0.64-6.61)	0.224	0.757	4.73 (0.49-45.77)	0.180	0.664	1.78 (0.22-14.50)	0.588	0.965
APOBR	0.93 (0.54-1.59)	0.788	0.994	1.38 (0.50-3.82)	0.537	0.953	1.48 (0.52-4.20)	0.464	0.942
APOC1	0.70 (0.34-1.43)	0.332	0.847	0.41 (0.11-1.50)	0.178	0.662	1.47 (0.43-5.04)	0.541	0.952
APOD	0.79 (0.38-1.66)	0.541	0.948	1.70 (0.63-4.62)	0.296	0.803	0.45 (0.10-2.05)	0.304	0.923
APOE	0.70 (0.42-1.18)	0.181	0.708	0.39 (0.14-1.10)	0.075	0.465	1.20 (0.62-2.31)	0.585	0.965
APOF	0.76 (0.36-1.62)	0.479	0.929	0.33 (0.09-1.17)	0.085	0.486	0.70 (0.19-2.58)	0.588	0.965
APOH	0.82 (0.46-1.46)	0.503	0.943	0.89 (0.31-2.55)	0.824	0.997	0.89 (0.30-2.58)	0.823	0.991
APOL1	0.95 (0.61-1.48)	0.823	0.995	1.15 (0.54-2.44)	0.716	0.995	0.48 (0.18-1.24)	0.128	0.796
APOM	0.48 (0.24-0.96)	0.037	0.407	0.64 (0.18-2.27)	0.489	0.939	0.87 (0.24-3.06)	0.822	0.991
APP	0.96 (0.69-1.35)	0.825	0.995	1.19 (0.64-2.19)	0.584	0.967	0.75 (0.39-1.42)	0.374	0.940
APPL2	0.94 (0.76-1.18)	0.613	0.966	0.93 (0.62-1.38)	0.713	0.995	0.82 (0.55-1.24)	0.358	0.939
APRT	0.90 (0.56-1.45)	0.665	0.971	0.64 (0.28-1.43)	0.275	0.788	1.91 (0.73-5.00)	0.189	0.862
ARAF	1.34 (0.99-1.82)	0.056	0.460	1.45 (0.87-2.42)	0.151	0.626	1.18 (0.64-2.18)	0.591	0.965
AREG	2.08 (1.35-3.19)	<0.001	0.069	1.25 (0.52-3.01)	0.618	0.979	3.01 (1.47-6.20)	0.003	0.281
ARF6	1.04 (0.82-1.32)	0.716	0.975	1.33 (0.85-2.09)	0.209	0.712	1.02 (0.65-1.61)	0.922	0.992
ARFIP1	0.96 (0.66-1.39)	0.823	0.995	1.54 (0.82-2.89)	0.176	0.662	0.55 (0.26-1.19)	0.132	0.796
ARG1	1.22 (0.80-1.85)	0.347	0.855	1.02 (0.49-2.15)	0.953	0.997	1.02 (0.48-2.18)	0.958	0.994
ARG2	1.15 (0.85-1.56)	0.350	0.856	0.62 (0.24-1.58)	0.316	0.819	1.07 (0.58-1.97)	0.824	0.991
ARHGAP1	1.05 (0.65-1.71)	0.836	0.997	1.31 (0.54-3.21)	0.551	0.960	1.66 (0.65-4.25)	0.288	0.915
ARHGAP25	0.96 (0.65-1.43)	0.842	0.998	0.77 (0.36-1.69)	0.520	0.946	1.10 (0.56-2.14)	0.782	0.987
ARHGAP30	1.48 (0.83-2.64)	0.183	0.708	1.80 (0.71-4.57)	0.218	0.728	1.05 (0.25-4.40)	0.944	0.992
ARHGAP45	1.00 (0.74-1.36)	0.992	0.999	1.17 (0.67-2.02)	0.582	0.967	0.91 (0.51-1.62)	0.741	0.983

ARHGAP5	1.26 (0.92-1.71)	0.148	0.671	1.44 (0.88-2.37)	0.149	0.622	0.66 (0.30-1.46)	0.310	0.923
ARHGEF1	1.03 (0.78-1.37)	0.811	0.995	1.16 (0.70-1.95)	0.563	0.962	0.80 (0.47-1.37)	0.422	0.942
ARHGEF10	1.09 (0.83-1.43)	0.541	0.948	1.27 (0.79-2.05)	0.329	0.832	1.02 (0.61-1.70)	0.949	0.992
ARHGEF12	1.13 (0.91-1.40)	0.268	0.796	1.07 (0.72-1.60)	0.724	0.995	1.10 (0.73-1.68)	0.644	0.973
ARHGEF5	1.36 (0.97-1.92)	0.074	0.512	1.27 (0.67-2.38)	0.465	0.926	2.07 (1.26-3.39)	0.004	0.316
ARID3A	0.88 (0.44-1.74)	0.705	0.975	1.00 (0.29-3.47)	0.997	0.998	0.46 (0.11-1.87)	0.276	0.910
ARID4B	0.89 (0.49-1.60)	0.690	0.975	1.16 (0.50-2.67)	0.732	0.997	0.77 (0.23-2.55)	0.664	0.975
ARL13B	0.82 (0.53-1.25)	0.351	0.856	1.02 (0.48-2.15)	0.963	0.997	0.70 (0.30-1.64)	0.415	0.942
ARL2BP	1.00 (0.51-1.96)	0.996	0.999	2.48 (0.87-7.05)	0.088	0.493	0.31 (0.07-1.28)	0.105	0.763
ARMCX2	0.94 (0.66-1.36)	0.753	0.986	0.83 (0.41-1.66)	0.592	0.970	1.08 (0.59-1.98)	0.792	0.989
ARNT	1.71 (0.97-3.02)	0.062	0.472	1.22 (0.40-3.76)	0.729	0.997	0.87 (0.24-3.19)	0.837	0.991
ARNTL	1.59 (1.06-2.38)	0.024	0.350	2.04 (1.14-3.65)	0.017	0.235	1.36 (0.57-3.26)	0.494	0.947
ARSA	1.07 (0.68-1.69)	0.764	0.987	1.49 (0.69-3.19)	0.309	0.816	1.48 (0.65-3.37)	0.355	0.939
ARSB	0.81 (0.54-1.22)	0.317	0.834	1.05 (0.51-2.18)	0.887	0.997	0.43 (0.19-0.97)	0.042	0.633
ART3	0.73 (0.41-1.30)	0.282	0.806	0.84 (0.29-2.41)	0.749	0.997	0.97 (0.34-2.71)	0.946	0.992
ART5	1.07 (0.71-1.62)	0.748	0.984	0.98 (0.42-2.29)	0.962	0.997	0.99 (0.42-2.36)	0.982	0.996
ARTN	1.43 (0.96-2.12)	0.079	0.515	1.04 (0.41-2.62)	0.933	0.997	2.18 (1.21-3.92)	0.009	0.402
ASAH1	1.10 (0.65-1.86)	0.719	0.975	2.05 (1.21-3.45)	0.007	0.164	0.64 (0.21-1.92)	0.424	0.942
ASAH2	0.87 (0.61-1.24)	0.435	0.907	0.72 (0.43-1.23)	0.232	0.742	0.81 (0.45-1.47)	0.492	0.947
ASGR1	2.12 (1.16-3.87)	0.014	0.286	2.82 (1.04-7.64)	0.041	0.350	2.56 (0.91-7.19)	0.076	0.718
ASGR2	2.10 (0.92-4.80)	0.078	0.514	3.85 (1.01-14.69)	0.049	0.383	2.36 (0.57-9.79)	0.238	0.889
ASPN	1.80 (0.77-4.21)	0.177	0.706	3.28 (0.71-15.22)	0.130	0.590	2.16 (0.44-10.49)	0.342	0.936
ASPSCR1	0.84 (0.56-1.26)	0.402	0.894	1.12 (0.54-2.32)	0.762	0.997	0.63 (0.29-1.36)	0.239	0.889
ASRGL1	1.08 (0.84-1.38)	0.567	0.956	1.17 (0.74-1.85)	0.501	0.941	1.15 (0.72-1.83)	0.556	0.955
ASS1	0.81 (0.60-1.08)	0.145	0.666	0.82 (0.48-1.39)	0.459	0.923	0.84 (0.49-1.43)	0.511	0.947

ATF2	0.92 (0.68-1.26)	0.619	0.966	0.93 (0.52-1.67)	0.820	0.997	1.17 (0.74-1.87)	0.501	0.947
ATF4	1.03 (0.56-1.88)	0.930	0.999	1.55 (0.63-3.81)	0.336	0.839	0.54 (0.16-1.82)	0.318	0.923
ATG16L1	1.07 (0.81-1.41)	0.641	0.969	1.06 (0.64-1.77)	0.822	0.997	1.00 (0.59-1.68)	0.994	0.998
ATG4A	1.08 (0.77-1.54)	0.645	0.971	0.57 (0.28-1.17)	0.123	0.580	1.20 (0.68-2.10)	0.534	0.952
ATOX1	1.04 (0.74-1.48)	0.807	0.995	1.27 (0.66-2.41)	0.476	0.932	0.84 (0.43-1.64)	0.619	0.967
ATP1B1	1.10 (0.68-1.77)	0.705	0.975	1.05 (0.45-2.46)	0.916	0.997	1.24 (0.59-2.62)	0.569	0.960
ATP1B2	1.22 (0.56-2.63)	0.615	0.966	2.99 (1.55-5.78)	0.001	0.066	0.72 (0.14-3.78)	0.701	0.975
ATP1B3	0.89 (0.45-1.75)	0.731	0.979	1.48 (0.64-3.41)	0.362	0.864	0.31 (0.05-1.83)	0.197	0.867
ATP1B4	1.15 (0.68-1.93)	0.604	0.962	1.33 (0.53-3.35)	0.546	0.959	0.90 (0.31-2.66)	0.854	0.992
ATP2B4	1.27 (0.90-1.79)	0.171	0.697	1.16 (0.57-2.36)	0.675	0.993	1.03 (0.47-2.29)	0.936	0.992
ATP5F1D	1.13 (0.71-1.79)	0.604	0.962	1.31 (0.65-2.63)	0.448	0.918	0.86 (0.33-2.22)	0.756	0.985
ATP5IF1	1.06 (0.86-1.31)	0.584	0.959	1.05 (0.71-1.55)	0.805	0.997	0.90 (0.61-1.34)	0.607	0.966
ATP5PO	0.99 (0.58-1.70)	0.973	0.999	0.84 (0.30-2.34)	0.737	0.997	1.44 (0.62-3.37)	0.401	0.942
ATP6AP2	1.47 (0.89-2.43)	0.128	0.626	2.17 (1.11-4.22)	0.023	0.269	1.33 (0.53-3.32)	0.547	0.954
ATP6V1D	0.52 (0.22-1.20)	0.126	0.625	0.39 (0.09-1.67)	0.205	0.711	0.41 (0.09-1.83)	0.241	0.891
ATP6V1F	1.10 (0.80-1.53)	0.548	0.949	0.97 (0.53-1.78)	0.933	0.997	1.42 (0.81-2.47)	0.219	0.869
ATP6V1G1	0.78 (0.56-1.09)	0.150	0.673	1.14 (0.64-2.03)	0.665	0.993	0.58 (0.30-1.10)	0.093	0.762
ATP6V1G2	1.13 (0.91-1.41)	0.280	0.804	1.50 (1.06-2.12)	0.021	0.257	0.78 (0.50-1.21)	0.268	0.910
ATRAID	1.85 (1.32-2.60)	<0.001	0.046	1.79 (1.00-3.22)	0.051	0.391	2.09 (1.32-3.29)	0.002	0.228
ATRN	0.59 (0.14-2.50)	0.472	0.924	0.79 (0.06-11.11)	0.858	0.997	0.61 (0.04-8.61)	0.715	0.976
ATXN10	0.84 (0.61-1.17)	0.303	0.821	0.89 (0.48-1.63)	0.700	0.994	0.90 (0.49-1.66)	0.742	0.983
ATXN2	1.14 (0.94-1.38)	0.195	0.720	1.20 (0.89-1.64)	0.236	0.742	1.27 (0.92-1.74)	0.140	0.808
ATXN2L	0.90 (0.53-1.54)	0.707	0.975	1.12 (0.43-2.88)	0.815	0.997	0.88 (0.30-2.58)	0.813	0.991
ATXN3	1.01 (0.69-1.48)	0.953	0.999	1.09 (0.56-2.11)	0.801	0.997	1.32 (0.71-2.46)	0.389	0.942
AXIN1	1.13 (0.90-1.43)	0.292	0.809	1.02 (0.67-1.56)	0.924	0.997	1.26 (0.81-1.95)	0.309	0.923

AXL	0.94 (0.44-1.98)	0.862	0.998	1.66 (0.44-6.18)	0.453	0.920	0.64 (0.16-2.55)	0.523	0.948
AZI2	0.87 (0.55-1.36)	0.534	0.948	1.17 (0.54-2.54)	0.685	0.993	0.62 (0.24-1.59)	0.318	0.923
AZU1	1.17 (0.90-1.54)	0.244	0.771	0.87 (0.48-1.58)	0.648	0.992	1.09 (0.65-1.83)	0.740	0.983
B2M	1.81 (1.21-2.71)	0.004	0.147	2.09 (1.15-3.80)	0.015	0.230	2.52 (1.45-4.36)	<0.001	0.180
B3GAT3	1.43 (0.39-5.25)	0.590	0.961	5.04 (0.86-29.46)	0.073	0.460	0.84 (0.07-10.20)	0.894	0.992
B3GNT7	1.92 (1.11-3.33)	0.019	0.313	4.03 (1.40-11.56)	0.010	0.191	0.99 (0.37-2.68)	0.988	0.997
B4GALT1	1.33 (0.62-2.85)	0.461	0.920	3.82 (1.24-11.80)	0.020	0.252	2.04 (0.54-7.72)	0.293	0.915
B4GAT1	1.34 (0.55-3.28)	0.522	0.948	0.77 (0.16-3.80)	0.750	0.997	1.76 (0.37-8.46)	0.481	0.944
BABAM1	1.04 (0.38-2.80)	0.944	0.999	1.97 (0.45-8.58)	0.367	0.871	0.11 (0.01-0.98)	0.048	0.664
BACH1	1.14 (0.84-1.53)	0.407	0.894	0.88 (0.50-1.55)	0.650	0.992	1.39 (0.81-2.37)	0.232	0.884
BAG3	0.81 (0.48-1.36)	0.418	0.898	2.21 (0.77-6.37)	0.141	0.604	1.30 (0.44-3.88)	0.632	0.973
BAG4	0.90 (0.52-1.56)	0.705	0.975	1.26 (0.51-3.13)	0.617	0.979	0.79 (0.28-2.25)	0.658	0.975
BAG6	1.46 (0.80-2.68)	0.218	0.746	1.26 (0.41-3.83)	0.684	0.993	1.49 (0.50-4.44)	0.470	0.942
BAIAP2	1.09 (0.79-1.49)	0.607	0.963	1.49 (0.88-2.51)	0.139	0.604	0.75 (0.39-1.45)	0.396	0.942
BAMBI	1.77 (0.98-3.21)	0.058	0.463	1.14 (0.35-3.72)	0.829	0.997	2.54 (1.02-6.30)	0.044	0.638
BANK1	1.08 (0.87-1.34)	0.472	0.924	1.01 (0.69-1.49)	0.951	0.997	1.01 (0.68-1.51)	0.949	0.992
BAP18	1.17 (0.83-1.65)	0.356	0.859	1.36 (0.77-2.39)	0.292	0.803	1.11 (0.59-2.08)	0.742	0.983
BATF	1.07 (0.78-1.46)	0.666	0.971	1.03 (0.56-1.89)	0.931	0.997	1.36 (0.80-2.29)	0.251	0.901
BAX	1.00 (0.78-1.30)	0.971	0.999	0.98 (0.61-1.57)	0.936	0.997	0.99 (0.60-1.63)	0.967	0.994
BCAM	2.47 (1.02-5.99)	0.045	0.426	5.75 (1.15-28.76)	0.033	0.318	3.13 (0.70-13.98)	0.134	0.796
BCAN	0.51 (0.28-0.94)	0.030	0.372	0.50 (0.17-1.47)	0.207	0.712	0.44 (0.15-1.29)	0.136	0.796
BCAT1	1.48 (0.68-3.24)	0.321	0.838	2.67 (0.88-8.09)	0.082	0.484	1.39 (0.31-6.17)	0.662	0.975
BCAT2	1.20 (0.86-1.69)	0.284	0.806	0.95 (0.40-2.26)	0.904	0.997	1.55 (1.15-2.08)	0.004	0.316
BCHE	1.91 (0.76-4.83)	0.170	0.697	3.42 (0.60-19.41)	0.165	0.647	1.17 (0.22-6.19)	0.852	0.992
BCL2	0.91 (0.55-1.50)	0.699	0.975	1.09 (0.47-2.50)	0.845	0.997	1.36 (0.63-2.93)	0.437	0.942

BCL2L1	0.93 (0.72-1.20)	0.598	0.962	1.12 (0.70-1.77)	0.643	0.991	0.72 (0.44-1.18)	0.192	0.863
BCL2L11	1.77 (0.96-3.26)	0.066	0.476	1.20 (0.42-3.39)	0.735	0.997	1.92 (0.59-6.23)	0.278	0.910
BCL2L15	1.29 (0.88-1.90)	0.187	0.712	1.82 (1.04-3.19)	0.036	0.326	1.15 (0.55-2.38)	0.710	0.976
BCL7A	1.42 (1.03-1.94)	0.030	0.372	1.26 (0.70-2.28)	0.446	0.917	0.96 (0.46-2.01)	0.912	0.992
BCL7B	0.78 (0.40-1.52)	0.469	0.924	0.85 (0.26-2.75)	0.784	0.997	0.60 (0.14-2.56)	0.494	0.947
BCR	1.05 (0.83-1.34)	0.666	0.971	0.94 (0.60-1.47)	0.791	0.997	1.02 (0.64-1.62)	0.934	0.992
BDNF	0.89 (0.67-1.18)	0.407	0.894	0.99 (0.58-1.69)	0.970	0.997	0.84 (0.50-1.41)	0.505	0.947
BECN1	0.87 (0.56-1.34)	0.517	0.947	1.16 (0.57-2.38)	0.685	0.993	0.83 (0.38-1.84)	0.649	0.973
BEX3	1.11 (0.74-1.68)	0.609	0.964	1.60 (1.00-2.58)	0.052	0.392	0.58 (0.19-1.81)	0.347	0.936
BGLAP	1.38 (1.07-1.80)	0.015	0.287	2.30 (1.38-3.82)	0.001	0.072	1.31 (0.82-2.08)	0.261	0.910
BGN	1.13 (0.93-1.38)	0.224	0.757	1.42 (1.01-2.02)	0.047	0.373	1.13 (0.78-1.63)	0.519	0.948
BHLHE40	0.86 (0.56-1.33)	0.499	0.939	1.51 (0.86-2.66)	0.153	0.626	0.68 (0.29-1.57)	0.364	0.939
BHMT2	2.89 (1.17-7.14)	0.021	0.338	4.83 (1.29-18.14)	0.020	0.252	1.39 (0.19-10.10)	0.747	0.985
BID	0.87 (0.60-1.26)	0.473	0.924	0.84 (0.42-1.65)	0.605	0.975	0.86 (0.43-1.74)	0.676	0.975
BIN2	1.03 (0.81-1.32)	0.796	0.994	1.05 (0.67-1.65)	0.842	0.997	0.92 (0.57-1.47)	0.719	0.976
BIRC2	0.99 (0.68-1.43)	0.948	0.999	0.75 (0.37-1.53)	0.430	0.908	0.89 (0.44-1.80)	0.745	0.984
BLMH	0.69 (0.34-1.42)	0.314	0.830	0.70 (0.19-2.64)	0.600	0.973	0.98 (0.28-3.42)	0.971	0.994
BLNK	0.60 (0.27-1.34)	0.212	0.742	1.07 (0.32-3.58)	0.912	0.997	0.63 (0.15-2.60)	0.527	0.949
BLOC1S2	0.99 (0.71-1.40)	0.976	0.999	1.39 (0.88-2.18)	0.157	0.633	1.17 (0.64-2.14)	0.602	0.966
BLOC1S3	0.80 (0.41-1.55)	0.512	0.945	1.20 (0.38-3.76)	0.760	0.997	0.44 (0.11-1.72)	0.236	0.885
BLVRB	1.06 (0.78-1.45)	0.692	0.975	0.71 (0.38-1.34)	0.290	0.801	1.23 (0.74-2.04)	0.428	0.942
BMP10	1.46 (0.64-3.35)	0.366	0.869	7.78 (1.63-37.04)	0.010	0.195	1.52 (0.33-6.88)	0.589	0.965
BMP4	0.78 (0.48-1.27)	0.321	0.838	1.07 (0.46-2.50)	0.874	0.997	1.34 (0.60-3.01)	0.479	0.944
BMP6	1.09 (0.63-1.88)	0.754	0.986	1.79 (0.70-4.58)	0.226	0.737	0.75 (0.27-2.07)	0.581	0.964
BMPER	2.21 (0.89-5.46)	0.086	0.539	4.32 (0.95-19.69)	0.058	0.407	2.91 (0.63-13.32)	0.170	0.853

BNIP2	0.99 (0.42-2.30)	0.975	0.999	2.05 (0.50-8.37)	0.319	0.823	0.27 (0.05-1.32)	0.105	0.763
BNIP3L	1.29 (0.75-2.22)	0.349	0.856	1.25 (0.47-3.33)	0.660	0.992	2.23 (1.01-4.92)	0.046	0.643
BOC	1.95 (0.73-5.18)	0.182	0.708	4.14 (0.68-25.25)	0.123	0.581	0.89 (0.14-5.70)	0.906	0.992
BOLA1	1.17 (0.81-1.70)	0.406	0.894	0.65 (0.29-1.46)	0.296	0.803	1.60 (0.89-2.88)	0.116	0.780
BOLA2_BOLA2B	0.81 (0.52-1.25)	0.341	0.854	0.97 (0.44-2.14)	0.945	0.997	1.07 (0.49-2.36)	0.863	0.992
BPIFA2	1.00 (0.80-1.25)	0.990	0.999	0.92 (0.62-1.37)	0.679	0.993	1.19 (0.79-1.77)	0.406	0.942
BPIFB1	1.14 (0.82-1.58)	0.448	0.915	0.49 (0.26-0.92)	0.027	0.288	1.46 (0.81-2.62)	0.205	0.867
BPIFB2	1.20 (0.89-1.61)	0.233	0.763	0.98 (0.56-1.71)	0.938	0.997	1.77 (1.11-2.84)	0.017	0.497
BRAP	0.93 (0.74-1.19)	0.573	0.956	0.98 (0.64-1.52)	0.945	0.997	0.84 (0.54-1.31)	0.442	0.942
BRD1	1.23 (0.50-2.99)	0.650	0.971	0.65 (0.11-3.84)	0.636	0.987	0.67 (0.12-3.89)	0.658	0.975
BRD2	1.46 (0.76-2.80)	0.255	0.783	1.02 (0.26-3.98)	0.974	0.997	1.44 (0.40-5.12)	0.575	0.960
BRD3	1.55 (0.81-2.97)	0.183	0.708	2.19 (0.80-5.96)	0.127	0.589	0.99 (0.26-3.74)	0.988	0.997
BRDT	1.04 (0.51-2.09)	0.922	0.999	0.57 (0.15-2.14)	0.409	0.903	1.22 (0.33-4.50)	0.762	0.987
BRK1	1.64 (1.01-2.65)	0.045	0.426	2.40 (1.04-5.55)	0.040	0.349	1.87 (0.77-4.58)	0.168	0.852
BRME1	1.00 (0.60-1.66)	0.994	0.999	1.06 (0.42-2.69)	0.900	0.997	1.52 (0.72-3.21)	0.268	0.910
BRSK2	0.96 (0.50-1.84)	0.899	0.999	1.27 (0.44-3.63)	0.656	0.992	0.44 (0.10-1.81)	0.252	0.901
BSG	2.23 (0.93-5.37)	0.072	0.503	4.27 (1.16-15.68)	0.029	0.295	2.31 (0.49-10.81)	0.289	0.915
BSND	1.58 (1.08-2.31)	0.019	0.311	0.57 (0.14-2.35)	0.434	0.909	2.34 (1.44-3.80)	<0.001	0.174
BST1	1.35 (0.77-2.35)	0.292	0.810	1.40 (0.49-4.02)	0.530	0.949	0.95 (0.42-2.12)	0.895	0.992
BST2	1.62 (1.24-2.11)	<0.001	0.046	1.93 (1.27-2.94)	0.002	0.089	1.41 (0.75-2.66)	0.290	0.915
BTC	0.95 (0.70-1.29)	0.752	0.986	0.77 (0.42-1.43)	0.409	0.903	1.11 (0.64-1.91)	0.708	0.976
BTD	0.51 (0.15-1.72)	0.280	0.804	0.20 (0.02-1.65)	0.134	0.600	0.46 (0.05-3.99)	0.479	0.944
BTLA	1.11 (0.80-1.54)	0.532	0.948	1.29 (0.77-2.17)	0.333	0.839	1.08 (0.59-1.98)	0.809	0.991
BTN1A1	0.73 (0.32-1.63)	0.437	0.907	1.06 (0.29-3.85)	0.931	0.997	0.41 (0.08-2.10)	0.286	0.915
BTN2A1	1.94 (0.97-3.86)	0.060	0.466	2.36 (0.76-7.38)	0.139	0.604	2.09 (0.64-6.83)	0.225	0.877

BTN3A2	1.59 (0.93-2.72)	0.088	0.545	2.31 (0.93-5.73)	0.071	0.456	1.24 (0.46-3.33)	0.671	0.975
BTNL10	0.93 (0.37-2.36)	0.878	0.999	2.60 (0.82-8.24)	0.103	0.528	0.22 (0.04-1.36)	0.104	0.763
C19orf12	0.79 (0.55-1.12)	0.180	0.707	0.84 (0.45-1.56)	0.580	0.967	0.71 (0.37-1.38)	0.315	0.923
C1GALT1C1	1.80 (1.00-3.22)	0.049	0.430	1.97 (0.80-4.85)	0.141	0.604	1.82 (0.55-6.02)	0.324	0.931
C1QA	1.32 (0.50-3.45)	0.576	0.956	1.70 (0.30-9.61)	0.551	0.960	5.51 (1.10-27.67)	0.038	0.623
C1QBP	1.06 (0.78-1.43)	0.718	0.975	1.01 (0.51-1.99)	0.975	0.997	0.61 (0.20-1.90)	0.398	0.942
C1QL2	1.28 (0.76-2.17)	0.352	0.856	1.95 (0.82-4.64)	0.129	0.590	1.27 (0.48-3.33)	0.630	0.972
C1QTNF1	1.97 (1.26-3.10)	0.003	0.132	1.52 (0.68-3.42)	0.308	0.816	1.82 (0.80-4.14)	0.152	0.816
C1QTNF5	2.35 (1.18-4.70)	0.015	0.293	7.11 (2.08-24.32)	0.002	0.084	3.67 (1.03-13.06)	0.045	0.638
C1QTNF6	1.19 (0.83-1.70)	0.342	0.854	1.30 (0.68-2.47)	0.430	0.908	1.20 (0.64-2.26)	0.572	0.960
C1QTNF9	1.32 (0.74-2.36)	0.343	0.854	6.24 (1.88-20.74)	0.003	0.110	1.23 (0.42-3.61)	0.709	0.976
C1R	0.71 (0.13-3.78)	0.686	0.975	1.16 (0.08-17.97)	0.913	0.997	0.84 (0.04-18.66)	0.914	0.992
C1RL	3.98 (1.04-15.26)	0.044	0.425	14.66 (1.13-190.83)	0.040	0.349	1.72 (0.16-18.89)	0.659	0.975
C1S	1.56 (0.42-5.86)	0.510	0.945	9.09 (0.78-105.87)	0.078	0.471	1.36 (0.12-15.27)	0.805	0.991
C2	1.05 (0.39-2.83)	0.930	0.999	0.79 (0.14-4.29)	0.780	0.997	1.54 (0.22-10.87)	0.666	0.975
C2CD2L	1.08 (0.61-1.93)	0.786	0.994	1.03 (0.36-2.91)	0.956	0.997	1.42 (0.56-3.60)	0.456	0.942
C2orf69	1.16 (0.74-1.83)	0.508	0.944	1.33 (0.60-2.94)	0.482	0.934	1.25 (0.62-2.52)	0.532	0.951
C3	0.59 (0.28-1.22)	0.151	0.673	0.97 (0.30-3.17)	0.960	0.997	0.34 (0.08-1.50)	0.155	0.822
C4BPB	1.27 (0.73-2.19)	0.393	0.888	1.00 (0.37-2.68)	0.996	0.998	1.15 (0.42-3.17)	0.781	0.987
C5	1.26 (0.28-5.69)	0.766	0.987	1.10 (0.07-18.38)	0.945	0.997	1.54 (0.17-13.84)	0.699	0.975
C7	1.61 (0.81-3.23)	0.177	0.706	3.20 (0.98-10.45)	0.055	0.393	0.33 (0.09-1.20)	0.091	0.760
C7orf50	0.41 (0.10-1.78)	0.236	0.767	0.92 (0.12-7.12)	0.937	0.997	0.21 (0.01-3.59)	0.279	0.910
C8B	1.17 (0.50-2.75)	0.716	0.975	0.98 (0.21-4.64)	0.982	0.998	0.59 (0.12-2.86)	0.516	0.947
C9	0.90 (0.58-1.40)	0.634	0.968	0.94 (0.42-2.14)	0.889	0.997	0.83 (0.38-1.80)	0.629	0.972
C9orf40	1.08 (0.81-1.44)	0.612	0.965	0.87 (0.52-1.47)	0.606	0.976	1.36 (0.83-2.23)	0.227	0.878

CA1	1.18 (0.83-1.69)	0.361	0.865	0.99 (0.53-1.85)	0.968	0.997	0.97 (0.51-1.86)	0.931	0.992
CA11	1.19 (0.64-2.20)	0.587	0.960	0.83 (0.21-3.24)	0.789	0.997	1.44 (0.53-3.91)	0.475	0.944
CA12	1.71 (0.97-3.01)	0.064	0.473	2.11 (0.78-5.69)	0.140	0.604	2.57 (0.92-7.13)	0.071	0.718
CA13	0.92 (0.74-1.15)	0.482	0.932	0.92 (0.62-1.36)	0.676	0.993	0.82 (0.55-1.23)	0.338	0.935
CA14	0.87 (0.50-1.51)	0.623	0.966	1.10 (0.42-2.84)	0.847	0.997	0.90 (0.33-2.44)	0.834	0.991
CA2	1.15 (0.86-1.54)	0.332	0.847	0.90 (0.53-1.53)	0.702	0.994	1.31 (0.79-2.15)	0.291	0.915
CA3	1.36 (0.99-1.87)	0.057	0.461	1.17 (0.66-2.09)	0.593	0.970	1.29 (0.74-2.25)	0.371	0.939
CA4	0.89 (0.37-2.17)	0.803	0.995	0.72 (0.14-3.62)	0.690	0.994	1.12 (0.23-5.48)	0.891	0.992
CA5A	0.96 (0.76-1.22)	0.751	0.986	1.05 (0.70-1.59)	0.800	0.997	0.97 (0.64-1.47)	0.872	0.992
CA6	0.92 (0.64-1.34)	0.678	0.974	1.42 (0.70-2.87)	0.328	0.832	1.13 (0.55-2.29)	0.745	0.984
CA7	1.30 (0.74-2.29)	0.356	0.859	1.09 (0.37-3.24)	0.874	0.997	1.27 (0.46-3.52)	0.643	0.973
CA8	1.84 (0.84-4.05)	0.128	0.626	2.28 (0.63-8.21)	0.208	0.712	0.22 (0.02-2.01)	0.179	0.853
CA9	1.50 (0.97-2.30)	0.066	0.474	1.50 (0.68-3.29)	0.314	0.818	1.49 (0.70-3.19)	0.298	0.917
CABP2	1.18 (0.79-1.78)	0.420	0.899	1.24 (0.59-2.60)	0.564	0.962	0.68 (0.25-1.84)	0.445	0.942
CACNA1C	0.97 (0.41-2.30)	0.943	0.999	1.85 (0.46-7.49)	0.389	0.892	0.55 (0.10-3.10)	0.495	0.947
CACNA1H	0.85 (0.47-1.52)	0.579	0.956	0.79 (0.25-2.45)	0.681	0.993	0.16 (0.03-0.75)	0.020	0.539
CACNB1	0.98 (0.65-1.47)	0.917	0.999	1.03 (0.49-2.15)	0.945	0.997	0.66 (0.26-1.66)	0.373	0.940
CACNB3	0.99 (0.73-1.35)	0.961	0.999	1.06 (0.60-1.87)	0.838	0.997	0.82 (0.45-1.49)	0.510	0.947
CACYBP	0.93 (0.72-1.20)	0.580	0.956	1.02 (0.65-1.62)	0.923	0.997	0.75 (0.47-1.21)	0.236	0.885
CADPS	0.90 (0.49-1.64)	0.729	0.978	0.72 (0.22-2.38)	0.587	0.967	0.48 (0.12-1.93)	0.301	0.919
CALB1	1.39 (0.84-2.30)	0.205	0.738	2.96 (1.26-6.95)	0.012	0.209	1.89 (0.77-4.64)	0.163	0.845
CALB2	1.52 (0.77-3.00)	0.233	0.763	1.22 (0.34-4.33)	0.758	0.997	1.98 (0.57-6.84)	0.281	0.913
CALCA	0.92 (0.67-1.26)	0.591	0.961	0.78 (0.42-1.46)	0.439	0.912	1.08 (0.62-1.88)	0.792	0.989
CALCB	1.67 (1.02-2.75)	0.043	0.421	0.98 (0.35-2.74)	0.969	0.997	2.18 (1.04-4.59)	0.039	0.625
CALCOCO1	1.14 (0.93-1.39)	0.222	0.755	0.96 (0.66-1.40)	0.838	0.997	1.21 (0.82-1.78)	0.346	0.936

CALCOCO2	0.93 (0.67-1.31)	0.691	0.975	1.00 (0.55-1.84)	0.989	0.998	0.93 (0.49-1.77)	0.820	0.991
CALY	1.46 (0.79-2.72)	0.229	0.762	2.45 (1.10-5.45)	0.028	0.292	0.42 (0.10-1.80)	0.242	0.892
CAMKK1	0.96 (0.55-1.66)	0.879	0.999	0.94 (0.35-2.50)	0.902	0.997	1.05 (0.38-2.87)	0.930	0.992
CAMLG	1.11 (0.71-1.72)	0.652	0.971	1.48 (0.77-2.85)	0.242	0.744	1.00 (0.43-2.36)	0.994	0.998
CAMSAP1	1.11 (0.88-1.39)	0.369	0.871	1.09 (0.72-1.65)	0.688	0.993	0.91 (0.58-1.42)	0.664	0.975
CANT1	1.60 (0.56-4.53)	0.379	0.875	3.07 (0.48-19.59)	0.235	0.742	0.46 (0.06-3.34)	0.441	0.942
CAPG	1.10 (0.74-1.63)	0.633	0.968	0.87 (0.42-1.80)	0.705	0.994	1.48 (0.79-2.77)	0.223	0.875
CAPN3	0.83 (0.49-1.41)	0.490	0.936	1.42 (0.64-3.17)	0.389	0.892	0.55 (0.17-1.77)	0.318	0.923
CAPS	1.26 (1.01-1.56)	0.041	0.416	1.23 (0.80-1.89)	0.345	0.852	1.16 (0.73-1.85)	0.521	0.948
CARHSP1	1.17 (0.81-1.67)	0.403	0.894	0.91 (0.46-1.82)	0.792	0.997	1.14 (0.59-2.20)	0.702	0.975
CASC3	0.86 (0.46-1.62)	0.637	0.968	0.91 (0.29-2.87)	0.867	0.997	0.58 (0.17-1.95)	0.375	0.941
CASP1	0.90 (0.62-1.32)	0.599	0.962	1.10 (0.56-2.15)	0.783	0.997	0.86 (0.43-1.70)	0.658	0.975
CASP10	0.93 (0.66-1.30)	0.657	0.971	1.27 (0.69-2.33)	0.439	0.912	1.03 (0.55-1.93)	0.930	0.992
CASP2	0.96 (0.68-1.35)	0.805	0.995	0.91 (0.48-1.73)	0.765	0.997	0.91 (0.47-1.75)	0.779	0.987
CASP3	0.99 (0.76-1.28)	0.929	0.999	1.09 (0.68-1.76)	0.721	0.995	0.88 (0.54-1.42)	0.602	0.966
CASP4	1.01 (0.86-1.19)	0.900	0.999	0.98 (0.72-1.32)	0.874	0.997	0.93 (0.68-1.27)	0.649	0.973
CASP7	0.95 (0.70-1.30)	0.762	0.987	1.26 (0.72-2.19)	0.419	0.903	0.66 (0.36-1.22)	0.189	0.862
CASP8	0.87 (0.59-1.27)	0.471	0.924	1.06 (0.54-2.10)	0.867	0.997	0.99 (0.50-1.96)	0.972	0.994
CASP9	0.97 (0.63-1.49)	0.884	0.999	0.91 (0.41-2.01)	0.807	0.997	0.72 (0.30-1.75)	0.474	0.944
CASQ2	1.35 (0.84-2.18)	0.212	0.742	1.80 (0.88-3.68)	0.108	0.543	0.83 (0.24-2.87)	0.766	0.987
CAT	0.89 (0.56-1.41)	0.619	0.966	0.76 (0.33-1.73)	0.509	0.942	1.13 (0.53-2.38)	0.756	0.985
CBLIF	1.05 (0.83-1.34)	0.681	0.975	1.13 (0.71-1.79)	0.615	0.979	1.00 (0.67-1.49)	0.999	1.000
CBLN1	0.98 (0.75-1.27)	0.857	0.998	0.82 (0.47-1.45)	0.493	0.940	0.81 (0.45-1.46)	0.481	0.944
CBLN4	1.05 (0.51-2.15)	0.892	0.999	3.20 (0.80-12.79)	0.100	0.525	0.77 (0.21-2.80)	0.692	0.975
CBS	0.76 (0.43-1.34)	0.337	0.852	1.15 (0.47-2.83)	0.756	0.997	0.56 (0.18-1.71)	0.308	0.923

CBX2	1.07 (0.61-1.88)	0.804	0.995	0.31 (0.06-1.73)	0.183	0.668	1.42 (0.59-3.43)	0.431	0.942
CC2D1A	1.14 (0.85-1.53)	0.394	0.890	0.99 (0.58-1.70)	0.971	0.997	1.09 (0.63-1.90)	0.752	0.985
CCAR2	0.96 (0.62-1.49)	0.849	0.998	1.37 (0.73-2.57)	0.326	0.831	0.42 (0.10-1.82)	0.249	0.901
CCDC134	0.99 (0.57-1.73)	0.986	0.999	1.63 (0.68-3.92)	0.278	0.788	0.30 (0.09-1.00)	0.050	0.670
CCDC28A	1.03 (0.44-2.40)	0.943	0.999	0.91 (0.20-4.23)	0.907	0.997	0.64 (0.14-2.82)	0.552	0.955
CCDC50	1.08 (0.81-1.44)	0.600	0.962	1.11 (0.66-1.87)	0.686	0.993	0.75 (0.38-1.50)	0.419	0.942
CCDC80	1.93 (1.20-3.10)	0.007	0.190	8.55 (3.57-20.47)	<0.001	0.002	1.09 (0.35-3.46)	0.878	0.992
CCER2	1.43 (1.01-2.04)	0.045	0.426	1.25 (0.65-2.39)	0.502	0.941	2.09 (1.09-4.00)	0.026	0.582
CCL11	1.99 (1.11-3.55)	0.021	0.326	1.87 (0.64-5.50)	0.253	0.761	1.44 (0.48-4.31)	0.512	0.947
CCL13	1.47 (1.07-2.01)	0.016	0.294	1.88 (1.07-3.30)	0.027	0.289	1.15 (0.64-2.04)	0.641	0.973
CCL14	1.23 (0.73-2.07)	0.446	0.914	0.94 (0.34-2.55)	0.901	0.997	0.75 (0.27-2.05)	0.570	0.960
CCL15	1.54 (0.97-2.43)	0.065	0.473	1.68 (0.75-3.75)	0.203	0.710	1.22 (0.51-2.87)	0.657	0.975
CCL16	2.02 (1.30-3.12)	0.002	0.097	1.78 (0.81-3.91)	0.151	0.626	1.86 (0.83-4.17)	0.130	0.796
CCL17	1.11 (0.88-1.41)	0.391	0.885	1.42 (0.94-2.15)	0.098	0.520	1.01 (0.67-1.52)	0.965	0.994
CCL18	1.18 (0.86-1.62)	0.314	0.830	1.74 (1.08-2.80)	0.023	0.268	0.93 (0.50-1.75)	0.828	0.991
CCL19	1.15 (0.99-1.34)	0.070	0.495	1.09 (0.81-1.46)	0.573	0.964	0.94 (0.67-1.31)	0.717	0.976
CCL2	1.32 (0.91-1.93)	0.146	0.669	1.30 (0.64-2.61)	0.467	0.926	1.05 (0.49-2.21)	0.908	0.992
CCL20	1.15 (0.93-1.42)	0.193	0.719	1.31 (0.91-1.87)	0.142	0.607	1.18 (0.78-1.77)	0.434	0.942
CCL21	1.23 (0.77-1.97)	0.391	0.884	2.23 (1.22-4.09)	0.009	0.188	0.79 (0.31-2.01)	0.623	0.967
CCL22	1.83 (1.29-2.59)	<0.001	0.066	2.32 (1.41-3.83)	<0.001	0.063	1.42 (0.69-2.94)	0.340	0.935
CCL23	1.10 (0.68-1.78)	0.695	0.975	1.09 (0.44-2.66)	0.855	0.997	1.66 (0.69-4.03)	0.259	0.907
CCL24	1.32 (1.02-1.70)	0.032	0.377	1.57 (0.99-2.49)	0.054	0.393	1.48 (0.94-2.31)	0.089	0.745
CCL25	1.56 (1.00-2.43)	0.052	0.443	2.44 (1.08-5.53)	0.033	0.314	1.47 (0.63-3.43)	0.374	0.940
CCL26	1.13 (0.94-1.36)	0.195	0.720	1.00 (0.64-1.56)	0.999	0.999	0.94 (0.60-1.46)	0.770	0.987
CCL27	1.22 (0.84-1.78)	0.288	0.809	2.74 (1.33-5.62)	0.006	0.153	1.12 (0.56-2.23)	0.755	0.985

CCL28	0.97 (0.64-1.46)	0.884	0.999	0.97 (0.46-2.05)	0.936	0.997	0.69 (0.31-1.52)	0.356	0.939
CCL3	1.15 (0.89-1.48)	0.299	0.817	1.37 (0.96-1.97)	0.082	0.484	0.88 (0.41-1.88)	0.739	0.983
CCL4	1.00 (0.74-1.35)	0.984	0.999	1.16 (0.73-1.83)	0.530	0.949	0.83 (0.44-1.57)	0.570	0.960
CCL5	1.00 (0.80-1.26)	0.983	0.999	1.25 (0.83-1.87)	0.283	0.794	0.88 (0.58-1.35)	0.568	0.960
CCL7	1.34 (0.98-1.84)	0.064	0.473	1.11 (0.59-2.07)	0.752	0.997	0.98 (0.50-1.90)	0.944	0.992
CCL8	0.87 (0.60-1.27)	0.473	0.925	0.96 (0.48-1.89)	0.895	0.997	0.56 (0.30-1.06)	0.074	0.718
CCN1	1.18 (0.86-1.63)	0.299	0.817	1.14 (0.64-2.03)	0.658	0.992	1.24 (0.68-2.26)	0.475	0.944
CCN2	0.91 (0.60-1.38)	0.655	0.971	0.90 (0.42-1.94)	0.794	0.997	1.10 (0.51-2.38)	0.807	0.991
CCN3	1.67 (0.99-2.81)	0.053	0.445	3.60 (1.50-8.64)	0.004	0.135	1.95 (0.84-4.55)	0.123	0.791
CCN4	1.67 (1.01-2.76)	0.046	0.428	1.62 (0.62-4.22)	0.327	0.832	2.19 (0.92-5.25)	0.078	0.730
CCN5	2.50 (1.51-4.16)	<0.001	0.046	3.98 (1.75-9.05)	<0.001	0.063	1.91 (0.77-4.74)	0.166	0.848
CCND2	1.01 (0.49-2.09)	0.981	0.999	5.93 (1.79-19.65)	0.004	0.121	0.96 (0.26-3.57)	0.956	0.992
CCNE1	1.17 (0.92-1.47)	0.195	0.720	1.15 (0.75-1.79)	0.517	0.945	1.24 (0.80-1.93)	0.340	0.935
CCS	0.83 (0.53-1.32)	0.436	0.907	0.76 (0.33-1.73)	0.509	0.942	0.91 (0.41-2.01)	0.822	0.991
CCT5	1.14 (0.69-1.90)	0.608	0.964	1.40 (0.58-3.35)	0.453	0.920	1.57 (0.65-3.76)	0.313	0.923
CD101	0.94 (0.58-1.53)	0.803	0.995	0.83 (0.37-1.88)	0.663	0.992	0.64 (0.33-1.25)	0.191	0.862
CD109	3.43 (1.77-6.62)	<0.001	0.046	2.91 (0.87-9.74)	0.083	0.484	3.53 (1.07-11.65)	0.039	0.623
CD14	1.71 (1.06-2.76)	0.028	0.366	1.76 (0.75-4.13)	0.198	0.702	2.08 (0.87-4.98)	0.101	0.762
CD160	1.31 (0.85-2.01)	0.217	0.746	1.15 (0.53-2.49)	0.718	0.995	1.53 (0.71-3.29)	0.275	0.910
CD163	1.19 (0.75-1.89)	0.455	0.920	1.31 (0.59-2.92)	0.508	0.942	1.40 (0.62-3.20)	0.421	0.942
CD164	1.54 (0.72-3.29)	0.261	0.788	1.80 (0.51-6.30)	0.358	0.862	0.86 (0.17-4.25)	0.853	0.992
CD164L2	1.21 (0.69-2.13)	0.499	0.939	0.53 (0.16-1.75)	0.294	0.803	1.84 (0.89-3.79)	0.100	0.762
CD177	1.03 (0.87-1.22)	0.756	0.986	1.08 (0.79-1.49)	0.614	0.979	1.09 (0.78-1.52)	0.623	0.967
CD1C	1.26 (0.52-3.05)	0.610	0.965	3.36 (0.58-19.56)	0.178	0.662	0.32 (0.09-1.21)	0.094	0.762
CD2	1.22 (0.76-1.97)	0.407	0.894	1.76 (1.00-3.09)	0.049	0.385	0.72 (0.16-3.17)	0.663	0.975

CD200	1.41 (0.63-3.18)	0.407	0.894	1.53 (0.34-6.78)	0.579	0.967	0.71 (0.16-3.25)	0.662	0.975
CD200R1	1.53 (0.84-2.78)	0.166	0.693	3.03 (1.20-7.70)	0.019	0.252	1.46 (0.47-4.57)	0.511	0.947
CD207	1.40 (0.81-2.40)	0.226	0.757	1.94 (0.76-5.00)	0.168	0.649	1.04 (0.38-2.83)	0.938	0.992
CD209	0.86 (0.47-1.54)	0.602	0.962	0.68 (0.23-1.97)	0.475	0.932	1.90 (0.63-5.68)	0.252	0.901
CD22	1.37 (0.85-2.23)	0.197	0.722	1.88 (0.79-4.47)	0.154	0.627	0.82 (0.33-2.03)	0.667	0.975
CD226	1.05 (0.58-1.91)	0.869	0.998	1.78 (0.63-5.03)	0.278	0.788	0.69 (0.22-2.18)	0.526	0.949
CD244	1.98 (0.93-4.24)	0.077	0.514	2.80 (0.74-10.51)	0.128	0.590	1.41 (0.33-6.00)	0.644	0.973
CD248	1.11 (0.60-2.04)	0.735	0.981	4.50 (1.69-12.01)	0.003	0.110	1.14 (0.39-3.38)	0.806	0.991
CD27	1.68 (1.07-2.63)	0.024	0.350	2.09 (0.99-4.43)	0.053	0.392	1.33 (0.58-3.07)	0.506	0.947
CD274	1.28 (0.71-2.30)	0.416	0.896	2.80 (1.16-6.76)	0.022	0.264	1.59 (0.53-4.80)	0.411	0.942
CD276	3.04 (1.67-5.53)	<0.001	0.046	3.23 (1.08-9.65)	0.036	0.326	2.79 (0.99-7.85)	0.052	0.680
CD28	1.54 (0.89-2.67)	0.121	0.619	3.07 (1.30-7.25)	0.010	0.195	1.30 (0.46-3.67)	0.622	0.967
CD2AP	1.34 (0.97-1.86)	0.079	0.514	1.16 (0.64-2.10)	0.624	0.983	1.30 (0.72-2.35)	0.389	0.942
CD300A	2.94 (1.46-5.90)	0.002	0.115	4.14 (1.42-12.05)	0.009	0.187	1.84 (0.46-7.29)	0.386	0.942
CD300C	2.10 (1.17-3.78)	0.013	0.278	3.11 (1.11-8.75)	0.031	0.310	1.41 (0.47-4.26)	0.543	0.952
CD300E	1.62 (1.03-2.57)	0.038	0.413	2.09 (0.99-4.43)	0.054	0.392	1.40 (0.58-3.41)	0.456	0.942
CD300LF	1.04 (0.72-1.51)	0.824	0.995	1.35 (0.68-2.66)	0.392	0.894	1.67 (0.82-3.41)	0.155	0.822
CD300LG	2.11 (1.25-3.57)	0.005	0.174	3.23 (1.32-7.91)	0.010	0.195	2.04 (0.85-4.89)	0.112	0.764
CD302	2.32 (1.34-4.02)	0.003	0.118	3.72 (1.54-8.98)	0.003	0.120	1.85 (0.64-5.29)	0.254	0.901
CD33	1.09 (0.82-1.44)	0.549	0.949	0.81 (0.50-1.31)	0.401	0.902	1.15 (0.68-1.94)	0.600	0.966
CD34	2.18 (0.90-5.30)	0.085	0.535	3.95 (0.79-19.84)	0.095	0.517	2.19 (0.46-10.33)	0.324	0.931
CD36	1.31 (0.76-2.25)	0.325	0.843	1.32 (0.48-3.59)	0.590	0.969	1.47 (0.56-3.88)	0.432	0.942
CD38	1.90 (1.07-3.38)	0.028	0.370	3.78 (1.51-9.47)	0.004	0.140	1.48 (0.52-4.21)	0.465	0.942
CD3D	1.04 (0.56-1.96)	0.894	0.999	1.22 (0.42-3.53)	0.716	0.995	0.98 (0.31-3.08)	0.967	0.994
CD3E	1.42 (0.93-2.17)	0.106	0.587	0.80 (0.26-2.50)	0.705	0.994	1.68 (0.83-3.41)	0.152	0.816

CD3G	1.08 (0.84-1.38)	0.550	0.949	1.02 (0.64-1.65)	0.926	0.997	0.95 (0.56-1.61)	0.860	0.992
CD4	1.65 (0.81-3.39)	0.169	0.697	2.16 (0.59-7.82)	0.242	0.744	2.46 (0.76-7.95)	0.132	0.796
CD40	1.24 (0.79-1.93)	0.352	0.857	1.39 (0.63-3.06)	0.411	0.903	1.04 (0.43-2.50)	0.932	0.992
CD40LG	0.75 (0.56-1.01)	0.061	0.468	0.67 (0.39-1.17)	0.158	0.633	0.75 (0.43-1.31)	0.308	0.923
CD46	1.26 (0.59-2.70)	0.555	0.950	2.27 (0.60-8.55)	0.227	0.738	0.95 (0.23-3.97)	0.949	0.992
CD48	2.13 (1.02-4.42)	0.044	0.425	6.24 (1.72-22.69)	0.005	0.146	1.32 (0.34-5.14)	0.689	0.975
CD5	1.42 (0.77-2.62)	0.259	0.787	1.81 (0.60-5.46)	0.290	0.802	1.13 (0.38-3.38)	0.827	0.991
CD55	1.02 (0.46-2.26)	0.957	0.999	2.08 (0.57-7.63)	0.268	0.777	0.76 (0.17-3.34)	0.719	0.976
CD58	2.38 (0.77-7.35)	0.133	0.635	8.05 (1.38-46.82)	0.020	0.256	1.78 (0.21-15.26)	0.600	0.966
CD59	1.66 (0.81-3.40)	0.168	0.693	1.73 (0.46-6.53)	0.417	0.903	1.34 (0.36-5.06)	0.664	0.975
CD5L	1.96 (1.15-3.34)	0.014	0.278	2.77 (1.08-7.09)	0.034	0.319	1.71 (0.64-4.58)	0.282	0.914
CD6	0.87 (0.57-1.32)	0.505	0.943	0.76 (0.35-1.63)	0.479	0.932	0.86 (0.41-1.81)	0.686	0.975
CD63	0.71 (0.46-1.09)	0.119	0.615	0.69 (0.32-1.51)	0.359	0.862	0.63 (0.29-1.37)	0.243	0.893
CD69	0.98 (0.75-1.29)	0.907	0.999	1.03 (0.62-1.72)	0.904	0.997	0.90 (0.53-1.52)	0.680	0.975
CD7	1.41 (0.82-2.44)	0.217	0.746	2.60 (1.26-5.34)	0.010	0.191	1.37 (0.53-3.57)	0.519	0.948
CD70	1.39 (0.80-2.40)	0.242	0.769	2.75 (1.02-7.38)	0.045	0.370	1.90 (0.72-5.04)	0.195	0.866
CD72	2.13 (1.38-3.28)	<0.001	0.055	2.97 (1.44-6.12)	0.003	0.116	2.11 (0.96-4.66)	0.065	0.710
CD74	1.49 (0.85-2.63)	0.167	0.693	2.30 (0.85-6.17)	0.099	0.524	1.81 (0.67-4.92)	0.242	0.893
CD79B	1.59 (1.03-2.46)	0.035	0.394	2.04 (0.96-4.33)	0.064	0.428	1.58 (0.70-3.55)	0.270	0.910
CD80	1.58 (0.85-2.93)	0.148	0.671	1.57 (0.51-4.83)	0.434	0.908	0.65 (0.20-2.16)	0.483	0.944
CD82	0.89 (0.48-1.64)	0.714	0.975	1.20 (0.49-2.94)	0.690	0.994	0.20 (0.04-1.01)	0.051	0.671
CD83	1.86 (1.04-3.35)	0.037	0.407	3.12 (1.16-8.38)	0.024	0.271	1.43 (0.50-4.11)	0.503	0.947
CD84	0.70 (0.33-1.48)	0.346	0.855	0.71 (0.17-2.87)	0.629	0.984	0.37 (0.09-1.52)	0.167	0.850
CD86	1.50 (0.71-3.18)	0.289	0.809	2.28 (0.71-7.37)	0.167	0.649	0.84 (0.28-2.51)	0.756	0.985
CD8A	1.19 (0.80-1.77)	0.395	0.892	1.23 (0.60-2.51)	0.569	0.963	0.79 (0.36-1.72)	0.548	0.954

CD93	1.71 (0.89-3.26)	0.105	0.584	4.13 (1.47-11.55)	0.007	0.160	1.59 (0.51-4.94)	0.421	0.942
CD99	2.06 (0.82-5.18)	0.125	0.624	5.55 (1.48-20.86)	0.011	0.199	1.70 (0.32-9.11)	0.538	0.952
CD99L2	1.73 (0.77-3.91)	0.186	0.711	2.83 (0.72-11.15)	0.136	0.600	1.92 (0.46-8.01)	0.371	0.939
CDA	1.14 (0.66-1.95)	0.639	0.968	0.87 (0.31-2.44)	0.792	0.997	1.51 (0.62-3.67)	0.360	0.939
CDAN1	0.62 (0.20-1.96)	0.417	0.896	1.07 (0.39-2.98)	0.892	0.997	0.20 (0.02-1.72)	0.144	0.808
CDC123	0.95 (0.61-1.49)	0.838	0.998	0.45 (0.17-1.18)	0.105	0.530	1.42 (0.75-2.68)	0.282	0.914
CDC25A	0.94 (0.45-1.93)	0.862	0.998	1.64 (0.54-5.00)	0.385	0.888	0.47 (0.11-2.03)	0.315	0.923
CDC26	1.06 (0.74-1.52)	0.746	0.984	1.45 (0.77-2.73)	0.250	0.756	1.30 (0.66-2.56)	0.444	0.942
CDC27	1.09 (0.78-1.53)	0.613	0.966	0.91 (0.47-1.75)	0.773	0.997	0.99 (0.52-1.88)	0.979	0.994
CDC37	0.98 (0.75-1.28)	0.882	0.999	1.08 (0.66-1.77)	0.752	0.997	0.81 (0.49-1.36)	0.428	0.942
CDC42BPB	1.07 (0.85-1.36)	0.567	0.956	1.10 (0.71-1.70)	0.683	0.993	1.05 (0.66-1.65)	0.846	0.992
CDCP1	0.95 (0.65-1.41)	0.813	0.995	1.14 (0.57-2.28)	0.705	0.994	1.32 (0.67-2.60)	0.421	0.942
CDH1	1.51 (0.92-2.47)	0.101	0.576	1.42 (0.61-3.34)	0.419	0.903	2.62 (1.38-4.97)	0.003	0.306
CDH15	0.79 (0.56-1.12)	0.182	0.708	1.24 (0.75-2.06)	0.403	0.902	0.68 (0.37-1.24)	0.208	0.867
CDH17	1.38 (1.01-1.89)	0.042	0.420	1.15 (0.64-2.08)	0.637	0.987	1.59 (0.94-2.67)	0.081	0.743
CDH2	1.36 (0.77-2.41)	0.295	0.814	1.97 (0.74-5.25)	0.177	0.662	0.70 (0.24-2.03)	0.509	0.947
CDH22	0.80 (0.28-2.26)	0.672	0.972	2.57 (0.45-14.83)	0.290	0.801	0.29 (0.04-2.09)	0.219	0.869
CDH23	1.35 (0.55-3.28)	0.515	0.946	2.04 (0.49-8.47)	0.325	0.830	0.68 (0.10-4.51)	0.687	0.975
CDH3	1.46 (0.84-2.55)	0.179	0.706	2.02 (0.81-5.01)	0.130	0.591	1.40 (0.52-3.77)	0.505	0.947
CDH4	0.95 (0.58-1.56)	0.842	0.998	0.65 (0.24-1.79)	0.410	0.903	1.54 (0.73-3.24)	0.255	0.901
CDH5	1.57 (0.77-3.19)	0.216	0.746	2.52 (0.71-8.90)	0.151	0.626	2.03 (0.55-7.53)	0.288	0.915
CDH6	1.15 (0.65-2.06)	0.629	0.966	3.82 (1.37-10.66)	0.011	0.195	0.57 (0.19-1.76)	0.331	0.934
CDHR1	0.90 (0.52-1.54)	0.693	0.975	1.28 (0.57-2.87)	0.548	0.960	1.19 (0.50-2.80)	0.699	0.975
CDHR2	1.03 (0.77-1.39)	0.833	0.997	1.05 (0.62-1.80)	0.849	0.997	1.07 (0.59-1.91)	0.831	0.991
CDHR5	0.74 (0.41-1.34)	0.321	0.838	0.95 (0.31-2.90)	0.930	0.997	0.71 (0.24-2.11)	0.540	0.952

CDK1	1.97 (1.01-3.85)	0.048	0.430	4.25 (1.45-12.41)	0.008	0.176	1.56 (0.49-4.98)	0.450	0.942
CDK5RAP3	0.73 (0.44-1.23)	0.237	0.767	0.64 (0.24-1.68)	0.360	0.862	0.51 (0.18-1.47)	0.214	0.867
CDKL5	1.35 (0.91-2.00)	0.132	0.632	1.44 (0.71-2.91)	0.309	0.816	1.23 (0.59-2.56)	0.588	0.965
CDKN1A	1.06 (0.85-1.31)	0.606	0.963	1.00 (0.68-1.46)	0.981	0.998	1.01 (0.68-1.51)	0.948	0.992
CDKN2D	0.97 (0.76-1.24)	0.796	0.994	1.02 (0.65-1.59)	0.937	0.997	0.92 (0.58-1.45)	0.716	0.976
CDNF	1.18 (0.62-2.25)	0.606	0.963	2.03 (0.72-5.73)	0.182	0.668	1.61 (0.53-4.85)	0.400	0.942
CDON	2.24 (0.96-5.22)	0.062	0.472	10.48 (2.15-51.04)	0.004	0.122	2.12 (0.45-10.05)	0.343	0.936
CDSN	1.76 (1.10-2.82)	0.018	0.308	2.23 (0.97-5.14)	0.061	0.418	1.76 (0.76-4.06)	0.186	0.862
CEACAM1	1.65 (0.64-4.23)	0.296	0.814	3.80 (0.78-18.57)	0.099	0.524	0.60 (0.10-3.44)	0.566	0.960
CEACAM16	1.02 (0.74-1.41)	0.907	0.999	1.24 (0.67-2.27)	0.494	0.940	1.04 (0.58-1.86)	0.893	0.992
CEACAM18	1.06 (0.77-1.44)	0.727	0.978	1.05 (0.57-1.93)	0.872	0.997	0.96 (0.53-1.72)	0.883	0.992
CEACAM19	0.96 (0.54-1.71)	0.882	0.999	1.16 (0.43-3.12)	0.761	0.997	1.75 (0.88-3.49)	0.113	0.768
CEACAM20	0.99 (0.62-1.57)	0.954	0.999	1.64 (0.77-3.46)	0.197	0.702	1.25 (0.58-2.70)	0.577	0.961
CEACAM21	1.45 (1.15-1.82)	0.001	0.085	1.00 (0.65-1.54)	0.994	0.998	2.04 (1.35-3.08)	<0.001	0.174
CEACAM3	0.87 (0.54-1.40)	0.562	0.956	1.17 (0.54-2.52)	0.689	0.994	0.46 (0.19-1.13)	0.089	0.747
CEACAM5	1.31 (0.97-1.77)	0.074	0.512	1.35 (0.77-2.38)	0.295	0.803	1.05 (0.59-1.86)	0.865	0.992
CEACAM6	1.73 (0.98-3.05)	0.059	0.466	2.17 (0.78-6.01)	0.135	0.600	1.72 (0.67-4.42)	0.261	0.910
CEACAM8	1.26 (0.83-1.93)	0.283	0.806	0.92 (0.40-2.11)	0.847	0.997	1.41 (0.69-2.90)	0.350	0.938
CEBPA	1.01 (0.51-1.97)	0.982	0.999	0.67 (0.14-3.19)	0.619	0.980	1.42 (0.54-3.71)	0.478	0.944
CEBPB	0.91 (0.49-1.69)	0.766	0.987	0.24 (0.07-0.83)	0.024	0.271	1.28 (0.45-3.70)	0.644	0.973
CELA2A	0.87 (0.66-1.14)	0.303	0.821	1.10 (0.66-1.84)	0.723	0.995	0.73 (0.46-1.14)	0.167	0.849
CELA3A	0.75 (0.53-1.06)	0.108	0.593	1.18 (0.65-2.16)	0.587	0.967	0.48 (0.27-0.84)	0.010	0.425
CELSR2	1.41 (0.68-2.93)	0.351	0.856	4.03 (1.18-13.77)	0.026	0.283	1.98 (0.53-7.48)	0.312	0.923
CEMIP2	1.07 (0.47-2.44)	0.873	0.998	1.11 (0.24-5.13)	0.898	0.997	0.72 (0.14-3.61)	0.687	0.975
CEND1	0.96 (0.57-1.60)	0.862	0.998	0.65 (0.19-2.16)	0.479	0.932	1.27 (0.68-2.39)	0.451	0.942

CENPF	1.09 (0.73-1.61)	0.678	0.974	0.89 (0.42-1.88)	0.756	0.997	1.34 (0.74-2.44)	0.334	0.934
CENPJ	0.88 (0.56-1.39)	0.593	0.961	1.00 (0.48-2.09)	0.994	0.998	0.76 (0.32-1.80)	0.528	0.949
CEP112	0.91 (0.49-1.68)	0.762	0.987	1.01 (0.38-2.70)	0.984	0.998	0.75 (0.22-2.54)	0.639	0.973
CEP152	1.05 (0.59-1.87)	0.867	0.998	1.67 (0.68-4.11)	0.263	0.773	0.23 (0.05-0.96)	0.045	0.638
CEP164	1.12 (0.92-1.37)	0.247	0.771	0.81 (0.44-1.52)	0.518	0.945	1.18 (0.84-1.64)	0.344	0.936
CEP170	0.97 (0.69-1.36)	0.857	0.998	0.94 (0.50-1.77)	0.845	0.997	0.96 (0.51-1.83)	0.911	0.992
CEP20	1.02 (0.75-1.38)	0.906	0.999	0.96 (0.54-1.71)	0.899	0.997	0.80 (0.45-1.45)	0.469	0.942
CEP290	0.87 (0.54-1.40)	0.568	0.956	0.30 (0.09-0.95)	0.040	0.347	1.24 (0.62-2.45)	0.543	0.952
CEP350	1.60 (0.89-2.90)	0.118	0.609	1.25 (0.37-4.29)	0.721	0.995	0.89 (0.24-3.26)	0.856	0.992
CEP43	0.83 (0.58-1.17)	0.284	0.806	0.89 (0.48-1.65)	0.715	0.995	0.98 (0.51-1.87)	0.943	0.992
CEP85	1.13 (0.81-1.59)	0.459	0.920	0.87 (0.45-1.66)	0.667	0.993	1.57 (0.86-2.86)	0.144	0.808
CERT	1.03 (0.70-1.52)	0.882	0.999	0.98 (0.47-2.02)	0.953	0.997	0.94 (0.45-1.97)	0.870	0.992
CES1	1.23 (0.98-1.55)	0.072	0.502	1.06 (0.68-1.65)	0.796	0.997	1.55 (1.06-2.27)	0.025	0.582
CES2	0.97 (0.66-1.43)	0.885	0.999	0.75 (0.36-1.57)	0.452	0.920	1.41 (0.75-2.66)	0.287	0.915
CES3	0.88 (0.62-1.25)	0.478	0.928	0.90 (0.49-1.67)	0.746	0.997	0.97 (0.52-1.79)	0.918	0.992
CETN2	0.85 (0.60-1.21)	0.360	0.864	0.82 (0.43-1.57)	0.545	0.959	1.05 (0.57-1.97)	0.867	0.992
CETN3	0.77 (0.55-1.09)	0.136	0.641	1.06 (0.59-1.92)	0.847	0.997	0.75 (0.40-1.41)	0.367	0.939
CFB	0.84 (0.39-1.83)	0.667	0.971	0.58 (0.15-2.32)	0.442	0.914	0.62 (0.15-2.60)	0.509	0.947
CFC1	0.90 (0.55-1.49)	0.688	0.975	1.42 (0.63-3.19)	0.396	0.897	0.81 (0.31-2.11)	0.663	0.975
CFD	2.51 (1.25-5.04)	0.009	0.230	3.56 (1.42-8.94)	0.007	0.160	2.32 (0.78-6.93)	0.130	0.796
CFH	1.39 (0.48-4.02)	0.549	0.949	2.85 (0.53-15.23)	0.220	0.729	0.30 (0.04-2.45)	0.263	0.910
CFHR2	1.55 (0.92-2.60)	0.100	0.573	2.18 (0.77-6.21)	0.143	0.607	1.77 (0.65-4.87)	0.266	0.910
CFHR4	1.32 (0.92-1.89)	0.134	0.639	1.32 (0.68-2.59)	0.413	0.903	1.25 (0.64-2.46)	0.515	0.947
CFHR5	1.83 (0.99-3.37)	0.052	0.443	1.61 (0.53-4.91)	0.401	0.901	1.69 (0.56-5.13)	0.353	0.938
CFI	2.65 (0.80-8.79)	0.112	0.600	7.31 (0.80-66.69)	0.078	0.471	1.70 (0.18-16.15)	0.642	0.973

CFP	0.52 (0.17-1.62)	0.262	0.789	1.52 (0.19-12.33)	0.694	0.994	0.17 (0.02-1.29)	0.087	0.745
CGA	1.29 (0.94-1.78)	0.116	0.608	1.43 (0.80-2.56)	0.233	0.742	1.79 (0.98-3.25)	0.056	0.698
CGB3_CGB5_CGB8	1.18 (0.62-2.28)	0.614	0.966	0.62 (0.19-1.97)	0.415	0.903	1.84 (0.53-6.40)	0.340	0.935
CGN	1.65 (1.05-2.60)	0.030	0.372	1.81 (0.82-4.00)	0.143	0.607	0.87 (0.25-3.05)	0.826	0.991
CGREF1	1.83 (1.18-2.83)	0.007	0.197	1.41 (0.60-3.33)	0.431	0.908	2.25 (1.09-4.63)	0.028	0.588
CHAC2	0.99 (0.73-1.36)	0.973	0.999	0.93 (0.51-1.70)	0.810	0.997	1.08 (0.63-1.87)	0.778	0.987
CHAD	1.31 (0.82-2.10)	0.258	0.786	3.63 (1.39-9.49)	0.009	0.182	1.20 (0.53-2.74)	0.663	0.975
CHCHD10	1.68 (1.12-2.52)	0.012	0.260	1.97 (1.07-3.62)	0.029	0.295	2.19 (1.25-3.82)	0.006	0.356
CHCHD6	1.62 (0.88-2.97)	0.120	0.617	2.79 (1.48-5.26)	0.002	0.078	1.59 (0.54-4.72)	0.400	0.942
CHEK2	0.88 (0.43-1.78)	0.719	0.975	0.97 (0.27-3.55)	0.963	0.997	1.00 (0.28-3.59)	0.994	0.998
CHGA	1.15 (0.94-1.42)	0.184	0.708	1.25 (0.87-1.79)	0.234	0.742	1.23 (0.85-1.78)	0.276	0.910
CHGB	0.99 (0.61-1.62)	0.973	0.999	0.88 (0.36-2.16)	0.782	0.997	1.11 (0.45-2.70)	0.824	0.991
CHI3L1	1.22 (0.95-1.57)	0.118	0.609	1.53 (0.98-2.37)	0.059	0.407	1.19 (0.77-1.84)	0.433	0.942
CHIT1	0.93 (0.83-1.05)	0.237	0.767	1.10 (0.82-1.47)	0.531	0.949	0.82 (0.69-0.98)	0.031	0.603
CHL1	1.14 (0.56-2.31)	0.711	0.975	2.09 (0.63-6.95)	0.229	0.739	0.49 (0.11-2.27)	0.363	0.939
CHM	0.92 (0.59-1.43)	0.707	0.975	0.78 (0.33-1.81)	0.556	0.962	0.70 (0.28-1.73)	0.436	0.942
CHMP1A	0.92 (0.68-1.25)	0.592	0.961	1.00 (0.57-1.76)	0.990	0.998	0.75 (0.43-1.33)	0.328	0.932
CHMP6	0.88 (0.55-1.40)	0.586	0.960	1.52 (0.68-3.43)	0.311	0.816	0.55 (0.22-1.37)	0.199	0.867
CHP1	1.07 (0.73-1.59)	0.720	0.975	1.24 (0.65-2.37)	0.508	0.942	0.98 (0.44-2.21)	0.964	0.994
CHRDL1	3.12 (1.70-5.75)	<0.001	0.046	6.69 (2.48-18.02)	<0.001	0.033	2.65 (0.86-8.15)	0.089	0.745
CHRDL2	0.96 (0.64-1.45)	0.849	0.998	1.82 (0.91-3.64)	0.089	0.495	0.94 (0.42-2.11)	0.884	0.992
CHRM1	1.87 (1.25-2.81)	0.002	0.115	1.88 (0.83-4.25)	0.131	0.595	1.75 (0.75-4.05)	0.193	0.865
CIAPIN1	1.04 (0.72-1.51)	0.824	0.995	1.26 (0.64-2.47)	0.509	0.942	0.76 (0.38-1.52)	0.432	0.942
CILP	1.11 (0.77-1.61)	0.577	0.956	2.07 (1.10-3.90)	0.024	0.271	1.25 (0.65-2.41)	0.502	0.947
CINP	1.13 (0.61-2.09)	0.696	0.975	1.44 (0.51-4.00)	0.490	0.939	0.85 (0.23-3.06)	0.799	0.991

CIRBP	0.90 (0.69-1.16)	0.399	0.894	1.10 (0.69-1.76)	0.683	0.993	0.63 (0.38-1.02)	0.062	0.698
CIT	0.87 (0.52-1.45)	0.594	0.962	1.12 (0.50-2.51)	0.786	0.997	1.01 (0.45-2.26)	0.975	0.994
CKAP4	1.99 (1.07-3.70)	0.030	0.372	2.16 (0.74-6.33)	0.162	0.639	2.64 (0.96-7.27)	0.060	0.698
CKB	1.55 (1.04-2.33)	0.033	0.386	1.62 (0.80-3.29)	0.182	0.668	0.99 (0.46-2.14)	0.979	0.994
CKMT1A_CKMT1B	0.93 (0.69-1.25)	0.629	0.966	1.34 (0.70-2.58)	0.374	0.879	1.49 (0.79-2.81)	0.219	0.869
CLASP1	1.14 (0.80-1.62)	0.460	0.920	1.59 (0.98-2.56)	0.058	0.407	1.15 (0.58-2.26)	0.694	0.975
CLC	0.95 (0.67-1.34)	0.762	0.987	1.62 (1.01-2.62)	0.047	0.375	0.46 (0.22-0.96)	0.038	0.623
CLEC10A	1.02 (0.56-1.87)	0.940	0.999	1.63 (0.56-4.77)	0.370	0.874	1.07 (0.37-3.13)	0.895	0.992
CLEC11A	1.48 (0.94-2.32)	0.090	0.548	2.19 (0.96-4.98)	0.062	0.425	1.37 (0.61-3.06)	0.442	0.942
CLEC12A	2.03 (0.57-7.16)	0.272	0.799	12.85 (2.96-55.74)	<0.001	0.059	0.82 (0.06-10.94)	0.881	0.992
CLEC14A	2.43 (1.37-4.31)	0.002	0.115	3.86 (1.53-9.77)	0.004	0.137	2.47 (0.93-6.57)	0.071	0.718
CLEC1A	1.41 (0.76-2.60)	0.271	0.799	2.95 (1.10-7.92)	0.032	0.314	0.64 (0.22-1.92)	0.430	0.942
CLEC1B	0.99 (0.78-1.27)	0.957	0.999	1.06 (0.67-1.67)	0.806	0.997	0.91 (0.56-1.47)	0.690	0.975
CLEC2L	1.04 (0.76-1.43)	0.783	0.994	0.95 (0.50-1.81)	0.873	0.997	0.96 (0.51-1.81)	0.892	0.992
CLEC3B	0.55 (0.16-1.92)	0.349	0.856	1.53 (0.71-3.30)	0.277	0.788	0.24 (0.03-2.19)	0.206	0.867
CLEC4A	0.92 (0.47-1.76)	0.791	0.994	1.07 (0.32-3.55)	0.915	0.997	0.88 (0.26-2.92)	0.831	0.991
CLEC4C	0.83 (0.53-1.31)	0.422	0.899	1.79 (0.82-3.93)	0.146	0.617	0.76 (0.34-1.73)	0.520	0.948
CLEC4D	1.55 (1.11-2.16)	0.010	0.232	1.23 (0.67-2.24)	0.503	0.941	1.99 (1.11-3.58)	0.021	0.550
CLEC4G	0.74 (0.37-1.45)	0.376	0.875	0.56 (0.16-1.94)	0.360	0.862	0.48 (0.13-1.80)	0.277	0.910
CLEC4M	1.22 (0.73-2.05)	0.442	0.911	0.86 (0.33-2.20)	0.750	0.997	1.65 (0.63-4.30)	0.305	0.923
CLEC5A	2.28 (1.16-4.47)	0.017	0.294	4.28 (1.34-13.68)	0.014	0.220	1.25 (0.33-4.67)	0.745	0.984
CLEC6A	1.38 (0.89-2.12)	0.147	0.671	1.44 (0.65-3.17)	0.364	0.866	1.24 (0.56-2.71)	0.598	0.966
CLEC7A	1.95 (1.24-3.07)	0.004	0.139	2.44 (1.08-5.51)	0.032	0.314	1.23 (0.55-2.73)	0.611	0.966
CLGN	1.42 (0.99-2.05)	0.056	0.460	1.20 (0.53-2.71)	0.669	0.993	1.68 (0.99-2.85)	0.055	0.698
CLIC5	1.62 (0.82-3.19)	0.166	0.692	1.17 (0.29-4.65)	0.827	0.997	4.78 (2.08-10.99)	<0.001	0.157

CLINT1	1.08 (0.68-1.71)	0.756	0.986	0.82 (0.33-2.04)	0.663	0.992	0.92 (0.35-2.38)	0.858	0.992
CLIP2	1.05 (0.86-1.27)	0.650	0.971	0.95 (0.67-1.36)	0.789	0.997	1.01 (0.70-1.46)	0.965	0.994
CLMP	1.75 (0.71-4.36)	0.226	0.757	2.15 (0.44-10.47)	0.342	0.848	2.44 (0.53-11.25)	0.254	0.901
CLNS1A	0.74 (0.36-1.52)	0.413	0.894	0.59 (0.15-2.24)	0.435	0.909	0.83 (0.25-2.72)	0.758	0.986
CLPP	1.02 (0.80-1.31)	0.845	0.998	0.88 (0.57-1.35)	0.549	0.960	0.84 (0.54-1.31)	0.448	0.942
CLPS	0.87 (0.64-1.17)	0.354	0.858	1.13 (0.66-1.91)	0.659	0.992	0.69 (0.39-1.22)	0.201	0.867
CLSPN	1.03 (0.55-1.94)	0.927	0.999	0.98 (0.33-2.91)	0.965	0.997	1.23 (0.56-2.73)	0.604	0.966
CLSTN1	0.77 (0.46-1.30)	0.332	0.847	0.56 (0.21-1.48)	0.242	0.744	1.98 (1.12-3.49)	0.019	0.519
CLSTN2	1.49 (0.88-2.52)	0.136	0.640	2.24 (0.88-5.66)	0.089	0.495	1.36 (0.51-3.62)	0.539	0.952
CLSTN3	2.13 (1.02-4.45)	0.045	0.426	5.78 (1.82-18.42)	0.003	0.110	1.99 (0.56-7.10)	0.288	0.915
CLTA	0.76 (0.48-1.19)	0.230	0.763	0.64 (0.27-1.54)	0.319	0.823	0.60 (0.23-1.55)	0.292	0.915
CLU	0.80 (0.22-2.95)	0.742	0.984	0.68 (0.06-8.14)	0.764	0.997	0.59 (0.03-10.42)	0.719	0.976
CLUL1	0.81 (0.50-1.33)	0.410	0.894	0.81 (0.33-2.00)	0.654	0.992	0.98 (0.39-2.42)	0.959	0.994
CMC1	0.90 (0.68-1.19)	0.464	0.922	1.09 (0.66-1.82)	0.738	0.997	0.66 (0.37-1.17)	0.151	0.816
CMIP	1.01 (0.75-1.34)	0.966	0.999	1.18 (0.70-1.98)	0.540	0.955	0.73 (0.42-1.28)	0.270	0.910
CNDP1	0.73 (0.45-1.17)	0.191	0.718	0.70 (0.30-1.65)	0.418	0.903	1.13 (0.46-2.77)	0.782	0.987
CNGB3	1.56 (1.18-2.07)	0.002	0.107	2.33 (1.60-3.38)	<0.001	0.007	1.22 (0.60-2.47)	0.585	0.965
CNP	0.93 (0.68-1.27)	0.663	0.971	1.00 (0.57-1.77)	0.995	0.998	0.83 (0.46-1.50)	0.539	0.952
CNPY2	1.10 (0.78-1.56)	0.586	0.960	1.13 (0.61-2.11)	0.702	0.994	0.45 (0.16-1.29)	0.139	0.807
CNPY4	0.87 (0.64-1.19)	0.381	0.875	0.73 (0.41-1.27)	0.264	0.773	0.91 (0.50-1.64)	0.752	0.985
CNST	1.07 (0.87-1.31)	0.519	0.947	0.95 (0.65-1.38)	0.780	0.997	0.98 (0.67-1.44)	0.919	0.992
CNTF	0.85 (0.39-1.84)	0.678	0.974	1.43 (0.42-4.89)	0.567	0.963	0.55 (0.14-2.09)	0.379	0.942
CNTN1	2.83 (1.29-6.20)	0.009	0.230	4.02 (0.93-17.27)	0.062	0.422	2.12 (0.48-9.29)	0.318	0.923
CNTN2	2.04 (1.27-3.29)	0.003	0.133	2.88 (1.24-6.69)	0.014	0.220	2.30 (0.94-5.63)	0.067	0.718
CNTN3	1.23 (0.63-2.39)	0.551	0.949	0.95 (0.28-3.16)	0.928	0.997	0.79 (0.23-2.72)	0.712	0.976

CNTN4	1.95 (0.81-4.67)	0.135	0.640	4.39 (0.93-20.74)	0.062	0.422	3.06 (0.60-15.73)	0.180	0.853
CNTN5	1.56 (0.91-2.70)	0.108	0.593	1.53 (0.55-4.30)	0.415	0.903	1.45 (0.54-3.93)	0.460	0.942
CNTNAP2	1.86 (1.16-2.98)	0.010	0.232	2.68 (1.17-6.15)	0.020	0.252	1.16 (0.46-2.88)	0.755	0.985
CNTNAP4	1.14 (0.89-1.46)	0.286	0.806	1.27 (0.85-1.89)	0.240	0.743	1.07 (0.64-1.80)	0.790	0.989
COCH	1.29 (0.78-2.12)	0.326	0.843	1.82 (0.74-4.50)	0.192	0.691	0.70 (0.26-1.91)	0.489	0.946
COL15A1	1.27 (0.59-2.72)	0.542	0.948	6.52 (2.21-19.27)	<0.001	0.061	0.68 (0.17-2.84)	0.602	0.966
COL18A1	3.55 (1.60-7.88)	0.002	0.107	3.54 (0.86-14.65)	0.081	0.483	5.07 (1.26-20.47)	0.023	0.551
COL1A1	1.47 (0.66-3.27)	0.348	0.855	4.97 (1.92-12.90)	<0.001	0.063	0.93 (0.21-4.20)	0.926	0.992
COL24A1	1.65 (1.06-2.59)	0.027	0.362	1.60 (0.71-3.62)	0.260	0.772	3.71 (1.41-9.79)	0.008	0.393
COL28A1	0.97 (0.72-1.32)	0.863	0.998	0.45 (0.24-0.86)	0.015	0.227	0.89 (0.51-1.56)	0.683	0.975
COL2A1	0.80 (0.57-1.12)	0.195	0.720	0.63 (0.32-1.22)	0.172	0.657	0.79 (0.43-1.47)	0.461	0.942
COL3A1	2.43 (0.99-6.00)	0.054	0.448	4.23 (1.34-13.39)	0.014	0.220	2.45 (0.43-14.00)	0.312	0.923
COL4A1	3.17 (1.91-5.26)	<0.001	0.006	4.55 (1.86-11.11)	<0.001	0.063	2.63 (1.01-6.83)	0.047	0.659
COL4A4	1.03 (0.72-1.47)	0.880	0.999	0.76 (0.47-1.26)	0.289	0.801	1.00 (0.53-1.91)	0.995	0.998
COL5A1	1.29 (0.77-2.15)	0.331	0.847	2.11 (1.09-4.07)	0.026	0.281	1.67 (0.73-3.81)	0.223	0.875
COL6A3	2.42 (1.55-3.79)	<0.001	0.024	3.95 (1.90-8.23)	<0.001	0.042	2.25 (1.03-4.90)	0.042	0.630
COL9A1	0.85 (0.55-1.32)	0.472	0.924	1.19 (0.57-2.50)	0.645	0.991	0.71 (0.30-1.64)	0.420	0.942
COL9A2	0.90 (0.50-1.60)	0.710	0.975	0.74 (0.23-2.35)	0.611	0.979	0.97 (0.41-2.34)	0.954	0.992
COLEC12	2.14 (1.10-4.16)	0.025	0.352	2.46 (0.77-7.85)	0.130	0.590	3.96 (1.44-10.85)	0.007	0.370
COMMD1	0.77 (0.49-1.21)	0.249	0.774	1.14 (0.52-2.52)	0.744	0.997	0.70 (0.30-1.68)	0.430	0.942
COMMD9	1.68 (0.99-2.88)	0.057	0.461	2.05 (0.85-4.96)	0.110	0.546	1.79 (0.71-4.53)	0.218	0.869
COMP	0.69 (0.36-1.30)	0.251	0.777	2.68 (0.90-7.93)	0.076	0.467	1.26 (0.42-3.80)	0.680	0.975
COMT	0.99 (0.74-1.31)	0.922	0.999	1.08 (0.64-1.81)	0.773	0.997	0.91 (0.54-1.56)	0.742	0.983
COPB2	1.05 (0.59-1.86)	0.881	0.999	1.09 (0.40-2.99)	0.870	0.997	0.83 (0.27-2.60)	0.753	0.985
COPE	1.30 (0.84-2.01)	0.239	0.768	1.11 (0.47-2.61)	0.805	0.997	1.08 (0.43-2.69)	0.873	0.992

COQ7	1.11 (0.62-1.99)	0.725	0.977	0.71 (0.21-2.44)	0.587	0.967	2.81 (1.28-6.19)	0.010	0.425
CORO1A	1.03 (0.80-1.31)	0.830	0.997	1.00 (0.63-1.59)	0.988	0.998	1.03 (0.65-1.64)	0.888	0.992
CORO6	1.17 (0.69-1.97)	0.554	0.950	1.01 (0.36-2.80)	0.991	0.998	1.56 (0.77-3.15)	0.216	0.869
COX5B	0.95 (0.69-1.31)	0.761	0.987	0.76 (0.42-1.40)	0.383	0.887	1.14 (0.64-2.06)	0.654	0.975
COX6B1	1.19 (0.55-2.57)	0.654	0.971	1.59 (0.42-5.99)	0.492	0.940	0.76 (0.16-3.56)	0.730	0.978
CPA1	0.93 (0.66-1.32)	0.696	0.975	1.34 (0.74-2.45)	0.334	0.839	0.94 (0.51-1.73)	0.849	0.992
CPA2	0.83 (0.60-1.15)	0.256	0.785	1.18 (0.67-2.08)	0.558	0.962	0.87 (0.49-1.57)	0.649	0.973
CPA4	1.35 (0.76-2.41)	0.309	0.826	9.83 (3.21-30.14)	<0.001	0.021	0.63 (0.23-1.68)	0.355	0.939
CPB1	0.86 (0.59-1.25)	0.430	0.904	1.24 (0.67-2.30)	0.486	0.934	1.19 (0.64-2.24)	0.582	0.964
CPB2	0.76 (0.25-2.33)	0.632	0.968	1.42 (0.21-9.61)	0.719	0.995	0.60 (0.08-4.27)	0.607	0.966
CPE	1.22 (0.67-2.23)	0.520	0.947	1.57 (0.48-5.12)	0.458	0.923	1.30 (0.42-4.02)	0.644	0.973
CPLX2	1.64 (0.88-3.07)	0.120	0.617	1.37 (0.36-5.21)	0.640	0.990	1.89 (0.70-5.14)	0.210	0.867
CPM	1.92 (1.04-3.52)	0.036	0.401	1.55 (0.50-4.81)	0.445	0.916	3.72 (1.25-11.10)	0.018	0.512
CPOX	1.14 (0.63-2.08)	0.658	0.971	0.90 (0.28-2.87)	0.861	0.997	1.45 (0.51-4.08)	0.486	0.944
CPPED1	1.32 (0.96-1.81)	0.089	0.546	1.01 (0.56-1.81)	0.974	0.997	1.47 (0.84-2.60)	0.181	0.853
CPQ	1.76 (0.97-3.18)	0.061	0.468	2.73 (0.99-7.51)	0.051	0.391	1.13 (0.37-3.42)	0.828	0.991
CPTP	0.96 (0.45-2.05)	0.909	0.999	1.68 (0.50-5.72)	0.403	0.902	0.39 (0.07-2.04)	0.264	0.910
CPVL	1.17 (0.68-2.02)	0.572	0.956	1.08 (0.39-2.93)	0.887	0.997	2.53 (0.95-6.73)	0.064	0.709
CPXM1	1.03 (0.74-1.43)	0.859	0.998	1.01 (0.55-1.87)	0.964	0.997	0.69 (0.36-1.32)	0.265	0.910
CPXM2	3.21 (1.40-7.36)	0.006	0.184	9.03 (2.39-34.09)	0.001	0.066	5.32 (1.16-24.36)	0.031	0.607
CR1	1.69 (0.87-3.29)	0.122	0.620	3.69 (1.54-8.86)	0.003	0.120	2.04 (0.59-7.12)	0.263	0.910
CR2	1.02 (0.65-1.61)	0.928	0.999	2.34 (1.12-4.89)	0.024	0.271	0.50 (0.20-1.22)	0.126	0.795
CRACR2A	0.99 (0.76-1.29)	0.947	0.999	0.87 (0.52-1.43)	0.572	0.964	1.00 (0.61-1.65)	0.994	0.998
CRADD	1.04 (0.77-1.41)	0.806	0.995	1.06 (0.61-1.84)	0.842	0.997	0.94 (0.53-1.65)	0.822	0.991
CREB3	1.24 (0.78-1.97)	0.370	0.871	1.41 (0.64-3.08)	0.392	0.894	1.52 (0.72-3.20)	0.275	0.910

CREBZF	1.81 (0.69-4.70)	0.226	0.757	1.82 (0.33-10.10)	0.492	0.940	0.19 (0.03-1.10)	0.064	0.709
CREG1	0.84 (0.49-1.46)	0.540	0.948	1.76 (0.80-3.91)	0.162	0.639	0.86 (0.32-2.27)	0.753	0.985
CRELD1	2.12 (1.10-4.10)	0.025	0.352	5.15 (1.61-16.51)	0.006	0.146	3.39 (1.06-10.86)	0.040	0.629
CRELD2	1.14 (0.75-1.75)	0.544	0.948	0.95 (0.43-2.12)	0.910	0.997	1.22 (0.55-2.70)	0.626	0.969
CRH	1.07 (0.83-1.38)	0.622	0.966	0.90 (0.55-1.46)	0.663	0.992	1.21 (0.75-1.93)	0.435	0.942
CRHBP	1.31 (0.59-2.93)	0.504	0.943	1.74 (0.40-7.51)	0.460	0.924	2.68 (0.61-11.77)	0.193	0.865
CRHR1	0.95 (0.67-1.36)	0.793	0.994	0.78 (0.37-1.62)	0.503	0.941	1.12 (0.61-2.07)	0.718	0.976
CRIM1	2.82 (1.04-7.65)	0.041	0.418	3.70 (0.59-23.15)	0.161	0.639	4.56 (0.77-26.94)	0.094	0.762
CRIP2	1.38 (0.75-2.55)	0.307	0.823	1.95 (0.65-5.86)	0.233	0.742	1.54 (0.50-4.81)	0.454	0.942
CRISP2	0.82 (0.57-1.18)	0.278	0.804	0.85 (0.43-1.68)	0.631	0.984	0.54 (0.29-1.04)	0.064	0.710
CRISP3	0.92 (0.35-2.44)	0.863	0.998	1.84 (0.30-11.11)	0.507	0.942	1.26 (0.22-7.08)	0.792	0.989
CRKL	1.01 (0.82-1.26)	0.903	0.999	1.06 (0.71-1.59)	0.764	0.997	0.91 (0.61-1.37)	0.664	0.975
CRLF1	1.00 (0.42-2.41)	0.999	1.000	8.21 (1.53-44.05)	0.014	0.220	0.89 (0.18-4.49)	0.888	0.992
CRNN	0.88 (0.63-1.21)	0.420	0.899	0.64 (0.36-1.14)	0.129	0.590	0.69 (0.39-1.23)	0.211	0.867
CRTAC1	0.74 (0.36-1.50)	0.405	0.894	1.28 (0.35-4.69)	0.707	0.994	0.91 (0.23-3.56)	0.897	0.992
CRTAM	0.91 (0.58-1.41)	0.668	0.971	1.26 (0.57-2.78)	0.567	0.963	0.52 (0.23-1.21)	0.130	0.796
CRTAP	1.11 (0.65-1.88)	0.706	0.975	1.15 (0.44-2.99)	0.771	0.997	0.47 (0.13-1.69)	0.247	0.900
CRX	1.05 (0.86-1.28)	0.635	0.968	1.32 (1.02-1.71)	0.033	0.317	0.82 (0.48-1.37)	0.442	0.942
CRYBB1	0.85 (0.58-1.25)	0.406	0.894	1.40 (0.72-2.76)	0.324	0.830	0.50 (0.23-1.09)	0.081	0.743
CRYBB2	0.85 (0.59-1.24)	0.400	0.894	0.72 (0.31-1.66)	0.439	0.912	1.16 (0.75-1.78)	0.507	0.947
CRYGD	0.81 (0.64-1.04)	0.100	0.573	0.86 (0.55-1.34)	0.502	0.941	0.64 (0.40-1.02)	0.062	0.698
CRYM	0.88 (0.59-1.30)	0.512	0.945	0.67 (0.31-1.46)	0.314	0.818	1.27 (0.69-2.36)	0.445	0.942
CRYZL1	0.86 (0.65-1.14)	0.295	0.813	0.97 (0.58-1.61)	0.897	0.997	0.88 (0.52-1.50)	0.644	0.973
CSDE1	1.08 (0.87-1.34)	0.497	0.939	1.21 (0.86-1.72)	0.275	0.788	0.80 (0.45-1.41)	0.442	0.942
CSF1	2.05 (1.03-4.07)	0.041	0.416	2.58 (0.77-8.66)	0.126	0.587	2.67 (0.74-9.65)	0.133	0.796

CSF1R	1.67 (1.00-2.79)	0.051	0.442	2.09 (0.93-4.70)	0.075	0.465	1.18 (0.40-3.51)	0.763	0.987
CSF2	1.59 (0.94-2.67)	0.081	0.522	1.64 (0.69-3.87)	0.262	0.773	1.49 (0.62-3.57)	0.373	0.940
CSF2RA	1.41 (0.98-2.04)	0.064	0.473	1.86 (0.97-3.60)	0.064	0.428	1.12 (0.56-2.24)	0.756	0.985
CSF2RB	1.55 (0.84-2.87)	0.159	0.680	1.62 (0.53-4.98)	0.398	0.899	1.12 (0.37-3.37)	0.845	0.992
CSF3	1.12 (0.71-1.78)	0.625	0.966	1.39 (0.64-3.04)	0.404	0.902	0.72 (0.29-1.79)	0.485	0.944
CSF3R	1.85 (0.97-3.51)	0.060	0.466	1.50 (0.47-4.77)	0.496	0.940	1.48 (0.48-4.63)	0.497	0.947
CSH1	0.98 (0.41-2.34)	0.962	0.999	1.08 (0.21-5.40)	0.929	0.997	0.95 (0.20-4.45)	0.946	0.992
CSNK1D	0.85 (0.56-1.30)	0.462	0.920	0.97 (0.47-2.03)	0.942	0.997	0.79 (0.34-1.80)	0.570	0.960
CSNK2A1	0.88 (0.48-1.61)	0.685	0.975	1.23 (0.43-3.47)	0.701	0.994	0.55 (0.17-1.76)	0.318	0.923
CSPG4	2.34 (1.13-4.86)	0.022	0.344	5.18 (1.40-19.11)	0.014	0.220	3.49 (0.97-12.50)	0.055	0.698
CSPG5	1.51 (0.95-2.38)	0.078	0.514	3.03 (1.76-5.22)	<0.001	0.021	1.63 (0.74-3.61)	0.224	0.877
CSRP3	1.08 (0.86-1.35)	0.497	0.939	1.14 (0.77-1.70)	0.511	0.943	0.96 (0.62-1.47)	0.838	0.991
CST1	1.31 (1.07-1.60)	0.008	0.204	1.26 (0.87-1.82)	0.215	0.723	1.76 (1.27-2.46)	<0.001	0.179
CST3	1.69 (0.91-3.16)	0.099	0.573	2.89 (1.00-8.41)	0.051	0.391	1.38 (0.44-4.34)	0.582	0.964
CST5	0.78 (0.52-1.18)	0.242	0.769	0.86 (0.41-1.81)	0.686	0.993	1.11 (0.54-2.31)	0.776	0.987
CST6	1.14 (0.75-1.74)	0.533	0.948	1.85 (0.89-3.81)	0.097	0.520	0.70 (0.32-1.52)	0.365	0.939
CST7	0.89 (0.69-1.15)	0.378	0.875	0.81 (0.48-1.36)	0.417	0.903	0.98 (0.63-1.51)	0.912	0.992
CSTB	1.53 (0.93-2.53)	0.097	0.569	1.15 (0.46-2.89)	0.765	0.997	1.51 (0.62-3.65)	0.365	0.939
CTAG1A_CTAG1B	1.09 (0.71-1.69)	0.686	0.975	0.67 (0.41-1.09)	0.110	0.546	0.72 (0.32-1.60)	0.416	0.942
CTBS	1.07 (0.43-2.65)	0.891	0.999	5.00 (1.01-24.73)	0.049	0.383	0.28 (0.07-1.11)	0.070	0.718
CTF1	1.14 (0.87-1.50)	0.344	0.855	1.54 (0.95-2.48)	0.080	0.481	0.91 (0.53-1.55)	0.718	0.976
CTHRC1	2.28 (1.20-4.37)	0.012	0.268	10.00 (4.02-24.88)	<0.001	0.002	1.60 (0.51-4.97)	0.420	0.942
CTLA4	1.38 (0.56-3.43)	0.485	0.933	3.94 (1.00-15.59)	0.050	0.391	0.28 (0.05-1.50)	0.137	0.799
CTNNA1	0.75 (0.31-1.84)	0.530	0.948	0.91 (0.20-4.11)	0.904	0.997	0.30 (0.04-2.20)	0.236	0.885
CTRB1	0.89 (0.63-1.27)	0.527	0.948	1.42 (0.81-2.48)	0.225	0.736	0.84 (0.45-1.54)	0.564	0.958

CTRC	0.80 (0.59-1.10)	0.170	0.697	1.22 (0.68-2.17)	0.503	0.941	0.65 (0.38-1.10)	0.106	0.763
CTRL	0.73 (0.54-0.99)	0.042	0.420	1.20 (0.68-2.13)	0.526	0.948	0.47 (0.28-0.79)	0.005	0.336
CTSB	1.12 (0.71-1.76)	0.637	0.968	1.53 (0.67-3.49)	0.308	0.816	0.91 (0.39-2.12)	0.834	0.991
CTSC	1.16 (0.71-1.91)	0.552	0.950	1.81 (0.84-3.90)	0.132	0.596	1.18 (0.46-3.02)	0.722	0.977
CTSD	1.11 (0.70-1.78)	0.656	0.971	1.44 (0.70-2.97)	0.319	0.823	1.50 (0.73-3.09)	0.271	0.910
CTSE	1.04 (0.64-1.68)	0.870	0.998	1.22 (0.51-2.88)	0.654	0.992	0.83 (0.34-1.98)	0.667	0.975
CTSF	1.17 (0.70-1.96)	0.541	0.948	1.14 (0.45-2.89)	0.788	0.997	0.63 (0.23-1.73)	0.371	0.939
CTSH	1.29 (0.95-1.76)	0.104	0.580	0.93 (0.52-1.65)	0.794	0.997	1.30 (0.73-2.30)	0.370	0.939
CTSL	1.50 (0.62-3.64)	0.373	0.874	2.54 (0.55-11.75)	0.233	0.742	1.90 (0.47-7.62)	0.365	0.939
CTSO	1.33 (0.70-2.51)	0.379	0.875	1.19 (0.41-3.49)	0.747	0.997	1.58 (0.50-4.95)	0.435	0.942
CTSS	0.90 (0.29-2.80)	0.862	0.998	1.55 (0.21-11.41)	0.666	0.993	2.13 (0.28-15.88)	0.462	0.942
CTSV	1.14 (0.70-1.86)	0.589	0.961	0.70 (0.29-1.68)	0.423	0.905	1.43 (0.64-3.20)	0.389	0.942
CTSZ	1.12 (0.60-2.09)	0.731	0.979	2.01 (0.66-6.06)	0.216	0.725	1.12 (0.35-3.56)	0.850	0.992
CUZD1	0.73 (0.34-1.57)	0.418	0.898	0.67 (0.16-2.81)	0.580	0.967	0.66 (0.16-2.77)	0.566	0.959
CWC15	1.12 (0.77-1.64)	0.546	0.949	1.42 (0.81-2.49)	0.220	0.729	1.04 (0.48-2.26)	0.914	0.992
CX3CL1	1.82 (1.01-3.28)	0.045	0.426	2.90 (1.05-8.02)	0.041	0.349	1.89 (0.69-5.20)	0.215	0.869
CXADR	1.25 (0.84-1.86)	0.273	0.799	1.15 (0.54-2.44)	0.722	0.995	0.94 (0.40-2.18)	0.878	0.992
CXCL1	1.00 (0.79-1.26)	0.980	0.999	1.02 (0.67-1.55)	0.938	0.997	0.75 (0.45-1.23)	0.255	0.901
CXCL10	1.20 (0.89-1.60)	0.231	0.763	1.80 (1.14-2.84)	0.011	0.199	1.08 (0.62-1.87)	0.792	0.989
CXCL11	1.14 (0.88-1.47)	0.313	0.830	1.48 (0.99-2.23)	0.058	0.406	0.87 (0.52-1.48)	0.615	0.966
CXCL12	1.05 (0.63-1.74)	0.862	0.998	1.28 (0.52-3.18)	0.589	0.969	0.73 (0.26-2.02)	0.544	0.952
CXCL13	1.40 (1.01-1.96)	0.046	0.428	2.04 (1.23-3.39)	0.006	0.146	0.75 (0.35-1.62)	0.469	0.942
CXCL14	0.89 (0.60-1.32)	0.567	0.956	1.06 (0.57-1.99)	0.847	0.997	1.24 (0.66-2.34)	0.504	0.947
CXCL16	2.14 (0.89-5.16)	0.089	0.546	1.52 (0.30-7.72)	0.613	0.979	1.97 (0.38-10.16)	0.416	0.942
CXCL17	1.10 (0.66-1.83)	0.721	0.975	1.11 (0.44-2.85)	0.821	0.997	1.01 (0.42-2.46)	0.975	0.994

CXCL3	0.92 (0.72-1.16)	0.462	0.920	0.93 (0.60-1.44)	0.748	0.997	0.94 (0.60-1.46)	0.769	0.987
CXCL5	0.95 (0.78-1.16)	0.628	0.966	0.91 (0.64-1.29)	0.586	0.967	0.98 (0.68-1.42)	0.930	0.992
CXCL6	1.04 (0.78-1.39)	0.808	0.995	1.06 (0.63-1.80)	0.817	0.997	0.99 (0.57-1.71)	0.969	0.994
CXCL8	1.07 (0.77-1.48)	0.694	0.975	0.81 (0.44-1.51)	0.511	0.943	1.02 (0.56-1.88)	0.942	0.992
CXCL9	1.06 (0.77-1.47)	0.710	0.975	1.49 (0.89-2.48)	0.128	0.590	0.75 (0.39-1.45)	0.397	0.942
CYB5A	0.90 (0.62-1.31)	0.579	0.956	0.69 (0.32-1.49)	0.347	0.854	0.92 (0.46-1.84)	0.808	0.991
CYB5R2	0.63 (0.44-0.90)	0.011	0.248	1.00 (0.46-2.20)	0.996	0.998	0.57 (0.34-0.95)	0.032	0.610
CYP24A1	0.79 (0.53-1.16)	0.224	0.757	1.17 (0.70-1.98)	0.550	0.960	0.64 (0.28-1.43)	0.275	0.910
CYTH3	0.93 (0.64-1.34)	0.686	0.975	1.27 (0.81-1.97)	0.295	0.803	0.41 (0.18-0.95)	0.038	0.623
CYTL1	1.50 (0.63-3.53)	0.359	0.863	1.79 (0.52-6.14)	0.357	0.862	0.60 (0.10-3.61)	0.574	0.960
DAAM1	0.99 (0.73-1.35)	0.958	0.999	0.94 (0.53-1.66)	0.819	0.997	0.97 (0.54-1.73)	0.905	0.992
DAB2	0.96 (0.78-1.17)	0.680	0.975	0.99 (0.68-1.44)	0.961	0.997	0.91 (0.62-1.33)	0.634	0.973
DAG1	0.91 (0.55-1.52)	0.724	0.977	1.14 (0.46-2.84)	0.779	0.997	0.64 (0.24-1.69)	0.367	0.939
DAND5	0.73 (0.48-1.11)	0.144	0.661	0.51 (0.25-1.02)	0.056	0.399	0.87 (0.41-1.87)	0.730	0.978
DAPK2	1.03 (0.63-1.69)	0.901	0.999	1.69 (0.85-3.37)	0.135	0.600	0.28 (0.10-0.84)	0.022	0.551
DAPP1	0.97 (0.81-1.17)	0.777	0.992	0.93 (0.67-1.28)	0.652	0.992	0.93 (0.67-1.30)	0.673	0.975
DARS1	0.95 (0.69-1.29)	0.732	0.979	1.06 (0.60-1.88)	0.843	0.997	0.87 (0.50-1.52)	0.619	0.967
DBH	1.40 (1.03-1.91)	0.034	0.392	1.55 (0.84-2.85)	0.156	0.633	1.42 (0.79-2.53)	0.241	0.891
DBI	1.00 (0.73-1.37)	0.992	0.999	0.91 (0.52-1.61)	0.752	0.997	0.96 (0.53-1.74)	0.901	0.992
DBN1	1.07 (0.63-1.80)	0.809	0.995	0.94 (0.36-2.49)	0.905	0.997	1.51 (0.57-4.02)	0.413	0.942
DBNL	0.98 (0.76-1.25)	0.850	0.998	0.98 (0.62-1.54)	0.915	0.997	0.92 (0.57-1.48)	0.717	0.976
DCBLD2	2.37 (1.33-4.24)	0.004	0.137	2.42 (0.84-6.96)	0.100	0.525	1.69 (0.58-4.98)	0.339	0.935
DCC	1.22 (0.49-3.00)	0.668	0.971	1.25 (0.26-5.94)	0.780	0.997	0.82 (0.12-5.73)	0.838	0.991
DCDC2C	0.85 (0.53-1.35)	0.492	0.936	0.94 (0.42-2.13)	0.886	0.997	0.50 (0.20-1.22)	0.128	0.796
DCLRE1C	0.94 (0.41-2.14)	0.874	0.998	1.17 (0.25-5.52)	0.844	0.997	0.16 (0.03-0.81)	0.027	0.588

DCN	3.20 (1.52-6.74)	0.002	0.113	8.69 (2.40-31.38)	<0.001	0.063	3.25 (0.85-12.38)	0.084	0.743
DCTD	1.01 (0.78-1.31)	0.945	0.999	1.13 (0.70-1.83)	0.618	0.979	0.80 (0.49-1.29)	0.359	0.939
DCTN1	1.18 (0.83-1.66)	0.359	0.863	1.06 (0.56-2.00)	0.856	0.997	1.30 (0.67-2.53)	0.442	0.942
DCTN2	0.82 (0.64-1.04)	0.105	0.586	0.81 (0.52-1.25)	0.341	0.847	0.78 (0.49-1.22)	0.273	0.910
DCTN6	0.95 (0.70-1.28)	0.744	0.984	0.94 (0.55-1.62)	0.833	0.997	0.79 (0.42-1.46)	0.448	0.942
DCTPP1	1.18 (0.58-2.41)	0.650	0.971	4.38 (1.32-14.50)	0.016	0.230	1.68 (0.48-5.91)	0.418	0.942
DCUN1D1	1.20 (0.98-1.47)	0.071	0.497	1.18 (0.80-1.76)	0.409	0.903	0.95 (0.59-1.53)	0.841	0.992
DCUN1D2	1.15 (0.88-1.50)	0.305	0.823	1.06 (0.63-1.77)	0.823	0.997	1.02 (0.61-1.69)	0.949	0.992
DCXR	0.71 (0.47-1.06)	0.093	0.560	0.93 (0.46-1.91)	0.852	0.997	0.63 (0.30-1.30)	0.209	0.867
DDA1	1.32 (0.89-1.96)	0.165	0.691	1.66 (1.03-2.66)	0.036	0.326	0.71 (0.19-2.63)	0.608	0.966
DDAH1	1.08 (0.64-1.83)	0.769	0.988	1.44 (0.56-3.70)	0.452	0.920	1.11 (0.43-2.86)	0.833	0.991
DDC	0.61 (0.39-0.96)	0.031	0.376	1.08 (0.48-2.40)	0.860	0.997	0.53 (0.24-1.18)	0.121	0.785
DDHD2	1.01 (0.70-1.47)	0.951	0.999	1.18 (0.60-2.33)	0.628	0.984	0.87 (0.42-1.81)	0.709	0.976
DDI2	0.94 (0.69-1.28)	0.684	0.975	0.61 (0.34-1.10)	0.101	0.526	1.06 (0.62-1.82)	0.823	0.991
DDR1	3.69 (1.45-9.42)	0.006	0.186	15.80 (2.83-88.26)	0.002	0.084	8.08 (1.54-42.45)	0.014	0.447
DDT	0.75 (0.42-1.36)	0.342	0.854	0.89 (0.33-2.42)	0.827	0.997	1.40 (0.59-3.28)	0.445	0.942
DDX1	0.90 (0.66-1.23)	0.506	0.943	1.16 (0.75-1.79)	0.504	0.942	0.94 (0.55-1.60)	0.810	0.991
DDX25	1.23 (0.89-1.69)	0.205	0.738	0.51 (0.15-1.74)	0.284	0.795	1.18 (0.62-2.23)	0.616	0.966
DDX39A	0.71 (0.21-2.42)	0.587	0.960	3.48 (0.51-23.63)	0.202	0.710	0.40 (0.04-3.88)	0.432	0.942
DDX4	0.78 (0.45-1.34)	0.363	0.865	0.93 (0.37-2.36)	0.885	0.997	0.94 (0.37-2.37)	0.895	0.992
DDX53	2.04 (0.96-4.30)	0.062	0.472	1.72 (0.66-4.49)	0.267	0.777	4.22 (1.47-12.10)	0.007	0.370
DDX58	0.92 (0.60-1.41)	0.716	0.975	0.96 (0.44-2.09)	0.923	0.997	0.56 (0.22-1.45)	0.231	0.883
DECR1	1.05 (0.83-1.33)	0.669	0.971	0.92 (0.60-1.42)	0.708	0.995	0.96 (0.61-1.51)	0.857	0.992
DEFA1_DEFA1B	1.26 (0.89-1.79)	0.194	0.720	1.21 (0.61-2.40)	0.582	0.967	1.31 (0.71-2.40)	0.383	0.942

DEFB103A_DEFB103B	0.91 (0.47-1.79)	0.790	0.994	2.94 (1.37-6.31)	0.006	0.146	0.74 (0.21-2.58)	0.635	0.973
DEFB104A_DEFB104B	0.97 (0.67-1.42)	0.893	0.999	0.75 (0.34-1.70)	0.496	0.940	1.66 (0.92-3.00)	0.095	0.762
DEFB116	1.17 (0.86-1.59)	0.318	0.835	1.43 (0.95-2.16)	0.090	0.496	0.86 (0.38-1.95)	0.713	0.976
DEFB118	1.23 (0.87-1.75)	0.247	0.771	0.60 (0.17-2.07)	0.420	0.903	1.34 (0.68-2.63)	0.393	0.942
DEFB4A_DEFB4B	1.02 (0.87-1.19)	0.833	0.997	1.21 (0.91-1.61)	0.180	0.664	0.96 (0.72-1.27)	0.774	0.987
DENND2B	0.95 (0.52-1.74)	0.876	0.998	2.23 (1.25-4.00)	0.007	0.160	0.21 (0.04-1.13)	0.069	0.718
DENR	1.11 (0.71-1.73)	0.658	0.971	1.43 (0.75-2.74)	0.282	0.793	0.95 (0.40-2.23)	0.902	0.992
DFFA	1.01 (0.71-1.43)	0.963	0.999	1.00 (0.54-1.88)	0.991	0.998	1.01 (0.53-1.94)	0.964	0.994
DGCR6	0.87 (0.52-1.45)	0.596	0.962	0.93 (0.37-2.34)	0.883	0.997	0.78 (0.30-2.03)	0.605	0.966
DGKA	0.80 (0.51-1.27)	0.342	0.854	1.17 (0.53-2.57)	0.697	0.994	0.57 (0.23-1.38)	0.211	0.867
DGKZ	1.10 (0.58-2.08)	0.777	0.992	2.47 (1.00-6.12)	0.050	0.391	0.67 (0.19-2.34)	0.533	0.951
DHODH	1.08 (0.60-1.92)	0.798	0.994	0.82 (0.27-2.53)	0.729	0.997	1.47 (0.65-3.35)	0.354	0.938
DHPS	0.86 (0.49-1.51)	0.607	0.963	0.97 (0.36-2.58)	0.947	0.997	0.94 (0.35-2.49)	0.898	0.992
DHRS4L2	1.20 (0.71-2.04)	0.492	0.936	0.91 (0.31-2.71)	0.872	0.997	0.58 (0.16-2.11)	0.410	0.942
DIABLO	1.00 (0.81-1.24)	0.985	0.999	1.03 (0.70-1.52)	0.879	0.997	0.86 (0.58-1.27)	0.447	0.942
DIPK1C	1.30 (0.77-2.18)	0.325	0.843	0.74 (0.24-2.32)	0.610	0.978	1.30 (0.46-3.70)	0.622	0.967
DIPK2B	1.57 (0.66-3.70)	0.306	0.823	9.14 (3.20-26.07)	<0.001	0.015	0.97 (0.19-4.92)	0.972	0.994
DKK1	1.03 (0.70-1.51)	0.877	0.998	1.04 (0.51-2.14)	0.910	0.997	0.82 (0.39-1.69)	0.585	0.965
DKK3	1.18 (0.57-2.43)	0.655	0.971	0.68 (0.18-2.62)	0.574	0.965	2.48 (0.66-9.34)	0.179	0.853
DKK4	1.60 (1.00-2.56)	0.050	0.433	2.04 (0.92-4.52)	0.078	0.471	1.31 (0.56-3.08)	0.532	0.951
DKKL1	1.18 (0.87-1.59)	0.279	0.804	1.16 (0.70-1.94)	0.556	0.962	1.16 (0.66-2.03)	0.614	0.966
DLG4	1.03 (0.72-1.47)	0.859	0.998	1.10 (0.58-2.10)	0.768	0.997	0.69 (0.33-1.45)	0.326	0.932
DLGAP5	1.03 (0.62-1.70)	0.921	0.999	1.58 (0.81-3.09)	0.179	0.664	0.65 (0.25-1.73)	0.392	0.942

DLK1	1.15 (0.80-1.65)	0.459	0.920	1.42 (0.75-2.68)	0.277	0.788	1.76 (0.95-3.27)	0.074	0.718
DLL1	2.07 (1.10-3.89)	0.023	0.349	3.11 (1.08-8.93)	0.035	0.324	2.02 (0.65-6.31)	0.225	0.877
DLL4	0.67 (0.36-1.25)	0.207	0.740	0.62 (0.19-2.04)	0.429	0.908	0.74 (0.27-2.07)	0.571	0.960
DMD	1.03 (0.60-1.76)	0.915	0.999	0.81 (0.28-2.37)	0.698	0.994	1.10 (0.40-2.99)	0.855	0.992
DMP1	1.08 (0.67-1.73)	0.762	0.987	1.77 (0.69-4.53)	0.234	0.742	1.26 (0.52-3.08)	0.611	0.966
DNAJA1	1.23 (0.74-2.05)	0.426	0.902	1.24 (0.51-3.03)	0.629	0.984	0.72 (0.22-2.35)	0.590	0.965
DNAJA2	1.02 (0.77-1.35)	0.880	0.999	0.96 (0.58-1.57)	0.856	0.997	0.96 (0.58-1.60)	0.877	0.992
DNAJA4	0.96 (0.68-1.36)	0.812	0.995	0.86 (0.47-1.58)	0.625	0.983	1.31 (0.75-2.30)	0.342	0.936
DNAJB1	1.16 (0.89-1.52)	0.278	0.804	1.00 (0.63-1.61)	0.987	0.998	1.25 (0.76-2.05)	0.375	0.941
DNAJB14	0.91 (0.60-1.36)	0.639	0.968	1.27 (0.62-2.58)	0.511	0.943	0.73 (0.34-1.58)	0.425	0.942
DNAJB2	1.00 (0.65-1.55)	0.987	0.999	0.91 (0.41-2.02)	0.822	0.997	1.20 (0.60-2.40)	0.601	0.966
DNAJB6	0.83 (0.61-1.12)	0.216	0.746	1.04 (0.61-1.79)	0.874	0.997	0.69 (0.39-1.22)	0.199	0.867
DNAJB8	1.18 (0.82-1.71)	0.370	0.871	1.27 (0.65-2.45)	0.486	0.934	1.05 (0.50-2.19)	0.898	0.992
DNAJC21	0.79 (0.38-1.65)	0.534	0.948	0.69 (0.17-2.77)	0.599	0.973	0.53 (0.12-2.32)	0.398	0.942
DNAJC6	0.95 (0.70-1.30)	0.748	0.984	1.00 (0.57-1.73)	0.989	0.998	0.89 (0.49-1.61)	0.700	0.975
DNAJC9	0.96 (0.71-1.29)	0.773	0.991	1.35 (0.80-2.30)	0.263	0.773	1.09 (0.64-1.86)	0.756	0.985
DNER	0.85 (0.37-1.96)	0.710	0.975	0.87 (0.19-4.09)	0.865	0.997	1.32 (0.30-5.84)	0.712	0.976
DNLZ	1.03 (0.76-1.38)	0.864	0.998	1.26 (0.85-1.87)	0.250	0.756	0.71 (0.35-1.41)	0.324	0.931
DNM1	0.92 (0.74-1.14)	0.438	0.907	1.05 (0.70-1.56)	0.820	0.997	0.68 (0.47-0.99)	0.042	0.633
DNM3	1.59 (1.06-2.38)	0.024	0.352	1.51 (0.73-3.15)	0.268	0.777	1.87 (0.94-3.72)	0.075	0.718
DNMBP	1.17 (0.82-1.66)	0.398	0.894	1.07 (0.55-2.09)	0.844	0.997	1.24 (0.65-2.37)	0.515	0.947
DNPEP	1.11 (0.73-1.68)	0.638	0.968	0.97 (0.46-2.05)	0.929	0.997	0.96 (0.46-2.01)	0.915	0.992
DNPH1	1.17 (0.78-1.77)	0.450	0.916	0.98 (0.47-2.08)	0.964	0.997	1.22 (0.58-2.56)	0.600	0.966
DOC2B	0.96 (0.41-2.25)	0.933	0.999	1.00 (0.22-4.59)	0.996	0.998	0.44 (0.07-2.66)	0.374	0.940
DOCK9	0.91 (0.71-1.16)	0.428	0.904	0.94 (0.62-1.43)	0.782	0.997	0.89 (0.55-1.44)	0.631	0.973

DOK1	1.01 (0.84-1.21)	0.945	0.999	1.08 (0.77-1.52)	0.672	0.993	0.82 (0.58-1.15)	0.242	0.892
DOK2	1.00 (0.83-1.21)	0.962	0.999	1.08 (0.77-1.53)	0.651	0.992	0.89 (0.62-1.27)	0.521	0.948
DPEP1	1.28 (0.82-2.02)	0.277	0.804	1.39 (0.63-3.08)	0.417	0.903	1.05 (0.46-2.40)	0.913	0.992
DPEP2	0.95 (0.45-1.98)	0.891	0.999	0.68 (0.18-2.63)	0.578	0.966	1.16 (0.31-4.37)	0.823	0.991
DPP10	1.42 (0.78-2.58)	0.254	0.782	0.96 (0.32-2.93)	0.945	0.997	0.45 (0.14-1.45)	0.180	0.853
DPP4	1.46 (0.73-2.94)	0.287	0.808	3.31 (0.98-11.18)	0.054	0.392	1.61 (0.45-5.70)	0.462	0.942
DPP6	1.33 (0.73-2.40)	0.347	0.855	2.13 (0.77-5.87)	0.144	0.610	0.93 (0.33-2.63)	0.886	0.992
DPP7	1.17 (0.79-1.73)	0.423	0.901	1.18 (0.58-2.38)	0.648	0.992	0.96 (0.46-2.02)	0.923	0.992
DPT	1.53 (0.64-3.65)	0.343	0.854	1.59 (0.32-7.77)	0.570	0.963	2.01 (0.41-9.91)	0.391	0.942
DPY30	1.25 (0.86-1.81)	0.246	0.771	1.32 (0.67-2.60)	0.420	0.903	1.32 (0.67-2.59)	0.428	0.942
DRAXIN	1.29 (0.79-2.13)	0.308	0.825	2.82 (1.28-6.23)	0.010	0.195	1.94 (0.79-4.75)	0.145	0.808
DRG2	1.01 (0.67-1.53)	0.954	0.999	1.07 (0.50-2.25)	0.867	0.997	0.94 (0.43-2.06)	0.882	0.992
DSC2	1.40 (0.76-2.57)	0.285	0.806	2.09 (0.71-6.11)	0.178	0.662	1.38 (0.46-4.13)	0.567	0.960
DSCAM	1.69 (0.86-3.33)	0.130	0.629	2.41 (0.76-7.64)	0.136	0.600	0.47 (0.13-1.74)	0.259	0.907
DSG2	1.62 (0.73-3.59)	0.234	0.765	0.88 (0.21-3.76)	0.867	0.997	1.81 (0.46-7.13)	0.396	0.942
DSG3	1.65 (0.90-3.04)	0.108	0.593	5.10 (1.86-13.96)	0.002	0.078	0.90 (0.31-2.67)	0.856	0.992
DSG4	1.04 (0.67-1.63)	0.850	0.998	1.83 (0.82-4.09)	0.141	0.604	1.00 (0.44-2.28)	0.998	1.000
DTD1	0.94 (0.70-1.26)	0.670	0.971	1.14 (0.67-1.94)	0.620	0.980	0.65 (0.36-1.16)	0.144	0.808
DTNB	1.13 (0.73-1.75)	0.591	0.961	1.27 (0.57-2.80)	0.561	0.962	2.08 (1.08-3.99)	0.028	0.588
DTX2	0.81 (0.50-1.33)	0.414	0.894	1.27 (0.58-2.76)	0.551	0.960	0.31 (0.10-0.94)	0.039	0.625
DTX3	2.44 (1.18-5.06)	0.016	0.294	5.19 (1.90-14.18)	0.001	0.072	2.00 (0.49-8.24)	0.337	0.934
DTYMK	0.88 (0.62-1.25)	0.488	0.936	1.36 (0.75-2.48)	0.315	0.819	0.72 (0.37-1.37)	0.314	0.923
DUOX2	0.95 (0.67-1.35)	0.789	0.994	1.07 (0.59-1.93)	0.820	0.997	0.49 (0.20-1.17)	0.108	0.763
DUSP13	0.86 (0.46-1.59)	0.627	0.966	2.35 (1.24-4.45)	0.009	0.187	0.50 (0.16-1.59)	0.241	0.891
DUSP29	1.06 (0.78-1.45)	0.698	0.975	1.27 (0.82-1.98)	0.288	0.800	1.08 (0.68-1.73)	0.744	0.984

DUSP3	1.07 (0.81-1.42)	0.643	0.971	1.03 (0.60-1.74)	0.925	0.997	1.44 (0.95-2.19)	0.088	0.745
DUT	1.08 (0.86-1.37)	0.510	0.945	1.11 (0.72-1.69)	0.637	0.987	0.85 (0.55-1.30)	0.449	0.942
DXO	1.05 (0.62-1.77)	0.858	0.998	1.23 (0.48-3.14)	0.671	0.993	0.51 (0.18-1.49)	0.220	0.869
DYNC1H1	1.03 (0.61-1.73)	0.906	0.999	0.93 (0.33-2.65)	0.891	0.997	1.31 (0.56-3.05)	0.534	0.952
DYNLT1	0.84 (0.59-1.20)	0.335	0.851	0.79 (0.41-1.54)	0.490	0.939	0.92 (0.49-1.70)	0.778	0.987
DYNLT3	0.83 (0.44-1.53)	0.545	0.948	0.48 (0.14-1.58)	0.226	0.737	1.10 (0.36-3.37)	0.862	0.992
EBAG9	0.98 (0.77-1.25)	0.888	0.999	0.99 (0.63-1.55)	0.968	0.997	0.88 (0.55-1.41)	0.602	0.966
EBI3_IL27	1.20 (0.61-2.36)	0.604	0.962	0.83 (0.23-2.99)	0.773	0.997	4.92 (1.61-14.99)	0.005	0.343
ECE1	1.32 (0.71-2.46)	0.383	0.876	2.61 (0.84-8.04)	0.096	0.517	1.39 (0.42-4.58)	0.584	0.965
ECHDC3	0.99 (0.71-1.37)	0.948	0.999	1.09 (0.62-1.91)	0.766	0.997	0.71 (0.37-1.38)	0.317	0.923
ECHS1	0.99 (0.79-1.23)	0.916	0.999	0.97 (0.65-1.45)	0.881	0.997	0.94 (0.62-1.44)	0.781	0.987
ECI2	0.85 (0.55-1.34)	0.490	0.936	0.98 (0.45-2.13)	0.962	0.997	0.47 (0.17-1.33)	0.155	0.822
ECM1	1.11 (0.64-1.95)	0.707	0.975	2.00 (0.59-6.76)	0.264	0.773	1.05 (0.38-2.94)	0.926	0.992
ECSCR	1.39 (0.95-2.05)	0.089	0.546	1.57 (0.80-3.09)	0.188	0.680	1.09 (0.48-2.51)	0.834	0.991
EDA2R	2.28 (1.58-3.31)	<0.001	0.007	2.11 (1.05-4.27)	0.037	0.330	2.48 (1.34-4.57)	0.004	0.316
EDAR	1.07 (0.82-1.38)	0.619	0.966	0.86 (0.54-1.38)	0.533	0.951	1.08 (0.67-1.73)	0.764	0.987
EDDM3B	0.68 (0.45-1.05)	0.081	0.519	0.62 (0.28-1.38)	0.240	0.742	0.52 (0.24-1.16)	0.112	0.764
EDEM2	1.04 (0.56-1.94)	0.892	0.999	1.09 (0.39-3.00)	0.870	0.997	1.68 (0.80-3.52)	0.169	0.852
EDF1	1.10 (0.74-1.65)	0.638	0.968	1.21 (0.63-2.32)	0.576	0.965	0.53 (0.20-1.39)	0.197	0.867
EDIL3	1.36 (0.68-2.69)	0.382	0.876	1.18 (0.34-4.09)	0.793	0.997	1.19 (0.35-4.10)	0.778	0.987
EDN1	1.70 (0.78-3.70)	0.183	0.708	5.42 (1.77-16.54)	0.003	0.110	2.32 (0.63-8.51)	0.204	0.867
EDNRB	0.81 (0.39-1.68)	0.579	0.956	0.26 (0.05-1.30)	0.101	0.526	1.48 (0.57-3.86)	0.423	0.942
EEF1D	1.13 (0.86-1.49)	0.386	0.879	1.14 (0.68-1.90)	0.615	0.979	1.05 (0.63-1.78)	0.842	0.992
EFCAB14	1.97 (1.00-3.90)	0.050	0.433	2.86 (1.13-7.20)	0.026	0.281	2.98 (1.16-7.66)	0.023	0.559
EFCAB2	1.09 (0.52-2.28)	0.816	0.995	3.15 (0.98-10.07)	0.053	0.392	0.91 (0.22-3.68)	0.893	0.992

EFEMP1	1.44 (0.76-2.74)	0.269	0.798	3.72 (1.33-10.42)	0.012	0.209	1.58 (0.51-4.95)	0.431	0.942
EFHD1	1.51 (0.87-2.63)	0.141	0.657	1.64 (0.63-4.23)	0.310	0.816	2.24 (1.06-4.75)	0.036	0.623
EFNA1	1.97 (1.10-3.53)	0.023	0.348	1.32 (0.43-4.03)	0.629	0.984	3.13 (1.30-7.53)	0.011	0.429
EFNA4	2.29 (1.41-3.72)	<0.001	0.070	2.62 (1.19-5.79)	0.017	0.236	2.23 (0.93-5.36)	0.073	0.718
EFNB2	1.20 (0.91-1.60)	0.201	0.730	1.29 (0.85-1.96)	0.238	0.742	1.49 (0.99-2.26)	0.059	0.698
EGF	0.95 (0.75-1.19)	0.635	0.968	0.94 (0.61-1.43)	0.764	0.997	0.80 (0.52-1.22)	0.302	0.919
EGFL7	1.49 (0.86-2.57)	0.154	0.676	2.19 (0.85-5.63)	0.104	0.529	1.23 (0.43-3.57)	0.697	0.975
EGFLAM	1.57 (0.93-2.64)	0.090	0.552	2.06 (0.91-4.65)	0.082	0.484	1.55 (0.61-3.93)	0.359	0.939
EGFR	0.50 (0.16-1.59)	0.239	0.768	0.66 (0.07-6.18)	0.716	0.995	0.35 (0.06-2.05)	0.245	0.895
EGLN1	1.12 (0.76-1.66)	0.562	0.956	0.97 (0.43-2.17)	0.944	0.997	1.18 (0.62-2.22)	0.618	0.967
EHBP1	0.90 (0.64-1.27)	0.559	0.954	1.24 (0.69-2.24)	0.476	0.932	0.71 (0.36-1.39)	0.316	0.923
EHD3	0.95 (0.76-1.18)	0.634	0.968	0.91 (0.60-1.37)	0.654	0.992	0.81 (0.54-1.23)	0.330	0.934
EIF1AX	1.06 (0.69-1.62)	0.805	0.995	0.99 (0.44-2.23)	0.985	0.998	1.44 (0.71-2.95)	0.313	0.923
EIF2AK2	0.97 (0.77-1.22)	0.799	0.994	1.25 (0.84-1.86)	0.261	0.772	0.76 (0.48-1.21)	0.251	0.901
EIF2AK3	0.89 (0.61-1.30)	0.548	0.949	0.90 (0.44-1.84)	0.776	0.997	0.82 (0.39-1.71)	0.590	0.965
EIF2S2	1.03 (0.57-1.86)	0.931	0.999	1.37 (0.56-3.34)	0.495	0.940	0.62 (0.16-2.38)	0.483	0.944
EIF4B	0.97 (0.66-1.43)	0.878	0.999	0.96 (0.47-1.93)	0.901	0.997	0.88 (0.43-1.80)	0.730	0.978
EIF4E	0.89 (0.68-1.16)	0.374	0.874	1.15 (0.72-1.84)	0.558	0.962	0.77 (0.47-1.28)	0.320	0.926
EIF4EBP1	1.42 (1.06-1.91)	0.019	0.311	1.39 (0.83-2.31)	0.212	0.719	1.33 (0.80-2.23)	0.271	0.910
EIF4G1	1.13 (0.89-1.43)	0.306	0.823	1.14 (0.74-1.75)	0.552	0.960	1.08 (0.69-1.68)	0.743	0.984
EIF4G3	1.07 (0.74-1.55)	0.713	0.975	1.08 (0.54-2.15)	0.832	0.997	0.86 (0.41-1.83)	0.703	0.975
EIF5	1.51 (0.89-2.57)	0.128	0.626	2.52 (1.28-4.97)	0.008	0.167	1.20 (0.42-3.41)	0.732	0.979
EIF5A	0.90 (0.44-1.87)	0.786	0.994	0.71 (0.17-3.00)	0.641	0.990	0.77 (0.20-3.04)	0.712	0.976
ELAC1	0.80 (0.59-1.08)	0.151	0.673	1.11 (0.64-1.91)	0.710	0.995	0.58 (0.32-1.03)	0.061	0.698
ELAVL4	1.06 (0.79-1.43)	0.681	0.975	0.84 (0.46-1.53)	0.562	0.962	1.17 (0.65-2.08)	0.602	0.966

ELN	2.67 (1.69-4.21)	<0.001	0.009	3.85 (1.64-9.00)	0.002	0.086	2.81 (1.35-5.87)	0.006	0.346
ELOA	1.13 (0.77-1.65)	0.540	0.948	1.20 (0.61-2.38)	0.600	0.973	0.81 (0.35-1.89)	0.627	0.970
ELOB	1.21 (0.74-1.98)	0.451	0.916	2.10 (1.16-3.80)	0.014	0.221	0.66 (0.19-2.32)	0.520	0.948
ENAH	1.15 (0.71-1.85)	0.567	0.956	1.95 (0.90-4.24)	0.090	0.499	1.49 (0.65-3.41)	0.341	0.935
ENDOU	1.03 (0.72-1.48)	0.874	0.998	1.12 (0.58-2.16)	0.746	0.997	0.87 (0.43-1.75)	0.689	0.975
ENG	3.24 (0.93-11.24)	0.064	0.473	11.10 (1.12-109.79)	0.040	0.347	1.12 (0.10-12.83)	0.925	0.992
ENO1	0.91 (0.67-1.23)	0.532	0.948	0.82 (0.48-1.41)	0.479	0.932	0.89 (0.50-1.57)	0.681	0.975
ENO2	0.96 (0.67-1.37)	0.820	0.995	0.96 (0.50-1.86)	0.915	0.997	0.98 (0.50-1.91)	0.941	0.992
ENO3	0.74 (0.51-1.09)	0.126	0.625	1.31 (0.76-2.25)	0.329	0.832	0.90 (0.47-1.70)	0.744	0.984
ENOPH1	0.63 (0.28-1.39)	0.252	0.777	0.56 (0.11-2.79)	0.478	0.932	0.18 (0.02-1.33)	0.093	0.762
ENOX2	0.86 (0.35-2.11)	0.735	0.981	3.13 (1.18-8.30)	0.022	0.261	0.40 (0.06-2.76)	0.350	0.938
ENPEP	1.38 (0.83-2.30)	0.211	0.741	1.67 (0.72-3.84)	0.230	0.740	1.65 (0.71-3.81)	0.241	0.891
ENPP2	0.74 (0.35-1.59)	0.445	0.913	1.25 (0.33-4.77)	0.740	0.997	1.07 (0.26-4.44)	0.929	0.992
ENPP5	0.75 (0.51-1.11)	0.151	0.673	1.25 (0.47-3.33)	0.649	0.992	0.57 (0.30-1.11)	0.100	0.762
ENPP6	1.02 (0.52-1.99)	0.958	0.999	1.21 (0.36-4.08)	0.757	0.997	1.35 (0.37-4.86)	0.648	0.973
ENPP7	1.27 (0.98-1.65)	0.074	0.512	1.79 (1.11-2.87)	0.016	0.234	0.90 (0.55-1.48)	0.675	0.975
ENSA	0.77 (0.44-1.35)	0.358	0.862	1.15 (0.46-2.88)	0.761	0.997	0.24 (0.07-0.86)	0.029	0.588
ENTPD2	1.68 (1.05-2.70)	0.031	0.376	2.64 (1.20-5.84)	0.016	0.234	0.87 (0.33-2.26)	0.773	0.987
ENTPD5	0.78 (0.29-2.09)	0.622	0.966	2.20 (0.33-14.49)	0.413	0.903	3.48 (0.50-24.22)	0.208	0.867
ENTPD6	1.45 (0.63-3.35)	0.379	0.875	5.07 (0.87-29.74)	0.072	0.459	1.28 (0.28-5.87)	0.755	0.985
ENTR1	0.90 (0.59-1.38)	0.629	0.966	1.53 (0.79-2.94)	0.204	0.711	1.02 (0.49-2.11)	0.963	0.994
EP300	1.34 (0.60-3.04)	0.476	0.928	1.80 (0.42-7.61)	0.426	0.906	0.47 (0.08-2.75)	0.400	0.942
EPB41L5	1.03 (0.88-1.21)	0.690	0.975	0.92 (0.62-1.37)	0.680	0.993	0.99 (0.72-1.36)	0.945	0.992
EPCAM	0.89 (0.71-1.13)	0.347	0.855	0.73 (0.46-1.15)	0.178	0.662	0.74 (0.47-1.17)	0.200	0.867
EPGN	1.47 (1.05-2.07)	0.025	0.352	1.69 (1.05-2.72)	0.030	0.306	0.97 (0.35-2.70)	0.956	0.992

EPHA1	0.92 (0.47-1.77)	0.794	0.994	3.21 (0.95-10.86)	0.061	0.421	0.67 (0.21-2.11)	0.494	0.947
EPHA10	1.25 (0.90-1.73)	0.189	0.716	1.13 (0.57-2.24)	0.728	0.997	1.40 (0.85-2.33)	0.189	0.862
EPHA2	2.49 (1.42-4.34)	0.001	0.085	3.05 (1.21-7.68)	0.018	0.243	2.21 (0.78-6.21)	0.134	0.796
EPHA4	2.05 (0.87-4.85)	0.102	0.576	9.52 (2.21-40.96)	0.002	0.106	1.22 (0.26-5.76)	0.803	0.991
EPHB4	1.86 (0.92-3.75)	0.083	0.528	3.15 (0.85-11.60)	0.085	0.486	1.55 (0.42-5.74)	0.516	0.947
EPHB6	1.33 (0.73-2.46)	0.354	0.858	1.58 (0.53-4.68)	0.408	0.903	1.36 (0.45-4.11)	0.587	0.965
EPHX2	1.08 (0.78-1.50)	0.635	0.968	1.21 (0.71-2.08)	0.482	0.934	0.97 (0.49-1.91)	0.932	0.992
EPN1	0.99 (0.59-1.68)	0.972	0.999	1.15 (0.49-2.68)	0.745	0.997	0.62 (0.19-1.99)	0.418	0.942
EPO	1.11 (0.88-1.41)	0.382	0.875	1.24 (0.85-1.80)	0.263	0.773	1.11 (0.72-1.72)	0.637	0.973
EPPK1	0.90 (0.61-1.30)	0.564	0.956	1.15 (0.60-2.20)	0.667	0.993	1.00 (0.52-1.91)	0.990	0.998
EPS8L2	1.06 (0.54-2.09)	0.855	0.998	1.53 (0.47-4.92)	0.478	0.932	2.57 (0.88-7.52)	0.085	0.745
ERBB2	1.07 (0.47-2.45)	0.870	0.998	2.86 (0.60-13.68)	0.188	0.681	1.54 (0.31-7.63)	0.597	0.966
ERBB3	1.16 (0.37-3.67)	0.795	0.994	0.12 (0.01-1.16)	0.067	0.441	2.26 (0.32-16.24)	0.416	0.942
ERBB4	2.17 (0.96-4.88)	0.062	0.472	1.55 (0.38-6.35)	0.545	0.959	0.87 (0.19-4.08)	0.862	0.992
ERBIN	1.03 (0.82-1.28)	0.825	0.995	1.02 (0.68-1.53)	0.935	0.997	0.97 (0.63-1.49)	0.889	0.992
ERC2	1.14 (0.83-1.55)	0.414	0.894	1.26 (0.74-2.15)	0.385	0.888	1.30 (0.82-2.07)	0.269	0.910
ERCC1	0.77 (0.34-1.73)	0.526	0.948	0.72 (0.16-3.29)	0.676	0.993	0.32 (0.07-1.53)	0.152	0.816
EREG	0.92 (0.68-1.24)	0.566	0.956	0.98 (0.56-1.69)	0.931	0.997	0.62 (0.33-1.15)	0.128	0.796
ERI1	1.06 (0.58-1.94)	0.845	0.998	0.99 (0.31-3.11)	0.981	0.998	0.84 (0.26-2.67)	0.767	0.987
ERMAP	1.16 (0.65-2.08)	0.622	0.966	0.82 (0.28-2.44)	0.722	0.995	2.23 (1.00-4.97)	0.051	0.671
ERN1	0.91 (0.47-1.74)	0.770	0.988	0.83 (0.25-2.76)	0.758	0.997	1.48 (0.46-4.73)	0.506	0.947
ERP29	0.90 (0.73-1.11)	0.330	0.846	1.02 (0.69-1.50)	0.921	0.997	0.71 (0.47-1.06)	0.096	0.762
ERP44	0.36 (0.14-0.90)	0.029	0.372	0.22 (0.04-1.22)	0.083	0.486	0.80 (0.15-4.24)	0.794	0.989
ERVV_1	1.48 (0.84-2.63)	0.176	0.706	2.70 (1.27-5.73)	0.010	0.192	0.85 (0.25-2.93)	0.802	0.991
ESAM	1.65 (0.88-3.09)	0.121	0.619	3.24 (1.12-9.35)	0.030	0.305	1.02 (0.30-3.46)	0.971	0.994

ESM1	0.99 (0.51-1.93)	0.983	0.999	1.31 (0.40-4.31)	0.661	0.992	2.04 (0.62-6.74)	0.244	0.893
ESPL1	1.13 (0.73-1.72)	0.587	0.960	1.32 (0.63-2.78)	0.456	0.923	0.53 (0.19-1.51)	0.235	0.885
ESR1	0.99 (0.62-1.58)	0.965	0.999	0.73 (0.27-1.95)	0.527	0.948	0.90 (0.34-2.34)	0.827	0.991
ESYT2	0.94 (0.69-1.28)	0.692	0.975	0.91 (0.51-1.60)	0.733	0.997	0.73 (0.40-1.31)	0.292	0.915
EVI2B	1.42 (0.88-2.28)	0.147	0.669	1.13 (0.35-3.63)	0.837	0.997	0.75 (0.15-3.76)	0.727	0.978
EVI5	1.03 (0.79-1.34)	0.841	0.998	1.21 (0.77-1.90)	0.410	0.903	0.93 (0.56-1.54)	0.768	0.987
EVPL	1.40 (0.65-3.01)	0.390	0.884	1.54 (0.41-5.79)	0.520	0.946	1.34 (0.30-5.90)	0.701	0.975
EXOSC10	0.98 (0.59-1.61)	0.927	0.999	1.11 (0.46-2.69)	0.823	0.997	1.18 (0.51-2.70)	0.703	0.975
EXTL1	1.11 (0.62-2.01)	0.723	0.975	1.05 (0.35-3.13)	0.935	0.997	1.19 (0.42-3.36)	0.739	0.983
EZR	1.15 (0.47-2.81)	0.755	0.986	1.59 (0.33-7.58)	0.559	0.962	1.18 (0.25-5.67)	0.833	0.991
F10	1.00 (0.43-2.30)	1.000	1.000	0.33 (0.08-1.33)	0.120	0.573	0.75 (0.18-3.16)	0.696	0.975
F11	0.40 (0.15-1.03)	0.058	0.463	0.15 (0.03-0.85)	0.032	0.314	0.26 (0.04-1.51)	0.133	0.796
F11R	1.00 (0.66-1.53)	0.992	0.999	1.03 (0.48-2.21)	0.930	0.997	0.93 (0.41-2.11)	0.863	0.992
F12	1.35 (0.70-2.59)	0.375	0.874	2.14 (0.64-7.12)	0.216	0.725	0.94 (0.29-3.06)	0.914	0.992
F13B	0.93 (0.39-2.24)	0.870	0.998	1.05 (0.21-5.38)	0.950	0.997	0.49 (0.10-2.38)	0.380	0.942
F2	0.95 (0.19-4.90)	0.953	0.999	2.50 (0.12-53.42)	0.558	0.962	0.43 (0.02-8.80)	0.586	0.965
F2R	1.14 (0.75-1.74)	0.541	0.948	1.19 (0.55-2.59)	0.655	0.992	0.73 (0.33-1.64)	0.450	0.942
F3	0.98 (0.45-2.14)	0.963	0.999	0.47 (0.11-2.10)	0.325	0.830	1.19 (0.29-4.96)	0.811	0.991
F7	1.18 (0.61-2.28)	0.616	0.966	0.48 (0.15-1.48)	0.202	0.709	0.85 (0.27-2.69)	0.778	0.987
F9	0.55 (0.16-1.87)	0.337	0.852	0.66 (0.07-5.91)	0.711	0.995	0.55 (0.06-5.32)	0.604	0.966
FABP1	1.12 (0.90-1.41)	0.308	0.825	0.97 (0.64-1.47)	0.896	0.997	1.63 (1.11-2.39)	0.012	0.433
FABP2	1.11 (0.85-1.46)	0.445	0.913	1.16 (0.71-1.90)	0.541	0.956	1.61 (1.04-2.49)	0.034	0.623
FABP3	1.46 (1.03-2.08)	0.033	0.386	1.57 (0.88-2.82)	0.129	0.590	1.69 (0.92-3.10)	0.088	0.745
FABP4	1.43 (1.02-2.02)	0.038	0.408	1.23 (0.64-2.34)	0.536	0.952	1.52 (0.84-2.75)	0.166	0.848
FABP5	1.08 (0.77-1.52)	0.651	0.971	1.18 (0.64-2.17)	0.604	0.974	0.93 (0.49-1.76)	0.830	0.991

FABP6	1.05 (0.75-1.48)	0.772	0.990	0.92 (0.48-1.77)	0.798	0.997	1.43 (0.86-2.38)	0.166	0.848
FABP9	1.46 (1.02-2.09)	0.041	0.416	1.65 (0.85-3.22)	0.139	0.604	1.58 (0.82-3.06)	0.175	0.853
FADD	0.91 (0.67-1.24)	0.551	0.949	1.12 (0.64-1.96)	0.687	0.993	0.70 (0.39-1.25)	0.226	0.878
FAM13A	1.14 (0.81-1.62)	0.451	0.916	0.98 (0.50-1.91)	0.949	0.997	1.47 (0.81-2.64)	0.204	0.867
FAM171A2	0.91 (0.76-1.08)	0.276	0.804	1.07 (0.81-1.40)	0.632	0.986	0.60 (0.37-0.96)	0.034	0.618
FAM171B	1.71 (1.03-2.84)	0.039	0.413	1.29 (0.44-3.78)	0.646	0.991	2.94 (1.40-6.14)	0.004	0.316
FAM172A	0.74 (0.47-1.17)	0.198	0.724	0.91 (0.40-2.07)	0.817	0.997	0.72 (0.30-1.72)	0.454	0.942
FAM20A	2.07 (0.89-4.81)	0.092	0.559	8.70 (2.09-36.17)	0.003	0.110	2.66 (0.59-12.10)	0.205	0.867
FAM3B	1.55 (0.80-2.98)	0.192	0.718	2.47 (0.72-8.54)	0.152	0.626	2.72 (0.78-9.46)	0.117	0.780
FAM3C	1.86 (1.01-3.42)	0.045	0.426	2.97 (1.08-8.15)	0.035	0.323	1.51 (0.48-4.80)	0.481	0.944
FAM3D	1.13 (0.85-1.50)	0.397	0.894	0.72 (0.42-1.22)	0.223	0.735	1.42 (0.86-2.34)	0.169	0.852
FAP	1.18 (0.58-2.41)	0.653	0.971	4.18 (1.02-17.05)	0.046	0.373	1.18 (0.32-4.41)	0.807	0.991
FARSA	1.08 (0.74-1.57)	0.690	0.975	1.45 (0.82-2.56)	0.202	0.710	0.68 (0.30-1.58)	0.376	0.941
FAS	1.04 (0.58-1.85)	0.895	0.999	1.53 (0.78-3.01)	0.217	0.725	0.62 (0.16-2.38)	0.486	0.944
FASLG	1.29 (0.70-2.37)	0.411	0.894	0.79 (0.26-2.39)	0.670	0.993	0.83 (0.28-2.52)	0.746	0.985
FBLN2	1.65 (0.89-3.07)	0.110	0.598	6.29 (2.45-16.13)	<0.001	0.027	1.10 (0.37-3.31)	0.862	0.992
FBN2	1.06 (0.80-1.41)	0.670	0.971	0.85 (0.48-1.51)	0.584	0.967	1.28 (0.79-2.07)	0.312	0.923
FBP1	0.91 (0.67-1.22)	0.517	0.946	1.14 (0.68-1.91)	0.619	0.979	0.74 (0.42-1.32)	0.307	0.923
FCAMR	0.94 (0.67-1.32)	0.713	0.975	1.04 (0.56-1.93)	0.894	0.997	1.24 (0.65-2.34)	0.515	0.947
FCAR	1.64 (1.03-2.60)	0.035	0.399	2.79 (1.23-6.36)	0.014	0.223	0.97 (0.41-2.28)	0.948	0.992
FCER1A	2.03 (0.72-5.73)	0.179	0.706	1.59 (0.25-10.20)	0.624	0.983	6.01 (0.79-45.93)	0.084	0.743
FCER2	1.08 (0.72-1.63)	0.695	0.975	1.55 (0.79-3.04)	0.203	0.710	0.91 (0.43-1.94)	0.809	0.991
FCGR2A	0.96 (0.67-1.38)	0.846	0.998	0.57 (0.37-0.88)	0.012	0.206	1.44 (0.67-3.07)	0.346	0.936
FCGR2B	0.84 (0.56-1.27)	0.410	0.894	1.07 (0.52-2.20)	0.850	0.997	0.39 (0.18-0.84)	0.016	0.487
FCGR3B	0.84 (0.54-1.30)	0.430	0.904	1.61 (0.66-3.93)	0.292	0.803	0.42 (0.20-0.91)	0.027	0.588

FCN1	1.30 (0.77-2.21)	0.326	0.843	0.77 (0.35-1.70)	0.516	0.945	2.08 (0.77-5.64)	0.149	0.813
FCN2	0.83 (0.57-1.21)	0.330	0.846	0.98 (0.39-2.43)	0.961	0.997	0.81 (0.39-1.66)	0.562	0.956
FCRL1	1.27 (0.78-2.04)	0.336	0.851	3.08 (1.39-6.82)	0.006	0.146	0.46 (0.19-1.14)	0.094	0.762
FCRL2	1.16 (0.74-1.81)	0.526	0.948	1.92 (0.94-3.92)	0.074	0.464	0.55 (0.23-1.34)	0.190	0.862
FCRL3	1.05 (0.71-1.56)	0.806	0.995	1.43 (0.73-2.80)	0.303	0.813	1.00 (0.49-2.03)	0.999	1.000
FCRL5	1.40 (0.96-2.04)	0.077	0.514	1.73 (0.90-3.31)	0.100	0.525	0.88 (0.44-1.74)	0.713	0.976
FCRL6	1.03 (0.75-1.42)	0.859	0.998	1.11 (0.63-1.95)	0.717	0.995	1.13 (0.64-2.00)	0.669	0.975
FCRLB	1.26 (0.92-1.74)	0.157	0.676	1.50 (0.84-2.69)	0.168	0.649	1.01 (0.51-1.99)	0.985	0.996
FDX1	1.41 (1.03-1.92)	0.030	0.372	1.47 (0.89-2.44)	0.136	0.600	1.26 (0.63-2.52)	0.512	0.947
FDX2	1.15 (0.80-1.66)	0.444	0.913	0.90 (0.42-1.95)	0.797	0.997	1.09 (0.53-2.26)	0.817	0.991
FEN1	1.28 (0.96-1.71)	0.098	0.572	1.26 (0.72-2.23)	0.420	0.903	0.77 (0.37-1.62)	0.496	0.947
FES	0.83 (0.50-1.40)	0.493	0.936	0.43 (0.16-1.11)	0.081	0.483	1.42 (0.63-3.22)	0.400	0.942
FETUB	0.80 (0.39-1.63)	0.534	0.948	1.25 (0.32-4.84)	0.745	0.997	0.98 (0.25-3.81)	0.974	0.994
FGA	0.86 (0.40-1.84)	0.690	0.975	0.67 (0.17-2.63)	0.561	0.962	0.48 (0.12-1.92)	0.298	0.917
FGD3	0.94 (0.68-1.30)	0.711	0.975	1.29 (0.73-2.29)	0.375	0.879	0.67 (0.35-1.27)	0.220	0.869
FGF12	1.03 (0.54-1.97)	0.932	0.999	1.40 (0.54-3.64)	0.495	0.940	0.72 (0.19-2.74)	0.634	0.973
FGF16	1.11 (0.73-1.68)	0.638	0.968	0.63 (0.26-1.54)	0.311	0.816	1.24 (0.58-2.64)	0.575	0.960
FGF19	0.91 (0.69-1.20)	0.512	0.945	1.15 (0.68-1.95)	0.600	0.973	1.05 (0.63-1.75)	0.854	0.992
FGF2	1.02 (0.72-1.44)	0.923	0.999	1.22 (0.66-2.27)	0.528	0.948	0.99 (0.51-1.91)	0.977	0.994
FGF20	1.11 (0.62-1.99)	0.730	0.978	1.80 (0.85-3.85)	0.127	0.589	0.39 (0.07-2.27)	0.296	0.917
FGF21	1.17 (1.00-1.38)	0.055	0.456	0.84 (0.60-1.17)	0.299	0.807	1.50 (1.14-1.98)	0.003	0.306
FGF23	1.57 (1.22-2.02)	<0.001	0.046	1.92 (1.26-2.94)	0.003	0.110	1.89 (1.31-2.73)	<0.001	0.174
FGF3	1.13 (0.53-2.41)	0.755	0.986	0.72 (0.14-3.59)	0.689	0.994	0.72 (0.16-3.31)	0.676	0.975
FGF5	1.45 (0.76-2.78)	0.259	0.787	2.65 (0.84-8.32)	0.095	0.517	2.17 (0.65-7.18)	0.206	0.867
FGF6	1.29 (0.80-2.05)	0.295	0.813	0.96 (0.28-3.22)	0.942	0.997	1.59 (0.98-2.59)	0.061	0.698

FGF7	1.11 (0.82-1.51)	0.498	0.939	1.19 (0.68-2.09)	0.535	0.951	1.11 (0.62-1.98)	0.722	0.977
FGF9	1.11 (0.63-1.93)	0.720	0.975	1.18 (0.41-3.36)	0.756	0.997	1.29 (0.46-3.63)	0.635	0.973
FGFBP1	0.98 (0.53-1.84)	0.961	0.999	0.73 (0.24-2.19)	0.572	0.964	0.54 (0.17-1.70)	0.295	0.916
FGFBP2	1.17 (0.73-1.89)	0.513	0.945	2.62 (1.17-5.88)	0.020	0.252	0.90 (0.37-2.22)	0.823	0.991
FGFBP3	0.94 (0.49-1.79)	0.849	0.998	1.11 (0.32-3.78)	0.870	0.997	0.60 (0.19-1.93)	0.394	0.942
FGFR2	2.62 (1.10-6.27)	0.030	0.372	3.09 (0.66-14.44)	0.152	0.626	3.15 (0.68-14.69)	0.144	0.808
FGFR4	1.03 (0.66-1.61)	0.892	0.999	1.02 (0.46-2.29)	0.961	0.997	1.27 (0.57-2.85)	0.559	0.955
FGL1	1.50 (1.06-2.12)	0.022	0.344	1.08 (0.55-2.11)	0.825	0.997	2.21 (1.26-3.89)	0.006	0.346
FGR	1.23 (0.90-1.68)	0.185	0.711	1.22 (0.67-2.22)	0.508	0.942	0.84 (0.39-1.79)	0.648	0.973
FH	0.79 (0.52-1.18)	0.245	0.771	0.99 (0.53-1.82)	0.964	0.997	0.65 (0.27-1.55)	0.326	0.932
FHIP2A	1.09 (0.60-1.97)	0.776	0.992	1.97 (0.99-3.93)	0.053	0.392	1.14 (0.42-3.13)	0.798	0.990
FHIT	0.93 (0.68-1.27)	0.633	0.968	0.80 (0.46-1.41)	0.440	0.912	1.04 (0.57-1.88)	0.901	0.992
FIS1	1.02 (0.76-1.37)	0.906	0.999	0.82 (0.47-1.44)	0.485	0.934	0.86 (0.49-1.53)	0.617	0.966
FKBP14	0.87 (0.69-1.10)	0.233	0.763	1.16 (0.75-1.77)	0.503	0.941	0.61 (0.39-0.97)	0.035	0.623
FKBP1B	0.89 (0.72-1.10)	0.282	0.806	0.94 (0.64-1.37)	0.744	0.997	0.94 (0.63-1.40)	0.769	0.987
FKBP4	1.00 (0.59-1.71)	0.993	0.999	1.02 (0.39-2.71)	0.961	0.997	1.33 (0.47-3.74)	0.592	0.965
FKBP5	1.00 (0.78-1.27)	0.980	0.999	0.96 (0.62-1.48)	0.847	0.997	0.86 (0.55-1.35)	0.522	0.948
FKBP7	0.76 (0.44-1.32)	0.337	0.852	1.02 (0.45-2.31)	0.966	0.997	0.60 (0.18-2.02)	0.413	0.942
FKBPL	0.80 (0.55-1.15)	0.228	0.761	1.07 (0.56-2.04)	0.833	0.997	0.87 (0.45-1.70)	0.691	0.975
FLI1	0.91 (0.74-1.13)	0.404	0.894	0.73 (0.49-1.09)	0.129	0.590	0.99 (0.66-1.47)	0.950	0.992
FLRT2	1.82 (0.87-3.81)	0.112	0.601	4.12 (1.16-14.64)	0.029	0.295	3.36 (0.92-12.22)	0.066	0.717
FLT1	2.88 (1.18-7.04)	0.020	0.319	3.94 (0.81-19.12)	0.089	0.494	4.49 (0.85-23.72)	0.077	0.724
FLT3	0.68 (0.32-1.47)	0.330	0.846	0.73 (0.18-2.93)	0.659	0.992	0.22 (0.05-0.96)	0.044	0.638
FLT3LG	0.84 (0.45-1.58)	0.591	0.961	2.50 (0.85-7.36)	0.095	0.517	1.41 (0.47-4.22)	0.543	0.952
FLT4	1.05 (0.55-2.03)	0.873	0.998	0.45 (0.17-1.25)	0.126	0.587	1.06 (0.32-3.47)	0.924	0.992

FMNL1	1.12 (0.81-1.55)	0.508	0.944	1.11 (0.60-2.08)	0.737	0.997	1.06 (0.58-1.94)	0.847	0.992
FMR1	0.73 (0.38-1.42)	0.362	0.865	0.35 (0.10-1.20)	0.095	0.517	0.80 (0.25-2.51)	0.696	0.975
FN1	0.95 (0.41-2.22)	0.904	0.999	1.09 (0.24-4.94)	0.907	0.997	0.42 (0.09-1.96)	0.267	0.910
FNDC1	1.18 (0.73-1.90)	0.492	0.936	2.62 (1.03-6.67)	0.043	0.362	2.56 (1.06-6.23)	0.038	0.623
FNTA	1.01 (0.69-1.50)	0.947	0.999	0.91 (0.41-2.01)	0.809	0.997	0.97 (0.47-2.00)	0.941	0.992
FOLH1	1.13 (0.91-1.40)	0.280	0.804	1.05 (0.68-1.64)	0.812	0.997	0.97 (0.57-1.66)	0.916	0.992
FOLR1	1.83 (0.94-3.57)	0.075	0.512	2.24 (0.72-6.96)	0.162	0.639	2.30 (0.70-7.55)	0.168	0.852
FOLR2	1.43 (0.72-2.84)	0.311	0.827	2.26 (0.70-7.31)	0.173	0.660	1.23 (0.36-4.23)	0.744	0.984
FOLR3	1.02 (0.91-1.14)	0.696	0.975	1.01 (0.82-1.24)	0.931	0.997	0.94 (0.74-1.20)	0.621	0.967
FOS	1.04 (0.52-2.06)	0.915	0.999	1.65 (0.61-4.44)	0.320	0.824	0.35 (0.10-1.26)	0.109	0.763
FOSB	1.10 (0.56-2.19)	0.777	0.992	1.02 (0.28-3.69)	0.973	0.997	0.42 (0.09-1.85)	0.250	0.901
FOXJ3	0.84 (0.43-1.62)	0.599	0.962	1.01 (0.31-3.30)	0.983	0.998	0.79 (0.22-2.76)	0.707	0.976
FOXO1	1.01 (0.77-1.32)	0.955	0.999	0.90 (0.54-1.47)	0.663	0.992	0.94 (0.56-1.57)	0.812	0.991
FOXO3	1.07 (0.85-1.34)	0.571	0.956	0.91 (0.58-1.41)	0.663	0.992	1.20 (0.81-1.78)	0.369	0.939
FRMD4B	1.15 (0.80-1.65)	0.466	0.922	0.84 (0.40-1.74)	0.630	0.984	0.99 (0.47-2.05)	0.973	0.994
FRMD7	0.87 (0.40-1.88)	0.720	0.975	1.83 (0.67-5.00)	0.237	0.742	0.88 (0.21-3.81)	0.868	0.992
FRZB	0.61 (0.26-1.43)	0.257	0.786	0.38 (0.09-1.71)	0.208	0.712	0.27 (0.06-1.16)	0.077	0.724
FSHB	1.31 (1.02-1.68)	0.032	0.377	0.90 (0.54-1.50)	0.681	0.993	1.76 (1.19-2.59)	0.005	0.335
FST	0.90 (0.59-1.39)	0.645	0.971	0.93 (0.44-2.00)	0.862	0.997	2.01 (0.95-4.27)	0.070	0.718
FSTL1	2.48 (0.86-7.11)	0.092	0.559	6.94 (1.08-44.59)	0.041	0.350	9.32 (1.51-57.62)	0.016	0.490
FSTL3	1.85 (1.06-3.23)	0.031	0.373	2.97 (1.27-6.93)	0.012	0.203	2.45 (0.97-6.21)	0.059	0.698
FTCD	0.85 (0.66-1.10)	0.208	0.740	0.87 (0.55-1.36)	0.533	0.951	0.86 (0.54-1.38)	0.544	0.952
FUCA1	1.24 (0.94-1.65)	0.128	0.626	1.36 (0.81-2.28)	0.238	0.742	1.24 (0.75-2.06)	0.396	0.942
FUOM	0.64 (0.46-0.91)	0.013	0.272	0.93 (0.49-1.76)	0.815	0.997	0.56 (0.30-1.05)	0.071	0.718
FURIN	1.01 (0.54-1.87)	0.984	0.999	1.67 (0.54-5.17)	0.370	0.874	1.09 (0.36-3.33)	0.882	0.992

FUS	1.26 (0.73-2.18)	0.410	0.894	0.98 (0.33-2.92)	0.967	0.997	1.33 (0.49-3.62)	0.581	0.964
FUT1	0.88 (0.58-1.35)	0.567	0.956	1.00 (0.49-2.02)	0.995	0.998	0.95 (0.47-1.90)	0.874	0.992
FUT3_FUT5	1.07 (0.68-1.69)	0.759	0.987	1.68 (0.74-3.81)	0.214	0.721	1.11 (0.50-2.47)	0.806	0.991
FUT8	1.09 (0.74-1.61)	0.662	0.971	1.43 (0.71-2.87)	0.316	0.819	0.54 (0.25-1.17)	0.118	0.780
FXN	0.93 (0.68-1.27)	0.649	0.971	0.83 (0.47-1.47)	0.518	0.945	0.93 (0.51-1.67)	0.803	0.991
FXYD5	1.04 (0.72-1.51)	0.816	0.995	0.95 (0.48-1.89)	0.886	0.997	1.13 (0.57-2.26)	0.724	0.978
FYB1	0.94 (0.73-1.20)	0.611	0.965	1.21 (0.76-1.92)	0.426	0.905	0.75 (0.46-1.22)	0.249	0.901
FZD10	1.19 (0.87-1.64)	0.274	0.801	1.20 (0.69-2.08)	0.524	0.948	1.49 (0.97-2.30)	0.068	0.718
FZD8	1.90 (1.06-3.40)	0.031	0.372	1.99 (0.68-5.77)	0.207	0.712	2.52 (1.06-6.02)	0.037	0.623
GABARAP	1.33 (0.82-2.16)	0.250	0.775	0.91 (0.32-2.58)	0.854	0.997	1.38 (0.56-3.39)	0.486	0.944
GABARAPL1	1.17 (0.87-1.58)	0.299	0.817	0.94 (0.50-1.78)	0.859	0.997	1.41 (0.82-2.42)	0.217	0.869
GABRA4	0.85 (0.59-1.23)	0.401	0.894	0.69 (0.32-1.47)	0.337	0.841	1.04 (0.56-1.91)	0.909	0.992
GAD1	1.06 (0.38-2.98)	0.910	0.999	4.22 (0.63-28.24)	0.138	0.602	0.21 (0.04-1.06)	0.059	0.698
GAD2	0.93 (0.49-1.79)	0.831	0.997	1.26 (0.49-3.26)	0.633	0.986	0.25 (0.05-1.22)	0.086	0.745
GADD45B	0.93 (0.50-1.74)	0.817	0.995	0.78 (0.22-2.77)	0.701	0.994	1.28 (0.49-3.38)	0.613	0.966
GADD45GIP1	0.70 (0.42-1.18)	0.182	0.708	0.53 (0.20-1.43)	0.209	0.713	0.58 (0.21-1.59)	0.290	0.915
GAGE2A	0.92 (0.65-1.30)	0.638	0.968	0.95 (0.50-1.82)	0.877	0.997	0.57 (0.24-1.37)	0.209	0.867
GAL	1.01 (0.73-1.40)	0.947	0.999	1.11 (0.61-2.03)	0.740	0.997	0.93 (0.51-1.71)	0.817	0.991
GALNT10	1.95 (0.87-4.36)	0.103	0.578	2.44 (0.57-10.56)	0.232	0.742	2.54 (0.60-10.74)	0.204	0.867
GALNT2	0.90 (0.42-1.94)	0.791	0.994	1.27 (0.31-5.12)	0.740	0.997	1.22 (0.32-4.70)	0.775	0.987
GALNT3	1.33 (0.79-2.22)	0.282	0.806	1.64 (0.65-4.10)	0.295	0.803	1.35 (0.54-3.39)	0.519	0.948
GALNT5	2.61 (1.40-4.87)	0.002	0.116	3.65 (1.48-9.02)	0.005	0.146	4.07 (1.34-12.35)	0.013	0.437
GALNT7	1.06 (0.40-2.81)	0.914	0.999	2.79 (0.59-13.24)	0.196	0.700	0.40 (0.06-2.59)	0.336	0.934
GAMT	1.26 (0.66-2.40)	0.484	0.933	1.96 (0.85-4.52)	0.112	0.554	0.30 (0.04-2.02)	0.214	0.867
GAPDH	2.43 (0.95-6.22)	0.063	0.473	0.77 (0.17-3.48)	0.735	0.997	2.88 (0.52-15.97)	0.226	0.878

GART	0.34 (0.10-1.21)	0.096	0.568	0.68 (0.09-5.31)	0.711	0.995	0.35 (0.04-3.33)	0.361	0.939
GAS2	1.20 (0.82-1.75)	0.353	0.858	0.99 (0.46-2.14)	0.987	0.998	1.72 (1.07-2.76)	0.024	0.573
GAS6	1.38 (0.61-3.14)	0.445	0.913	1.91 (0.42-8.60)	0.400	0.901	2.41 (0.52-11.15)	0.261	0.910
GASK1A	1.41 (0.84-2.37)	0.190	0.718	3.41 (1.46-7.95)	0.004	0.140	1.47 (0.59-3.66)	0.413	0.942
GAST	1.12 (1.00-1.26)	0.059	0.463	1.09 (0.88-1.36)	0.429	0.908	1.09 (0.87-1.37)	0.455	0.942
GATA3	1.03 (0.46-2.33)	0.935	0.999	3.24 (0.95-11.03)	0.060	0.414	1.14 (0.25-5.15)	0.867	0.992
GATD3	0.86 (0.63-1.18)	0.355	0.859	0.73 (0.41-1.30)	0.287	0.800	0.86 (0.47-1.56)	0.615	0.966
GBA	1.01 (0.66-1.56)	0.948	0.999	1.02 (0.46-2.25)	0.969	0.997	0.79 (0.32-1.95)	0.614	0.966
GBP1	0.77 (0.51-1.15)	0.202	0.732	1.02 (0.48-2.15)	0.967	0.997	0.64 (0.30-1.37)	0.250	0.901
GBP2	0.78 (0.51-1.18)	0.238	0.768	1.18 (0.56-2.48)	0.661	0.992	0.62 (0.29-1.35)	0.227	0.878
GBP4	1.34 (0.92-1.93)	0.123	0.620	1.03 (0.50-2.14)	0.934	0.997	1.17 (0.57-2.40)	0.671	0.975
GBP6	1.24 (0.75-2.04)	0.406	0.894	0.55 (0.14-2.08)	0.376	0.880	0.56 (0.15-2.12)	0.394	0.942
GC	0.53 (0.20-1.41)	0.203	0.734	0.31 (0.05-1.92)	0.207	0.712	0.65 (0.11-3.73)	0.628	0.970
GCC1	0.98 (0.79-1.24)	0.895	0.999	0.98 (0.65-1.47)	0.908	0.997	0.83 (0.54-1.28)	0.403	0.942
GCG	1.03 (0.86-1.23)	0.768	0.987	1.02 (0.74-1.41)	0.897	0.997	1.19 (0.85-1.65)	0.313	0.923
GCHFR	0.84 (0.49-1.42)	0.510	0.945	1.34 (0.59-3.06)	0.487	0.935	1.34 (0.62-2.89)	0.458	0.942
GCLM	1.24 (0.72-2.13)	0.441	0.909	1.25 (0.48-3.24)	0.648	0.992	0.71 (0.21-2.34)	0.569	0.960
GCNT1	0.98 (0.52-1.87)	0.959	0.999	3.04 (1.02-9.11)	0.047	0.373	0.80 (0.25-2.61)	0.713	0.976
GDF15	1.88 (1.35-2.62)	<0.001	0.043	1.53 (0.80-2.92)	0.197	0.702	2.28 (1.35-3.85)	0.002	0.228
GDF2	1.72 (0.97-3.06)	0.062	0.472	3.01 (1.10-8.23)	0.032	0.314	2.08 (0.72-6.04)	0.177	0.853
GDNF	1.21 (0.69-2.12)	0.504	0.943	1.48 (0.55-3.98)	0.433	0.908	0.66 (0.21-2.07)	0.479	0.944
GET3	1.09 (0.68-1.74)	0.715	0.975	1.35 (0.66-2.80)	0.412	0.903	1.29 (0.55-3.01)	0.552	0.955
GFAP	2.90 (1.84-4.57)	<0.001	0.005	5.47 (2.36-12.67)	<0.001	0.022	3.53 (1.53-8.16)	0.003	0.306
GFER	0.89 (0.62-1.28)	0.546	0.949	0.74 (0.37-1.46)	0.381	0.886	0.92 (0.46-1.85)	0.819	0.991
GFOD2	1.14 (0.98-1.31)	0.080	0.517	1.17 (0.90-1.52)	0.253	0.761	0.86 (0.57-1.31)	0.488	0.946

GFRA1	2.28 (1.18-4.42)	0.014	0.286	3.10 (0.97-9.88)	0.056	0.399	3.55 (1.12-11.29)	0.032	0.610
GFRA2	1.05 (0.37-2.95)	0.925	0.999	3.08 (0.47-20.26)	0.241	0.744	0.77 (0.11-5.22)	0.789	0.989
GFRA3	2.36 (1.16-4.84)	0.018	0.308	1.24 (0.32-4.72)	0.757	0.997	4.98 (1.32-18.85)	0.018	0.512
GFRAL	0.98 (0.72-1.34)	0.919	0.999	0.89 (0.51-1.57)	0.687	0.993	0.89 (0.51-1.55)	0.680	0.975
GGA1	1.00 (0.70-1.42)	0.989	0.999	0.90 (0.44-1.82)	0.762	0.997	0.92 (0.43-1.98)	0.838	0.991
GGACT	0.79 (0.56-1.11)	0.180	0.706	1.42 (0.78-2.59)	0.248	0.753	0.48 (0.24-0.93)	0.029	0.588
GGCT	0.60 (0.35-1.03)	0.065	0.474	1.24 (0.50-3.13)	0.641	0.990	0.60 (0.22-1.60)	0.306	0.923
GGH	0.92 (0.49-1.74)	0.802	0.995	1.52 (0.59-3.87)	0.385	0.888	0.70 (0.21-2.28)	0.551	0.955
GGT1	1.12 (0.76-1.65)	0.559	0.954	1.67 (0.91-3.05)	0.097	0.520	0.74 (0.33-1.66)	0.468	0.942
GGT5	1.79 (0.77-4.15)	0.173	0.701	2.43 (0.53-11.17)	0.254	0.763	1.06 (0.22-5.10)	0.946	0.992
GH1	1.02 (0.90-1.17)	0.736	0.981	0.91 (0.71-1.17)	0.474	0.932	1.09 (0.85-1.38)	0.503	0.947
GH2	1.11 (0.99-1.25)	0.078	0.514	1.05 (0.83-1.32)	0.701	0.994	1.23 (1.01-1.50)	0.039	0.623
GHR	0.90 (0.37-2.20)	0.817	0.995	0.96 (0.19-4.93)	0.964	0.997	1.38 (0.24-8.01)	0.717	0.976
GHRHR	1.15 (0.84-1.56)	0.383	0.876	1.25 (0.74-2.12)	0.409	0.903	1.16 (0.65-2.07)	0.607	0.966
GHRL	1.07 (0.82-1.40)	0.629	0.966	0.92 (0.57-1.48)	0.721	0.995	1.26 (0.78-2.02)	0.348	0.937
GID8	0.66 (0.27-1.62)	0.362	0.865	0.61 (0.11-3.30)	0.563	0.962	0.22 (0.04-1.22)	0.084	0.743
GIGYF2	1.02 (0.74-1.42)	0.888	0.999	1.03 (0.57-1.89)	0.912	0.997	0.89 (0.47-1.68)	0.724	0.978
GIMAP7	0.99 (0.43-2.26)	0.981	0.999	3.03 (1.46-6.28)	0.003	0.110	0.33 (0.06-1.88)	0.211	0.867
GIMAP8	1.23 (0.96-1.58)	0.097	0.569	1.48 (1.14-1.91)	0.003	0.119	1.15 (0.62-2.12)	0.666	0.975
GIP	1.43 (0.92-2.21)	0.110	0.598	1.72 (0.84-3.56)	0.140	0.604	1.17 (0.51-2.66)	0.713	0.976
GIPC2	1.69 (0.92-3.10)	0.089	0.546	1.36 (0.45-4.08)	0.584	0.967	2.88 (1.18-7.03)	0.020	0.539
GIPC3	0.93 (0.73-1.18)	0.537	0.948	1.02 (0.66-1.58)	0.933	0.997	0.60 (0.39-0.92)	0.019	0.519
GIPR	0.82 (0.60-1.10)	0.185	0.710	0.84 (0.48-1.45)	0.522	0.947	0.41 (0.14-1.21)	0.107	0.763
GIT1	1.06 (0.82-1.38)	0.647	0.971	1.13 (0.70-1.82)	0.610	0.978	1.10 (0.66-1.80)	0.721	0.977
GJA8	1.19 (0.88-1.63)	0.265	0.792	1.04 (0.53-2.02)	0.911	0.997	0.80 (0.33-1.91)	0.608	0.966

GKN1	1.71 (0.78-3.75)	0.182	0.708	2.54 (0.74-8.67)	0.136	0.600	2.10 (0.62-7.10)	0.232	0.884
GLA	0.96 (0.51-1.82)	0.908	0.999	1.23 (0.39-3.93)	0.723	0.995	1.10 (0.35-3.48)	0.876	0.992
GLB1	0.96 (0.57-1.60)	0.863	0.998	1.34 (0.56-3.19)	0.509	0.942	0.82 (0.32-2.14)	0.687	0.975
GLI2	1.04 (0.42-2.58)	0.936	0.999	1.39 (0.29-6.71)	0.686	0.993	0.94 (0.15-5.86)	0.947	0.992
GLO1	1.47 (1.04-2.08)	0.030	0.372	1.14 (0.59-2.22)	0.696	0.994	1.48 (0.80-2.74)	0.212	0.867
GLOD4	1.08 (0.57-2.05)	0.805	0.995	1.02 (0.32-3.24)	0.972	0.997	1.10 (0.35-3.48)	0.867	0.992
GLP1R	1.09 (0.65-1.82)	0.756	0.986	1.04 (0.45-2.42)	0.919	0.997	0.84 (0.31-2.30)	0.734	0.981
GLRX	1.06 (0.73-1.54)	0.765	0.987	0.60 (0.29-1.25)	0.171	0.656	1.06 (0.56-1.99)	0.868	0.992
GLRX5	1.01 (0.74-1.36)	0.972	0.999	1.25 (0.74-2.12)	0.401	0.902	0.82 (0.45-1.48)	0.508	0.947
GLT8D2	1.16 (0.72-1.85)	0.544	0.948	1.08 (0.42-2.80)	0.875	0.997	1.71 (0.96-3.06)	0.069	0.718
GLYR1	1.01 (0.63-1.63)	0.953	0.999	1.33 (0.63-2.82)	0.452	0.920	0.84 (0.31-2.32)	0.741	0.983
GM2A	1.50 (1.08-2.09)	0.016	0.294	1.46 (0.79-2.68)	0.230	0.739	2.30 (1.37-3.86)	0.002	0.228
GMFG	0.89 (0.62-1.28)	0.535	0.948	1.27 (0.67-2.43)	0.464	0.926	0.54 (0.27-1.11)	0.093	0.762
GMPR	0.95 (0.61-1.49)	0.838	0.998	0.66 (0.30-1.47)	0.311	0.816	2.05 (0.85-4.96)	0.109	0.763
GMPR2	0.78 (0.53-1.15)	0.210	0.741	0.95 (0.47-1.91)	0.891	0.997	0.77 (0.38-1.55)	0.466	0.942
GNAS	0.83 (0.61-1.13)	0.243	0.771	1.08 (0.67-1.75)	0.757	0.997	0.87 (0.50-1.50)	0.612	0.966
GNE	0.68 (0.47-1.00)	0.049	0.430	0.68 (0.34-1.36)	0.279	0.788	0.67 (0.33-1.38)	0.277	0.910
GNGT1	1.07 (0.66-1.74)	0.773	0.991	0.60 (0.20-1.79)	0.363	0.866	0.52 (0.16-1.72)	0.287	0.915
GNLY	1.60 (1.06-2.42)	0.026	0.352	1.16 (0.56-2.40)	0.698	0.994	1.89 (0.88-4.02)	0.100	0.762
GNPDA1	0.93 (0.38-2.26)	0.867	0.998	0.55 (0.09-3.34)	0.513	0.944	1.26 (0.39-4.04)	0.694	0.975
GNPDA2	1.08 (0.56-2.10)	0.821	0.995	2.76 (0.88-8.67)	0.081	0.483	0.53 (0.14-1.93)	0.332	0.934
GOLGA3	0.91 (0.65-1.27)	0.582	0.957	1.05 (0.59-1.89)	0.859	0.997	0.71 (0.35-1.41)	0.325	0.932
GOLM2	1.08 (0.42-2.78)	0.880	0.999	1.70 (0.30-9.76)	0.552	0.960	1.69 (0.30-9.36)	0.550	0.955
GOPC	1.04 (0.83-1.31)	0.712	0.975	0.85 (0.57-1.27)	0.430	0.908	1.01 (0.66-1.53)	0.966	0.994
GORASP2	0.84 (0.50-1.41)	0.506	0.943	1.24 (0.50-3.09)	0.646	0.991	0.62 (0.22-1.77)	0.370	0.939

GOT1	0.70 (0.41-1.18)	0.176	0.706	1.20 (0.51-2.81)	0.671	0.993	1.52 (0.70-3.33)	0.291	0.915
GP1BA	1.04 (0.56-1.96)	0.894	0.999	1.90 (0.60-6.05)	0.277	0.788	0.88 (0.28-2.78)	0.833	0.991
GP1BB	0.82 (0.56-1.19)	0.288	0.809	1.34 (0.68-2.63)	0.397	0.898	0.42 (0.19-0.91)	0.029	0.588
GP2	0.86 (0.67-1.10)	0.240	0.769	0.97 (0.64-1.47)	0.901	0.997	0.91 (0.57-1.44)	0.676	0.975
GP5	0.92 (0.44-1.93)	0.821	0.995	1.07 (0.27-4.28)	0.926	0.997	0.52 (0.14-1.96)	0.333	0.934
GP6	0.92 (0.70-1.21)	0.566	0.956	0.99 (0.60-1.64)	0.981	0.998	0.78 (0.47-1.29)	0.326	0.932
GPA33	0.87 (0.74-1.02)	0.089	0.546	0.82 (0.60-1.12)	0.208	0.712	0.78 (0.58-1.06)	0.118	0.780
GPC1	1.15 (0.55-2.39)	0.705	0.975	3.19 (0.86-11.80)	0.082	0.484	2.08 (0.55-7.78)	0.279	0.910
GPC5	0.90 (0.61-1.34)	0.616	0.966	0.74 (0.36-1.52)	0.412	0.903	1.16 (0.55-2.44)	0.698	0.975
GPD1	0.89 (0.57-1.39)	0.600	0.962	0.88 (0.40-1.93)	0.754	0.997	1.49 (0.73-3.04)	0.276	0.910
GPHA2	0.96 (0.70-1.31)	0.779	0.992	0.50 (0.16-1.54)	0.227	0.738	1.28 (0.83-1.96)	0.262	0.910
GPI	0.87 (0.50-1.52)	0.623	0.966	1.54 (0.60-3.97)	0.368	0.873	0.58 (0.19-1.76)	0.336	0.934
GPIHBP1	1.11 (0.76-1.62)	0.576	0.956	1.47 (0.73-2.98)	0.279	0.788	1.36 (0.67-2.75)	0.395	0.942
GPKOW	1.21 (0.59-2.45)	0.605	0.963	1.62 (0.54-4.85)	0.389	0.892	1.40 (0.51-3.85)	0.515	0.947
GPNMB	1.87 (0.89-3.95)	0.100	0.573	5.25 (1.37-20.13)	0.015	0.230	0.94 (0.25-3.60)	0.931	0.992
GPR101	0.85 (0.59-1.23)	0.379	0.875	0.49 (0.19-1.30)	0.153	0.626	0.70 (0.31-1.63)	0.411	0.942
GPR158	1.04 (0.71-1.54)	0.837	0.998	0.48 (0.15-1.50)	0.208	0.712	1.52 (0.86-2.69)	0.152	0.816
GPR15L	1.23 (0.84-1.81)	0.280	0.804	1.12 (0.54-2.34)	0.765	0.997	1.00 (0.50-2.00)	0.998	1.000
GPR37	1.27 (0.94-1.72)	0.122	0.619	1.23 (0.71-2.13)	0.467	0.926	1.71 (1.02-2.87)	0.043	0.634
GPRC5C	1.16 (0.82-1.62)	0.403	0.894	1.33 (0.77-2.30)	0.312	0.817	1.05 (0.53-2.08)	0.896	0.992
GRAP2	1.00 (0.84-1.19)	0.997	0.999	1.09 (0.78-1.52)	0.617	0.979	0.89 (0.64-1.24)	0.490	0.947
GRHPR	0.83 (0.57-1.19)	0.310	0.826	0.98 (0.50-1.91)	0.950	0.997	0.72 (0.36-1.43)	0.351	0.938
GRIK2	1.44 (0.92-2.26)	0.112	0.600	2.94 (1.59-5.45)	<0.001	0.059	0.72 (0.29-1.80)	0.478	0.944
GRIN2B	1.04 (0.68-1.60)	0.851	0.998	0.81 (0.33-1.96)	0.637	0.987	1.08 (0.51-2.30)	0.834	0.991
GRK5	0.74 (0.44-1.25)	0.260	0.787	0.78 (0.32-1.92)	0.592	0.970	0.73 (0.27-1.94)	0.526	0.949

GRN	2.75 (1.11-6.81)	0.028	0.370	10.70 (2.24-51.23)	0.003	0.110	5.65 (1.17-27.40)	0.032	0.610
GRP	0.87 (0.55-1.36)	0.532	0.948	1.31 (0.61-2.79)	0.485	0.934	0.96 (0.41-2.22)	0.920	0.992
GRPEL1	1.08 (0.75-1.55)	0.683	0.975	0.97 (0.49-1.92)	0.939	0.997	0.86 (0.41-1.80)	0.694	0.975
GRSF1	1.18 (0.86-1.61)	0.304	0.822	1.06 (0.59-1.89)	0.844	0.997	0.65 (0.33-1.29)	0.220	0.869
GSAP	1.10 (0.90-1.35)	0.359	0.863	0.95 (0.62-1.44)	0.800	0.997	0.89 (0.54-1.46)	0.635	0.973
GSN	0.53 (0.17-1.63)	0.269	0.798	4.20 (0.45-39.21)	0.208	0.712	1.35 (0.17-10.39)	0.776	0.987
GSR	1.33 (0.46-3.84)	0.602	0.962	7.32 (1.15-46.51)	0.035	0.324	1.32 (0.20-8.61)	0.772	0.987
GSTA1	0.87 (0.68-1.12)	0.294	0.813	0.98 (0.63-1.53)	0.940	0.997	0.77 (0.48-1.25)	0.297	0.917
GSTA3	0.76 (0.56-1.02)	0.063	0.472	0.85 (0.51-1.41)	0.518	0.945	0.58 (0.32-1.05)	0.071	0.718
GSTM4	1.14 (0.84-1.56)	0.401	0.894	1.22 (0.72-2.07)	0.463	0.926	1.16 (0.69-1.93)	0.578	0.962
GSTP1	0.67 (0.45-1.00)	0.049	0.430	0.56 (0.27-1.17)	0.121	0.576	0.60 (0.29-1.25)	0.170	0.853
GSTT2B	1.00 (0.90-1.11)	0.974	0.999	1.11 (0.90-1.37)	0.311	0.816	0.93 (0.76-1.13)	0.443	0.942
GTF2IRD1	1.05 (0.75-1.46)	0.792	0.994	1.38 (0.89-2.14)	0.145	0.611	1.07 (0.61-1.89)	0.814	0.991
GTPBP2	0.90 (0.65-1.25)	0.528	0.948	1.07 (0.60-1.92)	0.808	0.997	0.59 (0.31-1.14)	0.119	0.783
GUCA2A	1.29 (0.76-2.22)	0.347	0.855	1.67 (0.70-4.02)	0.250	0.756	1.41 (0.56-3.53)	0.461	0.942
GUCY2C	1.45 (1.18-1.78)	<0.001	0.046	1.85 (1.36-2.52)	<0.001	0.024	1.01 (0.60-1.71)	0.963	0.994
GUK1	1.03 (0.62-1.72)	0.900	0.999	0.82 (0.29-2.31)	0.701	0.994	0.68 (0.23-1.99)	0.484	0.944
GUSB	1.22 (0.90-1.67)	0.202	0.732	1.15 (0.66-1.99)	0.616	0.979	1.52 (0.87-2.64)	0.141	0.808
GYS1	1.00 (0.78-1.27)	0.972	0.999	1.01 (0.65-1.59)	0.950	0.997	0.90 (0.56-1.44)	0.666	0.975
GZMA	0.68 (0.35-1.33)	0.264	0.791	0.56 (0.16-1.90)	0.350	0.857	0.49 (0.14-1.66)	0.251	0.901
GZMB	0.86 (0.59-1.26)	0.442	0.911	0.55 (0.27-1.15)	0.111	0.550	0.64 (0.31-1.34)	0.236	0.885
GZMH	0.91 (0.65-1.26)	0.554	0.950	0.61 (0.32-1.17)	0.136	0.600	0.62 (0.33-1.17)	0.142	0.808
H2AP	0.77 (0.36-1.65)	0.499	0.939	1.33 (0.51-3.47)	0.563	0.962	0.62 (0.14-2.85)	0.542	0.952
HADH	1.18 (0.89-1.58)	0.251	0.776	0.98 (0.52-1.85)	0.952	0.997	0.93 (0.49-1.78)	0.829	0.991
HAGH	1.12 (0.84-1.50)	0.446	0.914	0.88 (0.52-1.50)	0.648	0.992	1.19 (0.71-1.99)	0.516	0.947

HAO1	0.98 (0.83-1.16)	0.813	0.995	0.88 (0.64-1.21)	0.435	0.909	1.06 (0.78-1.45)	0.692	0.975
HARS1	0.94 (0.70-1.28)	0.708	0.975	1.00 (0.57-1.73)	0.988	0.998	0.88 (0.49-1.56)	0.656	0.975
HAVCR1	1.30 (0.98-1.72)	0.070	0.494	1.17 (0.70-1.96)	0.539	0.954	1.85 (1.17-2.92)	0.009	0.402
HAVCR2	1.58 (0.93-2.68)	0.092	0.559	2.09 (0.81-5.38)	0.128	0.590	1.58 (0.59-4.25)	0.362	0.939
HBEGF	0.88 (0.65-1.20)	0.430	0.904	0.83 (0.47-1.47)	0.525	0.948	0.70 (0.39-1.24)	0.218	0.869
HBQ1	1.37 (0.98-1.91)	0.064	0.473	1.02 (0.55-1.90)	0.957	0.997	1.86 (1.07-3.23)	0.027	0.588
HBZ	0.96 (0.76-1.23)	0.764	0.987	0.93 (0.60-1.45)	0.753	0.997	1.12 (0.77-1.65)	0.554	0.955
HCG22	1.41 (0.95-2.10)	0.088	0.545	1.99 (1.12-3.53)	0.019	0.246	0.64 (0.18-2.25)	0.486	0.944
HCLS1	0.85 (0.57-1.28)	0.434	0.906	1.10 (0.51-2.37)	0.803	0.997	0.57 (0.26-1.23)	0.149	0.813
HDAC8	1.04 (0.67-1.61)	0.852	0.998	1.00 (0.43-2.29)	0.991	0.998	0.88 (0.33-2.34)	0.803	0.991
HDAC9	0.90 (0.57-1.40)	0.631	0.967	0.61 (0.22-1.66)	0.334	0.839	0.88 (0.37-2.10)	0.768	0.987
HDDC2	1.00 (0.61-1.64)	0.987	0.999	1.16 (0.50-2.71)	0.725	0.995	1.37 (0.60-3.12)	0.456	0.942
HDGF	1.14 (0.90-1.45)	0.289	0.809	0.88 (0.56-1.37)	0.564	0.962	1.12 (0.72-1.75)	0.608	0.966
HDGFL2	1.06 (0.72-1.57)	0.766	0.987	1.43 (0.74-2.74)	0.288	0.800	0.80 (0.36-1.77)	0.579	0.964
HEBP1	1.29 (0.91-1.82)	0.150	0.673	1.20 (0.61-2.37)	0.595	0.972	1.39 (0.78-2.51)	0.267	0.910
HEG1	2.03 (0.86-4.79)	0.106	0.587	5.75 (1.27-25.97)	0.023	0.267	0.55 (0.11-2.71)	0.464	0.942
HEPACAM2	1.13 (0.61-2.10)	0.697	0.975	1.55 (0.53-4.58)	0.427	0.907	0.67 (0.23-1.95)	0.459	0.942
HEPH	2.27 (0.92-5.61)	0.077	0.514	8.02 (1.49-43.09)	0.015	0.230	0.85 (0.19-3.77)	0.827	0.991
HEXIM1	1.18 (0.89-1.56)	0.238	0.768	1.09 (0.65-1.81)	0.755	0.997	1.28 (0.75-2.19)	0.369	0.939
HGF	1.21 (0.69-2.12)	0.495	0.938	1.04 (0.36-3.03)	0.937	0.997	1.05 (0.36-3.09)	0.926	0.992
HGFAC	1.05 (0.45-2.48)	0.907	0.999	0.33 (0.07-1.52)	0.154	0.627	0.66 (0.15-2.97)	0.591	0.965
HGS	0.91 (0.67-1.24)	0.556	0.951	0.70 (0.40-1.23)	0.215	0.723	1.07 (0.62-1.86)	0.804	0.991
HHEX	1.07 (0.77-1.49)	0.689	0.975	1.18 (0.64-2.16)	0.591	0.970	0.91 (0.49-1.70)	0.767	0.987
HIF1A	0.98 (0.64-1.50)	0.928	0.999	1.53 (0.97-2.42)	0.067	0.441	1.19 (0.63-2.24)	0.590	0.965
HIP1	1.10 (0.54-2.26)	0.794	0.994	1.46 (0.53-4.02)	0.463	0.926	1.17 (0.36-3.78)	0.795	0.989

HIP1R	1.50 (0.93-2.42)	0.094	0.562	1.36 (0.61-3.00)	0.452	0.920	2.48 (1.31-4.69)	0.005	0.343
HJV	1.55 (0.85-2.83)	0.151	0.673	1.74 (0.64-4.76)	0.281	0.793	3.27 (1.30-8.18)	0.012	0.433
HK2	0.96 (0.40-2.32)	0.933	0.999	1.36 (0.27-6.93)	0.712	0.995	0.41 (0.08-2.07)	0.283	0.914
HLA_A	1.53 (0.75-3.10)	0.241	0.769	4.40 (1.30-14.91)	0.017	0.236	1.56 (0.45-5.41)	0.482	0.944
HLA_DRA	1.07 (0.58-1.96)	0.831	0.997	0.80 (0.27-2.38)	0.682	0.993	1.18 (0.42-3.31)	0.756	0.985
HLA_E	1.43 (0.78-2.61)	0.249	0.774	2.13 (0.81-5.56)	0.124	0.581	1.15 (0.36-3.65)	0.810	0.991
HMBS	1.25 (0.77-2.02)	0.371	0.872	0.62 (0.26-1.47)	0.277	0.788	1.29 (0.52-3.20)	0.582	0.964
HMCN2	1.35 (0.83-2.17)	0.225	0.757	2.96 (1.17-7.50)	0.022	0.264	1.84 (0.75-4.49)	0.180	0.853
HMGCL	0.74 (0.45-1.21)	0.230	0.762	1.00 (0.42-2.37)	0.998	0.999	0.41 (0.15-1.09)	0.073	0.718
HMGCS1	1.19 (0.91-1.56)	0.209	0.740	0.80 (0.40-1.63)	0.545	0.959	1.36 (0.88-2.08)	0.162	0.839
HMMR	1.05 (0.72-1.54)	0.785	0.994	1.39 (0.76-2.55)	0.290	0.801	0.65 (0.26-1.64)	0.358	0.939
HMOX1	0.80 (0.50-1.26)	0.329	0.846	0.93 (0.41-2.12)	0.863	0.997	0.98 (0.42-2.27)	0.961	0.994
HMOX2	1.18 (0.74-1.88)	0.481	0.931	1.55 (0.78-3.06)	0.211	0.717	1.25 (0.50-3.10)	0.628	0.970
HNF1A	1.19 (0.93-1.53)	0.159	0.679	1.11 (0.68-1.80)	0.687	0.993	1.11 (0.71-1.74)	0.654	0.975
HNMT	0.99 (0.64-1.53)	0.969	0.999	1.15 (0.53-2.50)	0.726	0.996	1.76 (0.83-3.76)	0.141	0.808
HNRNPK	0.98 (0.73-1.31)	0.893	0.999	1.26 (0.76-2.10)	0.375	0.879	0.96 (0.55-1.67)	0.877	0.992
HNRNPUL1	0.87 (0.51-1.47)	0.591	0.961	1.40 (0.68-2.88)	0.358	0.862	0.93 (0.38-2.29)	0.874	0.992
HPCAL1	0.87 (0.64-1.19)	0.393	0.888	0.77 (0.45-1.34)	0.358	0.862	0.67 (0.38-1.17)	0.161	0.839
HPGDS	0.66 (0.37-1.18)	0.161	0.680	1.47 (0.65-3.36)	0.355	0.862	0.48 (0.16-1.45)	0.191	0.863
HPSE	0.95 (0.74-1.22)	0.693	0.975	1.36 (0.86-2.15)	0.189	0.682	0.73 (0.45-1.17)	0.190	0.862
HRAS	0.99 (0.76-1.29)	0.957	0.999	0.94 (0.55-1.62)	0.828	0.997	1.07 (0.71-1.60)	0.750	0.985
HRC	1.09 (0.66-1.80)	0.737	0.981	1.05 (0.40-2.71)	0.924	0.997	0.83 (0.28-2.41)	0.729	0.978
HRG	0.79 (0.36-1.71)	0.545	0.948	1.86 (0.44-7.84)	0.399	0.900	0.89 (0.21-3.72)	0.877	0.992
HS1BP3	0.91 (0.67-1.24)	0.542	0.948	1.18 (0.69-2.03)	0.541	0.956	0.79 (0.44-1.43)	0.435	0.942
HS3ST3B1	1.71 (0.93-3.15)	0.085	0.535	2.11 (0.70-6.43)	0.187	0.679	2.35 (0.81-6.82)	0.117	0.780

HS6ST1	0.81 (0.43-1.56)	0.534	0.948	0.78 (0.24-2.55)	0.684	0.993	1.26 (0.44-3.64)	0.670	0.975
HS6ST2	1.51 (0.80-2.88)	0.207	0.740	1.72 (0.63-4.68)	0.288	0.800	2.18 (0.84-5.67)	0.108	0.763
HSBP1	1.08 (0.81-1.43)	0.616	0.966	1.21 (0.72-2.05)	0.466	0.926	1.35 (0.79-2.33)	0.274	0.910
HSD11B1	1.38 (0.82-2.31)	0.223	0.757	2.66 (1.06-6.71)	0.038	0.334	0.92 (0.37-2.30)	0.860	0.992
HSD17B14	1.16 (0.80-1.67)	0.433	0.906	0.72 (0.36-1.43)	0.347	0.854	1.71 (0.95-3.08)	0.075	0.718
HSD17B3	1.52 (0.93-2.47)	0.096	0.568	1.49 (0.60-3.74)	0.391	0.893	1.92 (0.86-4.27)	0.109	0.763
HSDL2	0.80 (0.47-1.38)	0.422	0.899	1.07 (0.42-2.75)	0.888	0.997	0.84 (0.31-2.25)	0.724	0.978
HSP90B1	1.12 (0.87-1.45)	0.377	0.875	0.79 (0.39-1.61)	0.517	0.945	1.15 (0.71-1.86)	0.573	0.960
HSPA1A	0.99 (0.71-1.39)	0.972	0.999	0.90 (0.49-1.67)	0.743	0.997	1.06 (0.57-1.97)	0.863	0.992
HSPA2	1.68 (0.96-2.93)	0.069	0.490	2.32 (0.98-5.52)	0.057	0.403	1.93 (0.70-5.32)	0.204	0.867
HSPB1	0.92 (0.66-1.28)	0.611	0.965	0.84 (0.46-1.54)	0.571	0.964	0.83 (0.45-1.54)	0.559	0.955
HSPB6	1.22 (0.74-2.00)	0.444	0.913	1.87 (0.81-4.27)	0.141	0.604	1.63 (0.71-3.75)	0.252	0.901
HSPG2	1.93 (1.06-3.51)	0.032	0.377	3.74 (1.55-9.02)	0.003	0.117	1.91 (0.70-5.21)	0.209	0.867
HTR1A	1.19 (0.88-1.60)	0.267	0.796	0.76 (0.38-1.55)	0.450	0.920	1.48 (0.98-2.22)	0.061	0.698
HTR1B	1.63 (0.99-2.66)	0.053	0.445	1.28 (0.49-3.38)	0.619	0.979	1.68 (0.71-3.96)	0.235	0.885
HTRA2	0.96 (0.66-1.38)	0.824	0.995	1.06 (0.55-2.06)	0.864	0.997	0.67 (0.32-1.39)	0.283	0.914
HYAL1	1.14 (0.46-2.80)	0.780	0.992	1.95 (0.44-8.58)	0.377	0.880	0.49 (0.08-2.85)	0.423	0.942
HYOU1	2.05 (0.86-4.93)	0.107	0.592	2.17 (0.42-11.27)	0.357	0.862	1.22 (0.24-6.16)	0.806	0.991
ICA1	1.04 (0.78-1.39)	0.780	0.992	1.11 (0.65-1.88)	0.712	0.995	0.94 (0.53-1.65)	0.822	0.991
ICAM1	1.64 (0.84-3.18)	0.145	0.667	1.73 (0.54-5.51)	0.357	0.862	1.53 (0.47-5.01)	0.483	0.944
ICAM2	1.60 (0.84-3.04)	0.154	0.676	1.71 (0.55-5.36)	0.357	0.862	1.21 (0.35-4.13)	0.764	0.987
ICAM3	2.20 (1.07-4.51)	0.032	0.377	3.54 (1.12-11.19)	0.032	0.314	0.73 (0.16-3.31)	0.687	0.975
ICAM4	1.01 (0.66-1.55)	0.951	0.999	0.72 (0.33-1.55)	0.403	0.902	0.74 (0.35-1.58)	0.432	0.942
ICAM5	2.24 (1.44-3.50)	<0.001	0.046	1.88 (0.82-4.30)	0.136	0.600	1.36 (0.56-3.34)	0.496	0.947
ICOSLG	1.32 (0.44-4.01)	0.620	0.966	1.91 (0.27-13.50)	0.517	0.945	0.52 (0.06-4.17)	0.534	0.952

ID4	1.03 (0.59-1.78)	0.927	0.999	1.03 (0.36-2.95)	0.957	0.997	1.30 (0.48-3.49)	0.607	0.966
IDI2	1.01 (0.65-1.59)	0.950	0.999	1.41 (0.67-2.96)	0.359	0.862	0.87 (0.39-1.92)	0.722	0.977
IDO1	1.14 (0.79-1.65)	0.469	0.924	1.80 (1.04-3.11)	0.036	0.326	0.96 (0.49-1.88)	0.895	0.992
IDS	0.29 (0.05-1.80)	0.184	0.708	5.56 (0.05-618.61)	0.475	0.932	0.07 (0.01-0.51)	0.009	0.402
IDUA	1.18 (0.74-1.90)	0.484	0.933	1.34 (0.56-3.20)	0.515	0.945	0.76 (0.34-1.72)	0.515	0.947
IFI30	0.93 (0.51-1.71)	0.813	0.995	1.02 (0.35-2.98)	0.971	0.997	1.17 (0.43-3.16)	0.762	0.987
IFIT1	1.22 (0.81-1.83)	0.348	0.855	1.40 (0.72-2.72)	0.325	0.830	1.39 (0.60-3.19)	0.443	0.942
IFIT3	1.49 (0.72-3.08)	0.283	0.806	2.19 (0.61-7.90)	0.231	0.742	0.31 (0.07-1.47)	0.140	0.808
IFNAR1	1.54 (0.70-3.39)	0.281	0.806	3.09 (1.00-9.58)	0.051	0.391	1.21 (0.27-5.47)	0.807	0.991
IFNG	1.00 (0.83-1.21)	0.964	0.999	1.14 (0.80-1.62)	0.468	0.926	1.06 (0.74-1.54)	0.740	0.983
IFNGR1	1.97 (0.77-5.04)	0.155	0.676	5.62 (1.30-24.26)	0.021	0.257	1.55 (0.28-8.52)	0.612	0.966
IFNGR2	1.44 (0.99-2.10)	0.055	0.457	1.51 (0.77-2.95)	0.227	0.738	1.64 (0.86-3.15)	0.135	0.796
IFNL1	0.88 (0.55-1.40)	0.590	0.961	0.83 (0.35-1.98)	0.671	0.993	1.74 (1.01-3.01)	0.045	0.643
IFNL2	1.00 (0.85-1.18)	0.987	0.999	0.97 (0.70-1.36)	0.866	0.997	0.85 (0.55-1.31)	0.458	0.942
IFNLR1	1.24 (0.69-2.23)	0.466	0.922	1.64 (0.74-3.60)	0.220	0.729	1.25 (0.42-3.70)	0.689	0.975
IFNW1	0.77 (0.35-1.70)	0.523	0.948	1.52 (0.85-2.71)	0.154	0.627	0.27 (0.04-1.84)	0.181	0.853
IFT20	0.99 (0.53-1.87)	0.983	0.999	1.22 (0.39-3.88)	0.733	0.997	0.99 (0.28-3.46)	0.983	0.996
IGBP1	0.78 (0.52-1.16)	0.220	0.751	1.22 (0.60-2.45)	0.582	0.967	0.54 (0.25-1.16)	0.115	0.779
IGDCC3	0.82 (0.53-1.26)	0.362	0.865	1.03 (0.52-2.04)	0.926	0.997	1.08 (0.56-2.07)	0.820	0.991
IGDCC4	1.10 (0.46-2.64)	0.824	0.995	4.90 (1.42-16.85)	0.012	0.203	0.66 (0.15-2.98)	0.592	0.965
IGF1R	1.98 (0.79-4.97)	0.146	0.669	2.64 (0.53-13.09)	0.234	0.742	1.05 (0.18-6.32)	0.955	0.992
IGF2BP3	1.55 (0.89-2.71)	0.123	0.620	1.85 (0.77-4.42)	0.168	0.649	1.28 (0.37-4.43)	0.697	0.975
IGF2R	1.77 (0.70-4.49)	0.227	0.757	1.63 (0.30-8.77)	0.570	0.963	3.40 (0.64-18.00)	0.150	0.813
IGFBP1	1.07 (0.90-1.27)	0.436	0.907	0.93 (0.68-1.27)	0.645	0.991	1.18 (0.86-1.61)	0.309	0.923
IGFBP2	1.63 (1.15-2.30)	0.006	0.184	1.37 (0.72-2.61)	0.331	0.836	1.39 (0.74-2.61)	0.307	0.923

IGFBP3	0.64 (0.37-1.10)	0.105	0.584	0.57 (0.21-1.54)	0.271	0.781	0.81 (0.30-2.20)	0.686	0.975
IGFBP4	1.67 (1.21-2.32)	0.002	0.107	1.91 (1.12-3.24)	0.017	0.236	1.65 (0.94-2.92)	0.082	0.743
IGFBP6	1.24 (0.68-2.24)	0.485	0.933	2.34 (0.93-5.90)	0.071	0.456	1.01 (0.34-2.97)	0.985	0.996
IGFBP7	2.16 (1.17-3.99)	0.014	0.286	4.00 (1.38-11.58)	0.011	0.195	3.17 (1.13-8.89)	0.029	0.588
IGFBPL1	1.80 (1.08-3.00)	0.025	0.352	2.55 (0.99-6.55)	0.052	0.392	1.52 (0.58-4.04)	0.396	0.942
IGFL4	0.88 (0.60-1.30)	0.523	0.948	0.97 (0.49-1.91)	0.926	0.997	0.99 (0.51-1.92)	0.978	0.994
IGHMBP2	0.90 (0.54-1.50)	0.689	0.975	1.81 (0.95-3.45)	0.070	0.453	0.30 (0.09-1.00)	0.049	0.670
IGLC2	1.97 (0.89-4.38)	0.095	0.568	3.54 (1.18-10.58)	0.024	0.271	2.81 (0.87-9.05)	0.083	0.743
IGLON5	1.08 (0.82-1.42)	0.596	0.962	1.02 (0.60-1.74)	0.946	0.997	0.91 (0.48-1.75)	0.787	0.989
IGSF21	0.55 (0.26-1.16)	0.118	0.609	0.80 (0.22-2.88)	0.730	0.997	0.49 (0.12-1.95)	0.312	0.923
IGSF3	1.93 (1.18-3.15)	0.009	0.220	2.77 (1.26-6.12)	0.012	0.203	2.32 (1.05-5.16)	0.038	0.623
IGSF8	2.20 (1.14-4.24)	0.018	0.308	2.57 (0.80-8.30)	0.114	0.559	0.87 (0.22-3.45)	0.840	0.992
IGSF9	1.01 (0.81-1.26)	0.899	0.999	1.03 (0.69-1.54)	0.887	0.997	1.21 (0.82-1.79)	0.332	0.934
IKBKG	1.14 (0.89-1.46)	0.300	0.818	1.07 (0.68-1.69)	0.764	0.997	1.24 (0.77-1.99)	0.377	0.942
IKZF2	0.98 (0.77-1.25)	0.865	0.998	0.84 (0.50-1.42)	0.517	0.945	0.99 (0.64-1.53)	0.968	0.994
IL10	1.20 (0.92-1.55)	0.177	0.706	1.00 (0.60-1.70)	0.985	0.998	1.24 (0.76-2.02)	0.384	0.942
IL10RA	1.29 (0.92-1.80)	0.141	0.657	1.79 (1.17-2.75)	0.008	0.171	0.95 (0.42-2.18)	0.912	0.992
IL10RB	1.58 (0.79-3.15)	0.198	0.724	3.59 (1.18-10.88)	0.024	0.271	1.68 (0.47-5.99)	0.422	0.942
IL11	1.07 (0.68-1.67)	0.781	0.992	1.42 (0.71-2.86)	0.321	0.824	0.54 (0.19-1.53)	0.246	0.897
IL12A_IL12B	1.14 (0.88-1.49)	0.326	0.843	1.43 (0.90-2.27)	0.132	0.596	1.04 (0.64-1.70)	0.872	0.992
IL12B	1.20 (0.82-1.77)	0.346	0.855	1.80 (0.88-3.69)	0.106	0.536	1.16 (0.58-2.33)	0.669	0.975
IL12RB1	1.11 (0.60-2.06)	0.741	0.983	1.63 (0.54-4.90)	0.386	0.889	1.17 (0.39-3.47)	0.781	0.987
IL12RB2	1.01 (0.55-1.84)	0.981	0.999	0.96 (0.31-2.92)	0.937	0.997	0.74 (0.22-2.46)	0.620	0.967
IL13	0.96 (0.64-1.45)	0.854	0.998	0.87 (0.35-2.14)	0.758	0.997	1.06 (0.56-2.00)	0.856	0.992
IL13RA1	1.02 (0.39-2.69)	0.961	0.999	2.38 (0.39-14.54)	0.347	0.854	0.67 (0.12-3.71)	0.649	0.973

IL13RA2	1.00 (0.37-2.67)	0.996	0.999	1.94 (0.30-12.39)	0.482	0.934	0.87 (0.13-5.64)	0.884	0.992
IL15	1.60 (0.84-3.05)	0.156	0.676	1.75 (0.53-5.76)	0.354	0.862	2.29 (0.70-7.55)	0.173	0.853
IL15RA	0.78 (0.42-1.45)	0.429	0.904	3.50 (1.25-9.77)	0.017	0.236	0.66 (0.22-1.99)	0.463	0.942
IL16	1.02 (0.60-1.75)	0.944	0.999	0.92 (0.33-2.54)	0.869	0.997	1.04 (0.40-2.69)	0.942	0.992
IL17A	1.14 (0.70-1.86)	0.602	0.962	0.98 (0.37-2.57)	0.964	0.997	0.94 (0.36-2.47)	0.899	0.992
IL17C	1.39 (1.05-1.83)	0.022	0.338	1.28 (0.75-2.17)	0.360	0.862	1.24 (0.74-2.07)	0.416	0.942
IL17D	1.70 (1.01-2.86)	0.047	0.428	1.76 (0.73-4.22)	0.204	0.711	1.47 (0.59-3.69)	0.409	0.942
IL17F	1.15 (0.88-1.49)	0.302	0.820	1.46 (1.00-2.13)	0.051	0.391	0.66 (0.31-1.39)	0.273	0.910
IL17RA	1.06 (0.64-1.76)	0.826	0.995	1.01 (0.40-2.53)	0.990	0.998	1.28 (0.50-3.27)	0.605	0.966
IL17RB	1.17 (0.76-1.78)	0.479	0.929	0.88 (0.40-1.94)	0.749	0.997	0.79 (0.36-1.75)	0.561	0.956
IL18	0.67 (0.40-1.14)	0.143	0.661	0.79 (0.30-2.10)	0.642	0.990	0.79 (0.31-2.01)	0.624	0.967
IL18BP	1.21 (0.63-2.33)	0.567	0.956	2.34 (0.79-6.97)	0.125	0.585	0.66 (0.19-2.34)	0.522	0.948
IL18R1	2.95 (1.62-5.36)	<0.001	0.046	2.59 (0.88-7.62)	0.085	0.486	3.12 (1.03-9.42)	0.043	0.637
IL18RAP	0.93 (0.81-1.08)	0.347	0.855	0.91 (0.70-1.19)	0.499	0.941	0.72 (0.51-1.01)	0.055	0.698
IL19	1.14 (0.85-1.52)	0.399	0.894	1.81 (1.12-2.91)	0.015	0.230	0.79 (0.44-1.40)	0.417	0.942
IL1A	0.72 (0.45-1.16)	0.177	0.706	0.49 (0.19-1.31)	0.156	0.633	0.48 (0.19-1.25)	0.135	0.796
IL1B	1.05 (0.72-1.53)	0.791	0.994	1.68 (0.99-2.86)	0.057	0.403	0.47 (0.19-1.16)	0.102	0.763
IL1R1	3.29 (1.41-7.70)	0.006	0.184	11.63 (2.50-54.09)	0.002	0.084	3.91 (0.86-17.81)	0.078	0.730
IL1R2	1.89 (0.64-5.53)	0.249	0.774	3.65 (0.52-25.60)	0.193	0.691	3.46 (0.48-24.98)	0.218	0.869
IL1RAP	0.99 (0.61-1.61)	0.979	0.999	0.57 (0.21-1.53)	0.264	0.773	1.04 (0.43-2.53)	0.931	0.992
IL1RL1	1.44 (0.92-2.25)	0.115	0.607	1.93 (0.89-4.23)	0.098	0.521	2.02 (0.84-4.86)	0.116	0.780
IL1RL2	1.20 (0.67-2.13)	0.535	0.948	0.57 (0.20-1.61)	0.288	0.800	1.40 (0.49-3.96)	0.526	0.949
IL1RN	1.16 (0.81-1.65)	0.417	0.896	0.91 (0.46-1.80)	0.793	0.997	0.89 (0.43-1.83)	0.742	0.983
IL2	0.94 (0.46-1.94)	0.869	0.998	0.76 (0.19-2.98)	0.696	0.994	0.33 (0.08-1.27)	0.106	0.763
IL20	1.17 (0.79-1.75)	0.434	0.906	1.58 (0.87-2.87)	0.132	0.596	0.96 (0.46-2.02)	0.917	0.992

IL20RA	0.91 (0.51-1.63)	0.756	0.986	0.72 (0.23-2.27)	0.576	0.965	1.13 (0.43-2.98)	0.798	0.990
IL20RB	1.14 (0.63-2.05)	0.665	0.971	0.35 (0.11-1.13)	0.079	0.477	1.53 (0.57-4.09)	0.399	0.942
IL21R	1.03 (0.69-1.55)	0.868	0.998	1.99 (1.28-3.08)	0.002	0.098	0.62 (0.29-1.31)	0.208	0.867
IL22	1.32 (0.93-1.86)	0.118	0.609	1.41 (0.77-2.58)	0.266	0.775	0.96 (0.51-1.79)	0.889	0.992
IL22RA1	1.06 (0.62-1.81)	0.824	0.995	1.20 (0.45-3.17)	0.720	0.995	0.97 (0.38-2.48)	0.949	0.992
IL24	1.28 (0.98-1.68)	0.074	0.512	1.24 (0.77-2.01)	0.376	0.880	0.85 (0.42-1.71)	0.648	0.973
IL25	1.29 (0.68-2.45)	0.429	0.904	1.83 (0.71-4.71)	0.214	0.721	0.88 (0.24-3.26)	0.848	0.992
IL2RA	1.42 (0.85-2.35)	0.176	0.706	2.90 (1.26-6.71)	0.013	0.211	0.85 (0.31-2.31)	0.748	0.985
IL2RB	1.32 (0.88-1.99)	0.183	0.708	1.31 (0.60-2.87)	0.495	0.940	1.60 (0.85-3.02)	0.145	0.808
IL2RG	1.13 (0.82-1.56)	0.469	0.924	1.02 (0.53-1.98)	0.942	0.997	1.05 (0.53-2.07)	0.886	0.992
IL3	1.09 (0.73-1.64)	0.663	0.971	2.32 (1.06-5.08)	0.036	0.326	1.13 (0.55-2.30)	0.742	0.983
IL31	0.87 (0.53-1.42)	0.577	0.956	1.04 (0.46-2.37)	0.920	0.997	0.46 (0.14-1.51)	0.201	0.867
IL31RA	1.22 (0.66-2.27)	0.527	0.948	1.55 (0.51-4.72)	0.444	0.914	0.84 (0.25-2.89)	0.787	0.989
IL32	1.13 (0.69-1.84)	0.629	0.966	2.17 (1.06-4.45)	0.034	0.318	0.90 (0.35-2.30)	0.822	0.991
IL33	1.31 (0.74-2.33)	0.355	0.859	1.92 (0.67-5.50)	0.223	0.735	0.62 (0.22-1.74)	0.365	0.939
IL34	1.45 (0.78-2.72)	0.241	0.769	1.16 (0.39-3.48)	0.793	0.997	1.78 (0.58-5.50)	0.313	0.923
IL36A	1.41 (0.93-2.15)	0.109	0.596	2.13 (1.11-4.08)	0.023	0.264	0.85 (0.32-2.26)	0.749	0.985
IL36G	1.29 (0.94-1.77)	0.116	0.607	1.74 (1.12-2.70)	0.013	0.213	0.57 (0.26-1.24)	0.158	0.826
IL3RA	1.32 (0.69-2.51)	0.400	0.894	3.30 (1.20-9.08)	0.021	0.257	0.81 (0.24-2.77)	0.740	0.983
IL4	0.93 (0.75-1.16)	0.531	0.948	1.18 (0.68-2.04)	0.560	0.962	0.80 (0.62-1.04)	0.100	0.762
IL4R	1.43 (0.87-2.35)	0.163	0.683	2.03 (0.91-4.52)	0.084	0.486	1.54 (0.57-4.18)	0.400	0.942
IL5	0.91 (0.75-1.09)	0.296	0.814	1.07 (0.82-1.40)	0.596	0.973	0.55 (0.36-0.84)	0.006	0.358
IL5RA	1.00 (0.66-1.50)	0.982	0.999	0.98 (0.47-2.05)	0.954	0.997	0.74 (0.36-1.55)	0.428	0.942
IL6	1.20 (0.91-1.57)	0.192	0.718	1.16 (0.70-1.90)	0.570	0.963	1.29 (0.80-2.07)	0.291	0.915
IL6R	0.65 (0.33-1.29)	0.221	0.753	0.56 (0.16-1.94)	0.358	0.862	0.43 (0.12-1.52)	0.190	0.862

IL6ST	2.68 (1.14-6.31)	0.024	0.351	6.67 (2.27-19.60)	<0.001	0.059	0.58 (0.07-4.93)	0.620	0.967
IL7	0.79 (0.54-1.15)	0.214	0.746	0.70 (0.35-1.40)	0.316	0.819	0.75 (0.37-1.51)	0.420	0.942
IL7R	1.35 (0.87-2.10)	0.183	0.708	2.06 (0.91-4.66)	0.082	0.484	0.96 (0.44-2.07)	0.913	0.992
IL9	1.01 (0.67-1.52)	0.965	0.999	1.12 (0.58-2.18)	0.740	0.997	1.46 (0.92-2.34)	0.111	0.763
ILKAP	1.00 (0.66-1.50)	0.992	0.999	1.41 (0.77-2.61)	0.266	0.774	0.76 (0.34-1.72)	0.512	0.947
IMMT	0.72 (0.41-1.25)	0.245	0.771	1.03 (0.40-2.68)	0.949	0.997	1.23 (0.52-2.92)	0.640	0.973
IMPA1	0.79 (0.53-1.19)	0.264	0.792	0.88 (0.42-1.83)	0.724	0.995	0.66 (0.30-1.46)	0.309	0.923
IMPACT	1.02 (0.66-1.57)	0.925	0.999	1.48 (0.69-3.16)	0.310	0.816	1.04 (0.48-2.27)	0.920	0.992
IMPG1	0.75 (0.29-1.95)	0.562	0.956	0.97 (0.18-5.21)	0.968	0.997	0.82 (0.15-4.57)	0.820	0.991
ING1	0.97 (0.58-1.64)	0.919	0.999	1.14 (0.46-2.82)	0.769	0.997	1.06 (0.41-2.71)	0.908	0.992
INHBB	1.09 (0.72-1.64)	0.686	0.975	1.44 (0.73-2.83)	0.294	0.803	1.15 (0.56-2.34)	0.703	0.975
INHBC	0.89 (0.52-1.52)	0.658	0.971	0.91 (0.33-2.48)	0.852	0.997	1.19 (0.44-3.24)	0.734	0.981
INPP1	0.70 (0.45-1.09)	0.113	0.602	0.96 (0.44-2.10)	0.922	0.997	0.69 (0.30-1.59)	0.381	0.942
INPP5D	0.98 (0.64-1.52)	0.945	0.999	1.14 (0.54-2.39)	0.731	0.997	1.14 (0.55-2.34)	0.729	0.978
INPP5J	0.64 (0.32-1.30)	0.217	0.746	0.30 (0.08-1.17)	0.084	0.486	0.59 (0.17-2.06)	0.412	0.942
INPPL1	1.21 (0.97-1.51)	0.099	0.573	1.24 (0.82-1.88)	0.298	0.805	1.33 (0.87-2.04)	0.185	0.861
INSL3	0.81 (0.68-0.96)	0.016	0.294	0.85 (0.61-1.18)	0.328	0.832	0.71 (0.53-0.95)	0.022	0.551
INSL4	1.19 (0.92-1.53)	0.180	0.706	1.36 (0.91-2.03)	0.137	0.601	1.20 (0.75-1.91)	0.456	0.942
INSL5	1.06 (0.84-1.32)	0.638	0.968	1.00 (0.66-1.52)	0.995	0.998	1.24 (0.82-1.87)	0.318	0.923
INSR	1.71 (0.42-6.91)	0.453	0.917	4.64 (0.36-59.37)	0.238	0.742	1.18 (0.08-17.16)	0.905	0.992
IPCEF1	1.10 (0.86-1.41)	0.460	0.920	1.19 (0.75-1.88)	0.458	0.923	0.93 (0.58-1.51)	0.772	0.987
IQGAP2	0.78 (0.51-1.19)	0.241	0.769	0.66 (0.30-1.46)	0.309	0.816	0.70 (0.31-1.60)	0.402	0.942
IRAG2	1.03 (0.81-1.33)	0.789	0.994	0.89 (0.54-1.46)	0.634	0.986	1.13 (0.73-1.73)	0.588	0.965
IRAK1	1.18 (0.86-1.63)	0.310	0.826	1.41 (0.79-2.50)	0.246	0.751	1.12 (0.60-2.08)	0.729	0.978
IRAK4	1.08 (0.86-1.34)	0.516	0.946	1.04 (0.70-1.55)	0.835	0.997	1.09 (0.72-1.65)	0.692	0.975

ISLR2	2.02 (1.17-3.49)	0.012	0.262	5.47 (1.96-15.22)	0.001	0.066	1.72 (0.65-4.56)	0.276	0.910
ISM1	1.25 (0.70-2.23)	0.459	0.920	4.49 (1.55-13.00)	0.006	0.146	2.73 (0.93-7.99)	0.068	0.718
ISM2	1.07 (0.59-1.94)	0.821	0.995	2.29 (1.11-4.73)	0.024	0.271	1.29 (0.44-3.77)	0.642	0.973
IST1	0.89 (0.70-1.14)	0.370	0.871	1.22 (0.78-1.92)	0.381	0.886	0.81 (0.51-1.30)	0.386	0.942
ITGA11	0.77 (0.43-1.38)	0.377	0.875	1.99 (0.63-6.26)	0.238	0.742	1.27 (0.42-3.86)	0.668	0.975
ITGA2	1.40 (0.75-2.62)	0.291	0.809	4.55 (1.45-14.28)	0.009	0.188	0.80 (0.25-2.50)	0.695	0.975
ITGA5	1.99 (0.89-4.44)	0.093	0.560	3.12 (0.75-12.95)	0.117	0.564	0.93 (0.20-4.34)	0.930	0.992
ITGA6	0.66 (0.41-1.08)	0.100	0.573	0.88 (0.38-2.05)	0.763	0.997	0.47 (0.18-1.24)	0.126	0.796
ITGAL	0.47 (0.30-0.74)	0.001	0.078	0.55 (0.23-1.32)	0.181	0.667	0.62 (0.24-1.59)	0.317	0.923
ITGAM	1.25 (0.65-2.41)	0.496	0.938	2.56 (0.83-7.91)	0.103	0.528	0.88 (0.27-2.93)	0.836	0.991
ITGAV	0.50 (0.15-1.65)	0.255	0.783	1.44 (0.15-13.59)	0.748	0.997	1.38 (0.16-12.25)	0.773	0.987
ITGAX	0.97 (0.78-1.19)	0.740	0.982	1.05 (0.74-1.48)	0.781	0.997	1.03 (0.73-1.45)	0.880	0.992
ITGB1	1.12 (0.43-2.92)	0.811	0.995	3.16 (0.67-14.95)	0.147	0.617	1.42 (0.25-8.08)	0.691	0.975
ITGB1BP1	0.84 (0.49-1.44)	0.524	0.948	0.42 (0.16-1.11)	0.081	0.483	1.95 (0.74-5.10)	0.175	0.853
ITGB1BP2	0.99 (0.80-1.22)	0.916	0.999	1.08 (0.74-1.59)	0.681	0.993	0.92 (0.62-1.37)	0.683	0.975
ITGB2	1.06 (0.52-2.16)	0.882	0.999	1.18 (0.33-4.17)	0.802	0.997	0.81 (0.22-3.04)	0.753	0.985
ITGB5	1.05 (0.59-1.88)	0.865	0.998	2.08 (0.82-5.29)	0.123	0.581	0.92 (0.29-2.92)	0.888	0.992
ITGB6	1.56 (0.76-3.22)	0.225	0.757	3.62 (0.94-13.88)	0.061	0.421	3.74 (0.98-14.22)	0.053	0.687
ITGB7	1.20 (0.77-1.88)	0.413	0.894	1.94 (0.96-3.93)	0.064	0.428	1.29 (0.55-2.99)	0.559	0.955
ITGBL1	1.05 (0.63-1.75)	0.852	0.998	2.80 (1.31-6.02)	0.008	0.176	0.97 (0.38-2.49)	0.953	0.992
ITIH1	1.10 (0.35-3.47)	0.874	0.998	1.44 (0.23-8.92)	0.698	0.994	0.68 (0.07-6.53)	0.741	0.983
ITIH3	1.39 (0.77-2.52)	0.273	0.799	0.85 (0.29-2.44)	0.760	0.997	1.52 (0.52-4.42)	0.440	0.942
ITIH4	0.59 (0.22-1.57)	0.289	0.809	0.59 (0.10-3.55)	0.567	0.963	0.19 (0.03-1.37)	0.100	0.762
ITIH5	1.14 (0.66-1.98)	0.643	0.971	1.25 (0.44-3.55)	0.678	0.993	1.70 (0.68-4.24)	0.252	0.901
ITM2A	0.95 (0.66-1.38)	0.797	0.994	1.00 (0.51-1.97)	0.993	0.998	0.99 (0.51-1.94)	0.986	0.996

ITPA	1.12 (0.78-1.62)	0.535	0.948	1.81 (0.96-3.41)	0.068	0.447	0.97 (0.49-1.92)	0.924	0.992
ITPR1	0.76 (0.51-1.15)	0.202	0.732	1.05 (0.57-1.91)	0.885	0.997	0.57 (0.25-1.29)	0.178	0.853
ITPRIP	0.88 (0.46-1.69)	0.699	0.975	1.09 (0.36-3.30)	0.872	0.997	0.77 (0.21-2.84)	0.696	0.975
IVD	1.23 (0.77-1.96)	0.389	0.882	1.29 (0.56-2.98)	0.553	0.960	2.33 (1.20-4.51)	0.012	0.433
IZUMO1	1.17 (0.77-1.79)	0.464	0.922	0.79 (0.26-2.39)	0.678	0.993	0.93 (0.32-2.69)	0.900	0.992
JAM2	2.51 (1.45-4.37)	0.001	0.075	3.34 (1.42-7.85)	0.006	0.146	2.24 (0.83-6.03)	0.110	0.763
JAM3	1.12 (0.66-1.89)	0.673	0.972	1.57 (0.66-3.72)	0.306	0.816	1.31 (0.54-3.17)	0.553	0.955
JCHAIN	1.01 (0.70-1.45)	0.953	0.999	1.35 (0.74-2.44)	0.329	0.832	0.44 (0.22-0.88)	0.021	0.548
JMJD1C	0.91 (0.57-1.44)	0.681	0.975	1.11 (0.55-2.23)	0.780	0.997	0.70 (0.27-1.81)	0.465	0.942
JPT2	0.98 (0.75-1.29)	0.902	0.999	1.27 (0.77-2.10)	0.342	0.848	0.89 (0.53-1.49)	0.650	0.975
JUN	1.01 (0.62-1.62)	0.979	0.999	1.01 (0.42-2.43)	0.974	0.997	0.88 (0.32-2.41)	0.797	0.990
KAZALD1	0.89 (0.56-1.42)	0.625	0.966	1.65 (0.66-4.11)	0.282	0.793	0.90 (0.39-2.08)	0.808	0.991
KAZN	1.04 (0.81-1.33)	0.765	0.987	1.01 (0.64-1.59)	0.969	0.997	1.08 (0.68-1.72)	0.752	0.985
KCNC4	0.72 (0.42-1.24)	0.240	0.769	0.69 (0.26-1.84)	0.459	0.923	0.67 (0.23-1.92)	0.452	0.942
KCNH2	1.14 (0.82-1.59)	0.421	0.899	1.29 (0.79-2.12)	0.314	0.818	0.77 (0.33-1.81)	0.556	0.955
KCNIP4	1.08 (0.72-1.63)	0.699	0.975	1.15 (0.57-2.33)	0.689	0.994	1.25 (0.61-2.56)	0.538	0.952
KCTD5	0.89 (0.48-1.65)	0.717	0.975	1.31 (0.49-3.48)	0.588	0.967	0.65 (0.19-2.22)	0.493	0.947
KDM3A	1.01 (0.79-1.29)	0.963	0.999	0.68 (0.35-1.30)	0.242	0.744	1.18 (0.78-1.78)	0.443	0.942
KDR	0.61 (0.29-1.30)	0.198	0.724	0.66 (0.18-2.40)	0.529	0.949	0.20 (0.06-0.64)	0.006	0.359
KEL	0.97 (0.52-1.79)	0.917	0.999	1.42 (0.48-4.22)	0.530	0.949	1.60 (0.53-4.88)	0.407	0.942
KHDC3L	0.95 (0.67-1.34)	0.770	0.988	1.23 (0.75-2.02)	0.417	0.903	0.55 (0.22-1.38)	0.202	0.867
KHK	0.69 (0.48-1.00)	0.052	0.443	1.20 (0.63-2.31)	0.574	0.965	0.55 (0.27-1.13)	0.105	0.763
KIAA0319	2.03 (1.13-3.66)	0.018	0.303	2.08 (0.72-6.05)	0.178	0.662	2.56 (0.91-7.22)	0.075	0.718
KIAA1549	0.82 (0.57-1.18)	0.290	0.809	0.94 (0.48-1.84)	0.866	0.997	0.69 (0.35-1.35)	0.275	0.910
KIAA1549L	1.03 (0.42-2.52)	0.941	0.999	1.08 (0.24-4.82)	0.923	0.997	0.30 (0.06-1.59)	0.156	0.823

KIAA2013	1.07 (0.63-1.81)	0.805	0.995	1.10 (0.42-2.92)	0.845	0.997	1.48 (0.65-3.39)	0.353	0.938
KIF1C	1.57 (0.74-3.35)	0.241	0.769	3.57 (1.44-8.84)	0.006	0.151	2.11 (0.78-5.68)	0.140	0.808
KIF20B	0.98 (0.70-1.36)	0.904	0.999	0.96 (0.52-1.77)	0.892	0.997	0.92 (0.51-1.65)	0.782	0.987
KIF22	1.02 (0.82-1.27)	0.839	0.998	1.04 (0.70-1.54)	0.861	0.997	1.03 (0.69-1.53)	0.887	0.992
KIFBP	1.00 (0.76-1.32)	0.991	0.999	1.04 (0.63-1.71)	0.876	0.997	1.09 (0.64-1.85)	0.751	0.985
KIR2DL2	1.08 (0.81-1.43)	0.603	0.962	1.17 (0.71-1.93)	0.545	0.959	1.09 (0.66-1.81)	0.729	0.978
KIR2DL3	1.06 (0.80-1.40)	0.689	0.975	1.15 (0.70-1.90)	0.581	0.967	0.90 (0.54-1.51)	0.694	0.975
KIR2DS4	1.03 (0.89-1.18)	0.722	0.975	1.12 (0.87-1.44)	0.377	0.880	1.03 (0.79-1.35)	0.815	0.991
KIR3DL1	1.06 (0.89-1.27)	0.528	0.948	0.94 (0.68-1.30)	0.714	0.995	1.19 (0.86-1.65)	0.299	0.917
KIR3DL2	1.30 (1.03-1.64)	0.030	0.372	1.66 (1.24-2.24)	<0.001	0.063	1.39 (0.89-2.16)	0.146	0.808
KIRREL1	1.23 (0.86-1.76)	0.258	0.786	1.26 (0.67-2.39)	0.473	0.932	1.59 (0.94-2.67)	0.081	0.743
KIRREL2	0.91 (0.52-1.58)	0.741	0.983	1.45 (0.54-3.86)	0.458	0.923	0.74 (0.26-2.11)	0.569	0.960
KIT	0.91 (0.44-1.89)	0.809	0.995	1.15 (0.33-4.01)	0.823	0.997	0.17 (0.05-0.61)	0.007	0.366
KITLG	1.00 (0.57-1.76)	0.998	0.999	0.92 (0.33-2.57)	0.870	0.997	0.73 (0.27-1.94)	0.526	0.949
KLB	1.34 (1.05-1.70)	0.018	0.308	1.08 (0.66-1.79)	0.751	0.997	0.99 (0.59-1.66)	0.976	0.994
KLF4	1.52 (0.99-2.35)	0.058	0.463	2.18 (1.23-3.86)	0.007	0.167	1.57 (0.77-3.18)	0.212	0.867
KLHL41	1.08 (0.70-1.66)	0.717	0.975	0.87 (0.36-2.12)	0.756	0.997	1.10 (0.56-2.16)	0.772	0.987
KLK1	1.02 (0.86-1.21)	0.790	0.994	1.12 (0.83-1.51)	0.455	0.923	1.06 (0.78-1.45)	0.699	0.975
KLK10	1.39 (0.85-2.28)	0.194	0.720	2.26 (1.01-5.09)	0.049	0.383	1.26 (0.49-3.21)	0.633	0.973
KLK11	1.26 (0.73-2.18)	0.413	0.894	1.95 (0.99-3.87)	0.055	0.396	0.99 (0.32-3.03)	0.983	0.996
KLK12	0.92 (0.78-1.07)	0.260	0.787	0.92 (0.69-1.22)	0.572	0.964	0.93 (0.70-1.23)	0.604	0.966
KLK13	1.74 (1.09-2.80)	0.022	0.338	2.94 (1.26-6.87)	0.013	0.211	1.16 (0.48-2.78)	0.747	0.985
KLK14	2.09 (1.28-3.39)	0.003	0.129	2.80 (1.20-6.52)	0.017	0.236	1.24 (0.49-3.17)	0.653	0.975
KLK15	1.16 (0.79-1.72)	0.449	0.916	1.02 (0.49-2.12)	0.967	0.997	1.35 (0.66-2.77)	0.410	0.942
KLK3	0.66 (0.53-0.81)	<0.001	0.021	0.83 (0.57-1.21)	0.338	0.842	0.43 (0.29-0.62)	<0.001	0.034

KLK4	1.12 (0.81-1.55)	0.503	0.943	1.12 (0.62-2.00)	0.709	0.995	1.03 (0.56-1.87)	0.932	0.992
KLK6	1.21 (0.62-2.33)	0.577	0.956	1.16 (0.36-3.76)	0.807	0.997	0.96 (0.28-3.31)	0.954	0.992
KLK7	1.02 (0.55-1.88)	0.962	0.999	1.80 (0.71-4.56)	0.213	0.721	0.28 (0.09-0.92)	0.036	0.623
KLK8	1.65 (0.93-2.92)	0.085	0.535	2.57 (1.02-6.49)	0.046	0.372	1.06 (0.37-3.03)	0.916	0.992
KLKB1	0.43 (0.16-1.16)	0.097	0.569	0.72 (0.12-4.22)	0.717	0.995	0.16 (0.03-0.93)	0.042	0.630
KLRB1	1.62 (0.97-2.69)	0.063	0.472	1.36 (0.55-3.35)	0.500	0.941	1.63 (0.64-4.18)	0.306	0.923
KLRC1	1.32 (0.97-1.80)	0.079	0.514	1.21 (0.62-2.36)	0.578	0.966	0.73 (0.30-1.80)	0.499	0.947
KLRD1	1.08 (0.68-1.71)	0.757	0.986	1.83 (0.83-4.05)	0.137	0.600	0.83 (0.35-1.97)	0.669	0.975
KLRF1	1.52 (0.97-2.38)	0.065	0.474	1.74 (0.85-3.60)	0.132	0.596	1.47 (0.64-3.41)	0.364	0.939
KLRK1	1.25 (0.70-2.25)	0.452	0.916	1.82 (0.63-5.24)	0.268	0.777	2.59 (0.85-7.89)	0.095	0.762
KRT14	1.05 (0.47-2.35)	0.900	0.999	2.44 (0.71-8.44)	0.157	0.633	0.26 (0.05-1.28)	0.098	0.762
KRT17	1.04 (0.56-1.94)	0.901	0.999	0.42 (0.11-1.58)	0.200	0.707	0.92 (0.29-2.93)	0.881	0.992
KRT18	0.92 (0.72-1.19)	0.544	0.948	0.97 (0.62-1.51)	0.881	0.997	1.03 (0.66-1.61)	0.897	0.992
KRT19	1.11 (0.82-1.51)	0.487	0.935	0.78 (0.42-1.43)	0.423	0.905	1.28 (0.75-2.19)	0.362	0.939
KRT5	0.64 (0.41-1.02)	0.060	0.466	1.06 (0.49-2.28)	0.888	0.997	0.51 (0.22-1.16)	0.109	0.763
KRT6C	0.99 (0.45-2.17)	0.985	0.999	2.34 (1.04-5.28)	0.041	0.349	0.96 (0.24-3.89)	0.953	0.992
KRT8	0.93 (0.73-1.20)	0.599	0.962	1.03 (0.71-1.51)	0.865	0.997	1.08 (0.74-1.58)	0.678	0.975
KYAT1	0.76 (0.51-1.15)	0.201	0.732	1.10 (0.53-2.29)	0.801	0.997	0.68 (0.31-1.45)	0.315	0.923
KYNU	0.53 (0.32-0.87)	0.012	0.268	0.48 (0.19-1.22)	0.124	0.581	0.65 (0.26-1.62)	0.360	0.939
L1CAM	2.15 (1.11-4.19)	0.024	0.350	3.01 (0.92-9.81)	0.067	0.443	1.26 (0.34-4.72)	0.730	0.978
L3HYPDH	0.84 (0.63-1.13)	0.252	0.777	0.96 (0.56-1.64)	0.876	0.997	0.57 (0.34-0.95)	0.032	0.611
LACRT	1.03 (0.73-1.45)	0.858	0.998	0.86 (0.40-1.82)	0.687	0.993	1.50 (0.92-2.44)	0.101	0.762
LACTB2	1.01 (0.70-1.46)	0.949	0.999	1.24 (0.65-2.39)	0.516	0.945	0.76 (0.38-1.53)	0.441	0.942
LAG3	1.03 (0.59-1.78)	0.927	0.999	2.50 (1.09-5.74)	0.031	0.309	0.75 (0.27-2.11)	0.591	0.965
LAIR1	1.29 (0.82-2.04)	0.273	0.799	1.31 (0.58-2.95)	0.512	0.943	1.42 (0.66-3.05)	0.373	0.940

LAIR2	0.87 (0.71-1.07)	0.198	0.724	1.01 (0.67-1.53)	0.963	0.997	0.92 (0.62-1.37)	0.696	0.975
LAMA1	1.02 (0.58-1.77)	0.954	0.999	1.21 (0.56-2.65)	0.624	0.983	0.38 (0.08-1.76)	0.215	0.868
LAMA4	1.70 (0.94-3.08)	0.078	0.514	3.68 (1.35-10.03)	0.011	0.199	1.57 (0.53-4.64)	0.418	0.942
LAMB1	1.72 (0.79-3.72)	0.170	0.697	1.85 (0.45-7.68)	0.396	0.897	2.95 (0.74-11.82)	0.127	0.796
LAMP1	2.45 (0.81-7.45)	0.113	0.602	18.01 (3.72-87.14)	<0.001	0.047	0.37 (0.04-3.48)	0.381	0.942
LAMP2	1.16 (0.37-3.57)	0.802	0.995	0.50 (0.06-4.00)	0.517	0.945	3.38 (0.46-24.97)	0.233	0.884
LAMP3	1.23 (0.83-1.82)	0.309	0.826	0.93 (0.45-1.96)	0.859	0.997	1.94 (0.98-3.84)	0.059	0.698
LAMTOR5	1.44 (0.77-2.68)	0.256	0.783	0.82 (0.22-3.06)	0.772	0.997	2.10 (0.78-5.66)	0.143	0.808
LAP3	0.69 (0.41-1.16)	0.163	0.683	0.70 (0.26-1.84)	0.466	0.926	0.88 (0.32-2.37)	0.794	0.989
LARP1	1.23 (0.82-1.84)	0.317	0.834	2.15 (1.26-3.67)	0.005	0.146	1.65 (0.90-3.01)	0.104	0.763
LAT	0.91 (0.67-1.23)	0.527	0.948	0.91 (0.52-1.58)	0.729	0.997	0.74 (0.43-1.29)	0.288	0.915
LAT2	1.02 (0.86-1.22)	0.789	0.994	1.01 (0.73-1.39)	0.973	0.997	0.98 (0.69-1.38)	0.895	0.992
LATS1	0.84 (0.61-1.15)	0.286	0.806	1.01 (0.57-1.78)	0.977	0.997	0.66 (0.36-1.24)	0.197	0.867
LAYN	1.55 (0.96-2.51)	0.073	0.509	1.90 (0.88-4.12)	0.104	0.528	2.08 (0.99-4.40)	0.054	0.693
LBP	0.93 (0.64-1.34)	0.686	0.975	0.77 (0.40-1.50)	0.444	0.914	0.85 (0.43-1.67)	0.636	0.973
LBR	1.12 (0.81-1.55)	0.490	0.936	1.07 (0.58-2.00)	0.821	0.997	0.98 (0.51-1.88)	0.948	0.992
LCAT	1.14 (0.35-3.68)	0.831	0.997	1.01 (0.12-8.27)	0.995	0.998	0.77 (0.09-6.39)	0.806	0.991
LCN15	1.01 (0.66-1.53)	0.975	0.999	0.99 (0.46-2.14)	0.986	0.998	1.29 (0.65-2.57)	0.469	0.942
LCN2	1.19 (0.69-2.07)	0.530	0.948	1.90 (0.77-4.68)	0.162	0.639	1.21 (0.47-3.08)	0.696	0.975
LCP1	1.25 (0.75-2.08)	0.390	0.884	2.11 (0.90-4.91)	0.085	0.486	0.94 (0.35-2.53)	0.909	0.992
LDLR	1.35 (0.90-2.04)	0.149	0.672	1.42 (0.68-3.00)	0.354	0.862	1.15 (0.54-2.47)	0.713	0.976
LDLRAP1	0.93 (0.72-1.20)	0.598	0.962	1.20 (0.76-1.90)	0.432	0.908	0.65 (0.39-1.06)	0.085	0.745
LECT2	1.92 (1.40-2.62)	<0.001	0.014	1.85 (1.05-3.25)	0.032	0.314	2.12 (1.23-3.66)	0.007	0.370
LEFTY2	0.93 (0.62-1.40)	0.715	0.975	1.27 (0.61-2.65)	0.526	0.948	0.74 (0.35-1.59)	0.446	0.942
LEG1	1.22 (0.90-1.66)	0.199	0.725	1.30 (0.75-2.27)	0.348	0.854	1.61 (0.99-2.64)	0.056	0.698

LELP1	1.02 (0.74-1.40)	0.894	0.999	1.22 (0.77-1.94)	0.390	0.893	0.83 (0.43-1.61)	0.581	0.964
LEO1	1.15 (0.61-2.14)	0.671	0.971	1.45 (0.71-2.93)	0.305	0.816	1.81 (0.83-3.93)	0.133	0.796
LEP	1.47 (1.05-2.05)	0.025	0.352	1.80 (0.96-3.37)	0.068	0.447	1.58 (0.85-2.95)	0.149	0.813
LEPR	2.02 (1.02-4.02)	0.045	0.426	3.34 (0.98-11.41)	0.054	0.393	1.22 (0.35-4.21)	0.756	0.985
LETM1	0.93 (0.58-1.48)	0.759	0.987	1.22 (0.58-2.56)	0.593	0.970	0.62 (0.24-1.63)	0.332	0.934
LGALS1	1.05 (0.56-1.97)	0.873	0.998	2.34 (0.82-6.63)	0.111	0.550	0.60 (0.19-1.92)	0.386	0.942
LGALS3	2.02 (1.01-4.04)	0.047	0.428	1.40 (0.39-5.03)	0.603	0.974	2.39 (0.71-8.07)	0.162	0.839
LGALS3BP	1.47 (0.79-2.72)	0.219	0.748	1.64 (0.55-4.89)	0.371	0.875	2.03 (0.71-5.77)	0.185	0.861
LGALS4	1.56 (1.09-2.25)	0.016	0.294	1.35 (0.69-2.66)	0.384	0.888	2.19 (1.16-4.13)	0.015	0.480
LGALS7_LGALS7B	1.01 (0.62-1.62)	0.984	0.999	1.46 (0.66-3.21)	0.345	0.853	1.22 (0.53-2.78)	0.643	0.973
LGALS8	0.98 (0.67-1.44)	0.918	0.999	1.08 (0.53-2.19)	0.831	0.997	0.74 (0.34-1.61)	0.446	0.942
LGALS9	1.42 (0.74-2.72)	0.297	0.815	2.52 (0.80-7.93)	0.114	0.560	1.48 (0.46-4.79)	0.509	0.947
LGMN	1.25 (0.67-2.34)	0.483	0.933	1.10 (0.35-3.43)	0.866	0.997	1.26 (0.40-3.95)	0.695	0.975
LHB	0.73 (0.52-1.04)	0.079	0.514	0.76 (0.41-1.42)	0.384	0.888	0.74 (0.39-1.39)	0.350	0.938
LHPP	1.10 (0.78-1.54)	0.587	0.960	0.90 (0.49-1.65)	0.737	0.997	1.32 (0.74-2.37)	0.350	0.938
LIF	1.09 (0.68-1.76)	0.722	0.975	1.18 (0.50-2.78)	0.698	0.994	1.70 (0.89-3.23)	0.108	0.763
LIFR	2.62 (1.26-5.42)	0.010	0.232	3.26 (1.01-10.50)	0.047	0.375	2.69 (0.73-9.90)	0.136	0.796
LILRA2	1.02 (0.56-1.85)	0.950	0.999	1.37 (0.44-4.23)	0.588	0.967	0.91 (0.31-2.68)	0.861	0.992
LILRA3	1.27 (0.92-1.75)	0.140	0.652	1.33 (0.72-2.47)	0.359	0.862	1.30 (0.70-2.40)	0.411	0.942
LILRA4	0.70 (0.41-1.22)	0.208	0.740	0.95 (0.39-2.35)	0.918	0.997	0.66 (0.23-1.93)	0.449	0.942
LILRA5	2.49 (1.25-4.96)	0.009	0.230	3.57 (1.05-12.10)	0.041	0.350	0.96 (0.26-3.56)	0.954	0.992
LILRA6	0.94 (0.68-1.29)	0.690	0.975	0.86 (0.48-1.53)	0.604	0.974	1.28 (0.70-2.36)	0.424	0.942
LILRB1	1.98 (0.87-4.46)	0.102	0.576	2.00 (0.47-8.55)	0.349	0.857	1.86 (0.40-8.68)	0.432	0.942
LILRB2	1.00 (0.52-1.94)	0.998	0.999	2.26 (0.64-7.93)	0.203	0.710	0.61 (0.18-2.00)	0.411	0.942
LILRB4	1.25 (0.78-1.98)	0.351	0.856	2.16 (0.99-4.68)	0.053	0.392	0.89 (0.38-2.10)	0.794	0.989

LILRB5	1.09 (0.79-1.50)	0.599	0.962	1.62 (0.82-3.22)	0.166	0.649	0.71 (0.43-1.18)	0.190	0.862
LIPF	0.92 (0.66-1.28)	0.616	0.966	1.20 (0.66-2.17)	0.547	0.960	1.05 (0.58-1.90)	0.868	0.992
LMNB1	0.99 (0.67-1.46)	0.956	0.999	1.44 (0.78-2.69)	0.246	0.751	0.94 (0.46-1.92)	0.873	0.992
LMNB2	2.08 (1.04-4.17)	0.039	0.413	3.31 (1.10-9.97)	0.033	0.318	3.09 (1.02-9.38)	0.046	0.643
LMOD1	1.83 (1.21-2.75)	0.004	0.142	2.06 (1.05-4.04)	0.036	0.326	2.20 (1.20-4.04)	0.011	0.429
LMOD2	1.13 (0.71-1.79)	0.603	0.962	1.47 (0.75-2.88)	0.262	0.772	0.69 (0.25-1.88)	0.467	0.942
LONP1	1.00 (0.75-1.34)	0.989	0.999	0.85 (0.50-1.46)	0.556	0.962	0.85 (0.49-1.48)	0.572	0.960
LPA	1.09 (0.94-1.26)	0.237	0.767	1.02 (0.79-1.33)	0.868	0.997	0.97 (0.75-1.26)	0.820	0.991
LPCAT2	1.00 (0.63-1.60)	0.991	0.999	0.95 (0.36-2.51)	0.912	0.997	0.48 (0.12-1.95)	0.307	0.923
LPL	1.17 (0.72-1.88)	0.532	0.948	1.15 (0.47-2.81)	0.757	0.997	1.06 (0.44-2.57)	0.899	0.992
LPO	1.28 (0.92-1.78)	0.141	0.657	1.02 (0.57-1.83)	0.950	0.997	1.39 (0.79-2.46)	0.258	0.907
LPP	0.92 (0.62-1.35)	0.654	0.971	1.24 (0.61-2.49)	0.553	0.960	0.69 (0.34-1.39)	0.297	0.917
LRCH4	0.90 (0.61-1.32)	0.575	0.956	1.16 (0.60-2.24)	0.659	0.992	0.73 (0.34-1.55)	0.414	0.942
LRFN2	1.64 (1.01-2.65)	0.043	0.425	1.25 (0.43-3.66)	0.684	0.993	2.35 (1.00-5.56)	0.051	0.671
LRG1	1.93 (0.75-4.94)	0.170	0.697	1.43 (0.26-7.97)	0.680	0.993	0.94 (0.17-5.27)	0.948	0.992
LRIG1	1.15 (0.62-2.13)	0.662	0.971	1.26 (0.40-3.93)	0.692	0.994	1.69 (0.53-5.41)	0.380	0.942
LRIG3	1.94 (1.13-3.33)	0.016	0.294	2.58 (1.16-5.76)	0.021	0.257	1.83 (0.71-4.77)	0.213	0.867
LRP1	1.19 (0.56-2.51)	0.647	0.971	1.93 (0.56-6.61)	0.298	0.804	2.49 (0.70-8.85)	0.158	0.827
LRP11	1.42 (0.84-2.39)	0.187	0.712	2.81 (1.16-6.80)	0.022	0.261	1.21 (0.48-3.04)	0.690	0.975
LRP2	0.90 (0.63-1.29)	0.574	0.956	0.27 (0.07-1.01)	0.052	0.391	1.00 (0.54-1.87)	0.991	0.998
LRP2BP	0.96 (0.68-1.35)	0.805	0.995	0.83 (0.40-1.72)	0.618	0.979	0.70 (0.31-1.58)	0.394	0.942
LRPAP1	1.02 (0.74-1.42)	0.888	0.999	0.72 (0.33-1.57)	0.404	0.902	1.41 (0.86-2.29)	0.172	0.853
LRRC25	1.06 (0.63-1.77)	0.839	0.998	1.29 (0.51-3.24)	0.592	0.970	0.99 (0.38-2.57)	0.991	0.998
LRRC37A2	1.63 (0.89-2.99)	0.111	0.598	1.33 (0.46-3.83)	0.598	0.973	1.35 (0.45-4.02)	0.589	0.965
LRRC38	1.29 (0.85-1.94)	0.229	0.762	1.35 (0.62-2.95)	0.446	0.917	1.64 (0.87-3.08)	0.125	0.795

LRRC59	0.96 (0.55-1.68)	0.897	0.999	0.93 (0.32-2.66)	0.887	0.997	0.70 (0.23-2.15)	0.528	0.949
LRRFIP1	1.02 (0.60-1.74)	0.936	0.999	0.75 (0.26-2.16)	0.599	0.973	0.83 (0.29-2.38)	0.731	0.978
LRRN1	0.80 (0.48-1.34)	0.398	0.894	2.64 (0.95-7.37)	0.064	0.428	0.69 (0.28-1.74)	0.437	0.942
LRTM1	1.13 (0.58-2.18)	0.717	0.975	1.41 (0.44-4.55)	0.569	0.963	0.50 (0.13-2.00)	0.330	0.934
LRTM2	0.64 (0.26-1.59)	0.338	0.852	0.84 (0.17-4.23)	0.835	0.997	2.15 (0.55-8.39)	0.270	0.910
LSM1	0.73 (0.47-1.13)	0.156	0.676	1.02 (0.51-2.01)	0.966	0.997	0.23 (0.09-0.58)	0.002	0.228
LSM8	1.00 (0.76-1.33)	0.995	0.999	0.87 (0.47-1.62)	0.670	0.993	1.24 (0.83-1.83)	0.292	0.915
LSP1	1.19 (0.68-2.08)	0.537	0.948	0.89 (0.32-2.50)	0.826	0.997	0.86 (0.31-2.40)	0.772	0.987
LTA	1.18 (0.66-2.12)	0.567	0.956	2.09 (0.75-5.88)	0.160	0.638	0.74 (0.27-2.04)	0.559	0.955
LTA4H	0.94 (0.65-1.35)	0.744	0.984	1.33 (0.71-2.48)	0.374	0.879	1.21 (0.63-2.31)	0.568	0.960
LTB	1.38 (0.63-3.02)	0.426	0.902	2.28 (0.62-8.35)	0.214	0.721	2.23 (0.66-7.55)	0.196	0.867
LTBP2	2.57 (1.38-4.78)	0.003	0.126	4.35 (1.39-13.62)	0.012	0.203	4.14 (1.48-11.55)	0.007	0.359
LTBP3	1.18 (0.79-1.78)	0.420	0.899	1.14 (0.53-2.45)	0.732	0.997	1.33 (0.64-2.74)	0.445	0.942
LTBR	2.05 (1.10-3.80)	0.023	0.348	3.11 (1.21-7.95)	0.018	0.241	1.86 (0.61-5.71)	0.278	0.910
LTO1	1.14 (0.72-1.81)	0.573	0.956	0.89 (0.31-2.57)	0.830	0.997	1.14 (0.42-3.12)	0.794	0.989
LUZP2	1.30 (0.90-1.88)	0.160	0.680	1.56 (0.89-2.73)	0.118	0.567	1.27 (0.67-2.38)	0.462	0.942
LXN	0.90 (0.65-1.26)	0.549	0.949	0.79 (0.43-1.45)	0.447	0.918	0.97 (0.58-1.63)	0.911	0.992
LY6D	1.51 (0.96-2.40)	0.077	0.514	1.80 (0.85-3.79)	0.122	0.579	1.29 (0.55-3.02)	0.560	0.955
LY75	1.30 (0.74-2.27)	0.363	0.865	1.13 (0.41-3.10)	0.817	0.997	1.45 (0.49-4.27)	0.501	0.947
LY9	1.42 (0.66-3.03)	0.368	0.871	2.22 (0.60-8.19)	0.231	0.742	0.79 (0.19-3.36)	0.754	0.985
LY96	1.32 (0.73-2.39)	0.363	0.865	2.76 (1.18-6.46)	0.019	0.251	0.61 (0.19-2.03)	0.423	0.942
LYAR	1.10 (0.75-1.63)	0.616	0.966	1.05 (0.48-2.31)	0.894	0.997	1.24 (0.67-2.29)	0.493	0.947
LYN	0.96 (0.73-1.25)	0.758	0.986	1.07 (0.66-1.76)	0.777	0.997	0.85 (0.51-1.41)	0.536	0.952
LYPD1	1.09 (0.77-1.55)	0.615	0.966	1.03 (0.50-2.12)	0.936	0.997	1.05 (0.53-2.07)	0.889	0.992
LYPD3	2.00 (1.23-3.26)	0.005	0.174	2.66 (1.26-5.60)	0.010	0.195	1.38 (0.48-3.98)	0.554	0.955

LYPD8	0.98 (0.63-1.52)	0.913	0.999	0.91 (0.40-2.08)	0.828	0.997	1.41 (0.80-2.49)	0.231	0.883
LYPLA2	1.05 (0.87-1.27)	0.596	0.962	1.25 (0.92-1.68)	0.153	0.626	0.90 (0.59-1.37)	0.609	0.966
LYSMD3	1.07 (0.87-1.31)	0.526	0.948	0.95 (0.64-1.43)	0.817	0.997	1.15 (0.80-1.65)	0.446	0.942
LYVE1	2.76 (0.89-8.55)	0.079	0.514	1.24 (0.12-12.58)	0.857	0.997	2.18 (0.20-23.68)	0.521	0.948
LYZL2	1.08 (0.70-1.67)	0.715	0.975	1.20 (0.56-2.59)	0.633	0.986	0.82 (0.33-2.05)	0.664	0.975
LZTFL1	0.79 (0.53-1.15)	0.217	0.746	1.06 (0.55-2.05)	0.870	0.997	0.52 (0.23-1.16)	0.110	0.763
M6PR	0.58 (0.24-1.42)	0.237	0.767	0.48 (0.09-2.52)	0.389	0.892	0.42 (0.08-2.25)	0.308	0.923
MAD1L1	1.13 (0.72-1.76)	0.594	0.962	1.26 (0.58-2.75)	0.561	0.962	0.83 (0.32-2.16)	0.696	0.975
MAEA	1.09 (0.64-1.88)	0.744	0.984	1.17 (0.46-3.01)	0.739	0.997	1.83 (0.85-3.93)	0.123	0.791
MAG	0.69 (0.43-1.12)	0.129	0.629	0.65 (0.27-1.58)	0.342	0.848	0.71 (0.29-1.74)	0.455	0.942
MAGEA3	1.09 (0.81-1.48)	0.555	0.950	1.31 (0.75-2.29)	0.336	0.839	0.97 (0.58-1.63)	0.920	0.992
MAGED1	1.11 (0.69-1.76)	0.673	0.972	0.52 (0.20-1.37)	0.189	0.681	1.99 (0.95-4.16)	0.067	0.718
MAMDC2	1.72 (0.91-3.24)	0.094	0.561	3.57 (1.27-10.00)	0.016	0.230	1.46 (0.48-4.45)	0.510	0.947
MAMDC4	1.32 (0.86-2.03)	0.198	0.724	1.99 (0.94-4.22)	0.072	0.460	1.49 (0.71-3.13)	0.287	0.915
MAN1A2	1.86 (0.71-4.88)	0.209	0.740	4.64 (0.82-26.32)	0.083	0.484	2.76 (0.45-16.99)	0.273	0.910
MAN2B2	0.74 (0.51-1.08)	0.114	0.605	1.43 (0.74-2.74)	0.285	0.796	0.53 (0.26-1.07)	0.076	0.718
MANEAL	1.62 (1.06-2.48)	0.026	0.352	2.59 (1.50-4.46)	<0.001	0.059	2.22 (1.15-4.28)	0.018	0.512
MANF	0.95 (0.78-1.15)	0.577	0.956	0.88 (0.63-1.22)	0.434	0.908	0.88 (0.62-1.23)	0.442	0.942
MANSC1	1.71 (0.79-3.70)	0.173	0.701	2.56 (0.65-10.03)	0.177	0.662	0.90 (0.22-3.72)	0.884	0.992
MANSC4	1.23 (0.88-1.71)	0.219	0.749	1.22 (0.70-2.15)	0.484	0.934	1.01 (0.54-1.88)	0.977	0.994
MAP1LC3A	2.14 (0.91-5.03)	0.083	0.528	2.31 (0.53-10.08)	0.265	0.774	1.85 (0.37-9.21)	0.450	0.942
MAP1LC3B2	0.95 (0.63-1.42)	0.800	0.995	1.12 (0.61-2.06)	0.709	0.995	0.50 (0.16-1.54)	0.229	0.879
MAP2	1.43 (0.90-2.26)	0.127	0.625	1.71 (0.82-3.58)	0.153	0.626	1.22 (0.47-3.18)	0.681	0.975
MAP2K1	0.99 (0.70-1.41)	0.953	0.999	1.32 (0.71-2.46)	0.386	0.889	1.20 (0.63-2.31)	0.575	0.960
MAP2K6	0.95 (0.75-1.20)	0.639	0.968	0.88 (0.57-1.35)	0.553	0.961	0.87 (0.56-1.38)	0.562	0.956

MAP3K5	1.05 (0.84-1.31)	0.687	0.975	0.79 (0.51-1.24)	0.309	0.816	1.20 (0.81-1.78)	0.370	0.939
MAP4K5	1.01 (0.83-1.24)	0.914	0.999	0.98 (0.68-1.42)	0.925	0.997	1.02 (0.69-1.51)	0.907	0.992
MAPK13	1.03 (0.53-2.00)	0.934	0.999	1.34 (0.41-4.34)	0.624	0.983	1.34 (0.45-3.96)	0.600	0.966
MAPK9	0.93 (0.55-1.56)	0.779	0.992	1.27 (0.50-3.24)	0.614	0.979	1.42 (0.57-3.53)	0.455	0.942
MAPKAPK2	1.07 (0.76-1.50)	0.717	0.975	1.38 (0.78-2.45)	0.272	0.782	0.62 (0.31-1.23)	0.171	0.853
MAPRE3	1.12 (0.97-1.31)	0.124	0.621	1.15 (0.89-1.50)	0.292	0.803	1.15 (0.86-1.54)	0.351	0.938
MAPT	1.42 (0.85-2.36)	0.177	0.706	1.47 (0.57-3.78)	0.425	0.905	1.27 (0.51-3.14)	0.608	0.966
MARCO	1.59 (0.68-3.73)	0.283	0.806	1.69 (0.36-7.93)	0.506	0.942	0.99 (0.20-4.88)	0.993	0.998
MARS1	0.94 (0.70-1.25)	0.664	0.971	1.09 (0.65-1.83)	0.739	0.997	0.83 (0.47-1.46)	0.520	0.948
MASP1	0.41 (0.16-1.09)	0.073	0.509	0.57 (0.10-3.28)	0.531	0.949	0.14 (0.02-0.81)	0.028	0.588
MATN2	1.46 (0.69-3.07)	0.324	0.843	1.08 (0.27-4.23)	0.917	0.997	2.19 (0.56-8.56)	0.262	0.910
MATN3	0.72 (0.36-1.42)	0.344	0.855	0.70 (0.20-2.50)	0.585	0.967	1.35 (0.38-4.74)	0.641	0.973
MAVS	1.02 (0.80-1.31)	0.850	0.998	0.89 (0.56-1.42)	0.627	0.984	1.05 (0.66-1.68)	0.829	0.991
MAX	0.99 (0.79-1.23)	0.900	0.999	0.98 (0.65-1.46)	0.914	0.997	0.88 (0.58-1.33)	0.531	0.951
MB	1.07 (0.76-1.51)	0.708	0.975	1.33 (0.75-2.34)	0.327	0.832	0.94 (0.50-1.76)	0.850	0.992
MBL2	0.85 (0.68-1.06)	0.152	0.673	1.13 (0.75-1.70)	0.556	0.962	0.86 (0.56-1.30)	0.469	0.942
MCAM	1.13 (0.62-2.07)	0.695	0.975	1.40 (0.50-3.94)	0.525	0.948	1.24 (0.44-3.55)	0.682	0.975
MCEE	1.44 (0.99-2.10)	0.056	0.460	0.99 (0.38-2.58)	0.977	0.997	1.02 (0.40-2.61)	0.967	0.994
MCEMP1	0.92 (0.68-1.24)	0.581	0.957	0.77 (0.43-1.38)	0.380	0.885	1.05 (0.61-1.79)	0.868	0.992
MCFD2	0.98 (0.58-1.65)	0.932	0.999	1.20 (0.47-3.07)	0.707	0.994	0.82 (0.30-2.26)	0.705	0.975
MCTS1	0.26 (0.08-0.85)	0.026	0.352	0.33 (0.04-2.65)	0.298	0.804	0.55 (0.08-3.82)	0.542	0.952
MDGA1	1.00 (0.72-1.37)	0.978	0.999	1.79 (0.97-3.31)	0.064	0.428	0.71 (0.40-1.26)	0.243	0.893
MDH1	0.79 (0.41-1.50)	0.465	0.922	1.54 (0.55-4.35)	0.415	0.903	1.05 (0.36-3.07)	0.923	0.992
MDK	1.04 (0.66-1.65)	0.865	0.998	1.53 (0.68-3.44)	0.301	0.809	0.70 (0.29-1.72)	0.442	0.942
MDM1	0.99 (0.44-2.22)	0.977	0.999	0.87 (0.20-3.87)	0.860	0.997	0.37 (0.08-1.65)	0.194	0.866

MECR	0.89 (0.66-1.20)	0.440	0.909	0.88 (0.51-1.50)	0.629	0.984	0.73 (0.41-1.28)	0.273	0.910
MED18	0.92 (0.68-1.24)	0.568	0.956	0.81 (0.47-1.40)	0.452	0.920	0.86 (0.49-1.52)	0.601	0.966
MED21	1.13 (0.71-1.80)	0.611	0.965	1.63 (0.94-2.82)	0.083	0.484	0.97 (0.33-2.84)	0.951	0.992
MEGF10	1.07 (0.59-1.92)	0.829	0.997	1.86 (0.63-5.44)	0.260	0.772	1.21 (0.44-3.36)	0.712	0.976
MEGF11	1.11 (0.47-2.61)	0.814	0.995	1.65 (0.37-7.30)	0.508	0.942	1.06 (0.19-5.76)	0.950	0.992
MEGF9	1.15 (0.40-3.33)	0.796	0.994	1.96 (0.28-13.69)	0.500	0.941	0.36 (0.05-2.56)	0.307	0.923
MELTF	1.42 (0.62-3.29)	0.407	0.894	4.77 (1.34-17.03)	0.016	0.234	1.92 (0.42-8.81)	0.399	0.942
MENT	1.50 (0.56-4.04)	0.423	0.901	2.00 (0.32-12.62)	0.459	0.923	1.22 (0.21-7.09)	0.823	0.991
MEP1A	0.98 (0.74-1.31)	0.904	0.999	0.84 (0.49-1.44)	0.521	0.946	1.29 (0.77-2.14)	0.333	0.934
MEP1B	0.87 (0.73-1.05)	0.149	0.673	1.08 (0.76-1.55)	0.658	0.992	0.86 (0.61-1.22)	0.401	0.942
MEPE	0.58 (0.27-1.26)	0.171	0.697	1.63 (0.34-7.91)	0.542	0.957	1.08 (0.24-4.83)	0.921	0.992
MERTK	2.26 (1.08-4.76)	0.031	0.376	0.81 (0.18-3.66)	0.785	0.997	1.64 (0.37-7.26)	0.512	0.947
MESD	0.94 (0.78-1.13)	0.520	0.947	0.98 (0.70-1.39)	0.931	0.997	0.87 (0.61-1.24)	0.434	0.942
MET	1.79 (0.61-5.24)	0.288	0.809	1.55 (0.23-10.52)	0.654	0.992	1.13 (0.19-6.59)	0.891	0.992
METAP1	1.26 (0.59-2.69)	0.542	0.948	1.36 (0.34-5.41)	0.660	0.992	1.60 (0.39-6.52)	0.515	0.947
METAP1D	0.96 (0.72-1.28)	0.784	0.994	0.90 (0.54-1.51)	0.695	0.994	0.92 (0.54-1.58)	0.770	0.987
METAP2	1.07 (0.70-1.63)	0.747	0.984	1.19 (0.55-2.57)	0.663	0.992	1.37 (0.60-3.14)	0.457	0.942
MFAP3	0.90 (0.55-1.47)	0.674	0.973	0.83 (0.33-2.05)	0.679	0.993	0.90 (0.38-2.17)	0.821	0.991
MFAP3L	0.90 (0.64-1.27)	0.552	0.950	0.80 (0.40-1.58)	0.517	0.945	0.47 (0.17-1.28)	0.140	0.808
MFAP4	1.37 (0.65-2.89)	0.411	0.894	2.56 (0.66-9.89)	0.172	0.658	1.94 (0.51-7.46)	0.332	0.934
MFAP5	1.77 (1.07-2.93)	0.027	0.362	2.79 (1.10-7.06)	0.030	0.308	2.17 (0.86-5.46)	0.101	0.762
MFGE8	0.97 (0.61-1.53)	0.896	0.999	1.14 (0.49-2.66)	0.756	0.997	1.05 (0.45-2.44)	0.912	0.992
MGLL	1.02 (0.79-1.34)	0.856	0.998	1.01 (0.62-1.63)	0.978	0.997	0.96 (0.59-1.55)	0.856	0.992
MGMT	0.98 (0.82-1.18)	0.867	0.998	0.98 (0.70-1.37)	0.912	0.997	0.98 (0.69-1.39)	0.918	0.992
MIA	1.42 (0.79-2.57)	0.242	0.769	1.03 (0.35-2.98)	0.960	0.997	1.25 (0.43-3.64)	0.681	0.975

MICALL2	1.04 (0.71-1.52)	0.832	0.997	1.04 (0.52-2.08)	0.911	0.997	1.34 (0.69-2.59)	0.382	0.942
MICB_MICA	1.21 (0.98-1.48)	0.071	0.498	1.17 (0.82-1.67)	0.376	0.880	1.34 (0.85-2.10)	0.211	0.867
MIF	0.99 (0.70-1.40)	0.949	0.999	0.94 (0.51-1.76)	0.856	0.997	1.01 (0.53-1.91)	0.980	0.995
MILR1	1.12 (0.74-1.71)	0.589	0.961	1.31 (0.62-2.76)	0.478	0.932	0.96 (0.44-2.11)	0.919	0.992
MINDY1	0.98 (0.78-1.23)	0.857	0.998	1.01 (0.66-1.54)	0.977	0.997	0.80 (0.51-1.26)	0.335	0.934
MINK1	0.88 (0.67-1.15)	0.344	0.855	1.06 (0.65-1.73)	0.804	0.997	0.73 (0.44-1.22)	0.228	0.878
MITD1	0.97 (0.76-1.23)	0.789	0.994	1.01 (0.65-1.56)	0.961	0.997	0.84 (0.54-1.31)	0.454	0.942
MKI67	1.28 (0.70-2.31)	0.422	0.899	2.56 (1.32-4.96)	0.006	0.146	1.80 (0.83-3.91)	0.136	0.796
MLLT1	1.08 (0.78-1.50)	0.635	0.968	1.13 (0.64-2.00)	0.671	0.993	1.13 (0.59-2.17)	0.712	0.976
MLN	1.00 (0.77-1.29)	0.978	0.999	1.14 (0.72-1.82)	0.567	0.963	1.24 (0.76-2.01)	0.391	0.942
MME	1.41 (1.12-1.78)	0.004	0.140	1.42 (0.93-2.17)	0.103	0.528	1.11 (0.68-1.79)	0.680	0.975
MMP1	1.06 (0.83-1.34)	0.647	0.971	0.90 (0.59-1.37)	0.618	0.979	1.25 (0.79-1.96)	0.337	0.934
MMP10	0.88 (0.60-1.30)	0.515	0.946	0.92 (0.45-1.86)	0.819	0.997	0.83 (0.40-1.72)	0.624	0.968
MMP12	1.36 (0.90-2.06)	0.149	0.672	1.71 (0.81-3.60)	0.160	0.637	1.03 (0.48-2.21)	0.936	0.992
MMP13	1.41 (0.73-2.73)	0.305	0.822	2.65 (0.80-8.77)	0.112	0.551	1.87 (0.58-6.07)	0.295	0.916
MMP15	0.89 (0.52-1.53)	0.671	0.971	1.33 (0.71-2.50)	0.369	0.873	0.30 (0.05-1.98)	0.211	0.867
MMP3	0.75 (0.49-1.17)	0.206	0.740	0.90 (0.40-1.98)	0.786	0.997	0.90 (0.40-1.98)	0.785	0.989
MMP7	1.62 (1.04-2.53)	0.033	0.388	2.12 (0.99-4.57)	0.054	0.392	1.55 (0.69-3.48)	0.294	0.915
MMP8	1.25 (0.93-1.67)	0.134	0.639	1.01 (0.58-1.74)	0.979	0.997	1.06 (0.61-1.82)	0.842	0.992
MMP9	1.08 (0.75-1.56)	0.667	0.971	0.71 (0.37-1.35)	0.298	0.805	1.40 (0.73-2.70)	0.312	0.923
MMUT	1.06 (0.63-1.77)	0.825	0.995	0.75 (0.28-2.00)	0.560	0.962	0.75 (0.28-2.04)	0.576	0.961
MN1	0.95 (0.59-1.54)	0.828	0.996	1.19 (0.56-2.51)	0.655	0.992	0.46 (0.14-1.54)	0.209	0.867
MNAT1	0.90 (0.47-1.73)	0.759	0.987	0.35 (0.09-1.43)	0.145	0.612	1.13 (0.36-3.57)	0.835	0.991
MNDA	1.12 (0.91-1.36)	0.286	0.806	0.96 (0.62-1.47)	0.835	0.997	0.92 (0.60-1.40)	0.690	0.975
MOCS2	0.95 (0.65-1.40)	0.801	0.995	1.47 (0.75-2.87)	0.262	0.772	0.97 (0.47-1.99)	0.926	0.992

MOG	1.63 (0.94-2.84)	0.084	0.535	0.92 (0.31-2.70)	0.878	0.997	1.70 (0.62-4.63)	0.299	0.917
MORC3	1.88 (0.95-3.72)	0.069	0.490	0.51 (0.09-2.84)	0.440	0.912	3.42 (1.53-7.61)	0.003	0.281
MORF4L1	1.72 (1.11-2.65)	0.015	0.287	1.77 (0.86-3.64)	0.121	0.575	1.85 (0.88-3.88)	0.104	0.763
MORF4L2	1.86 (1.18-2.94)	0.007	0.195	1.89 (0.81-4.42)	0.143	0.607	0.85 (0.28-2.57)	0.777	0.987
MORN4	1.24 (0.93-1.66)	0.143	0.661	1.58 (1.04-2.41)	0.034	0.318	0.77 (0.38-1.54)	0.453	0.942
MPHOSPH8	0.86 (0.62-1.20)	0.375	0.875	1.00 (0.56-1.81)	0.987	0.998	0.75 (0.40-1.40)	0.367	0.939
MPI	1.06 (0.80-1.40)	0.671	0.971	1.10 (0.66-1.82)	0.715	0.995	0.94 (0.55-1.60)	0.821	0.991
MPIG6B	1.02 (0.74-1.41)	0.904	0.999	1.03 (0.57-1.86)	0.912	0.997	0.85 (0.48-1.50)	0.577	0.961
MPO	1.52 (1.02-2.27)	0.039	0.413	1.21 (0.53-2.76)	0.658	0.992	1.16 (0.52-2.61)	0.717	0.976
MPRIP	1.00 (0.54-1.85)	0.992	0.999	1.90 (0.76-4.73)	0.168	0.649	0.54 (0.18-1.67)	0.287	0.915
MRC1	1.76 (0.75-4.13)	0.192	0.718	1.84 (0.39-8.57)	0.440	0.912	2.92 (0.67-12.64)	0.152	0.816
MRI1	0.93 (0.64-1.34)	0.686	0.975	1.04 (0.52-2.07)	0.916	0.997	0.70 (0.38-1.30)	0.255	0.901
MRPL24	1.07 (0.56-2.01)	0.843	0.998	0.98 (0.30-3.25)	0.978	0.997	1.44 (0.62-3.38)	0.398	0.942
MRPL28	0.51 (0.19-1.36)	0.179	0.706	0.57 (0.10-3.33)	0.530	0.949	0.44 (0.07-3.00)	0.404	0.942
MRPL46	0.63 (0.26-1.54)	0.313	0.830	0.46 (0.09-2.40)	0.355	0.862	1.26 (0.28-5.74)	0.764	0.987
MRPL52	1.12 (0.44-2.85)	0.817	0.995	0.97 (0.17-5.45)	0.976	0.997	1.90 (0.33-10.97)	0.474	0.944
MRPL58	0.68 (0.44-1.07)	0.096	0.568	0.54 (0.22-1.32)	0.178	0.662	0.28 (0.09-0.85)	0.025	0.582
MRPS16	1.06 (0.38-2.93)	0.917	0.999	1.19 (0.21-6.71)	0.843	0.997	0.45 (0.04-4.55)	0.499	0.947
MSLN	1.20 (0.85-1.70)	0.306	0.823	0.63 (0.32-1.23)	0.174	0.660	2.84 (1.60-5.03)	<0.001	0.157
MSLNL	0.55 (0.24-1.28)	0.166	0.692	0.89 (0.23-3.41)	0.869	0.997	0.42 (0.08-2.10)	0.290	0.915
MSMB	1.06 (0.78-1.44)	0.704	0.975	0.94 (0.54-1.65)	0.839	0.997	1.21 (0.71-2.06)	0.483	0.944
MSR1	1.28 (0.83-1.99)	0.263	0.790	1.45 (0.66-3.17)	0.350	0.858	1.19 (0.51-2.77)	0.684	0.975
MSRA	0.91 (0.66-1.26)	0.561	0.956	0.96 (0.53-1.75)	0.905	0.997	0.67 (0.36-1.25)	0.211	0.867
MST1	1.52 (0.88-2.63)	0.135	0.640	1.27 (0.50-3.23)	0.611	0.979	1.96 (0.64-6.00)	0.236	0.885
MSTN	0.85 (0.56-1.30)	0.457	0.920	1.06 (0.49-2.29)	0.889	0.997	0.77 (0.36-1.65)	0.504	0.947

MTDH	0.89 (0.67-1.18)	0.410	0.894	0.88 (0.52-1.49)	0.641	0.990	0.60 (0.33-1.08)	0.088	0.745
MTHFD2	1.26 (0.46-3.46)	0.648	0.971	2.63 (0.56-12.35)	0.219	0.729	0.40 (0.04-3.59)	0.414	0.942
MTHFSD	1.10 (0.89-1.36)	0.365	0.868	1.03 (0.70-1.52)	0.867	0.997	0.88 (0.59-1.32)	0.546	0.954
MTIF3	0.91 (0.70-1.18)	0.469	0.924	0.99 (0.62-1.58)	0.961	0.997	0.72 (0.43-1.19)	0.203	0.867
MTPN	0.81 (0.36-1.81)	0.608	0.963	0.97 (0.22-4.24)	0.963	0.997	1.14 (0.27-4.87)	0.863	0.992
MTR	1.13 (0.82-1.55)	0.456	0.920	1.23 (0.74-2.03)	0.428	0.907	0.99 (0.50-1.97)	0.974	0.994
MTSS1	1.17 (0.94-1.45)	0.157	0.677	1.13 (0.76-1.67)	0.541	0.956	1.32 (0.88-1.97)	0.173	0.853
MTSS2	1.01 (0.78-1.31)	0.923	0.999	0.96 (0.59-1.56)	0.860	0.997	0.85 (0.51-1.41)	0.532	0.951
MTUS1	1.10 (0.70-1.73)	0.671	0.971	0.48 (0.18-1.27)	0.141	0.604	0.91 (0.37-2.25)	0.834	0.991
MUC13	1.22 (0.83-1.79)	0.308	0.825	0.87 (0.42-1.77)	0.698	0.994	1.84 (0.98-3.47)	0.058	0.698
MUC16	0.89 (0.80-0.99)	0.038	0.411	1.00 (0.81-1.25)	0.968	0.997	0.99 (0.79-1.22)	0.891	0.992
MUC2	1.01 (0.76-1.34)	0.928	0.999	1.35 (0.82-2.22)	0.235	0.742	0.96 (0.57-1.61)	0.873	0.992
MUCL3	1.15 (0.66-1.99)	0.615	0.966	1.70 (0.76-3.82)	0.200	0.707	0.98 (0.31-3.09)	0.967	0.994
MVK	1.10 (0.85-1.42)	0.478	0.928	1.02 (0.63-1.65)	0.942	0.997	1.17 (0.73-1.87)	0.507	0.947
MXRA8	1.23 (0.57-2.66)	0.602	0.962	9.54 (2.51-36.24)	<0.001	0.063	2.55 (0.63-10.35)	0.190	0.862
MYBPC1	0.89 (0.66-1.20)	0.437	0.907	1.29 (0.81-2.05)	0.282	0.793	1.06 (0.64-1.75)	0.828	0.991
MYBPC2	1.01 (0.78-1.29)	0.967	0.999	1.30 (0.89-1.89)	0.174	0.661	0.73 (0.40-1.32)	0.294	0.915
MYCBP2	0.86 (0.56-1.32)	0.492	0.936	1.57 (0.86-2.88)	0.145	0.611	0.65 (0.27-1.56)	0.335	0.934
MYDGF	0.89 (0.68-1.15)	0.375	0.874	1.17 (0.74-1.85)	0.509	0.942	0.73 (0.44-1.22)	0.227	0.878
MYH4	1.09 (0.69-1.72)	0.710	0.975	1.30 (0.66-2.56)	0.442	0.914	0.86 (0.35-2.09)	0.735	0.982
MYH7B	0.93 (0.67-1.29)	0.671	0.971	0.80 (0.39-1.64)	0.549	0.960	0.78 (0.37-1.61)	0.498	0.947
MYH9	1.13 (0.90-1.41)	0.290	0.809	0.98 (0.65-1.47)	0.905	0.997	1.05 (0.69-1.60)	0.824	0.991
MYL1	0.58 (0.29-1.18)	0.132	0.632	1.24 (0.48-3.17)	0.660	0.992	0.21 (0.05-0.91)	0.037	0.623
MYL3	1.04 (0.78-1.39)	0.804	0.995	1.12 (0.66-1.90)	0.666	0.993	0.92 (0.54-1.58)	0.764	0.987
MYL4	1.04 (0.67-1.61)	0.862	0.998	0.87 (0.38-1.98)	0.737	0.997	1.15 (0.59-2.24)	0.681	0.975

MYL6B	1.16 (0.90-1.48)	0.257	0.786	1.35 (0.90-2.02)	0.142	0.607	0.98 (0.56-1.73)	0.955	0.992
MYLPF	1.29 (0.96-1.75)	0.095	0.568	1.73 (1.07-2.80)	0.026	0.281	1.42 (0.86-2.36)	0.175	0.853
MYO6	0.92 (0.40-2.08)	0.834	0.997	0.69 (0.15-3.23)	0.638	0.987	0.42 (0.07-2.36)	0.324	0.931
MYO9B	0.97 (0.68-1.38)	0.874	0.998	0.89 (0.46-1.74)	0.734	0.997	0.64 (0.30-1.38)	0.254	0.901
MYOC	1.22 (0.74-2.02)	0.434	0.906	1.35 (0.54-3.39)	0.519	0.945	3.20 (1.60-6.39)	<0.001	0.180
MYOM1	0.76 (0.39-1.47)	0.411	0.894	1.26 (0.57-2.77)	0.566	0.963	0.53 (0.14-2.09)	0.367	0.939
MYOM2	0.85 (0.56-1.31)	0.466	0.922	1.17 (0.59-2.34)	0.649	0.992	0.59 (0.24-1.48)	0.259	0.907
MYOM3	0.94 (0.75-1.18)	0.573	0.956	1.13 (0.76-1.68)	0.552	0.960	0.84 (0.55-1.29)	0.435	0.942
MZB1	1.24 (0.83-1.84)	0.300	0.818	1.28 (0.63-2.63)	0.497	0.940	0.76 (0.34-1.66)	0.483	0.944
MZT1	0.75 (0.46-1.22)	0.243	0.769	0.53 (0.20-1.40)	0.202	0.709	0.81 (0.33-2.03)	0.661	0.975
NAA10	1.23 (0.72-2.08)	0.451	0.916	1.09 (0.41-2.91)	0.858	0.997	0.90 (0.35-2.34)	0.827	0.991
NAA80	0.92 (0.75-1.14)	0.452	0.916	1.14 (0.78-1.67)	0.496	0.940	0.69 (0.45-1.05)	0.083	0.743
NAAA	0.83 (0.56-1.22)	0.341	0.854	0.72 (0.35-1.46)	0.359	0.862	0.66 (0.31-1.38)	0.267	0.910
NACC1	1.46 (0.97-2.18)	0.069	0.493	1.62 (0.77-3.40)	0.206	0.712	1.22 (0.54-2.76)	0.635	0.973
NADK	1.28 (0.91-1.81)	0.152	0.673	1.01 (0.49-2.08)	0.975	0.997	0.90 (0.42-1.92)	0.790	0.989
NAGA	1.00 (0.63-1.59)	0.986	0.999	1.27 (0.64-2.54)	0.498	0.941	0.71 (0.25-2.07)	0.535	0.952
NAGK	0.85 (0.53-1.35)	0.483	0.933	1.19 (0.52-2.70)	0.684	0.993	1.08 (0.48-2.43)	0.846	0.992
NAGPA	1.78 (0.62-5.16)	0.286	0.806	12.74 (1.64-98.98)	0.015	0.229	0.67 (0.10-4.55)	0.685	0.975
NAMPT	1.11 (0.75-1.63)	0.597	0.962	1.05 (0.49-2.24)	0.900	0.997	0.82 (0.35-1.94)	0.658	0.975
NAP1L4	0.97 (0.56-1.66)	0.903	0.999	1.24 (0.45-3.47)	0.675	0.993	1.12 (0.41-3.03)	0.831	0.991
NAPRT	1.06 (0.51-2.21)	0.877	0.998	0.91 (0.23-3.59)	0.896	0.997	2.18 (0.93-5.07)	0.072	0.718
NARS1	0.96 (0.56-1.64)	0.888	0.999	1.52 (0.60-3.81)	0.375	0.879	0.58 (0.21-1.58)	0.288	0.915
NBL1	2.20 (1.10-4.38)	0.025	0.352	2.89 (0.89-9.39)	0.078	0.471	1.72 (0.48-6.11)	0.401	0.942
NBN	0.92 (0.58-1.46)	0.732	0.979	1.08 (0.50-2.34)	0.835	0.997	0.67 (0.26-1.71)	0.398	0.942
NCAM1	0.94 (0.48-1.82)	0.844	0.998	1.42 (0.46-4.43)	0.541	0.956	0.51 (0.15-1.74)	0.282	0.914

NCAM2	1.73 (0.82-3.63)	0.148	0.671	2.65 (0.68-10.33)	0.159	0.635	0.74 (0.18-3.04)	0.674	0.975
NCAN	0.83 (0.49-1.41)	0.489	0.936	0.38 (0.15-1.02)	0.054	0.393	0.98 (0.37-2.56)	0.960	0.994
NCF2	1.13 (0.88-1.44)	0.345	0.855	1.06 (0.65-1.72)	0.821	0.997	0.78 (0.45-1.35)	0.373	0.940
NCK2	1.01 (0.81-1.26)	0.917	0.999	0.99 (0.67-1.47)	0.968	0.997	0.96 (0.64-1.44)	0.859	0.992
NCLN	0.99 (0.59-1.64)	0.962	0.999	0.40 (0.10-1.67)	0.210	0.713	1.34 (0.58-3.07)	0.489	0.946
NCR1	1.73 (1.00-2.98)	0.049	0.431	2.35 (0.92-5.97)	0.073	0.460	1.62 (0.62-4.21)	0.322	0.929
NCR3LG1	1.70 (0.90-3.23)	0.102	0.576	1.95 (0.66-5.78)	0.229	0.739	3.31 (1.34-8.17)	0.009	0.402
NCS1	2.58 (1.30-5.15)	0.007	0.195	3.50 (1.02-11.98)	0.046	0.373	4.66 (1.43-15.22)	0.011	0.429
NDRG1	1.03 (0.73-1.45)	0.864	0.998	1.62 (1.12-2.35)	0.010	0.195	0.72 (0.32-1.61)	0.423	0.942
NDST1	1.22 (0.96-1.54)	0.102	0.576	0.88 (0.49-1.59)	0.670	0.993	1.16 (0.76-1.77)	0.481	0.944
NDUFA5	1.06 (0.55-2.07)	0.855	0.998	1.09 (0.33-3.58)	0.892	0.997	0.76 (0.22-2.71)	0.676	0.975
NDUFB7	0.90 (0.68-1.19)	0.452	0.916	1.07 (0.64-1.76)	0.806	0.997	0.59 (0.34-1.02)	0.057	0.698
NDUFS6	1.11 (0.85-1.43)	0.448	0.915	0.91 (0.50-1.65)	0.754	0.997	1.15 (0.73-1.81)	0.543	0.952
NEB	1.37 (0.81-2.31)	0.242	0.769	1.66 (0.68-4.04)	0.261	0.772	0.51 (0.13-1.95)	0.324	0.931
NECAP2	1.00 (0.68-1.48)	0.991	0.999	1.07 (0.54-2.10)	0.849	0.997	1.08 (0.54-2.13)	0.831	0.991
NECTIN1	1.39 (0.73-2.63)	0.317	0.834	0.59 (0.13-2.63)	0.485	0.934	1.45 (0.40-5.22)	0.574	0.960
NECTIN2	1.62 (0.86-3.07)	0.136	0.641	2.01 (0.65-6.24)	0.227	0.738	0.83 (0.24-2.90)	0.776	0.987
NECTIN4	1.49 (0.87-2.55)	0.147	0.669	1.46 (0.57-3.73)	0.432	0.908	1.17 (0.42-3.22)	0.768	0.987
NEDD4L	0.70 (0.43-1.14)	0.154	0.676	0.91 (0.38-2.22)	0.842	0.997	0.69 (0.32-1.49)	0.340	0.935
NEDD9	1.43 (0.75-2.71)	0.276	0.804	2.31 (0.85-6.31)	0.101	0.526	1.14 (0.32-4.09)	0.846	0.992
NEFL	2.26 (1.64-3.12)	<0.001	0.002	1.90 (1.04-3.45)	0.036	0.326	2.11 (1.18-3.78)	0.012	0.433
NEK7	0.81 (0.54-1.23)	0.333	0.847	1.08 (0.59-1.98)	0.802	0.997	0.95 (0.49-1.86)	0.885	0.992
NELL1	1.05 (0.65-1.69)	0.842	0.998	1.34 (0.58-3.09)	0.487	0.935	0.73 (0.30-1.80)	0.495	0.947
NELL2	1.38 (0.66-2.89)	0.387	0.882	2.79 (0.78-10.00)	0.115	0.563	1.37 (0.34-5.43)	0.656	0.975
NENF	1.06 (0.86-1.31)	0.576	0.956	1.15 (0.80-1.65)	0.449	0.919	0.99 (0.63-1.56)	0.961	0.994

NEO1	1.47 (0.54-4.05)	0.452	0.916	1.23 (0.19-7.94)	0.831	0.997	4.55 (1.21-17.14)	0.025	0.582
NEXN	0.94 (0.65-1.37)	0.746	0.984	0.93 (0.47-1.84)	0.836	0.997	0.87 (0.42-1.78)	0.700	0.975
NFASC	1.78 (0.90-3.53)	0.100	0.573	3.69 (1.06-12.78)	0.040	0.347	2.31 (0.68-7.79)	0.178	0.853
NFAT5	1.07 (0.81-1.40)	0.646	0.971	0.93 (0.54-1.60)	0.796	0.997	0.87 (0.49-1.52)	0.615	0.966
NFATC1	1.04 (0.83-1.31)	0.720	0.975	0.99 (0.65-1.50)	0.944	0.997	0.99 (0.64-1.54)	0.978	0.994
NFATC3	1.41 (1.00-1.99)	0.049	0.431	1.09 (0.56-2.12)	0.805	0.997	1.62 (0.89-2.96)	0.113	0.768
NFE2	0.96 (0.70-1.34)	0.826	0.995	1.09 (0.60-1.95)	0.783	0.997	0.99 (0.54-1.83)	0.976	0.994
NFIC	0.99 (0.58-1.69)	0.964	0.999	1.61 (0.93-2.76)	0.087	0.491	0.27 (0.06-1.34)	0.109	0.763
NFKB1	0.97 (0.67-1.39)	0.861	0.998	1.08 (0.55-2.11)	0.821	0.997	0.93 (0.47-1.86)	0.839	0.991
NFKB2	0.61 (0.29-1.27)	0.186	0.711	0.79 (0.28-2.29)	0.669	0.993	1.17 (0.55-2.50)	0.686	0.975
NFKBIE	0.86 (0.53-1.37)	0.518	0.947	0.68 (0.28-1.68)	0.403	0.902	1.05 (0.45-2.44)	0.906	0.992
NFU1	1.01 (0.78-1.29)	0.968	0.999	1.04 (0.66-1.64)	0.866	0.997	0.87 (0.55-1.38)	0.559	0.955
NFX1	1.21 (0.85-1.72)	0.286	0.806	1.70 (0.93-3.10)	0.083	0.484	1.04 (0.52-2.05)	0.915	0.992
NFYA	0.57 (0.25-1.28)	0.171	0.697	1.20 (0.45-3.19)	0.719	0.995	0.65 (0.16-2.72)	0.558	0.955
NGF	0.98 (0.15-6.51)	0.986	0.999	0.44 (0.01-15.17)	0.653	0.992	0.84 (0.03-26.10)	0.918	0.992
NGFR	1.21 (0.60-2.45)	0.593	0.961	1.92 (0.84-4.40)	0.124	0.581	0.27 (0.05-1.58)	0.147	0.813
NGRN	0.94 (0.32-2.74)	0.907	0.999	1.72 (0.33-8.92)	0.520	0.946	0.98 (0.15-6.27)	0.986	0.996
NHLRC3	2.50 (1.22-5.14)	0.013	0.268	3.58 (0.93-13.72)	0.063	0.426	2.87 (0.76-10.84)	0.120	0.783
NID1	1.20 (0.67-2.18)	0.538	0.948	1.60 (0.57-4.51)	0.377	0.880	0.66 (0.23-1.89)	0.440	0.942
NID2	0.85 (0.62-1.17)	0.315	0.831	1.07 (0.62-1.85)	0.820	0.997	0.74 (0.41-1.33)	0.318	0.923
NINJ1	0.87 (0.55-1.37)	0.540	0.948	1.00 (0.47-2.13)	0.991	0.998	1.15 (0.58-2.28)	0.691	0.975
NIT1	0.84 (0.50-1.38)	0.486	0.934	1.15 (0.45-2.95)	0.769	0.997	0.99 (0.38-2.59)	0.977	0.994
NIT2	0.81 (0.58-1.14)	0.231	0.763	1.25 (0.71-2.22)	0.444	0.914	0.69 (0.37-1.30)	0.249	0.901
NLGN1	1.04 (0.72-1.50)	0.825	0.995	1.29 (0.74-2.24)	0.370	0.874	0.56 (0.24-1.32)	0.189	0.862
NLGN2	0.83 (0.44-1.58)	0.576	0.956	1.22 (0.48-3.10)	0.671	0.993	0.44 (0.11-1.70)	0.234	0.884

NME1	0.84 (0.23-3.02)	0.787	0.994	1.06 (0.11-10.20)	0.958	0.997	0.35 (0.03-3.99)	0.395	0.942
NME3	0.93 (0.35-2.44)	0.876	0.998	3.15 (0.86-11.59)	0.084	0.486	0.43 (0.07-2.60)	0.356	0.939
NMI	1.10 (0.78-1.57)	0.580	0.956	1.95 (1.09-3.50)	0.024	0.271	0.83 (0.42-1.64)	0.597	0.966
NMNAT1	0.98 (0.72-1.34)	0.918	0.999	0.73 (0.38-1.42)	0.357	0.862	0.88 (0.50-1.57)	0.667	0.975
NMRK2	0.88 (0.44-1.77)	0.715	0.975	1.31 (0.50-3.45)	0.584	0.967	0.22 (0.04-1.36)	0.104	0.763
NMT1	0.88 (0.55-1.39)	0.578	0.956	1.12 (0.52-2.44)	0.770	0.997	0.54 (0.21-1.44)	0.222	0.873
NOMO1	1.06 (0.44-2.55)	0.889	0.999	4.82 (1.03-22.58)	0.046	0.373	0.82 (0.16-4.33)	0.816	0.991
NOP56	1.00 (0.80-1.24)	0.991	0.999	1.23 (0.92-1.65)	0.156	0.633	0.86 (0.53-1.40)	0.547	0.954
NOS1	1.18 (0.83-1.69)	0.361	0.865	1.71 (0.95-3.08)	0.076	0.467	1.37 (0.76-2.47)	0.295	0.916
NOS2	1.05 (0.51-2.17)	0.888	0.999	2.45 (0.77-7.75)	0.128	0.590	0.93 (0.25-3.49)	0.917	0.992
NOS3	1.33 (0.92-1.93)	0.131	0.632	2.07 (1.24-3.45)	0.005	0.146	0.95 (0.41-2.21)	0.906	0.992
NOTCH1	2.31 (0.62-8.56)	0.210	0.741	3.47 (0.33-36.59)	0.300	0.808	1.57 (0.14-17.15)	0.710	0.976
NOTCH2	1.12 (0.36-3.52)	0.842	0.998	12.45 (2.12-72.99)	0.005	0.146	0.33 (0.03-3.16)	0.337	0.934
NOTCH3	1.34 (0.73-2.47)	0.342	0.854	1.95 (0.65-5.92)	0.236	0.742	1.60 (0.53-4.84)	0.407	0.942
NPC2	1.83 (0.94-3.59)	0.077	0.514	4.70 (1.70-13.02)	0.003	0.110	1.54 (0.46-5.14)	0.484	0.944
NPDC1	1.67 (1.02-2.73)	0.043	0.421	2.28 (1.03-5.06)	0.043	0.362	1.04 (0.37-2.97)	0.935	0.992
NPHS1	0.54 (0.28-1.02)	0.057	0.461	0.80 (0.25-2.52)	0.705	0.994	0.30 (0.10-0.93)	0.037	0.623
NPHS2	1.08 (0.86-1.35)	0.502	0.943	1.50 (1.10-2.05)	0.011	0.199	0.78 (0.47-1.29)	0.327	0.932
NPL	0.93 (0.56-1.56)	0.791	0.994	1.44 (0.57-3.60)	0.438	0.912	1.29 (0.56-3.00)	0.554	0.955
NPPB	1.03 (0.88-1.21)	0.726	0.977	1.03 (0.76-1.41)	0.842	0.997	1.27 (0.96-1.69)	0.100	0.762
NPPC	1.00 (0.69-1.45)	0.990	0.999	1.37 (0.74-2.55)	0.321	0.824	0.85 (0.43-1.68)	0.636	0.973
NPR1	1.31 (0.69-2.47)	0.404	0.894	1.55 (0.54-4.50)	0.418	0.903	0.80 (0.19-3.39)	0.760	0.987
NPTN	1.12 (0.89-1.42)	0.322	0.839	1.44 (1.08-1.91)	0.014	0.220	1.28 (0.87-1.87)	0.211	0.867
NPTX1	1.43 (0.89-2.30)	0.135	0.640	1.39 (0.58-3.34)	0.458	0.923	2.13 (0.94-4.83)	0.069	0.718
NPTX2	1.48 (0.74-2.96)	0.271	0.799	5.37 (1.51-19.07)	0.009	0.188	2.35 (0.66-8.40)	0.188	0.862

NPTXR	0.61 (0.31-1.19)	0.149	0.672	0.67 (0.19-2.32)	0.527	0.948	1.02 (0.34-3.10)	0.970	0.994
NPY	1.33 (0.94-1.87)	0.103	0.578	1.29 (0.69-2.39)	0.423	0.905	1.58 (0.81-3.08)	0.180	0.853
NRCAM	2.01 (1.04-3.87)	0.037	0.405	2.95 (0.94-9.24)	0.064	0.428	0.65 (0.18-2.32)	0.508	0.947
NRGN	0.97 (0.73-1.29)	0.815	0.995	1.15 (0.69-1.93)	0.585	0.967	0.76 (0.44-1.32)	0.329	0.934
NRN1	1.06 (0.45-2.48)	0.900	0.999	0.96 (0.21-4.50)	0.962	0.997	0.67 (0.13-3.62)	0.645	0.973
NRP1	1.30 (0.65-2.60)	0.459	0.920	3.18 (0.91-11.18)	0.071	0.456	0.86 (0.25-2.94)	0.813	0.991
NRP2	1.90 (0.87-4.18)	0.108	0.593	3.73 (0.87-16.00)	0.077	0.470	1.89 (0.44-8.12)	0.390	0.942
NRTN	1.30 (0.85-1.97)	0.222	0.754	2.07 (1.22-3.50)	0.007	0.159	1.26 (0.59-2.68)	0.550	0.955
NRXN3	1.16 (0.71-1.88)	0.549	0.949	0.60 (0.14-2.53)	0.484	0.934	1.04 (0.39-2.77)	0.944	0.992
NSFL1C	1.11 (0.80-1.55)	0.527	0.948	0.98 (0.54-1.78)	0.951	0.997	1.26 (0.70-2.25)	0.440	0.942
NT5C	0.87 (0.62-1.24)	0.447	0.914	1.39 (0.75-2.58)	0.295	0.803	0.80 (0.43-1.48)	0.477	0.944
NT5C1A	2.18 (1.37-3.47)	0.001	0.075	2.16 (0.98-4.77)	0.058	0.405	4.10 (2.12-7.91)	<0.001	0.039
NT5C3A	0.97 (0.75-1.25)	0.834	0.997	1.04 (0.65-1.67)	0.872	0.997	0.95 (0.59-1.54)	0.845	0.992
NT5E	1.35 (0.93-1.95)	0.115	0.607	1.57 (0.83-2.97)	0.163	0.643	0.68 (0.29-1.60)	0.380	0.942
NTF3	0.92 (0.51-1.66)	0.778	0.992	1.82 (0.82-4.04)	0.141	0.604	1.03 (0.33-3.24)	0.959	0.994
NTF4	0.78 (0.40-1.55)	0.485	0.933	1.41 (0.64-3.14)	0.396	0.897	0.38 (0.09-1.69)	0.203	0.867
NTproBNP	1.26 (1.07-1.50)	0.007	0.190	1.44 (1.07-1.94)	0.018	0.236	1.44 (1.09-1.90)	0.009	0.402
NTRK2	3.71 (1.55-8.86)	0.003	0.132	20.10 (3.87-104.43)	<0.001	0.047	1.32 (0.27-6.47)	0.730	0.978
NTRK3	1.12 (0.44-2.90)	0.810	0.995	0.95 (0.16-5.66)	0.957	0.997	1.21 (0.22-6.66)	0.829	0.991
NUB1	0.94 (0.71-1.25)	0.681	0.975	0.96 (0.57-1.62)	0.877	0.997	0.82 (0.48-1.42)	0.482	0.944
NUBP1	1.22 (0.96-1.53)	0.100	0.573	1.13 (0.71-1.79)	0.600	0.973	1.16 (0.70-1.93)	0.562	0.956
NUCB2	1.18 (0.65-2.16)	0.589	0.961	1.60 (0.54-4.70)	0.394	0.896	1.54 (0.49-4.83)	0.455	0.942
NUDC	1.08 (0.74-1.58)	0.673	0.972	1.23 (0.61-2.50)	0.562	0.962	1.10 (0.53-2.26)	0.799	0.991
NUDT10	1.33 (0.46-3.83)	0.598	0.962	1.46 (0.22-9.56)	0.694	0.994	1.25 (0.16-9.76)	0.829	0.991
NUDT15	1.38 (0.96-1.98)	0.078	0.514	1.64 (1.01-2.64)	0.044	0.363	0.97 (0.34-2.78)	0.954	0.992

NUDT16	1.06 (0.79-1.41)	0.718	0.975	1.37 (0.81-2.31)	0.236	0.742	0.95 (0.55-1.64)	0.855	0.992
NUDT2	0.59 (0.39-0.88)	0.011	0.245	0.82 (0.42-1.62)	0.571	0.963	0.41 (0.18-0.94)	0.036	0.623
NUDT5	1.03 (0.65-1.63)	0.900	0.999	1.08 (0.48-2.46)	0.850	0.997	1.05 (0.46-2.42)	0.910	0.992
NUMB	1.02 (0.75-1.40)	0.883	0.999	1.28 (0.73-2.27)	0.391	0.893	0.76 (0.41-1.41)	0.383	0.942
NUP50	0.65 (0.25-1.67)	0.366	0.869	1.10 (0.25-4.75)	0.901	0.997	0.56 (0.09-3.37)	0.528	0.949
NXPE4	1.38 (0.88-2.15)	0.159	0.680	1.98 (1.19-3.29)	0.009	0.182	0.69 (0.14-3.45)	0.647	0.973
NXPH1	0.95 (0.52-1.74)	0.864	0.998	0.92 (0.29-2.86)	0.880	0.997	1.26 (0.52-3.08)	0.609	0.966
NXPH3	1.98 (1.03-3.80)	0.040	0.413	2.93 (1.04-8.29)	0.042	0.359	3.20 (1.18-8.66)	0.022	0.551
OBP2B	1.32 (0.94-1.88)	0.113	0.603	1.23 (0.66-2.31)	0.512	0.943	1.09 (0.57-2.08)	0.788	0.989
OCLN	1.48 (0.90-2.43)	0.127	0.625	1.39 (0.56-3.48)	0.479	0.932	2.70 (1.34-5.44)	0.006	0.346
ODAM	0.84 (0.54-1.33)	0.460	0.920	0.66 (0.29-1.53)	0.336	0.839	0.61 (0.26-1.43)	0.255	0.901
OFD1	1.39 (1.01-1.91)	0.042	0.420	1.36 (0.73-2.54)	0.335	0.839	0.63 (0.24-1.68)	0.358	0.939
OGA	0.89 (0.62-1.27)	0.511	0.945	1.20 (0.64-2.24)	0.575	0.965	0.70 (0.35-1.40)	0.310	0.923
OGFR	0.96 (0.48-1.95)	0.917	0.999	1.42 (0.43-4.67)	0.563	0.962	1.11 (0.32-3.88)	0.872	0.992
OGN	1.70 (1.11-2.62)	0.015	0.294	3.05 (1.61-5.78)	<0.001	0.059	1.99 (0.97-4.11)	0.062	0.698
OGT	1.22 (0.91-1.64)	0.186	0.711	1.10 (0.61-2.01)	0.746	0.997	0.93 (0.48-1.83)	0.837	0.991
OLFM4	1.26 (1.04-1.53)	0.018	0.307	1.34 (0.95-1.91)	0.097	0.520	1.39 (0.97-1.99)	0.074	0.718
OLR1	1.30 (0.91-1.86)	0.152	0.673	1.24 (0.63-2.43)	0.534	0.951	0.97 (0.47-2.00)	0.940	0.992
OMD	1.14 (0.70-1.87)	0.599	0.962	4.13 (1.51-11.29)	0.006	0.146	0.89 (0.39-2.05)	0.781	0.987
OMG	0.79 (0.57-1.09)	0.157	0.677	0.75 (0.42-1.35)	0.339	0.843	0.80 (0.45-1.44)	0.464	0.942
OMP	1.23 (0.78-1.96)	0.374	0.874	0.90 (0.33-2.42)	0.834	0.997	1.72 (0.86-3.46)	0.127	0.796
OPHN1	1.00 (0.79-1.26)	0.992	0.999	1.18 (0.78-1.80)	0.437	0.912	0.94 (0.61-1.45)	0.773	0.987
OPLAH	1.08 (0.76-1.55)	0.660	0.971	1.28 (0.68-2.42)	0.449	0.919	0.85 (0.42-1.72)	0.660	0.975
OPTC	1.57 (0.95-2.59)	0.076	0.514	2.33 (1.01-5.34)	0.047	0.373	2.63 (1.18-5.84)	0.018	0.512
ORM1	0.90 (0.24-3.35)	0.870	0.998	1.11 (0.10-12.08)	0.932	0.997	1.09 (0.09-13.16)	0.947	0.992

OSBPL2	1.50 (0.84-2.69)	0.171	0.697	2.18 (0.93-5.08)	0.073	0.460	1.63 (0.56-4.72)	0.370	0.939
OSCAR	1.35 (0.71-2.59)	0.363	0.866	2.70 (0.86-8.47)	0.087	0.493	1.04 (0.32-3.44)	0.943	0.992
OSM	1.15 (0.85-1.56)	0.353	0.857	0.78 (0.46-1.32)	0.352	0.860	1.34 (0.78-2.31)	0.291	0.915
OSMR	1.97 (0.69-5.66)	0.205	0.738	4.21 (0.72-24.44)	0.109	0.546	0.37 (0.05-3.02)	0.354	0.938
OSTN	0.70 (0.32-1.50)	0.356	0.859	0.55 (0.13-2.35)	0.417	0.903	0.91 (0.23-3.60)	0.889	0.992
OTOA	1.31 (0.93-1.86)	0.127	0.625	1.93 (1.31-2.84)	<0.001	0.063	1.00 (0.46-2.22)	0.991	0.998
OTUD6B	0.80 (0.52-1.23)	0.316	0.832	1.01 (0.47-2.18)	0.979	0.997	0.89 (0.40-1.96)	0.771	0.987
OTUD7B	0.94 (0.62-1.42)	0.764	0.987	1.06 (0.50-2.24)	0.883	0.997	1.06 (0.48-2.33)	0.883	0.992
OXCT1	1.01 (0.69-1.48)	0.944	0.999	0.94 (0.48-1.85)	0.856	0.997	1.02 (0.51-2.05)	0.952	0.992
OXT	0.92 (0.79-1.08)	0.334	0.849	1.01 (0.76-1.35)	0.952	0.997	0.99 (0.74-1.33)	0.939	0.992
P4HB	1.18 (0.67-2.09)	0.574	0.956	1.60 (0.56-4.56)	0.380	0.885	1.69 (0.58-4.98)	0.338	0.935
PACS2	0.98 (0.65-1.47)	0.922	0.999	1.63 (0.83-3.21)	0.157	0.633	0.73 (0.32-1.63)	0.438	0.942
PADI2	1.28 (1.00-1.65)	0.050	0.433	1.17 (0.66-2.07)	0.597	0.973	1.22 (0.73-2.04)	0.441	0.942
PADI4	1.12 (0.85-1.49)	0.410	0.894	0.85 (0.45-1.61)	0.616	0.979	1.12 (0.68-1.85)	0.643	0.973
PAEP	1.05 (0.88-1.26)	0.565	0.956	1.06 (0.77-1.46)	0.706	0.994	1.17 (0.84-1.64)	0.353	0.938
PAFAH1B3	1.11 (0.91-1.37)	0.305	0.823	1.23 (0.88-1.71)	0.229	0.739	1.06 (0.68-1.67)	0.791	0.989
PAFAH2	1.22 (0.71-2.08)	0.475	0.926	1.73 (0.67-4.46)	0.259	0.772	1.77 (0.66-4.79)	0.259	0.907
PAG1	0.78 (0.50-1.21)	0.268	0.797	0.54 (0.24-1.22)	0.136	0.600	0.54 (0.24-1.23)	0.145	0.808
PAGR1	0.83 (0.45-1.56)	0.571	0.956	1.64 (0.66-4.08)	0.288	0.800	0.57 (0.17-1.89)	0.361	0.939
PAIP2B	1.06 (0.66-1.70)	0.804	0.995	0.88 (0.34-2.31)	0.801	0.997	0.79 (0.28-2.22)	0.661	0.975
PAK4	1.03 (0.73-1.46)	0.865	0.998	1.09 (0.59-2.00)	0.783	0.997	1.03 (0.56-1.88)	0.936	0.992
PALLD	1.04 (0.53-2.05)	0.904	0.999	1.68 (0.72-3.94)	0.229	0.739	0.70 (0.16-3.18)	0.648	0.973
PALM	1.67 (0.98-2.84)	0.059	0.465	1.94 (0.88-4.29)	0.101	0.525	2.43 (1.22-4.85)	0.011	0.432
PALM2	2.09 (1.35-3.23)	<0.001	0.070	2.33 (1.16-4.67)	0.017	0.236	3.03 (1.66-5.54)	<0.001	0.157
PALM3	1.25 (0.94-1.67)	0.125	0.625	1.35 (0.86-2.13)	0.195	0.699	1.22 (0.64-2.29)	0.547	0.954

PAM	1.74 (0.73-4.13)	0.208	0.740	8.84 (1.84-42.51)	0.007	0.159	2.47 (0.50-12.15)	0.266	0.910
PAMR1	1.08 (0.50-2.32)	0.852	0.998	0.65 (0.16-2.63)	0.545	0.959	1.21 (0.31-4.82)	0.782	0.987
PAPPA	0.91 (0.55-1.51)	0.717	0.975	1.86 (0.76-4.57)	0.176	0.662	0.65 (0.25-1.70)	0.382	0.942
PARD3	1.07 (0.74-1.55)	0.720	0.975	0.93 (0.45-1.91)	0.842	0.997	1.32 (0.71-2.46)	0.374	0.940
PARK7	1.05 (0.68-1.63)	0.826	0.995	0.96 (0.44-2.12)	0.924	0.997	1.06 (0.48-2.34)	0.895	0.992
PARP1	1.13 (0.79-1.63)	0.491	0.936	1.24 (0.67-2.31)	0.496	0.940	0.80 (0.33-1.95)	0.631	0.973
PAXX	0.95 (0.53-1.70)	0.874	0.998	1.90 (0.78-4.64)	0.156	0.633	1.40 (0.55-3.57)	0.480	0.944
PBK	1.42 (0.63-3.22)	0.396	0.893	3.65 (0.84-15.92)	0.085	0.487	0.47 (0.11-1.94)	0.297	0.917
PBLD	0.87 (0.59-1.28)	0.471	0.924	0.77 (0.36-1.66)	0.502	0.941	1.09 (0.54-2.22)	0.804	0.991
PBXIP1	0.95 (0.52-1.71)	0.856	0.998	1.03 (0.35-3.02)	0.952	0.997	1.13 (0.40-3.22)	0.818	0.991
PCARE	1.04 (0.82-1.30)	0.762	0.987	1.18 (0.83-1.69)	0.360	0.862	0.75 (0.42-1.36)	0.344	0.936
PCBD1	0.74 (0.46-1.21)	0.236	0.767	1.19 (0.54-2.63)	0.666	0.993	0.81 (0.34-1.92)	0.637	0.973
PCBP2	0.96 (0.68-1.34)	0.797	0.994	1.51 (0.82-2.77)	0.183	0.668	0.57 (0.29-1.11)	0.096	0.762
PCDH1	0.42 (0.14-1.28)	0.126	0.625	0.33 (0.04-2.79)	0.310	0.816	1.03 (0.17-6.40)	0.970	0.994
PCDH12	1.86 (0.72-4.77)	0.200	0.727	5.99 (1.16-30.95)	0.033	0.314	2.60 (0.48-14.04)	0.266	0.910
PCDH17	1.81 (1.03-3.19)	0.041	0.416	4.91 (1.81-13.35)	0.002	0.085	1.11 (0.39-3.12)	0.844	0.992
PCDH7	2.10 (1.14-3.88)	0.018	0.304	2.25 (0.74-6.86)	0.155	0.631	3.87 (1.65-9.10)	0.002	0.228
PCDH9	2.77 (1.35-5.68)	0.005	0.178	4.08 (1.20-13.84)	0.024	0.271	1.47 (0.41-5.28)	0.559	0.955
PCDHB15	1.73 (1.01-2.94)	0.044	0.426	1.79 (0.76-4.20)	0.181	0.667	1.02 (0.37-2.83)	0.970	0.994
PCNA	1.23 (0.84-1.81)	0.293	0.811	1.69 (1.06-2.68)	0.026	0.283	0.42 (0.11-1.60)	0.203	0.867
PCSK7	0.94 (0.59-1.50)	0.795	0.994	0.85 (0.35-2.04)	0.712	0.995	0.89 (0.39-2.01)	0.774	0.987
PCSK9	1.53 (0.89-2.64)	0.126	0.625	0.86 (0.33-2.28)	0.765	0.997	3.31 (1.18-9.26)	0.022	0.551
PCYT2	0.84 (0.61-1.16)	0.285	0.806	1.27 (0.70-2.31)	0.424	0.905	0.69 (0.39-1.22)	0.199	0.867
PDAP1	0.94 (0.72-1.21)	0.623	0.966	1.18 (0.74-1.89)	0.479	0.932	0.84 (0.51-1.39)	0.501	0.947
PDCD1	1.11 (0.69-1.79)	0.661	0.971	1.75 (0.82-3.76)	0.149	0.624	0.43 (0.15-1.26)	0.124	0.794

PDCD1LG2	1.81 (0.93-3.51)	0.080	0.518	1.78 (0.52-6.09)	0.356	0.862	1.21 (0.35-4.15)	0.760	0.987
PDCD5	1.26 (0.87-1.84)	0.225	0.757	1.10 (0.57-2.15)	0.773	0.997	1.40 (0.72-2.73)	0.317	0.923
PDCD6	1.26 (0.85-1.88)	0.249	0.774	1.50 (0.68-3.35)	0.318	0.823	0.96 (0.53-1.74)	0.888	0.992
PDCL2	1.14 (0.68-1.91)	0.621	0.966	1.75 (0.79-3.85)	0.165	0.647	0.55 (0.16-1.92)	0.346	0.936
PDE1C	1.00 (0.64-1.54)	0.984	0.999	1.18 (0.63-2.18)	0.605	0.976	0.36 (0.08-1.67)	0.190	0.862
PDE4D	1.09 (0.78-1.53)	0.605	0.963	0.91 (0.46-1.79)	0.779	0.997	1.26 (0.76-2.08)	0.365	0.939
PDE5A	1.00 (0.78-1.29)	0.994	0.999	0.90 (0.58-1.41)	0.658	0.992	0.90 (0.57-1.42)	0.642	0.973
PDGFA	0.93 (0.68-1.26)	0.629	0.966	0.99 (0.56-1.76)	0.984	0.998	0.79 (0.45-1.39)	0.423	0.942
PDGFB	0.98 (0.76-1.28)	0.900	0.999	0.99 (0.61-1.60)	0.957	0.997	0.86 (0.53-1.39)	0.537	0.952
PDGFC	1.12 (0.47-2.68)	0.791	0.994	0.95 (0.19-4.77)	0.951	0.997	2.50 (0.55-11.48)	0.238	0.889
PDGFRA	1.66 (0.81-3.39)	0.169	0.697	3.09 (0.84-11.33)	0.088	0.494	1.04 (0.27-3.99)	0.952	0.992
PDGFRB	1.35 (0.69-2.66)	0.380	0.875	2.50 (0.70-8.91)	0.159	0.635	1.19 (0.36-3.96)	0.779	0.987
PDIA2	0.78 (0.39-1.55)	0.471	0.924	0.44 (0.12-1.56)	0.205	0.711	0.52 (0.15-1.79)	0.302	0.919
PDIA3	1.77 (0.72-4.40)	0.216	0.746	2.92 (0.83-10.23)	0.094	0.516	3.01 (0.67-13.46)	0.149	0.813
PDIA4	0.80 (0.52-1.24)	0.311	0.828	1.21 (0.58-2.55)	0.608	0.978	0.50 (0.21-1.21)	0.124	0.794
PDIA5	0.75 (0.47-1.21)	0.244	0.771	0.86 (0.37-1.97)	0.717	0.995	0.70 (0.29-1.67)	0.416	0.942
PDLIM5	0.98 (0.80-1.21)	0.876	0.998	1.29 (0.88-1.91)	0.197	0.702	0.77 (0.52-1.15)	0.199	0.867
PDLIM7	1.05 (0.88-1.25)	0.608	0.963	1.05 (0.75-1.45)	0.783	0.997	0.97 (0.68-1.37)	0.859	0.992
PDP1	1.12 (0.69-1.81)	0.640	0.969	0.23 (0.07-0.77)	0.017	0.236	0.71 (0.24-2.09)	0.529	0.950
PDRG1	1.01 (0.57-1.79)	0.982	0.999	1.03 (0.36-2.95)	0.953	0.997	0.56 (0.17-1.90)	0.355	0.939
PDXDC1	1.02 (0.64-1.64)	0.930	0.999	1.12 (0.51-2.49)	0.778	0.997	0.25 (0.05-1.30)	0.098	0.762
PDZD2	1.42 (1.02-1.97)	0.035	0.399	0.99 (0.47-2.05)	0.970	0.997	1.28 (0.70-2.35)	0.427	0.942
PDZK1	1.44 (0.91-2.30)	0.121	0.619	2.07 (1.05-4.09)	0.036	0.326	1.39 (0.62-3.12)	0.419	0.942
PEAR1	1.98 (0.75-5.20)	0.167	0.693	2.08 (0.36-12.07)	0.414	0.903	1.27 (0.20-7.90)	0.800	0.991
PEBP1	1.21 (0.82-1.77)	0.341	0.854	1.03 (0.51-2.06)	0.941	0.997	1.13 (0.57-2.25)	0.725	0.978

PECAM1	1.76 (0.85-3.63)	0.127	0.625	3.64 (1.03-12.81)	0.045	0.366	1.02 (0.25-4.20)	0.975	0.994
PECR	0.88 (0.39-2.00)	0.757	0.986	0.84 (0.18-3.88)	0.819	0.997	0.52 (0.08-3.28)	0.484	0.944
PENK	1.98 (1.34-2.93)	<0.001	0.055	2.02 (1.08-3.75)	0.027	0.286	2.56 (1.53-4.27)	<0.001	0.157
PEPD	1.14 (0.47-2.75)	0.770	0.988	4.12 (0.81-21.00)	0.088	0.494	2.43 (0.49-12.17)	0.279	0.910
PER3	0.99 (0.80-1.23)	0.920	0.999	1.12 (0.78-1.62)	0.537	0.953	0.72 (0.46-1.10)	0.128	0.796
PF4	0.87 (0.69-1.09)	0.236	0.767	1.09 (0.72-1.67)	0.675	0.993	0.78 (0.51-1.20)	0.258	0.907
PFDN2	1.01 (0.66-1.54)	0.979	0.999	1.05 (0.49-2.27)	0.892	0.997	1.04 (0.49-2.22)	0.923	0.992
PFDN4	1.28 (0.85-1.91)	0.233	0.763	0.84 (0.35-2.04)	0.705	0.994	1.42 (0.80-2.54)	0.234	0.884
PFDN6	0.70 (0.43-1.11)	0.130	0.629	0.48 (0.18-1.26)	0.136	0.600	0.68 (0.28-1.68)	0.408	0.942
PFKFB2	1.08 (0.81-1.46)	0.595	0.962	1.03 (0.60-1.78)	0.902	0.997	0.94 (0.55-1.63)	0.838	0.991
PGA4	1.20 (0.96-1.51)	0.117	0.608	1.58 (0.99-2.50)	0.055	0.393	1.56 (0.99-2.45)	0.057	0.698
PGD	0.84 (0.55-1.31)	0.449	0.916	1.33 (0.62-2.85)	0.464	0.926	0.80 (0.36-1.79)	0.591	0.965
PGF	1.50 (0.72-3.10)	0.278	0.804	2.56 (0.89-7.36)	0.081	0.483	2.48 (0.76-8.13)	0.134	0.796
PGLYRP1	1.49 (0.91-2.44)	0.116	0.607	1.45 (0.60-3.52)	0.413	0.903	0.80 (0.32-2.02)	0.642	0.973
PGLYRP2	1.25 (0.58-2.72)	0.572	0.956	9.24 (1.69-50.64)	0.010	0.195	0.23 (0.06-0.84)	0.027	0.588
PGLYRP4	1.07 (0.72-1.57)	0.746	0.984	1.64 (1.05-2.55)	0.028	0.295	0.38 (0.12-1.24)	0.110	0.763
PGM2	0.57 (0.31-1.05)	0.071	0.497	0.48 (0.15-1.55)	0.221	0.730	0.81 (0.33-1.95)	0.633	0.973
PGR	1.09 (0.86-1.38)	0.465	0.922	1.06 (0.67-1.66)	0.813	0.997	1.16 (0.75-1.81)	0.500	0.947
PHACTR2	0.99 (0.78-1.25)	0.918	0.999	1.16 (0.77-1.76)	0.485	0.934	0.75 (0.47-1.17)	0.202	0.867
PHLDB1	1.67 (1.10-2.54)	0.017	0.296	1.32 (0.61-2.84)	0.485	0.934	2.68 (1.37-5.27)	0.004	0.316
PHLDB2	1.37 (1.12-1.68)	0.002	0.107	1.13 (0.74-1.73)	0.569	0.963	1.19 (0.76-1.86)	0.441	0.942
PHOSPHO1	1.26 (0.61-2.64)	0.531	0.948	2.38 (0.62-9.08)	0.205	0.712	1.08 (0.30-3.83)	0.908	0.992
PHYKPL	0.74 (0.49-1.12)	0.151	0.673	1.15 (0.52-2.56)	0.730	0.997	0.61 (0.28-1.32)	0.208	0.867
PI16	1.08 (0.43-2.66)	0.875	0.998	1.78 (0.36-8.79)	0.482	0.934	1.35 (0.27-6.75)	0.719	0.976
PI3	1.30 (0.90-1.87)	0.167	0.693	1.41 (0.71-2.78)	0.329	0.832	0.70 (0.33-1.50)	0.363	0.939

PIBF1	0.83 (0.62-1.12)	0.235	0.767	0.74 (0.42-1.28)	0.283	0.794	0.62 (0.34-1.14)	0.123	0.791
PIGR	2.10 (1.21-3.66)	0.009	0.220	2.63 (0.94-7.36)	0.066	0.435	2.05 (0.73-5.76)	0.172	0.853
PIK3AP1	1.06 (0.76-1.49)	0.723	0.975	0.57 (0.30-1.10)	0.095	0.517	1.48 (0.85-2.58)	0.164	0.848
PIK3IP1	1.63 (0.89-3.01)	0.115	0.607	1.97 (0.75-5.23)	0.171	0.656	1.45 (0.49-4.35)	0.504	0.947
PIKFYVE	1.30 (0.82-2.04)	0.262	0.789	2.09 (0.99-4.40)	0.053	0.392	1.12 (0.44-2.81)	0.817	0.991
PILRA	2.10 (1.29-3.41)	0.003	0.121	3.76 (1.64-8.61)	0.002	0.084	1.01 (0.40-2.57)	0.985	0.996
PILRB	1.80 (1.18-2.73)	0.006	0.186	3.58 (1.56-8.20)	0.003	0.109	0.78 (0.38-1.61)	0.507	0.947
PINLYP	1.58 (1.08-2.32)	0.019	0.311	2.96 (1.36-6.44)	0.006	0.157	0.85 (0.44-1.62)	0.612	0.966
PITHD1	1.06 (0.74-1.50)	0.766	0.987	1.21 (0.70-2.10)	0.499	0.941	1.18 (0.70-1.99)	0.525	0.949
PKD1	2.93 (1.25-6.86)	0.013	0.278	7.32 (1.71-31.32)	0.007	0.164	3.21 (0.65-15.88)	0.153	0.816
PKD2	0.99 (0.65-1.52)	0.967	0.999	1.27 (0.64-2.55)	0.495	0.940	1.09 (0.50-2.40)	0.828	0.991
PKLR	1.19 (0.88-1.60)	0.265	0.792	0.77 (0.42-1.39)	0.383	0.886	1.36 (0.81-2.26)	0.244	0.893
PKN3	1.14 (0.93-1.40)	0.192	0.718	0.96 (0.61-1.50)	0.848	0.997	1.24 (0.87-1.78)	0.238	0.889
PLA2G10	1.26 (0.87-1.85)	0.226	0.757	1.46 (0.73-2.90)	0.284	0.795	1.61 (0.76-3.41)	0.212	0.867
PLA2G15	2.22 (1.09-4.54)	0.029	0.371	3.70 (1.26-10.87)	0.017	0.236	2.58 (0.71-9.34)	0.149	0.813
PLA2G1B	0.71 (0.46-1.11)	0.130	0.629	1.04 (0.48-2.25)	0.924	0.997	0.57 (0.27-1.21)	0.142	0.808
PLA2G2A	1.22 (0.91-1.64)	0.177	0.706	0.98 (0.51-1.89)	0.951	0.997	1.41 (0.92-2.16)	0.115	0.776
PLA2G4A	0.95 (0.73-1.24)	0.711	0.975	0.97 (0.60-1.58)	0.913	0.997	0.77 (0.47-1.27)	0.303	0.919
PLA2G7	0.94 (0.51-1.73)	0.852	0.998	0.81 (0.27-2.50)	0.720	0.995	0.82 (0.28-2.42)	0.719	0.976
PLAT	1.18 (0.78-1.77)	0.440	0.909	2.11 (1.00-4.46)	0.051	0.391	0.80 (0.39-1.66)	0.555	0.955
PLAU	1.59 (0.80-3.17)	0.186	0.711	3.57 (1.26-10.16)	0.017	0.236	1.35 (0.36-5.08)	0.653	0.975
PLAUR	2.18 (1.06-4.47)	0.034	0.389	2.07 (0.56-7.66)	0.278	0.788	2.83 (0.79-10.14)	0.110	0.763
PLB1	0.93 (0.61-1.41)	0.724	0.976	0.86 (0.41-1.83)	0.700	0.994	0.78 (0.36-1.72)	0.540	0.952
PLCB1	0.64 (0.38-1.11)	0.111	0.598	0.50 (0.16-1.56)	0.234	0.742	0.82 (0.34-1.94)	0.647	0.973
PLCB2	0.93 (0.73-1.19)	0.576	0.956	1.07 (0.68-1.68)	0.775	0.997	0.65 (0.40-1.06)	0.081	0.743

PLEKHO1	1.13 (0.79-1.62)	0.496	0.938	1.42 (0.75-2.70)	0.285	0.796	0.96 (0.47-1.94)	0.904	0.992
PLG	1.00 (0.30-3.35)	0.999	0.999	1.63 (0.19-14.14)	0.658	0.992	0.54 (0.06-4.61)	0.574	0.960
PLIN1	1.85 (1.09-3.14)	0.023	0.349	1.94 (0.78-4.81)	0.152	0.626	3.45 (1.58-7.54)	0.002	0.228
PLIN3	0.93 (0.53-1.66)	0.817	0.995	1.80 (0.69-4.72)	0.233	0.742	0.67 (0.22-1.99)	0.466	0.942
PLPBP	1.07 (0.76-1.51)	0.690	0.975	1.19 (0.64-2.22)	0.589	0.969	0.94 (0.50-1.78)	0.857	0.992
PLSCR3	1.01 (0.63-1.63)	0.961	0.999	1.01 (0.42-2.41)	0.987	0.998	1.26 (0.53-3.02)	0.604	0.966
PLTP	0.90 (0.51-1.57)	0.703	0.975	0.87 (0.32-2.39)	0.786	0.997	1.39 (0.47-4.05)	0.551	0.955
PLXDC1	0.74 (0.34-1.64)	0.460	0.920	1.19 (0.32-4.42)	0.800	0.997	1.66 (0.55-5.05)	0.369	0.939
PLXDC2	2.56 (1.31-4.98)	0.006	0.184	3.71 (1.50-9.20)	0.005	0.142	2.79 (0.95-8.16)	0.062	0.698
PLXNA4	1.02 (0.76-1.35)	0.911	0.999	1.01 (0.59-1.70)	0.983	0.998	0.98 (0.59-1.65)	0.947	0.992
PLXNB2	3.10 (1.52-6.33)	0.002	0.107	2.11 (0.59-7.54)	0.253	0.761	1.95 (0.50-7.56)	0.334	0.934
PLXNB3	1.08 (0.66-1.74)	0.768	0.987	1.10 (0.45-2.65)	0.839	0.997	0.83 (0.34-2.01)	0.673	0.975
PM20D1	1.02 (0.90-1.17)	0.718	0.975	1.11 (0.86-1.42)	0.425	0.905	1.02 (0.81-1.29)	0.855	0.992
PMCH	1.08 (0.86-1.35)	0.499	0.939	0.81 (0.45-1.47)	0.485	0.934	1.17 (0.81-1.69)	0.412	0.942
PMM2	0.91 (0.57-1.45)	0.687	0.975	1.37 (0.61-3.08)	0.447	0.918	0.53 (0.21-1.33)	0.176	0.853
PMS1	1.14 (0.68-1.90)	0.628	0.966	0.81 (0.27-2.37)	0.694	0.994	0.93 (0.35-2.45)	0.876	0.992
PMVK	1.02 (0.82-1.26)	0.888	0.999	1.02 (0.69-1.49)	0.935	0.997	0.86 (0.61-1.22)	0.395	0.942
PNLIP	0.84 (0.62-1.13)	0.244	0.771	0.94 (0.57-1.57)	0.826	0.997	1.06 (0.64-1.76)	0.821	0.991
PNLIPRP1	1.00 (0.73-1.36)	0.975	0.999	1.59 (0.96-2.65)	0.072	0.459	1.06 (0.62-1.83)	0.828	0.991
PNLIPRP2	1.01 (0.94-1.10)	0.743	0.984	1.03 (0.88-1.19)	0.743	0.997	0.89 (0.78-1.00)	0.052	0.680
PNMA1	0.91 (0.58-1.42)	0.667	0.971	0.85 (0.37-1.96)	0.702	0.994	0.73 (0.31-1.74)	0.478	0.944
PNMA2	0.70 (0.37-1.32)	0.272	0.799	1.08 (0.47-2.49)	0.853	0.997	0.95 (0.30-2.97)	0.928	0.992
PNPT1	0.89 (0.70-1.12)	0.327	0.843	1.03 (0.71-1.49)	0.874	0.997	0.46 (0.25-0.87)	0.016	0.490
PODXL	1.01 (0.27-3.79)	0.990	0.999	2.54 (0.29-21.88)	0.397	0.897	0.65 (0.06-6.67)	0.721	0.977
PODXL2	0.83 (0.39-1.77)	0.626	0.966	1.05 (0.27-4.11)	0.946	0.997	0.50 (0.12-1.98)	0.323	0.931

POF1B	1.71 (1.03-2.84)	0.039	0.413	2.06 (0.87-4.89)	0.102	0.527	1.40 (0.52-3.75)	0.505	0.947
POLR2A	1.07 (0.64-1.78)	0.802	0.995	0.62 (0.19-2.01)	0.425	0.905	1.24 (0.53-2.91)	0.614	0.966
POLR2F	1.24 (0.70-2.20)	0.471	0.924	1.50 (0.57-3.97)	0.412	0.903	1.08 (0.37-3.20)	0.885	0.992
POMC	0.89 (0.63-1.26)	0.521	0.947	1.30 (0.69-2.45)	0.416	0.903	0.81 (0.42-1.57)	0.540	0.952
PON1	0.53 (0.22-1.28)	0.158	0.678	1.27 (0.23-6.93)	0.780	0.997	0.29 (0.07-1.28)	0.103	0.763
PON2	1.15 (0.64-2.06)	0.632	0.968	0.76 (0.26-2.25)	0.620	0.980	1.75 (0.64-4.84)	0.278	0.910
PON3	0.65 (0.36-1.18)	0.157	0.676	0.75 (0.22-2.59)	0.651	0.992	0.73 (0.25-2.15)	0.563	0.957
POSTN	1.10 (0.60-2.02)	0.746	0.984	2.79 (1.05-7.43)	0.040	0.347	1.16 (0.40-3.38)	0.791	0.989
PPBP	0.85 (0.63-1.14)	0.282	0.806	1.00 (0.57-1.75)	0.990	0.998	0.62 (0.36-1.09)	0.099	0.762
PPCDC	0.94 (0.58-1.52)	0.806	0.995	0.72 (0.30-1.74)	0.468	0.926	1.46 (0.61-3.47)	0.395	0.942
PPIB	0.90 (0.70-1.15)	0.405	0.894	0.89 (0.57-1.41)	0.626	0.983	0.81 (0.51-1.30)	0.387	0.942
PPIE	0.94 (0.61-1.44)	0.766	0.987	1.31 (0.74-2.32)	0.348	0.855	1.04 (0.49-2.21)	0.925	0.992
PPIF	0.91 (0.59-1.42)	0.688	0.975	0.96 (0.44-2.11)	0.921	0.997	0.66 (0.26-1.67)	0.381	0.942
PPL	1.00 (0.48-2.09)	0.994	0.999	1.05 (0.29-3.87)	0.941	0.997	2.49 (0.87-7.12)	0.088	0.745
PPM1A	0.59 (0.26-1.31)	0.195	0.720	0.49 (0.11-2.25)	0.361	0.862	0.20 (0.04-1.03)	0.054	0.692
PPM1B	1.05 (0.68-1.63)	0.816	0.995	0.54 (0.16-1.79)	0.313	0.818	1.33 (0.65-2.69)	0.433	0.942
PPM1F	0.78 (0.47-1.28)	0.320	0.836	1.38 (0.58-3.28)	0.473	0.932	0.50 (0.19-1.34)	0.166	0.848
PPME1	1.07 (0.75-1.52)	0.726	0.977	0.68 (0.36-1.26)	0.218	0.729	1.26 (0.69-2.32)	0.450	0.942
PPP1CC	0.88 (0.64-1.20)	0.413	0.894	1.01 (0.57-1.79)	0.963	0.997	0.70 (0.38-1.30)	0.256	0.903
PPP1R12A	0.96 (0.76-1.22)	0.757	0.986	1.10 (0.71-1.70)	0.672	0.993	0.82 (0.51-1.30)	0.390	0.942
PPP1R12B	1.21 (0.87-1.69)	0.258	0.786	1.51 (0.90-2.52)	0.119	0.570	0.36 (0.13-1.04)	0.060	0.698
PPP1R14A	0.93 (0.66-1.31)	0.697	0.975	1.11 (0.63-1.96)	0.706	0.994	0.62 (0.31-1.25)	0.178	0.853
PPP1R14D	1.27 (0.74-2.18)	0.379	0.875	0.91 (0.29-2.91)	0.876	0.997	2.19 (1.14-4.22)	0.019	0.519
PPP1R2	0.99 (0.74-1.33)	0.952	0.999	1.14 (0.67-1.95)	0.628	0.984	0.83 (0.47-1.49)	0.535	0.952
PPP1R9B	1.04 (0.83-1.32)	0.716	0.975	1.05 (0.68-1.60)	0.835	0.997	1.15 (0.74-1.79)	0.528	0.949

PPP2R5A	1.00 (0.81-1.23)	0.980	0.999	1.08 (0.74-1.57)	0.692	0.994	0.87 (0.59-1.30)	0.500	0.947
PPP3R1	0.93 (0.54-1.57)	0.774	0.991	1.70 (0.63-4.56)	0.296	0.803	1.00 (0.37-2.70)	0.999	1.000
PPT1	1.32 (1.00-1.73)	0.052	0.443	0.99 (0.52-1.89)	0.977	0.997	1.36 (0.81-2.30)	0.249	0.901
PPY	0.99 (0.80-1.22)	0.917	0.999	1.19 (0.83-1.71)	0.344	0.852	0.89 (0.60-1.31)	0.545	0.952
PQBP1	0.93 (0.61-1.42)	0.741	0.983	0.87 (0.38-1.99)	0.737	0.997	0.72 (0.27-1.92)	0.515	0.947
PRAME	0.78 (0.30-2.08)	0.624	0.966	2.05 (0.36-11.68)	0.418	0.903	0.21 (0.05-0.96)	0.043	0.637
PRAP1	1.03 (0.60-1.77)	0.907	0.999	2.03 (0.74-5.56)	0.168	0.649	0.99 (0.37-2.62)	0.985	0.996
PRC1	0.54 (0.22-1.30)	0.168	0.693	0.62 (0.14-2.75)	0.525	0.948	0.88 (0.27-2.89)	0.838	0.991
PRCP	2.01 (1.17-3.46)	0.011	0.250	1.47 (0.52-4.12)	0.468	0.926	1.63 (0.58-4.63)	0.357	0.939
PRDX1	0.77 (0.59-1.00)	0.051	0.442	0.82 (0.52-1.31)	0.411	0.903	0.67 (0.42-1.07)	0.094	0.762
PRDX2	0.98 (0.66-1.44)	0.902	0.999	0.77 (0.38-1.56)	0.468	0.926	1.23 (0.66-2.32)	0.514	0.947
PRDX3	1.04 (0.75-1.43)	0.821	0.995	1.00 (0.56-1.80)	0.990	0.998	0.89 (0.49-1.63)	0.707	0.976
PRDX5	1.02 (0.72-1.44)	0.911	0.999	0.96 (0.53-1.77)	0.904	0.997	1.07 (0.55-2.08)	0.853	0.992
PRDX6	0.88 (0.61-1.28)	0.507	0.944	0.71 (0.36-1.41)	0.329	0.832	1.14 (0.60-2.18)	0.692	0.975
PREB	1.26 (0.64-2.51)	0.505	0.943	2.08 (0.70-6.15)	0.187	0.679	1.71 (0.52-5.58)	0.374	0.940
PRELP	2.49 (0.98-6.36)	0.056	0.460	3.97 (0.76-20.79)	0.103	0.527	4.19 (0.87-20.18)	0.074	0.718
PRG2	1.24 (0.78-1.98)	0.371	0.872	2.28 (1.05-4.94)	0.037	0.331	0.95 (0.38-2.35)	0.907	0.992
PRG3	1.01 (0.59-1.72)	0.985	0.999	1.93 (0.76-4.86)	0.165	0.647	0.82 (0.30-2.23)	0.693	0.975
PRKAB1	1.04 (0.67-1.61)	0.873	0.998	1.30 (0.60-2.83)	0.505	0.942	0.92 (0.40-2.16)	0.856	0.992
PRKAG3	0.96 (0.46-2.00)	0.911	0.999	0.64 (0.13-3.02)	0.570	0.963	0.34 (0.06-1.86)	0.214	0.867
PRKAR1A	0.92 (0.70-1.20)	0.532	0.948	0.95 (0.59-1.54)	0.845	0.997	0.76 (0.46-1.27)	0.298	0.917
PRKAR2A	0.96 (0.71-1.31)	0.813	0.995	1.06 (0.60-1.87)	0.842	0.997	0.82 (0.45-1.49)	0.509	0.947
PRKCQ	0.88 (0.50-1.53)	0.644	0.971	1.03 (0.39-2.73)	0.956	0.997	1.09 (0.43-2.76)	0.856	0.992
PRKD2	0.69 (0.31-1.53)	0.354	0.859	1.04 (0.31-3.56)	0.944	0.997	0.64 (0.13-3.12)	0.580	0.964
PRKG1	1.07 (0.88-1.30)	0.472	0.924	1.06 (0.74-1.51)	0.767	0.997	0.89 (0.62-1.27)	0.527	0.949

PRKRA	1.05 (0.76-1.45)	0.766	0.987	0.82 (0.44-1.51)	0.520	0.946	1.18 (0.65-2.14)	0.590	0.965
PRL	1.17 (0.89-1.54)	0.266	0.794	1.21 (0.74-1.98)	0.437	0.911	1.58 (0.95-2.64)	0.076	0.719
PRND	1.56 (1.11-2.21)	0.011	0.248	1.95 (1.05-3.62)	0.033	0.318	2.04 (1.19-3.51)	0.010	0.413
PROC	1.17 (0.52-2.62)	0.703	0.975	0.73 (0.17-3.02)	0.660	0.992	1.08 (0.24-4.80)	0.921	0.992
PROCR	1.99 (1.10-3.60)	0.022	0.344	2.14 (0.74-6.18)	0.160	0.638	0.85 (0.17-4.40)	0.851	0.992
PROK1	0.88 (0.60-1.30)	0.520	0.947	0.64 (0.31-1.31)	0.221	0.730	1.21 (0.59-2.48)	0.600	0.966
PROS1	0.52 (0.21-1.28)	0.155	0.676	0.25 (0.06-1.06)	0.059	0.410	0.35 (0.08-1.55)	0.166	0.848
PRR4	0.99 (0.69-1.42)	0.939	0.999	0.59 (0.28-1.22)	0.151	0.626	1.27 (0.69-2.34)	0.444	0.942
PRR5	1.28 (0.78-2.10)	0.329	0.846	2.40 (1.32-4.36)	0.004	0.129	0.40 (0.12-1.34)	0.136	0.798
PRRT3	0.70 (0.39-1.25)	0.232	0.763	0.73 (0.26-2.05)	0.551	0.960	0.84 (0.29-2.41)	0.739	0.983
PRSS2	1.00 (0.70-1.44)	0.981	0.999	1.28 (0.70-2.34)	0.418	0.903	0.98 (0.52-1.83)	0.946	0.992
PRSS22	1.56 (0.93-2.63)	0.093	0.559	3.84 (2.22-6.64)	<0.001	0.002	0.94 (0.25-3.55)	0.922	0.992
PRSS27	1.29 (0.76-2.20)	0.345	0.855	1.83 (0.70-4.78)	0.219	0.729	0.62 (0.22-1.75)	0.363	0.939
PRSS53	1.29 (0.69-2.41)	0.433	0.906	0.94 (0.30-2.96)	0.913	0.997	1.43 (0.47-4.34)	0.523	0.948
PRSS8	1.57 (0.97-2.53)	0.066	0.474	1.39 (0.57-3.37)	0.465	0.926	1.75 (0.78-3.93)	0.175	0.853
PRTFDC1	0.96 (0.78-1.18)	0.678	0.974	0.94 (0.65-1.36)	0.756	0.997	0.97 (0.66-1.43)	0.887	0.992
PRTG	2.32 (0.99-5.41)	0.052	0.443	6.59 (1.68-25.87)	0.007	0.160	3.02 (0.68-13.40)	0.146	0.808
PRTN3	1.15 (0.75-1.77)	0.511	0.945	0.83 (0.35-1.96)	0.673	0.993	1.25 (0.60-2.57)	0.553	0.955
PRUNE2	0.97 (0.40-2.35)	0.947	0.999	0.86 (0.16-4.71)	0.864	0.997	1.24 (0.30-5.11)	0.766	0.987
PSAP	1.09 (0.49-2.44)	0.825	0.995	4.14 (1.04-16.52)	0.044	0.363	0.85 (0.20-3.61)	0.820	0.991
PSAPL1	1.22 (0.77-1.93)	0.405	0.894	2.49 (1.19-5.23)	0.016	0.232	1.38 (0.62-3.07)	0.432	0.942
PSCA	0.99 (0.90-1.09)	0.876	0.998	0.98 (0.82-1.17)	0.798	0.997	1.08 (0.89-1.32)	0.421	0.942
PSG1	1.52 (1.18-1.96)	0.001	0.075	2.40 (1.42-4.05)	0.001	0.066	1.80 (1.11-2.91)	0.017	0.510
PSIP1	1.09 (0.78-1.53)	0.616	0.966	1.06 (0.55-2.03)	0.865	0.997	1.15 (0.64-2.06)	0.645	0.973
PSMA1	1.26 (0.82-1.93)	0.291	0.809	0.91 (0.35-2.34)	0.845	0.997	1.05 (0.45-2.43)	0.918	0.992

PSMC3	1.07 (0.50-2.28)	0.866	0.998	2.44 (0.85-6.99)	0.096	0.520	0.19 (0.04-0.91)	0.037	0.623
PSMD1	0.98 (0.61-1.57)	0.931	0.999	1.36 (0.63-2.93)	0.433	0.908	0.99 (0.43-2.24)	0.974	0.994
PSMD5	1.04 (0.62-1.75)	0.884	0.999	1.01 (0.39-2.63)	0.989	0.998	0.59 (0.17-2.04)	0.403	0.942
PSMD9	0.99 (0.69-1.42)	0.969	0.999	0.71 (0.33-1.50)	0.371	0.875	0.90 (0.48-1.71)	0.753	0.985
PSME1	1.02 (0.55-1.90)	0.950	0.999	0.89 (0.29-2.71)	0.835	0.997	1.52 (0.54-4.30)	0.426	0.942
PSME2	1.15 (0.62-2.13)	0.655	0.971	1.08 (0.37-3.19)	0.886	0.997	1.64 (0.59-4.55)	0.340	0.935
PSMG3	1.42 (0.95-2.11)	0.088	0.545	0.92 (0.43-1.94)	0.823	0.997	1.56 (0.78-3.11)	0.206	0.867
PSMG4	0.96 (0.62-1.47)	0.835	0.997	1.04 (0.51-2.13)	0.908	0.997	1.20 (0.63-2.31)	0.576	0.961
PSPN	0.91 (0.74-1.13)	0.409	0.894	0.92 (0.62-1.36)	0.681	0.993	0.85 (0.56-1.27)	0.425	0.942
PSRC1	0.81 (0.53-1.23)	0.325	0.843	0.48 (0.20-1.17)	0.105	0.532	1.02 (0.49-2.11)	0.964	0.994
PSTPIP2	1.05 (0.83-1.33)	0.693	0.975	1.04 (0.68-1.60)	0.846	0.997	0.90 (0.59-1.38)	0.622	0.967
PTEN	0.96 (0.44-2.10)	0.923	0.999	1.29 (0.30-5.50)	0.733	0.997	0.69 (0.17-2.76)	0.603	0.966
PTGDS	2.03 (1.16-3.55)	0.014	0.280	2.39 (0.93-6.12)	0.070	0.455	1.15 (0.40-3.36)	0.795	0.989
PTGES2	0.78 (0.50-1.22)	0.273	0.799	1.07 (0.48-2.38)	0.863	0.997	0.56 (0.24-1.32)	0.184	0.861
PTGR1	0.89 (0.66-1.20)	0.457	0.920	1.35 (0.81-2.25)	0.243	0.745	0.82 (0.47-1.41)	0.465	0.942
PTH	1.10 (0.85-1.43)	0.457	0.920	1.16 (0.73-1.87)	0.526	0.948	1.29 (0.82-2.01)	0.269	0.910
PTH1R	1.19 (0.70-2.03)	0.514	0.946	0.98 (0.35-2.74)	0.976	0.997	1.26 (0.55-2.92)	0.582	0.964
PTK7	2.73 (1.58-4.72)	<0.001	0.046	2.23 (0.82-6.05)	0.115	0.563	5.13 (2.05-12.86)	<0.001	0.174
PTN	1.01 (0.65-1.59)	0.950	0.999	1.54 (0.70-3.37)	0.281	0.793	0.56 (0.24-1.30)	0.178	0.853
PTP4A3	0.80 (0.46-1.39)	0.425	0.902	0.68 (0.24-1.94)	0.472	0.932	0.71 (0.25-2.01)	0.523	0.948
PTPN1	0.97 (0.72-1.29)	0.823	0.995	0.95 (0.56-1.61)	0.840	0.997	0.92 (0.52-1.61)	0.763	0.987
PTPN6	0.95 (0.74-1.23)	0.702	0.975	0.87 (0.56-1.35)	0.533	0.951	0.88 (0.56-1.40)	0.593	0.965
PTPN9	1.11 (0.57-2.16)	0.762	0.987	1.45 (0.44-4.76)	0.543	0.957	0.75 (0.21-2.69)	0.658	0.975
PTPRB	0.91 (0.41-2.00)	0.816	0.995	1.42 (0.36-5.65)	0.618	0.979	0.53 (0.13-2.22)	0.385	0.942
PTPRC	1.95 (0.71-5.30)	0.193	0.719	7.97 (1.84-34.42)	0.005	0.146	0.37 (0.05-2.74)	0.327	0.932

PTPRF	0.46 (0.23-0.95)	0.037	0.403	1.00 (0.25-4.00)	0.998	0.999	0.43 (0.11-1.69)	0.228	0.878
PTPRH	1.50 (0.95-2.37)	0.080	0.517	4.39 (1.94-9.93)	<0.001	0.048	1.45 (0.63-3.33)	0.381	0.942
PTPRK	0.76 (0.20-2.83)	0.681	0.975	0.99 (0.10-9.43)	0.993	0.998	0.50 (0.04-6.32)	0.594	0.965
PTPRM	2.10 (0.87-5.05)	0.097	0.570	2.53 (0.52-12.37)	0.251	0.758	1.25 (0.25-6.18)	0.782	0.987
PTPRN2	0.90 (0.50-1.61)	0.714	0.975	0.75 (0.25-2.22)	0.607	0.976	1.15 (0.42-3.15)	0.779	0.987
PTPRR	0.98 (0.59-1.64)	0.951	0.999	0.74 (0.27-2.03)	0.560	0.962	1.94 (0.93-4.09)	0.079	0.737
PTPRS	1.41 (0.62-3.23)	0.415	0.895	6.46 (1.31-31.89)	0.022	0.264	0.54 (0.13-2.30)	0.405	0.942
PTPRZ1	2.31 (1.11-4.78)	0.025	0.352	1.88 (0.49-7.23)	0.356	0.862	2.55 (0.64-10.15)	0.185	0.861
PTRHD1	0.80 (0.54-1.18)	0.262	0.789	1.17 (0.57-2.41)	0.673	0.993	0.53 (0.26-1.12)	0.097	0.762
PTS	0.85 (0.58-1.24)	0.402	0.894	0.73 (0.36-1.46)	0.370	0.874	1.15 (0.60-2.20)	0.673	0.975
PTTG1	1.01 (0.54-1.88)	0.978	0.999	1.00 (0.33-3.02)	0.994	0.998	1.26 (0.42-3.75)	0.683	0.975
PTX3	1.45 (0.86-2.43)	0.163	0.683	0.85 (0.37-1.99)	0.714	0.995	2.63 (0.90-7.64)	0.076	0.718
PVALB	0.99 (0.81-1.21)	0.926	0.999	1.11 (0.76-1.63)	0.594	0.972	1.04 (0.71-1.52)	0.829	0.991
PVR	1.36 (0.81-2.27)	0.241	0.769	1.17 (0.44-3.13)	0.758	0.997	2.20 (0.90-5.34)	0.083	0.743
PXDNL	1.20 (0.75-1.90)	0.444	0.913	1.55 (0.80-3.02)	0.195	0.697	1.14 (0.44-2.91)	0.790	0.989
PXN	1.41 (0.97-2.05)	0.075	0.512	1.32 (0.63-2.76)	0.465	0.926	1.51 (0.81-2.81)	0.195	0.866
PYDC1	1.59 (0.94-2.69)	0.085	0.535	1.86 (0.85-4.06)	0.121	0.575	1.39 (0.49-3.96)	0.542	0.952
PYY	1.24 (0.99-1.56)	0.060	0.466	1.18 (0.79-1.76)	0.411	0.903	1.35 (0.91-2.00)	0.132	0.796
PZP	1.18 (0.77-1.81)	0.436	0.907	0.94 (0.42-2.08)	0.878	0.997	1.36 (0.63-2.95)	0.434	0.942
QDPR	0.90 (0.52-1.55)	0.702	0.975	1.09 (0.41-2.91)	0.857	0.997	0.73 (0.27-1.98)	0.530	0.951
QPCT	2.61 (1.19-5.70)	0.016	0.294	2.41 (0.56-10.30)	0.236	0.742	2.07 (0.46-9.35)	0.345	0.936
QSOX1	1.66 (1.06-2.59)	0.026	0.352	2.37 (1.43-3.92)	<0.001	0.063	1.06 (0.33-3.42)	0.922	0.992
RAB10	0.82 (0.48-1.39)	0.462	0.920	1.09 (0.43-2.73)	0.858	0.997	0.59 (0.21-1.65)	0.316	0.923
RAB11FIP3	1.04 (0.83-1.31)	0.709	0.975	1.14 (0.76-1.72)	0.523	0.947	0.80 (0.53-1.22)	0.303	0.921
RAB27B	0.91 (0.67-1.24)	0.555	0.950	0.95 (0.54-1.68)	0.853	0.997	0.64 (0.37-1.11)	0.111	0.764

RAB2B	0.95 (0.61-1.47)	0.807	0.995	1.24 (0.57-2.67)	0.585	0.967	0.62 (0.26-1.49)	0.290	0.915
RAB33A	0.85 (0.55-1.29)	0.441	0.910	0.90 (0.41-1.97)	0.796	0.997	0.53 (0.23-1.25)	0.149	0.813
RAB37	0.84 (0.61-1.15)	0.277	0.804	1.21 (0.80-1.85)	0.366	0.869	0.42 (0.19-0.93)	0.033	0.613
RAB39B	1.03 (0.79-1.35)	0.809	0.995	1.25 (0.90-1.74)	0.185	0.676	0.57 (0.21-1.55)	0.271	0.910
RAB3GAP1	0.87 (0.53-1.43)	0.578	0.956	0.84 (0.34-2.08)	0.699	0.994	0.89 (0.37-2.14)	0.792	0.989
RAB44	1.21 (0.74-1.97)	0.455	0.920	1.95 (1.17-3.24)	0.010	0.195	0.62 (0.15-2.55)	0.504	0.947
RAB6A	0.76 (0.47-1.23)	0.262	0.789	0.88 (0.36-2.17)	0.785	0.997	1.21 (0.51-2.89)	0.664	0.975
RAB6B	1.03 (0.68-1.54)	0.906	0.999	0.74 (0.30-1.85)	0.523	0.947	1.19 (0.68-2.08)	0.533	0.951
RABEP1	0.93 (0.68-1.26)	0.618	0.966	0.87 (0.49-1.54)	0.637	0.987	0.97 (0.55-1.71)	0.911	0.992
RABEPK	0.67 (0.40-1.11)	0.120	0.616	0.56 (0.21-1.48)	0.245	0.750	0.62 (0.23-1.66)	0.345	0.936
RABGAP1L	1.11 (0.77-1.59)	0.573	0.956	1.04 (0.55-1.98)	0.900	0.997	1.31 (0.69-2.48)	0.410	0.942
RAC3	1.27 (0.95-1.69)	0.107	0.592	1.48 (0.96-2.28)	0.075	0.466	1.52 (0.93-2.48)	0.096	0.762
RAD23B	0.82 (0.49-1.38)	0.466	0.922	0.51 (0.19-1.37)	0.181	0.667	0.67 (0.25-1.80)	0.424	0.942
RAD51	1.16 (0.72-1.88)	0.547	0.949	1.04 (0.42-2.60)	0.926	0.997	1.49 (0.68-3.29)	0.320	0.926
RALB	0.83 (0.35-1.94)	0.665	0.971	1.24 (0.27-5.70)	0.784	0.997	0.61 (0.14-2.61)	0.502	0.947
RALY	0.83 (0.54-1.26)	0.381	0.875	1.03 (0.51-2.08)	0.941	0.997	0.98 (0.49-1.94)	0.945	0.992
RANBP1	1.03 (0.64-1.67)	0.890	0.999	1.09 (0.48-2.50)	0.831	0.997	1.33 (0.60-2.91)	0.481	0.944
RANBP2	1.26 (0.61-2.57)	0.532	0.948	1.18 (0.32-4.37)	0.801	0.997	0.87 (0.20-3.75)	0.856	0.992
RANGAP1	0.69 (0.38-1.23)	0.207	0.740	0.38 (0.12-1.22)	0.105	0.530	0.41 (0.13-1.35)	0.145	0.808
RAP1A	1.42 (1.12-1.81)	0.004	0.141	1.06 (0.54-2.06)	0.864	0.997	1.42 (0.86-2.35)	0.171	0.853
RAPGEF2	0.86 (0.53-1.38)	0.524	0.948	1.36 (0.69-2.68)	0.382	0.886	0.70 (0.28-1.78)	0.456	0.942
RARRES1	1.44 (0.84-2.48)	0.188	0.713	1.32 (0.43-4.04)	0.626	0.983	0.90 (0.22-3.66)	0.877	0.992
RARRES2	1.47 (0.95-2.28)	0.085	0.535	1.38 (0.62-3.08)	0.431	0.908	1.30 (0.57-2.94)	0.532	0.951
RASA1	1.72 (0.81-3.64)	0.158	0.678	1.44 (0.35-5.85)	0.611	0.979	4.89 (1.17-20.44)	0.030	0.595
RASGRF1	0.96 (0.53-1.72)	0.884	0.999	0.72 (0.24-2.20)	0.568	0.963	0.45 (0.16-1.26)	0.130	0.796

RASSF2	1.10 (0.76-1.59)	0.628	0.966	0.60 (0.25-1.42)	0.243	0.744	1.00 (0.49-2.04)	0.999	1.000
RBFOX3	1.90 (1.28-2.81)	0.001	0.085	2.66 (1.54-4.57)	<0.001	0.050	2.32 (1.26-4.26)	0.007	0.359
RBKS	0.95 (0.63-1.46)	0.829	0.997	0.95 (0.45-2.00)	0.889	0.997	1.47 (0.73-2.96)	0.279	0.910
RBM17	0.97 (0.71-1.32)	0.861	0.998	1.16 (0.66-2.03)	0.615	0.979	0.77 (0.42-1.41)	0.396	0.942
RBM19	1.12 (0.61-2.07)	0.717	0.975	2.03 (0.95-4.34)	0.068	0.447	0.82 (0.22-3.07)	0.768	0.987
RBM25	1.04 (0.67-1.59)	0.872	0.998	0.82 (0.37-1.83)	0.626	0.983	1.00 (0.50-2.02)	0.989	0.998
RBP1	1.12 (0.80-1.56)	0.508	0.944	0.90 (0.44-1.85)	0.771	0.997	1.19 (0.66-2.14)	0.558	0.955
RBP2	1.20 (0.91-1.58)	0.191	0.718	1.05 (0.64-1.74)	0.844	0.997	1.81 (1.14-2.88)	0.011	0.432
RBP5	1.02 (0.71-1.47)	0.918	0.999	1.07 (0.55-2.06)	0.840	0.997	1.06 (0.55-2.04)	0.853	0.992
RBP7	1.22 (0.85-1.74)	0.283	0.806	1.73 (0.98-3.03)	0.058	0.405	0.98 (0.50-1.93)	0.964	0.994
RBPMS	0.96 (0.64-1.46)	0.864	0.998	0.97 (0.45-2.06)	0.934	0.997	0.63 (0.19-2.08)	0.453	0.942
RBPMS2	1.03 (0.81-1.29)	0.822	0.995	0.94 (0.62-1.45)	0.791	0.997	1.01 (0.65-1.58)	0.964	0.994
RCC1	1.07 (0.80-1.44)	0.651	0.971	1.15 (0.68-1.93)	0.609	0.978	1.14 (0.68-1.90)	0.627	0.970
RCOR1	1.00 (0.53-1.88)	0.995	0.999	1.11 (0.36-3.43)	0.862	0.997	0.61 (0.14-2.55)	0.496	0.947
RECK	4.96 (1.47-16.73)	0.010	0.232	21.02 (2.50-177.06)	0.005	0.146	10.71 (1.06-108.62)	0.045	0.638
REEP4	0.86 (0.55-1.34)	0.495	0.938	0.79 (0.34-1.84)	0.579	0.967	0.67 (0.27-1.67)	0.396	0.942
REG1A	1.49 (1.04-2.13)	0.029	0.371	1.10 (0.53-2.28)	0.804	0.997	1.32 (0.68-2.58)	0.410	0.942
REG1B	1.38 (1.00-1.90)	0.047	0.428	1.03 (0.52-2.03)	0.925	0.997	1.10 (0.57-2.09)	0.782	0.987
REG3A	1.62 (1.21-2.15)	<0.001	0.074	1.79 (1.07-3.00)	0.025	0.279	1.26 (0.71-2.25)	0.425	0.942
REG3G	1.76 (0.97-3.19)	0.063	0.472	1.94 (0.67-5.62)	0.222	0.733	0.74 (0.23-2.33)	0.608	0.966
REG4	1.37 (0.94-1.99)	0.102	0.576	1.58 (0.83-2.98)	0.162	0.639	2.24 (1.18-4.23)	0.013	0.437
RELB	1.00 (0.58-1.73)	0.997	0.999	1.31 (0.61-2.82)	0.484	0.934	0.34 (0.08-1.49)	0.153	0.816
RELT	1.82 (1.03-3.21)	0.039	0.413	3.18 (1.41-7.20)	0.005	0.146	1.30 (0.43-3.89)	0.644	0.973
REN	1.02 (0.80-1.29)	0.901	0.999	1.40 (0.96-2.05)	0.078	0.471	0.98 (0.65-1.50)	0.936	0.992
REPS1	0.98 (0.49-1.97)	0.964	0.999	1.87 (0.91-3.84)	0.089	0.495	0.38 (0.08-1.73)	0.211	0.867

REST	1.43 (0.93-2.21)	0.107	0.592	1.71 (0.97-3.01)	0.066	0.435	0.35 (0.09-1.36)	0.129	0.796
RET	0.87 (0.51-1.47)	0.605	0.963	1.80 (0.68-4.78)	0.239	0.742	1.40 (0.50-3.87)	0.521	0.948
RETN	1.61 (1.00-2.60)	0.049	0.430	1.44 (0.60-3.43)	0.411	0.903	1.97 (0.86-4.52)	0.108	0.763
REXO2	1.27 (0.78-2.07)	0.332	0.847	1.38 (0.59-3.21)	0.461	0.924	1.18 (0.48-2.94)	0.716	0.976
RFC4	0.44 (0.12-1.59)	0.210	0.741	3.36 (0.83-13.58)	0.088	0.494	0.13 (0.01-1.48)	0.100	0.762
RGCC	0.93 (0.66-1.30)	0.670	0.971	1.14 (0.62-2.09)	0.674	0.993	0.60 (0.32-1.14)	0.118	0.780
RGL2	1.11 (0.71-1.72)	0.648	0.971	0.75 (0.29-1.89)	0.535	0.951	0.70 (0.27-1.78)	0.451	0.942
RGMA	1.12 (0.59-2.12)	0.738	0.982	1.91 (0.61-5.94)	0.263	0.773	1.31 (0.40-4.25)	0.655	0.975
RGMB	1.49 (0.75-2.96)	0.260	0.787	2.79 (1.02-7.68)	0.047	0.373	1.41 (0.39-5.01)	0.600	0.966
RGS10	0.83 (0.52-1.32)	0.424	0.902	1.18 (0.52-2.66)	0.690	0.994	0.48 (0.19-1.24)	0.132	0.796
RGS8	0.83 (0.56-1.24)	0.367	0.869	0.83 (0.40-1.72)	0.621	0.980	0.88 (0.45-1.72)	0.716	0.976
RHOC	0.94 (0.77-1.14)	0.537	0.948	0.99 (0.69-1.41)	0.942	0.997	0.79 (0.56-1.12)	0.184	0.861
RICTOR	0.58 (0.27-1.23)	0.153	0.676	1.02 (0.35-2.98)	0.977	0.997	0.70 (0.18-2.70)	0.600	0.966
RIDA	0.96 (0.63-1.45)	0.835	0.997	0.90 (0.43-1.89)	0.779	0.997	1.40 (0.70-2.81)	0.343	0.936
RILP	1.14 (0.79-1.64)	0.489	0.936	0.90 (0.47-1.74)	0.756	0.997	1.33 (0.70-2.52)	0.383	0.942
RILPL2	0.96 (0.74-1.24)	0.755	0.986	1.10 (0.69-1.76)	0.684	0.993	0.81 (0.51-1.28)	0.367	0.939
RIPK4	0.81 (0.53-1.25)	0.345	0.855	0.91 (0.43-1.94)	0.815	0.997	0.78 (0.33-1.82)	0.563	0.957
RLN1	1.02 (0.89-1.16)	0.793	0.994	0.87 (0.63-1.21)	0.413	0.903	1.09 (0.87-1.36)	0.476	0.944
RLN2	1.06 (0.78-1.43)	0.722	0.975	0.75 (0.36-1.59)	0.459	0.923	1.23 (0.70-2.18)	0.469	0.942
RNASE1	2.99 (1.73-5.17)	<0.001	0.021	3.21 (1.38-7.47)	0.007	0.160	3.75 (1.69-8.30)	0.001	0.185
RNASE10	1.15 (0.74-1.80)	0.537	0.948	0.72 (0.31-1.69)	0.457	0.923	2.20 (1.06-4.55)	0.033	0.613
RNASE3	1.17 (0.99-1.39)	0.065	0.473	1.29 (0.96-1.71)	0.086	0.491	1.00 (0.70-1.43)	1.000	1.000
RNASE4	3.11 (1.50-6.48)	0.002	0.115	2.77 (0.69-11.21)	0.153	0.626	4.12 (1.33-12.76)	0.014	0.452
RNASE6	2.17 (1.53-3.08)	<0.001	0.007	2.37 (1.29-4.33)	0.005	0.146	2.04 (1.21-3.44)	0.007	0.370
RNASEH2A	1.01 (0.56-1.84)	0.967	0.999	1.60 (0.74-3.45)	0.236	0.742	0.33 (0.07-1.58)	0.166	0.848

RNASET2	2.30 (1.10-4.81)	0.027	0.362	9.14 (2.73-30.65)	<0.001	0.047	1.77 (0.45-6.87)	0.411	0.942
RNF149	2.01 (1.02-3.95)	0.044	0.425	1.96 (0.59-6.44)	0.270	0.779	3.15 (0.93-10.65)	0.065	0.713
RNF168	1.00 (0.51-1.98)	0.989	0.999	0.56 (0.14-2.19)	0.400	0.901	2.23 (0.93-5.35)	0.074	0.718
RNF31	1.30 (0.79-2.15)	0.296	0.814	1.24 (0.50-3.08)	0.637	0.987	1.98 (0.89-4.41)	0.093	0.762
RNF4	0.95 (0.66-1.38)	0.787	0.994	1.23 (0.73-2.07)	0.433	0.908	1.01 (0.52-1.97)	0.972	0.994
RNF41	0.90 (0.64-1.27)	0.550	0.949	0.76 (0.40-1.45)	0.402	0.902	0.84 (0.42-1.66)	0.608	0.966
RNF43	1.06 (0.75-1.50)	0.753	0.986	1.08 (0.58-2.04)	0.801	0.997	1.23 (0.72-2.10)	0.449	0.942
RNF5	0.81 (0.56-1.15)	0.231	0.763	0.94 (0.49-1.82)	0.861	0.997	0.63 (0.33-1.19)	0.153	0.816
ROBO1	1.34 (0.60-2.99)	0.467	0.923	2.31 (0.62-8.68)	0.215	0.723	2.38 (0.62-9.09)	0.206	0.867
ROBO2	1.78 (0.75-4.22)	0.190	0.717	6.61 (1.52-28.77)	0.012	0.204	1.76 (0.38-8.16)	0.470	0.942
ROBO4	4.16 (1.12-15.51)	0.034	0.389	5.34 (0.47-61.23)	0.178	0.662	2.08 (0.18-23.58)	0.556	0.955
ROR1	1.60 (0.83-3.08)	0.160	0.680	3.22 (0.99-10.49)	0.052	0.392	1.43 (0.44-4.62)	0.554	0.955
RP2	1.01 (0.59-1.72)	0.980	0.999	1.12 (0.42-2.95)	0.820	0.997	0.76 (0.27-2.10)	0.592	0.965
RPA2	1.48 (0.98-2.24)	0.060	0.466	1.48 (0.72-3.03)	0.289	0.801	1.02 (0.26-3.92)	0.982	0.996
RPE	1.19 (0.86-1.65)	0.301	0.819	1.53 (0.89-2.64)	0.121	0.577	0.94 (0.47-1.85)	0.848	0.992
RPGR	1.64 (1.09-2.46)	0.017	0.298	0.93 (0.30-2.92)	0.902	0.997	1.26 (0.48-3.32)	0.642	0.973
RPL14	1.41 (0.72-2.75)	0.312	0.828	2.35 (0.85-6.51)	0.101	0.525	1.44 (0.47-4.37)	0.523	0.948
RPS10	1.68 (0.97-2.92)	0.064	0.473	1.90 (0.76-4.71)	0.168	0.649	2.62 (1.18-5.83)	0.018	0.512
RRAS	1.04 (0.86-1.27)	0.659	0.971	1.06 (0.75-1.51)	0.736	0.997	0.85 (0.57-1.25)	0.404	0.942
RRM2	1.09 (0.72-1.65)	0.677	0.974	1.61 (1.01-2.57)	0.043	0.362	1.04 (0.48-2.26)	0.920	0.992
RRM2B	0.71 (0.50-1.02)	0.064	0.473	0.80 (0.41-1.54)	0.500	0.941	0.63 (0.31-1.27)	0.194	0.866
RRP15	1.03 (0.78-1.36)	0.844	0.998	1.26 (0.84-1.89)	0.265	0.773	0.95 (0.55-1.64)	0.848	0.992
RSPO1	1.23 (0.67-2.24)	0.507	0.943	2.09 (0.75-5.79)	0.157	0.633	1.09 (0.37-3.27)	0.874	0.992
RSPO3	1.97 (1.01-3.85)	0.047	0.428	2.71 (0.92-7.94)	0.070	0.453	1.87 (0.57-6.12)	0.299	0.917
RTBDN	1.51 (0.75-3.04)	0.246	0.771	2.00 (0.57-6.97)	0.276	0.788	1.06 (0.28-4.03)	0.932	0.992

RTKN2	1.47 (1.02-2.10)	0.038	0.413	1.90 (1.11-3.23)	0.019	0.247	0.81 (0.33-1.97)	0.643	0.973
RTN4IP1	0.93 (0.70-1.22)	0.582	0.957	0.96 (0.58-1.59)	0.879	0.997	0.57 (0.33-1.00)	0.050	0.670
RTN4R	1.40 (0.77-2.55)	0.265	0.792	0.82 (0.27-2.50)	0.726	0.996	1.90 (0.63-5.68)	0.253	0.901
RUVBL1	0.97 (0.77-1.22)	0.768	0.987	0.68 (0.40-1.16)	0.158	0.633	0.97 (0.61-1.52)	0.883	0.992
RWDD1	1.04 (0.72-1.50)	0.826	0.995	1.06 (0.55-2.07)	0.857	0.997	0.96 (0.48-1.91)	0.907	0.992
RYR1	1.30 (0.62-2.73)	0.490	0.936	0.97 (0.21-4.56)	0.974	0.997	0.77 (0.15-4.03)	0.757	0.985
S100A11	1.14 (0.72-1.80)	0.587	0.960	1.38 (0.63-3.01)	0.423	0.905	0.53 (0.17-1.68)	0.280	0.913
S100A12	1.16 (0.84-1.58)	0.371	0.872	1.03 (0.57-1.87)	0.914	0.997	0.85 (0.46-1.56)	0.596	0.966
S100A13	1.78 (1.02-3.10)	0.043	0.421	1.26 (0.44-3.60)	0.672	0.993	1.29 (0.49-3.45)	0.607	0.966
S100A14	0.62 (0.38-1.01)	0.055	0.457	0.52 (0.21-1.30)	0.164	0.644	0.57 (0.22-1.42)	0.226	0.878
S100A16	0.71 (0.47-1.07)	0.104	0.582	0.66 (0.31-1.43)	0.293	0.803	1.01 (0.48-2.13)	0.984	0.996
S100A3	1.11 (0.69-1.79)	0.671	0.971	1.11 (0.46-2.69)	0.812	0.997	1.02 (0.42-2.49)	0.958	0.994
S100A4	0.94 (0.63-1.40)	0.765	0.987	0.75 (0.37-1.50)	0.416	0.903	0.76 (0.37-1.58)	0.467	0.942
S100G	1.09 (0.69-1.73)	0.700	0.975	0.99 (0.42-2.31)	0.981	0.998	2.13 (1.17-3.86)	0.013	0.437
S100P	1.45 (1.04-2.00)	0.027	0.362	1.17 (0.58-2.36)	0.660	0.992	1.76 (1.07-2.91)	0.027	0.588
SAA4	1.39 (0.77-2.52)	0.278	0.804	1.54 (0.53-4.46)	0.425	0.905	2.14 (0.95-4.83)	0.066	0.717
SAFB2	1.18 (0.46-3.06)	0.732	0.979	1.67 (0.33-8.34)	0.535	0.951	0.42 (0.05-3.32)	0.410	0.942
SAG	0.92 (0.53-1.58)	0.753	0.986	0.92 (0.34-2.49)	0.869	0.997	1.25 (0.46-3.41)	0.660	0.975
SAMD9L	0.99 (0.71-1.37)	0.933	0.999	1.09 (0.60-1.98)	0.780	0.997	0.78 (0.41-1.50)	0.460	0.942
SAP18	0.77 (0.54-1.12)	0.173	0.701	0.90 (0.45-1.83)	0.780	0.997	0.90 (0.44-1.82)	0.768	0.987
SARG	1.05 (0.84-1.31)	0.683	0.975	1.24 (0.82-1.87)	0.307	0.816	0.91 (0.60-1.38)	0.656	0.975
SART1	1.19 (0.69-2.05)	0.521	0.947	1.59 (0.80-3.19)	0.186	0.679	0.68 (0.18-2.60)	0.573	0.960
SAT1	1.10 (0.71-1.72)	0.664	0.971	1.38 (0.65-2.93)	0.406	0.903	0.57 (0.20-1.62)	0.294	0.915
SAT2	1.21 (0.84-1.73)	0.314	0.830	1.40 (0.79-2.50)	0.249	0.755	1.02 (0.47-2.17)	0.967	0.994
SATB1	1.21 (0.68-2.17)	0.513	0.945	0.61 (0.15-2.46)	0.491	0.939	1.58 (0.62-4.00)	0.336	0.934

SBSN	1.33 (0.67-2.65)	0.411	0.894	7.45 (2.28-24.31)	<0.001	0.063	0.51 (0.16-1.69)	0.272	0.910
SCAMP3	1.10 (0.84-1.43)	0.487	0.935	0.97 (0.60-1.58)	0.911	0.997	1.22 (0.74-1.99)	0.433	0.942
SCARA5	2.84 (1.59-5.07)	<0.001	0.046	3.73 (1.39-10.05)	0.009	0.187	3.76 (1.39-10.12)	0.009	0.402
SCARB1	1.25 (0.90-1.72)	0.180	0.706	1.15 (0.57-2.29)	0.700	0.994	1.15 (0.61-2.17)	0.673	0.975
SCARB2	1.97 (1.27-3.03)	0.002	0.113	1.89 (0.87-4.12)	0.108	0.545	1.96 (0.92-4.20)	0.083	0.743
SCARF1	1.11 (0.65-1.91)	0.708	0.975	1.42 (0.54-3.75)	0.477	0.932	0.76 (0.27-2.19)	0.616	0.966
SCARF2	2.56 (1.30-5.05)	0.007	0.190	6.03 (1.88-19.30)	0.002	0.106	2.32 (0.69-7.83)	0.174	0.853
SCG2	1.32 (0.72-2.40)	0.367	0.869	1.24 (0.41-3.78)	0.704	0.994	2.97 (1.07-8.24)	0.036	0.623
SCG3	0.90 (0.49-1.67)	0.748	0.984	2.03 (0.64-6.47)	0.232	0.742	1.02 (0.32-3.23)	0.977	0.994
SCGB1A1	0.97 (0.66-1.41)	0.857	0.998	0.65 (0.34-1.23)	0.186	0.679	1.05 (0.52-2.11)	0.902	0.992
SCGB2A2	1.37 (0.66-2.82)	0.401	0.894	2.16 (0.70-6.60)	0.178	0.662	0.58 (0.11-2.95)	0.512	0.947
SCGB3A1	0.83 (0.31-2.25)	0.714	0.975	2.36 (0.40-13.91)	0.342	0.848	2.22 (0.42-11.77)	0.347	0.936
SCGB3A2	1.10 (0.90-1.35)	0.369	0.871	0.91 (0.57-1.45)	0.691	0.994	1.24 (0.94-1.64)	0.121	0.785
SCGN	1.16 (0.69-1.95)	0.576	0.956	1.11 (0.43-2.86)	0.826	0.997	0.70 (0.26-1.91)	0.487	0.944
SCIN	0.91 (0.60-1.38)	0.649	0.971	0.42 (0.19-0.97)	0.042	0.355	1.78 (1.09-2.91)	0.022	0.551
SCLY	0.90 (0.58-1.40)	0.635	0.968	0.87 (0.39-1.95)	0.737	0.997	0.93 (0.41-2.11)	0.867	0.992
SCN2A	1.02 (0.59-1.76)	0.941	0.999	1.22 (0.51-2.92)	0.655	0.992	1.21 (0.45-3.27)	0.704	0.975
SCN2B	0.59 (0.30-1.16)	0.127	0.625	0.45 (0.11-1.75)	0.247	0.753	0.17 (0.03-0.95)	0.043	0.637
SCN3A	1.13 (0.57-2.22)	0.729	0.978	0.81 (0.20-3.21)	0.760	0.997	0.89 (0.23-3.52)	0.874	0.992
SCN3B	0.65 (0.39-1.09)	0.100	0.573	0.58 (0.23-1.52)	0.270	0.780	0.62 (0.24-1.61)	0.328	0.932
SCN4B	1.04 (0.60-1.79)	0.896	0.999	2.14 (0.99-4.63)	0.054	0.392	0.97 (0.36-2.61)	0.946	0.992
SCP2	0.79 (0.54-1.15)	0.215	0.746	0.60 (0.27-1.33)	0.212	0.718	0.46 (0.19-1.14)	0.094	0.762
SCPEP1	0.85 (0.50-1.43)	0.540	0.948	1.29 (0.52-3.20)	0.583	0.967	0.76 (0.28-2.10)	0.599	0.966
SCRG1	1.29 (0.58-2.87)	0.536	0.948	2.49 (0.74-8.33)	0.140	0.604	2.82 (0.75-10.67)	0.126	0.795
SCRIB	1.24 (0.99-1.57)	0.067	0.479	1.25 (0.82-1.92)	0.294	0.803	1.06 (0.64-1.74)	0.828	0.991

SCRN1	1.19 (0.81-1.75)	0.380	0.875	1.88 (1.02-3.46)	0.043	0.362	1.18 (0.58-2.41)	0.648	0.973
SCT	0.87 (0.53-1.42)	0.581	0.957	0.44 (0.16-1.20)	0.109	0.546	1.05 (0.43-2.56)	0.913	0.992
SDC1	1.48 (0.89-2.48)	0.132	0.633	3.50 (1.77-6.93)	<0.001	0.047	0.67 (0.23-2.00)	0.477	0.944
SDC4	0.79 (0.50-1.25)	0.322	0.838	0.85 (0.37-1.97)	0.705	0.994	0.63 (0.27-1.42)	0.263	0.910
SDCCAG8	0.99 (0.80-1.22)	0.916	0.999	0.96 (0.65-1.43)	0.845	0.997	1.01 (0.68-1.52)	0.945	0.992
SDHB	0.98 (0.67-1.42)	0.902	0.999	0.93 (0.47-1.86)	0.839	0.997	0.99 (0.49-2.02)	0.986	0.996
SDK2	1.76 (1.06-2.93)	0.030	0.372	2.69 (1.35-5.33)	0.005	0.142	0.84 (0.25-2.85)	0.781	0.987
SEC31A	0.85 (0.61-1.19)	0.346	0.855	0.85 (0.46-1.57)	0.609	0.978	0.64 (0.34-1.21)	0.171	0.853
SEL1L	1.12 (0.61-2.06)	0.717	0.975	1.24 (0.44-3.49)	0.677	0.993	0.85 (0.23-3.14)	0.807	0.991
SELE	1.73 (1.16-2.59)	0.007	0.201	1.45 (0.71-2.97)	0.310	0.816	1.92 (0.91-4.05)	0.086	0.745
SELENOP	0.40 (0.13-1.20)	0.102	0.576	0.70 (0.10-4.93)	0.721	0.995	0.12 (0.02-0.73)	0.022	0.551
SELL	0.66 (0.25-1.78)	0.412	0.894	4.39 (0.82-23.66)	0.085	0.487	0.29 (0.05-1.74)	0.176	0.853
SELP	0.90 (0.60-1.35)	0.611	0.965	1.35 (0.66-2.75)	0.415	0.903	0.75 (0.35-1.62)	0.471	0.943
SELPLG	0.71 (0.34-1.49)	0.366	0.869	0.69 (0.18-2.56)	0.576	0.965	1.15 (0.28-4.66)	0.845	0.992
SEMA3F	1.62 (0.76-3.45)	0.216	0.746	7.26 (1.97-26.70)	0.003	0.110	0.75 (0.19-3.03)	0.692	0.975
SEMA3G	0.92 (0.58-1.47)	0.723	0.975	1.04 (0.46-2.37)	0.927	0.997	1.30 (0.60-2.82)	0.505	0.947
SEMA4C	1.20 (0.61-2.35)	0.596	0.962	3.56 (1.68-7.55)	<0.001	0.063	0.37 (0.08-1.73)	0.205	0.867
SEMA4D	0.89 (0.41-1.96)	0.775	0.991	0.82 (0.19-3.54)	0.792	0.997	0.29 (0.07-1.23)	0.094	0.762
SEMA6C	1.66 (0.81-3.40)	0.170	0.697	2.54 (0.87-7.37)	0.087	0.493	1.70 (0.54-5.37)	0.370	0.939
SEMA7A	1.75 (0.88-3.47)	0.111	0.598	2.13 (0.67-6.82)	0.200	0.707	0.96 (0.25-3.73)	0.952	0.992
SEPTIN3	0.94 (0.68-1.30)	0.716	0.975	1.67 (1.09-2.56)	0.019	0.251	0.65 (0.34-1.22)	0.180	0.853
SEPTIN7	1.13 (0.80-1.61)	0.478	0.928	1.23 (0.74-2.06)	0.423	0.905	0.82 (0.36-1.88)	0.644	0.973
SEPTIN8	1.85 (1.13-3.03)	0.014	0.280	2.28 (1.04-5.00)	0.040	0.349	3.00 (1.58-5.69)	<0.001	0.179
SEPTIN9	0.74 (0.32-1.68)	0.466	0.922	1.46 (0.52-4.13)	0.476	0.932	0.23 (0.04-1.42)	0.113	0.769
SERPINA1	0.55 (0.03-11.39)	0.700	0.975	3.24 (0.01-761.28)	0.673	0.993	0.41 (0.00-134.55)	0.765	0.987

SERPINA11	1.58 (0.93-2.70)	0.093	0.559	1.98 (0.75-5.26)	0.169	0.652	2.47 (0.91-6.69)	0.075	0.718
SERPINA12	0.90 (0.73-1.12)	0.340	0.854	1.01 (0.73-1.41)	0.951	0.997	0.78 (0.48-1.26)	0.302	0.919
SERPINA3	1.07 (0.22-5.13)	0.931	0.999	2.46 (0.21-29.51)	0.477	0.932	0.23 (0.01-5.05)	0.351	0.938
SERPINA4	0.84 (0.30-2.37)	0.744	0.984	2.67 (0.40-17.79)	0.310	0.816	0.56 (0.09-3.52)	0.541	0.952
SERPINA5	0.66 (0.28-1.54)	0.340	0.854	0.80 (0.18-3.50)	0.770	0.997	0.25 (0.05-1.21)	0.085	0.745
SERPINA6	0.72 (0.16-3.36)	0.678	0.974	0.27 (0.02-4.26)	0.353	0.862	0.39 (0.02-6.49)	0.513	0.947
SERPINA7	0.30 (0.07-1.26)	0.100	0.573	0.12 (0.01-1.69)	0.116	0.564	0.09 (0.01-1.13)	0.062	0.698
SERPINA9	1.48 (1.14-1.92)	0.003	0.133	2.10 (1.34-3.28)	0.001	0.066	1.06 (0.64-1.75)	0.829	0.991
SERPINB1	0.94 (0.72-1.23)	0.664	0.971	0.98 (0.60-1.61)	0.949	0.997	0.95 (0.58-1.58)	0.855	0.992
SERPINB5	1.25 (0.75-2.08)	0.386	0.879	1.87 (0.69-5.06)	0.217	0.725	0.99 (0.40-2.48)	0.985	0.996
SERPINB6	0.98 (0.66-1.44)	0.901	0.999	1.02 (0.50-2.09)	0.948	0.997	0.98 (0.46-2.06)	0.955	0.992
SERPINB8	1.37 (1.02-1.86)	0.039	0.413	1.61 (0.96-2.70)	0.069	0.448	1.40 (0.85-2.33)	0.187	0.862
SERPINB9	1.09 (0.55-2.18)	0.798	0.994	1.05 (0.28-3.90)	0.940	0.997	1.07 (0.27-4.26)	0.928	0.992
SERPINC1	0.72 (0.15-3.58)	0.690	0.975	0.67 (0.04-12.00)	0.787	0.997	0.65 (0.03-12.64)	0.778	0.987
SERPIND1	0.76 (0.33-1.76)	0.518	0.947	1.13 (0.23-5.48)	0.880	0.997	0.23 (0.05-1.02)	0.053	0.687
SERPINE1	0.90 (0.65-1.26)	0.554	0.950	1.23 (0.65-2.30)	0.523	0.948	0.80 (0.44-1.47)	0.471	0.943
SERPINE2	0.91 (0.61-1.35)	0.638	0.968	1.21 (0.60-2.44)	0.601	0.973	0.48 (0.21-1.12)	0.088	0.745
SERPINF1	0.89 (0.39-2.05)	0.785	0.994	2.85 (0.66-12.27)	0.159	0.635	1.72 (0.39-7.62)	0.476	0.944
SERPINF2	0.90 (0.14-5.99)	0.913	0.999	0.18 (0.01-5.54)	0.325	0.830	0.47 (0.01-15.94)	0.675	0.975
SERPING1	1.56 (0.42-5.84)	0.506	0.943	4.24 (0.38-46.72)	0.238	0.742	0.44 (0.04-4.62)	0.496	0.947
SERPINH1	1.08 (0.89-1.32)	0.437	0.907	1.21 (0.85-1.72)	0.286	0.799	0.92 (0.63-1.37)	0.694	0.975
SERPINI1	0.96 (0.44-2.11)	0.922	0.999	3.60 (1.09-11.93)	0.036	0.326	0.44 (0.10-1.90)	0.272	0.910
SERPINI2	0.84 (0.55-1.28)	0.419	0.898	1.11 (0.54-2.27)	0.780	0.997	0.80 (0.37-1.71)	0.563	0.957
SESTD1	1.00 (0.70-1.44)	0.996	0.999	0.88 (0.43-1.77)	0.718	0.995	0.64 (0.30-1.41)	0.271	0.910
SETMAR	1.00 (0.49-2.02)	0.995	0.999	1.31 (0.37-4.61)	0.678	0.993	1.10 (0.31-3.87)	0.879	0.992

SEZ6	2.29 (1.09-4.79)	0.029	0.371	0.89 (0.20-3.84)	0.872	0.997	5.96 (2.23-15.94)	<0.001	0.157
SEZ6L	1.57 (0.75-3.30)	0.232	0.763	1.62 (0.42-6.20)	0.482	0.934	2.18 (0.57-8.30)	0.252	0.901
SEZ6L2	1.15 (0.50-2.66)	0.747	0.984	2.17 (0.57-8.29)	0.256	0.767	2.96 (0.81-10.85)	0.101	0.762
SF3B4	0.88 (0.63-1.22)	0.430	0.904	0.99 (0.54-1.80)	0.971	0.997	0.98 (0.55-1.76)	0.958	0.994
SFRP1	1.01 (0.61-1.67)	0.980	0.999	0.92 (0.37-2.29)	0.862	0.997	1.59 (0.76-3.32)	0.219	0.869
SFRP4	2.25 (1.25-4.05)	0.007	0.190	8.67 (2.87-26.16)	<0.001	0.027	3.28 (1.12-9.61)	0.030	0.601
SFTPA1	0.80 (0.52-1.22)	0.301	0.819	0.67 (0.29-1.54)	0.348	0.855	0.64 (0.31-1.33)	0.233	0.884
SFTPA2	1.02 (0.66-1.57)	0.934	0.999	0.86 (0.38-1.93)	0.711	0.995	1.29 (0.66-2.52)	0.457	0.942
SFTPD	1.03 (0.73-1.44)	0.874	0.998	1.20 (0.65-2.22)	0.561	0.962	0.83 (0.44-1.55)	0.556	0.955
SGSH	1.02 (0.72-1.45)	0.901	0.999	0.88 (0.46-1.68)	0.707	0.994	1.66 (0.93-2.97)	0.087	0.745
SH2B3	1.02 (0.81-1.29)	0.840	0.998	1.00 (0.66-1.53)	0.986	0.998	0.99 (0.64-1.53)	0.963	0.994
SH2D1A	0.95 (0.63-1.45)	0.826	0.995	0.94 (0.44-1.99)	0.872	0.997	0.64 (0.28-1.48)	0.301	0.919
SH3BGRL2	0.92 (0.58-1.44)	0.707	0.975	1.10 (0.54-2.23)	0.801	0.997	0.70 (0.26-1.89)	0.485	0.944
SH3BP1	0.71 (0.37-1.35)	0.291	0.809	0.75 (0.23-2.41)	0.631	0.984	0.68 (0.20-2.35)	0.546	0.954
SH3GL3	0.98 (0.71-1.34)	0.883	0.999	0.84 (0.50-1.40)	0.500	0.941	1.24 (0.66-2.35)	0.500	0.947
SH3GLB2	1.14 (0.80-1.63)	0.464	0.922	1.25 (0.67-2.34)	0.482	0.934	1.32 (0.72-2.42)	0.369	0.939
SHBG	1.27 (0.77-2.12)	0.350	0.856	2.18 (0.87-5.50)	0.098	0.520	1.42 (0.54-3.74)	0.479	0.944
SHC1	0.91 (0.75-1.12)	0.388	0.882	0.98 (0.68-1.42)	0.932	0.997	0.77 (0.50-1.17)	0.217	0.869
SHD	1.08 (0.84-1.40)	0.548	0.949	0.99 (0.61-1.61)	0.963	0.997	1.20 (0.82-1.74)	0.348	0.937
SHH	0.84 (0.41-1.70)	0.623	0.966	1.69 (0.50-5.64)	0.397	0.897	0.39 (0.12-1.28)	0.121	0.785
SHISA5	2.34 (1.22-4.48)	0.011	0.245	3.68 (1.53-8.86)	0.004	0.124	1.28 (0.32-5.23)	0.727	0.978
SHMT1	0.86 (0.66-1.11)	0.246	0.771	0.91 (0.55-1.49)	0.710	0.995	0.76 (0.46-1.26)	0.285	0.915
SHPK	1.02 (0.49-2.13)	0.952	0.999	1.14 (0.32-4.01)	0.840	0.997	1.44 (0.37-5.62)	0.597	0.966
SIAE	1.11 (0.68-1.82)	0.670	0.971	1.64 (0.69-3.89)	0.260	0.772	0.82 (0.32-2.10)	0.679	0.975
SIGLEC1	1.54 (1.01-2.36)	0.047	0.428	2.03 (1.00-4.16)	0.052	0.391	1.74 (0.84-3.62)	0.135	0.796

SIGLEC10	1.11 (0.59-2.09)	0.742	0.984	2.29 (0.83-6.35)	0.110	0.546	1.02 (0.33-3.17)	0.975	0.994
SIGLEC15	0.91 (0.66-1.26)	0.571	0.956	1.09 (0.66-1.82)	0.728	0.997	0.89 (0.48-1.66)	0.715	0.976
SIGLEC5	1.44 (1.10-1.88)	0.007	0.195	2.41 (1.41-4.10)	0.001	0.067	1.25 (0.79-1.97)	0.336	0.934
SIGLEC6	1.51 (0.76-2.98)	0.235	0.767	2.18 (0.65-7.37)	0.208	0.712	1.43 (0.43-4.79)	0.559	0.955
SIGLEC7	2.09 (1.02-4.29)	0.043	0.425	2.36 (0.65-8.59)	0.193	0.691	1.21 (0.30-4.92)	0.785	0.989
SIGLEC8	0.93 (0.53-1.60)	0.781	0.992	2.02 (0.78-5.23)	0.147	0.618	0.67 (0.23-1.94)	0.456	0.942
SIGLEC9	1.04 (0.53-2.04)	0.913	0.999	0.48 (0.14-1.62)	0.238	0.742	0.96 (0.28-3.28)	0.954	0.992
SIL1	1.18 (0.58-2.42)	0.644	0.971	1.14 (0.27-4.81)	0.855	0.997	1.13 (0.23-5.58)	0.885	0.992
SIRPA	1.23 (0.79-1.91)	0.350	0.856	1.48 (0.63-3.45)	0.364	0.867	1.57 (0.67-3.66)	0.300	0.919
SIRPB1	1.27 (0.79-2.04)	0.326	0.843	1.46 (0.64-3.33)	0.369	0.873	1.59 (0.66-3.81)	0.299	0.917
SIRT1	1.07 (0.80-1.44)	0.647	0.971	0.74 (0.33-1.68)	0.477	0.932	1.22 (0.73-2.02)	0.443	0.942
SIRT2	1.05 (0.80-1.36)	0.733	0.979	1.11 (0.69-1.79)	0.661	0.992	0.95 (0.58-1.55)	0.839	0.991
SIRT5	1.17 (0.81-1.69)	0.402	0.894	1.23 (0.62-2.45)	0.553	0.960	1.26 (0.64-2.49)	0.503	0.947
SIT1	0.97 (0.64-1.48)	0.889	0.999	1.44 (0.70-2.98)	0.325	0.830	0.79 (0.38-1.67)	0.543	0.952
SKAP1	0.85 (0.62-1.18)	0.336	0.851	1.15 (0.65-2.02)	0.634	0.986	0.83 (0.46-1.47)	0.518	0.948
SKAP2	0.98 (0.78-1.22)	0.835	0.997	1.04 (0.69-1.58)	0.845	0.997	0.81 (0.53-1.24)	0.333	0.934
SKIV2L	1.20 (0.76-1.91)	0.431	0.904	0.95 (0.37-2.43)	0.921	0.997	1.29 (0.60-2.77)	0.516	0.947
SLA2	0.83 (0.65-1.06)	0.142	0.659	0.92 (0.59-1.43)	0.706	0.994	0.61 (0.39-0.96)	0.033	0.613
SLAMF1	1.17 (0.79-1.75)	0.429	0.904	2.17 (1.05-4.48)	0.035	0.326	0.93 (0.46-1.89)	0.840	0.991
SLAMF6	0.96 (0.53-1.77)	0.904	0.999	1.54 (0.55-4.33)	0.411	0.903	1.12 (0.39-3.22)	0.831	0.991
SLAMF7	1.20 (0.82-1.77)	0.348	0.855	1.45 (0.73-2.88)	0.294	0.803	0.85 (0.40-1.79)	0.661	0.975
SLAMF8	1.03 (0.70-1.50)	0.891	0.999	1.01 (0.51-1.99)	0.972	0.997	1.01 (0.52-1.96)	0.969	0.994
SLC12A2	0.74 (0.24-2.27)	0.593	0.961	0.82 (0.11-6.18)	0.850	0.997	0.48 (0.05-4.17)	0.505	0.947
SLC13A1	0.95 (0.65-1.39)	0.799	0.994	1.43 (0.94-2.18)	0.093	0.509	1.11 (0.61-2.03)	0.727	0.978
SLC16A1	0.90 (0.63-1.28)	0.552	0.950	0.79 (0.38-1.63)	0.527	0.948	1.11 (0.62-2.01)	0.718	0.976

SLC1A4	1.07 (0.62-1.85)	0.806	0.995	1.27 (0.51-3.13)	0.606	0.976	0.79 (0.25-2.51)	0.690	0.975
SLC27A4	0.97 (0.68-1.37)	0.844	0.998	1.26 (0.72-2.21)	0.422	0.905	1.09 (0.58-2.05)	0.785	0.989
SLC28A1	0.81 (0.50-1.30)	0.388	0.882	0.96 (0.46-2.02)	0.924	0.997	0.46 (0.15-1.43)	0.181	0.853
SLC34A3	1.53 (0.95-2.44)	0.078	0.514	1.64 (0.70-3.86)	0.258	0.772	1.23 (0.47-3.20)	0.670	0.975
SLC39A14	1.67 (1.26-2.20)	<0.001	0.046	2.00 (1.37-2.92)	<0.001	0.047	1.56 (0.76-3.20)	0.230	0.882
SLC39A5	1.11 (0.72-1.70)	0.643	0.971	1.51 (0.68-3.34)	0.312	0.816	0.97 (0.44-2.13)	0.942	0.992
SLC44A4	0.71 (0.45-1.14)	0.155	0.676	0.39 (0.14-1.10)	0.075	0.465	0.30 (0.11-0.84)	0.022	0.551
SLC4A1	0.84 (0.48-1.45)	0.528	0.948	0.82 (0.31-2.19)	0.699	0.994	1.09 (0.43-2.74)	0.860	0.992
SLC51B	1.02 (0.74-1.40)	0.925	0.999	0.89 (0.46-1.71)	0.718	0.995	0.72 (0.34-1.53)	0.392	0.942
SLC9A3R1	0.91 (0.67-1.24)	0.565	0.956	1.02 (0.59-1.77)	0.943	0.997	0.66 (0.36-1.20)	0.173	0.853
SLC9A3R2	1.11 (0.68-1.82)	0.671	0.971	1.29 (0.62-2.70)	0.501	0.941	0.72 (0.22-2.34)	0.589	0.965
SLIRP	1.10 (0.71-1.69)	0.670	0.971	1.34 (0.75-2.39)	0.323	0.829	1.03 (0.44-2.42)	0.940	0.992
SLIT2	1.24 (0.81-1.87)	0.320	0.836	1.74 (0.90-3.34)	0.099	0.524	1.74 (0.89-3.40)	0.105	0.763
SLITRK1	1.12 (0.53-2.35)	0.768	0.987	0.53 (0.13-2.12)	0.366	0.869	1.42 (0.37-5.39)	0.609	0.966
SLITRK2	2.23 (1.26-3.95)	0.006	0.184	6.89 (2.22-21.40)	<0.001	0.063	2.01 (0.73-5.51)	0.176	0.853
SLITRK6	0.95 (0.51-1.80)	0.884	0.999	2.32 (0.75-7.10)	0.142	0.607	0.59 (0.19-1.81)	0.357	0.939
SLK	1.13 (0.86-1.47)	0.385	0.878	0.92 (0.51-1.68)	0.796	0.997	1.33 (0.87-2.04)	0.187	0.862
SLMAP	1.08 (0.81-1.45)	0.589	0.961	0.95 (0.55-1.64)	0.859	0.997	1.03 (0.59-1.79)	0.917	0.992
SLURP1	1.41 (0.82-2.39)	0.211	0.741	1.93 (0.81-4.57)	0.136	0.600	1.88 (0.72-4.91)	0.199	0.867
SMAD1	0.89 (0.68-1.17)	0.409	0.894	0.68 (0.41-1.12)	0.132	0.596	0.91 (0.56-1.48)	0.697	0.975
SMAD2	0.93 (0.66-1.33)	0.707	0.975	1.48 (0.84-2.62)	0.174	0.660	0.63 (0.31-1.30)	0.212	0.867
SMAD3	1.08 (0.65-1.81)	0.765	0.987	1.54 (0.64-3.72)	0.334	0.839	0.83 (0.30-2.32)	0.722	0.977
SMAD5	0.82 (0.29-2.32)	0.710	0.975	1.76 (0.23-13.48)	0.585	0.967	1.31 (0.17-9.88)	0.793	0.989
SMARCA2	1.39 (1.05-1.85)	0.024	0.350	1.11 (0.62-1.97)	0.724	0.995	1.08 (0.61-1.93)	0.787	0.989
SMC3	1.12 (0.77-1.64)	0.544	0.948	0.97 (0.42-2.23)	0.943	0.997	0.58 (0.20-1.66)	0.311	0.923

SMNDC1	0.92 (0.59-1.44)	0.711	0.975	1.18 (0.61-2.31)	0.623	0.983	0.94 (0.43-2.03)	0.869	0.992
SMOC1	1.64 (0.89-3.04)	0.116	0.607	1.46 (0.46-4.67)	0.519	0.945	1.07 (0.35-3.24)	0.909	0.992
SMOC2	1.86 (1.14-3.03)	0.013	0.278	5.71 (2.56-12.75)	<0.001	0.010	1.97 (0.81-4.80)	0.137	0.798
SMPD1	1.18 (0.78-1.79)	0.427	0.904	1.71 (0.92-3.14)	0.087	0.493	1.07 (0.49-2.33)	0.859	0.992
SMPD3	1.21 (0.84-1.73)	0.303	0.821	1.11 (0.54-2.30)	0.770	0.997	1.56 (0.87-2.78)	0.133	0.796
SMPDL3A	0.91 (0.66-1.25)	0.570	0.956	1.20 (0.67-2.16)	0.532	0.950	0.77 (0.43-1.38)	0.381	0.942
SMPDL3B	0.99 (0.71-1.37)	0.949	0.999	0.88 (0.45-1.70)	0.704	0.994	1.18 (0.66-2.11)	0.569	0.960
SMS	1.12 (0.77-1.63)	0.551	0.949	1.14 (0.58-2.25)	0.708	0.995	0.72 (0.30-1.76)	0.473	0.944
SMTN	1.26 (1.00-1.59)	0.047	0.428	0.98 (0.61-1.55)	0.919	0.997	1.59 (1.09-2.33)	0.015	0.480
SNAP23	0.97 (0.74-1.27)	0.811	0.995	1.05 (0.63-1.73)	0.856	0.997	0.81 (0.49-1.34)	0.417	0.942
SNAP25	1.22 (0.71-2.10)	0.470	0.924	1.62 (0.65-4.05)	0.303	0.814	1.29 (0.48-3.47)	0.614	0.966
SNAP29	0.95 (0.74-1.23)	0.710	0.975	1.17 (0.73-1.88)	0.517	0.945	0.65 (0.40-1.08)	0.097	0.762
SNAPIN	0.87 (0.55-1.38)	0.565	0.956	0.96 (0.43-2.15)	0.922	0.997	0.97 (0.43-2.19)	0.943	0.992
SNCA	0.94 (0.76-1.16)	0.553	0.950	0.95 (0.64-1.40)	0.787	0.997	1.02 (0.70-1.49)	0.898	0.992
SNCG	1.19 (0.87-1.62)	0.280	0.804	1.26 (0.72-2.20)	0.414	0.903	1.20 (0.71-2.04)	0.492	0.947
SNED1	1.33 (0.62-2.86)	0.461	0.920	3.74 (1.02-13.74)	0.047	0.374	1.06 (0.26-4.28)	0.937	0.992
SNRPB2	1.29 (0.86-1.94)	0.216	0.746	1.55 (0.84-2.88)	0.164	0.644	1.51 (0.92-2.49)	0.103	0.763
SNU13	0.79 (0.47-1.33)	0.372	0.872	1.10 (0.54-2.23)	0.792	0.997	0.39 (0.09-1.70)	0.209	0.867
SNX15	0.88 (0.57-1.38)	0.585	0.960	1.05 (0.49-2.25)	0.899	0.997	1.19 (0.56-2.51)	0.654	0.975
SNX18	1.26 (0.71-2.24)	0.426	0.902	2.74 (1.21-6.20)	0.016	0.232	0.67 (0.18-2.52)	0.555	0.955
SNX2	1.10 (0.83-1.47)	0.496	0.939	1.20 (0.73-1.97)	0.464	0.926	1.10 (0.66-1.83)	0.724	0.978
SNX5	0.53 (0.20-1.38)	0.191	0.718	0.57 (0.10-3.28)	0.529	0.949	0.18 (0.02-1.39)	0.099	0.762
SNX9	1.14 (0.82-1.58)	0.439	0.909	1.29 (0.72-2.33)	0.390	0.893	1.13 (0.60-2.13)	0.701	0.975
SOD1	1.22 (0.78-1.92)	0.379	0.875	1.24 (0.56-2.76)	0.600	0.973	1.19 (0.54-2.61)	0.670	0.975
SOD2	0.89 (0.52-1.53)	0.675	0.974	1.02 (0.39-2.65)	0.963	0.997	1.29 (0.51-3.30)	0.591	0.965

SOD3	0.84 (0.48-1.47)	0.547	0.949	1.29 (0.66-2.51)	0.455	0.923	0.94 (0.41-2.16)	0.883	0.992
SORBS1	1.21 (0.71-2.07)	0.479	0.928	1.58 (0.74-3.34)	0.237	0.742	1.11 (0.40-3.12)	0.837	0.991
SORCS2	2.06 (1.27-3.33)	0.003	0.134	1.66 (0.66-4.17)	0.277	0.788	2.12 (0.91-4.96)	0.083	0.743
SORD	0.77 (0.57-1.02)	0.067	0.481	0.70 (0.42-1.17)	0.175	0.662	0.69 (0.43-1.11)	0.125	0.795
SORT1	1.29 (0.73-2.27)	0.381	0.875	2.11 (0.76-5.84)	0.150	0.626	0.81 (0.28-2.33)	0.700	0.975
SOST	1.70 (0.98-2.93)	0.058	0.463	2.13 (0.78-5.79)	0.140	0.604	1.82 (0.67-4.92)	0.239	0.889
SOWAHA	1.27 (0.90-1.78)	0.177	0.706	1.20 (0.62-2.32)	0.595	0.972	0.99 (0.46-2.15)	0.980	0.995
SOX2	0.58 (0.19-1.78)	0.338	0.852	0.68 (0.08-5.49)	0.715	0.995	0.38 (0.05-2.88)	0.347	0.936
SOX9	1.28 (0.50-3.28)	0.610	0.965	1.45 (0.28-7.50)	0.660	0.992	3.43 (0.79-14.82)	0.099	0.762
SPACA5_SPACA5B	1.72 (0.69-4.29)	0.246	0.771	1.32 (0.26-6.77)	0.742	0.997	5.68 (1.45-22.30)	0.013	0.437
SPAG1	0.87 (0.53-1.42)	0.579	0.956	1.04 (0.45-2.42)	0.921	0.997	0.39 (0.12-1.27)	0.117	0.780
SPARC	0.96 (0.70-1.31)	0.782	0.994	0.99 (0.55-1.77)	0.975	0.997	0.69 (0.40-1.19)	0.178	0.853
SPARCL1	0.67 (0.29-1.52)	0.335	0.851	2.52 (0.67-9.51)	0.173	0.659	0.16 (0.03-0.83)	0.029	0.588
SPART	1.08 (0.78-1.48)	0.659	0.971	1.17 (0.65-2.12)	0.598	0.973	0.99 (0.54-1.81)	0.966	0.994
SPESP1	1.09 (0.51-2.32)	0.818	0.995	1.66 (0.45-6.16)	0.452	0.920	0.50 (0.12-2.10)	0.346	0.936
SPINK1	1.46 (1.01-2.11)	0.042	0.420	1.04 (0.50-2.16)	0.924	0.997	1.71 (0.90-3.24)	0.100	0.762
SPINK2	1.62 (1.02-2.57)	0.040	0.416	2.71 (1.23-5.97)	0.013	0.213	1.00 (0.41-2.46)	0.997	0.999
SPINK4	1.37 (1.02-1.83)	0.036	0.403	1.08 (0.61-1.90)	0.797	0.997	1.43 (0.85-2.40)	0.183	0.859
SPINK5	1.39 (0.77-2.50)	0.272	0.799	2.54 (0.99-6.53)	0.054	0.392	0.69 (0.22-2.11)	0.515	0.947
SPINK6	0.99 (0.65-1.52)	0.975	0.999	0.95 (0.44-2.05)	0.892	0.997	0.90 (0.40-2.00)	0.789	0.989
SPINK8	0.93 (0.43-2.02)	0.858	0.998	1.37 (0.46-4.08)	0.567	0.963	0.97 (0.22-4.24)	0.963	0.994
SPINT1	1.98 (0.89-4.42)	0.096	0.568	3.26 (0.75-14.19)	0.116	0.563	1.90 (0.46-7.87)	0.378	0.942
SPINT2	1.57 (0.89-2.76)	0.116	0.607	1.35 (0.48-3.81)	0.573	0.964	1.14 (0.39-3.30)	0.810	0.991
SPINT3	0.91 (0.83-0.99)	0.036	0.402	0.87 (0.74-1.03)	0.096	0.520	0.82 (0.70-0.95)	0.009	0.402
SPOCK1	0.66 (0.29-1.48)	0.315	0.832	0.48 (0.11-2.12)	0.336	0.839	0.58 (0.14-2.46)	0.463	0.942

SPON1	1.63 (1.06-2.52)	0.026	0.360	2.19 (1.16-4.14)	0.016	0.234	1.56 (0.80-3.04)	0.195	0.866
SPON2	1.92 (1.09-3.40)	0.024	0.350	3.31 (1.26-8.73)	0.016	0.230	1.74 (0.61-4.96)	0.301	0.919
SPP1	1.32 (0.82-2.12)	0.246	0.771	2.29 (0.98-5.37)	0.056	0.399	1.18 (0.50-2.82)	0.702	0.975
SPRED2	0.93 (0.49-1.75)	0.820	0.995	1.10 (0.35-3.43)	0.870	0.997	1.35 (0.42-4.31)	0.615	0.966
SPRING1	1.40 (1.00-1.98)	0.053	0.445	1.84 (1.13-2.99)	0.014	0.221	1.50 (0.76-2.95)	0.240	0.891
SPRR1B	1.08 (0.81-1.44)	0.583	0.957	0.86 (0.44-1.69)	0.668	0.993	1.09 (0.63-1.88)	0.765	0.987
SPRR3	1.01 (0.73-1.40)	0.960	0.999	1.21 (0.68-2.13)	0.521	0.946	0.96 (0.51-1.82)	0.908	0.992
SPRY2	0.87 (0.67-1.12)	0.282	0.806	0.94 (0.58-1.50)	0.782	0.997	0.80 (0.49-1.31)	0.371	0.939
SPTBN2	0.63 (0.25-1.59)	0.329	0.846	0.88 (0.22-3.54)	0.855	0.997	0.75 (0.15-3.77)	0.726	0.978
SPTLC1	1.13 (0.54-2.38)	0.739	0.982	1.77 (0.57-5.48)	0.323	0.828	0.76 (0.17-3.32)	0.715	0.976
SRC	0.92 (0.76-1.12)	0.410	0.894	0.92 (0.65-1.31)	0.654	0.992	0.77 (0.53-1.11)	0.156	0.824
SRP14	1.01 (0.67-1.53)	0.945	0.999	1.33 (0.67-2.67)	0.417	0.903	0.80 (0.37-1.75)	0.575	0.960
SRPK2	0.97 (0.74-1.27)	0.811	0.995	0.94 (0.57-1.54)	0.795	0.997	0.98 (0.59-1.64)	0.938	0.992
SRPX	1.83 (0.93-3.61)	0.079	0.515	3.65 (1.07-12.48)	0.039	0.342	2.69 (0.80-9.09)	0.110	0.763
SSB	1.00 (0.68-1.47)	0.998	0.999	1.10 (0.58-2.11)	0.763	0.997	1.19 (0.74-1.92)	0.466	0.942
SSBP1	0.86 (0.37-2.03)	0.732	0.979	1.29 (0.33-5.10)	0.715	0.995	0.26 (0.04-1.57)	0.141	0.808
SSC4D	0.80 (0.66-0.96)	0.019	0.313	0.99 (0.73-1.36)	0.970	0.997	0.70 (0.49-0.99)	0.041	0.630
SSC5D	0.91 (0.54-1.54)	0.738	0.982	0.99 (0.38-2.58)	0.991	0.998	0.68 (0.27-1.73)	0.424	0.942
SSH3	0.63 (0.27-1.48)	0.293	0.811	1.39 (0.27-7.08)	0.694	0.994	0.36 (0.07-1.95)	0.238	0.889
SSNA1	0.96 (0.61-1.51)	0.854	0.998	1.57 (0.76-3.24)	0.220	0.729	1.26 (0.58-2.76)	0.558	0.955
ST13	0.86 (0.56-1.33)	0.503	0.943	0.82 (0.37-1.79)	0.613	0.979	1.05 (0.49-2.24)	0.897	0.992
ST3GAL1	1.19 (0.74-1.90)	0.478	0.928	1.09 (0.47-2.50)	0.842	0.997	1.55 (0.83-2.90)	0.169	0.852
ST6GAL1	2.32 (1.14-4.71)	0.020	0.319	2.26 (0.60-8.55)	0.228	0.739	1.83 (0.49-6.86)	0.370	0.939
ST8SIA1	1.00 (0.59-1.70)	0.993	0.999	0.88 (0.30-2.58)	0.810	0.997	0.91 (0.28-2.93)	0.879	0.992
STAB2	2.79 (1.12-6.96)	0.027	0.362	18.62 (3.27-106.16)	<0.001	0.063	1.14 (0.22-6.03)	0.876	0.992

STAM	0.94 (0.45-1.98)	0.869	0.998	0.94 (0.23-3.85)	0.933	0.997	1.30 (0.37-4.60)	0.687	0.975
STAMBP	1.03 (0.72-1.47)	0.877	0.998	1.04 (0.54-2.00)	0.903	0.997	0.92 (0.47-1.81)	0.803	0.991
STAT2	1.11 (0.79-1.55)	0.554	0.950	1.39 (0.77-2.51)	0.272	0.782	0.90 (0.46-1.75)	0.752	0.985
STAT5B	1.06 (0.89-1.25)	0.520	0.947	1.13 (0.83-1.54)	0.451	0.920	1.07 (0.78-1.47)	0.684	0.975
STAU1	0.98 (0.63-1.51)	0.925	0.999	1.22 (0.61-2.45)	0.575	0.965	0.43 (0.13-1.45)	0.173	0.853
STC1	1.48 (0.95-2.31)	0.082	0.523	2.07 (0.93-4.60)	0.075	0.465	1.00 (0.41-2.48)	0.995	0.998
STC2	1.13 (0.45-2.87)	0.792	0.994	5.94 (1.23-28.68)	0.027	0.284	1.39 (0.24-8.15)	0.717	0.976
STEAP4	0.84 (0.54-1.31)	0.447	0.914	0.85 (0.37-1.94)	0.693	0.994	0.67 (0.26-1.76)	0.420	0.942
STIP1	1.03 (0.73-1.45)	0.876	0.998	1.02 (0.56-1.89)	0.937	0.997	1.11 (0.60-2.06)	0.730	0.978
STK11	0.96 (0.70-1.31)	0.794	0.994	0.94 (0.53-1.66)	0.833	0.997	0.88 (0.49-1.58)	0.664	0.975
STK24	0.99 (0.68-1.44)	0.969	0.999	1.20 (0.61-2.34)	0.602	0.973	0.89 (0.44-1.82)	0.757	0.985
STK4	0.93 (0.71-1.22)	0.603	0.962	0.93 (0.56-1.52)	0.762	0.997	0.81 (0.49-1.34)	0.422	0.942
STOML2	1.10 (0.82-1.48)	0.534	0.948	1.04 (0.58-1.86)	0.885	0.997	0.84 (0.42-1.67)	0.622	0.967
STX16	0.81 (0.53-1.23)	0.319	0.836	0.55 (0.23-1.31)	0.177	0.662	0.79 (0.34-1.84)	0.585	0.965
STX1B	0.97 (0.54-1.77)	0.931	0.999	1.15 (0.43-3.06)	0.774	0.997	1.39 (0.53-3.67)	0.506	0.947
STX3	0.78 (0.36-1.71)	0.535	0.948	0.60 (0.14-2.61)	0.492	0.940	0.67 (0.15-3.02)	0.601	0.966
STX4	0.94 (0.61-1.44)	0.774	0.991	1.00 (0.47-2.15)	0.997	0.998	0.85 (0.37-1.94)	0.703	0.975
STX5	0.98 (0.51-1.90)	0.956	0.999	1.14 (0.39-3.30)	0.814	0.997	0.73 (0.19-2.79)	0.644	0.973
STX6	0.99 (0.69-1.42)	0.962	0.999	0.73 (0.35-1.54)	0.408	0.903	1.00 (0.49-2.03)	0.997	0.999
STX7	0.98 (0.58-1.66)	0.952	0.999	1.25 (0.48-3.27)	0.652	0.992	0.83 (0.31-2.19)	0.703	0.975
STX8	1.26 (0.82-1.92)	0.290	0.809	0.93 (0.38-2.30)	0.875	0.997	1.27 (0.58-2.79)	0.549	0.955
STXBP1	1.00 (0.72-1.37)	0.980	0.999	1.10 (0.61-1.97)	0.759	0.997	0.61 (0.33-1.11)	0.106	0.763
STXBP3	0.85 (0.48-1.51)	0.585	0.960	0.37 (0.11-1.23)	0.104	0.528	0.86 (0.30-2.47)	0.786	0.989
SUGP1	0.98 (0.52-1.85)	0.949	0.999	1.34 (0.44-4.12)	0.604	0.975	0.87 (0.25-2.95)	0.818	0.991
SUGT1	0.91 (0.68-1.21)	0.526	0.948	0.77 (0.46-1.29)	0.320	0.824	0.79 (0.47-1.34)	0.381	0.942

SULT1A1	1.03 (0.86-1.23)	0.756	0.986	0.97 (0.71-1.34)	0.858	0.997	0.97 (0.70-1.36)	0.874	0.992
SULT2A1	0.94 (0.66-1.34)	0.734	0.980	0.65 (0.30-1.40)	0.272	0.783	0.99 (0.52-1.90)	0.984	0.996
SUMF1	0.95 (0.62-1.47)	0.821	0.995	1.42 (0.84-2.38)	0.189	0.682	1.06 (0.54-2.08)	0.856	0.992
SUMF2	1.20 (0.76-1.88)	0.437	0.907	1.43 (0.62-3.34)	0.403	0.902	1.16 (0.49-2.74)	0.737	0.983
SUOX	0.97 (0.66-1.42)	0.876	0.998	1.19 (0.61-2.29)	0.613	0.979	1.29 (0.67-2.49)	0.440	0.942
SUSD1	0.94 (0.58-1.52)	0.808	0.995	0.75 (0.32-1.78)	0.511	0.943	0.69 (0.29-1.65)	0.404	0.942
SUSD2	1.64 (0.86-3.16)	0.136	0.641	1.37 (0.40-4.72)	0.618	0.979	1.43 (0.41-4.95)	0.574	0.960
SUSD4	1.35 (0.86-2.12)	0.186	0.711	0.92 (0.38-2.24)	0.856	0.997	0.81 (0.33-2.03)	0.660	0.975
SUSD5	1.76 (0.94-3.28)	0.076	0.514	3.68 (1.33-10.21)	0.012	0.207	1.37 (0.45-4.17)	0.584	0.965
SV2A	1.26 (0.83-1.89)	0.274	0.800	0.64 (0.26-1.59)	0.338	0.842	1.78 (1.00-3.17)	0.048	0.664
SWAP70	0.87 (0.44-1.73)	0.696	0.975	1.77 (0.74-4.21)	0.198	0.703	0.89 (0.28-2.81)	0.842	0.992
SYAP1	0.75 (0.47-1.20)	0.230	0.762	1.20 (0.55-2.63)	0.645	0.991	0.60 (0.24-1.51)	0.274	0.910
SYNGAP1	1.02 (0.65-1.60)	0.932	0.999	1.44 (0.78-2.66)	0.242	0.744	0.57 (0.19-1.71)	0.318	0.923
SYT1	1.43 (1.02-2.02)	0.040	0.413	1.61 (0.93-2.81)	0.092	0.506	1.50 (0.96-2.35)	0.076	0.718
SYTL4	0.94 (0.65-1.35)	0.728	0.978	0.94 (0.48-1.86)	0.864	0.997	0.81 (0.40-1.61)	0.541	0.952
TAB2	1.10 (0.87-1.39)	0.413	0.894	1.21 (0.79-1.85)	0.375	0.879	1.02 (0.66-1.59)	0.916	0.992
TACC3	0.93 (0.77-1.14)	0.499	0.939	0.97 (0.68-1.39)	0.884	0.997	0.88 (0.61-1.27)	0.499	0.947
TACSTD2	1.80 (0.78-4.12)	0.167	0.693	2.64 (0.58-11.91)	0.207	0.712	2.59 (0.58-11.61)	0.215	0.868
TADA3	0.96 (0.50-1.81)	0.889	0.999	1.77 (0.58-5.40)	0.315	0.819	0.50 (0.15-1.65)	0.253	0.901
TAFA5	2.19 (1.39-3.44)	<0.001	0.060	2.77 (1.32-5.78)	0.007	0.160	1.82 (0.79-4.17)	0.160	0.834
TAGLN3	1.42 (1.10-1.82)	0.006	0.186	1.17 (0.62-2.23)	0.624	0.983	0.51 (0.22-1.21)	0.128	0.796
TALDO1	0.87 (0.52-1.48)	0.618	0.966	1.55 (0.66-3.68)	0.317	0.821	0.78 (0.30-2.05)	0.615	0.966
TANK	1.15 (0.83-1.58)	0.401	0.894	1.07 (0.59-1.95)	0.823	0.997	1.17 (0.63-2.17)	0.608	0.966
TAP1	1.17 (0.81-1.69)	0.404	0.894	0.73 (0.29-1.82)	0.495	0.940	0.63 (0.24-1.67)	0.353	0.938
TARBP2	1.00 (0.75-1.34)	0.993	0.999	0.96 (0.57-1.63)	0.887	0.997	0.95 (0.54-1.65)	0.849	0.992

TARM1	1.05 (0.75-1.45)	0.790	0.994	1.04 (0.57-1.88)	0.906	0.997	1.02 (0.54-1.90)	0.959	0.994
TARS1	1.12 (0.54-2.31)	0.765	0.987	1.98 (0.62-6.29)	0.247	0.753	0.59 (0.14-2.45)	0.472	0.944
TAX1BP1	0.97 (0.65-1.44)	0.867	0.998	0.80 (0.37-1.74)	0.576	0.965	1.12 (0.55-2.28)	0.764	0.987
TBC1D17	1.10 (0.76-1.61)	0.614	0.966	0.73 (0.33-1.61)	0.431	0.908	1.14 (0.61-2.13)	0.674	0.975
TBC1D23	0.97 (0.76-1.25)	0.817	0.995	0.83 (0.52-1.33)	0.444	0.914	0.92 (0.56-1.49)	0.727	0.978
TBC1D5	1.13 (0.86-1.48)	0.385	0.878	1.00 (0.59-1.71)	0.987	0.998	1.10 (0.66-1.81)	0.723	0.977
TBCA	0.97 (0.74-1.27)	0.825	0.995	1.19 (0.73-1.95)	0.493	0.940	0.90 (0.56-1.47)	0.687	0.975
TBCB	1.01 (0.80-1.27)	0.959	0.999	1.14 (0.74-1.76)	0.555	0.962	0.90 (0.59-1.37)	0.617	0.967
TBCC	1.04 (0.78-1.40)	0.780	0.992	1.08 (0.64-1.85)	0.770	0.997	1.08 (0.62-1.89)	0.782	0.987
TBL1X	0.95 (0.72-1.24)	0.705	0.975	0.88 (0.54-1.43)	0.595	0.972	0.83 (0.50-1.36)	0.453	0.942
TBR1	0.82 (0.47-1.43)	0.493	0.936	0.49 (0.12-1.97)	0.316	0.819	1.00 (0.46-2.19)	0.998	1.000
TCL1A	0.87 (0.70-1.08)	0.207	0.740	0.73 (0.49-1.08)	0.116	0.563	0.87 (0.60-1.26)	0.468	0.942
TCL1B	0.97 (0.73-1.30)	0.850	0.998	0.97 (0.57-1.65)	0.909	0.997	0.89 (0.50-1.56)	0.675	0.975
TCN1	2.61 (1.42-4.78)	0.002	0.107	2.91 (1.03-8.26)	0.044	0.363	1.77 (0.48-6.52)	0.394	0.942
TCN2	1.83 (0.91-3.68)	0.092	0.559	3.18 (0.96-10.54)	0.059	0.407	2.81 (0.80-9.94)	0.108	0.763
TCOF1	1.50 (0.86-2.64)	0.155	0.676	2.33 (1.13-4.83)	0.022	0.264	0.71 (0.18-2.82)	0.630	0.972
TCP11	0.93 (0.55-1.58)	0.799	0.994	1.11 (0.50-2.47)	0.790	0.997	0.74 (0.23-2.42)	0.619	0.967
TCTN3	0.98 (0.54-1.79)	0.943	0.999	1.71 (0.52-5.62)	0.375	0.879	1.04 (0.35-3.08)	0.941	0.992
TDGF1	1.22 (1.06-1.40)	0.005	0.159	1.28 (1.00-1.65)	0.054	0.392	1.22 (0.94-1.58)	0.131	0.796
TDO2	0.63 (0.28-1.43)	0.272	0.799	0.58 (0.13-2.60)	0.475	0.932	0.52 (0.12-2.23)	0.379	0.942
TDP1	1.16 (0.82-1.62)	0.401	0.894	1.26 (0.69-2.30)	0.456	0.923	0.84 (0.36-1.99)	0.696	0.975
TDRKH	1.06 (0.81-1.39)	0.670	0.971	1.08 (0.65-1.77)	0.771	0.997	1.31 (0.80-2.14)	0.286	0.915
TEF	1.34 (0.87-2.05)	0.187	0.712	1.41 (0.73-2.74)	0.308	0.816	0.84 (0.25-2.75)	0.767	0.987
TEK	2.69 (1.07-6.81)	0.036	0.402	2.92 (0.58-14.73)	0.195	0.697	3.02 (0.56-16.42)	0.201	0.867
TERF1	1.74 (0.88-3.42)	0.111	0.598	3.27 (1.55-6.90)	0.002	0.086	0.89 (0.15-5.36)	0.901	0.992

TET2	1.09 (0.88-1.36)	0.425	0.902	1.17 (0.82-1.66)	0.395	0.897	1.06 (0.70-1.61)	0.781	0.987
TEX101	1.10 (0.75-1.62)	0.630	0.967	1.29 (0.64-2.61)	0.476	0.932	0.93 (0.45-1.90)	0.838	0.991
TEX33	0.92 (0.47-1.79)	0.809	0.995	1.46 (0.68-3.15)	0.335	0.839	0.28 (0.06-1.35)	0.113	0.768
TF	1.38 (0.51-3.76)	0.526	0.948	2.09 (0.34-13.01)	0.430	0.908	2.75 (0.43-17.39)	0.284	0.915
TFAP2A	1.35 (0.92-1.98)	0.129	0.627	0.88 (0.36-2.17)	0.785	0.997	1.21 (0.57-2.56)	0.615	0.966
TFF1	1.26 (1.00-1.59)	0.054	0.452	1.31 (0.86-1.98)	0.208	0.712	1.50 (1.03-2.18)	0.035	0.623
TFF2	1.70 (1.21-2.38)	0.002	0.113	1.85 (1.01-3.40)	0.046	0.373	2.23 (1.26-3.93)	0.006	0.346
TFF3	1.41 (0.92-2.18)	0.118	0.609	1.42 (0.63-3.17)	0.394	0.896	1.65 (0.80-3.39)	0.174	0.853
TFPI	1.31 (0.65-2.65)	0.456	0.920	1.31 (0.39-4.41)	0.668	0.993	0.62 (0.17-2.29)	0.474	0.944
TFPI2	1.30 (0.95-1.79)	0.100	0.573	1.52 (0.97-2.39)	0.067	0.441	1.22 (0.71-2.10)	0.477	0.944
TFRC	1.20 (0.70-2.05)	0.517	0.947	1.26 (0.48-3.31)	0.645	0.991	1.06 (0.41-2.74)	0.901	0.992
TG	1.24 (1.02-1.51)	0.034	0.389	1.22 (0.85-1.73)	0.277	0.788	1.56 (1.16-2.11)	0.004	0.316
TGFA	1.46 (0.96-2.22)	0.078	0.514	1.33 (0.61-2.90)	0.474	0.932	1.99 (0.97-4.07)	0.061	0.698
TGFB1	0.96 (0.54-1.73)	0.897	0.999	1.75 (0.61-5.02)	0.297	0.804	1.17 (0.38-3.53)	0.787	0.989
TGFB2	1.42 (0.95-2.12)	0.086	0.538	1.59 (0.78-3.22)	0.200	0.707	1.21 (0.56-2.65)	0.626	0.969
TGFBI	0.97 (0.46-2.05)	0.942	0.999	2.46 (0.73-8.31)	0.148	0.621	0.57 (0.13-2.38)	0.437	0.942
TGFBR1	2.86 (1.04-7.86)	0.042	0.420	3.70 (0.72-19.04)	0.117	0.565	3.96 (0.83-18.86)	0.084	0.743
TGFBR2	1.90 (1.21-2.99)	0.006	0.179	2.20 (1.09-4.47)	0.029	0.295	1.75 (0.77-3.96)	0.182	0.857
TGFBR3	1.44 (0.73-2.84)	0.298	0.816	4.47 (1.52-13.09)	0.006	0.157	1.29 (0.37-4.53)	0.693	0.975
TGM2	1.21 (0.91-1.63)	0.195	0.720	0.81 (0.47-1.41)	0.461	0.924	1.33 (0.80-2.22)	0.271	0.910
TGOLN2	2.06 (0.99-4.26)	0.052	0.443	4.04 (1.38-11.84)	0.011	0.199	3.36 (1.02-11.01)	0.046	0.643
THAP12	0.68 (0.44-1.05)	0.083	0.528	0.94 (0.49-1.80)	0.859	0.997	0.38 (0.14-1.03)	0.058	0.698
THBD	1.12 (0.56-2.24)	0.749	0.985	2.93 (0.99-8.63)	0.052	0.391	0.48 (0.12-1.87)	0.289	0.915
THBS2	2.04 (1.32-3.16)	0.001	0.085	2.65 (1.29-5.44)	0.008	0.175	1.96 (0.87-4.39)	0.104	0.763
THBS4	1.46 (0.96-2.24)	0.078	0.514	2.35 (1.13-4.92)	0.023	0.267	1.78 (0.83-3.79)	0.136	0.796

THOP1	1.11 (0.60-2.04)	0.748	0.984	0.89 (0.29-2.70)	0.835	0.997	1.37 (0.50-3.76)	0.546	0.954
THPO	0.88 (0.49-1.59)	0.677	0.974	0.88 (0.30-2.63)	0.825	0.997	0.71 (0.24-2.12)	0.539	0.952
THRAP3	1.13 (1.00-1.28)	0.057	0.461	0.96 (0.70-1.30)	0.780	0.997	1.21 (0.99-1.49)	0.068	0.718
THSD1	0.99 (0.46-2.15)	0.987	0.999	0.83 (0.20-3.39)	0.794	0.997	0.81 (0.17-3.75)	0.787	0.989
THTPA	0.93 (0.70-1.24)	0.629	0.966	0.94 (0.56-1.58)	0.816	0.997	0.85 (0.51-1.42)	0.544	0.952
THY1	2.36 (1.29-4.30)	0.005	0.174	3.23 (1.17-8.90)	0.023	0.269	2.17 (0.75-6.26)	0.150	0.816
TIA1	0.92 (0.76-1.12)	0.411	0.894	0.96 (0.68-1.35)	0.793	0.997	0.87 (0.61-1.25)	0.458	0.942
TIE1	1.86 (0.92-3.75)	0.085	0.535	2.87 (0.71-11.51)	0.137	0.601	3.42 (0.77-15.13)	0.105	0.763
TIGAR	0.95 (0.56-1.60)	0.839	0.998	0.83 (0.31-2.17)	0.697	0.994	1.03 (0.41-2.62)	0.944	0.992
TIGIT	1.54 (1.02-2.34)	0.041	0.417	1.41 (0.59-3.41)	0.440	0.912	1.49 (0.72-3.06)	0.283	0.914
TIMD4	1.21 (0.76-1.92)	0.431	0.904	1.82 (0.85-3.87)	0.122	0.579	0.90 (0.37-2.19)	0.824	0.991
TIMM10	0.87 (0.44-1.72)	0.690	0.975	0.70 (0.19-2.51)	0.582	0.967	0.43 (0.11-1.65)	0.219	0.869
TIMM8A	0.83 (0.61-1.11)	0.212	0.743	0.99 (0.58-1.69)	0.969	0.997	0.59 (0.33-1.06)	0.080	0.740
TIMP1	1.17 (0.58-2.38)	0.656	0.971	2.09 (0.63-6.92)	0.225	0.736	0.94 (0.25-3.52)	0.925	0.992
TIMP2	2.13 (1.34-3.38)	0.001	0.085	3.52 (1.97-6.28)	<0.001	0.010	2.44 (1.31-4.55)	0.005	0.336
TIMP3	1.01 (0.85-1.20)	0.950	0.999	0.99 (0.72-1.36)	0.928	0.997	1.01 (0.74-1.38)	0.971	0.994
TIMP4	1.48 (0.90-2.42)	0.123	0.620	1.61 (0.64-4.06)	0.309	0.816	1.64 (0.68-3.95)	0.273	0.910
TINAGL1	2.00 (0.76-5.27)	0.162	0.683	9.33 (1.61-53.92)	0.013	0.211	0.96 (0.16-5.82)	0.964	0.994
TJAP1	1.01 (0.77-1.31)	0.964	0.999	0.99 (0.61-1.61)	0.957	0.997	0.82 (0.49-1.39)	0.461	0.942
TJP3	0.71 (0.37-1.39)	0.319	0.836	0.40 (0.10-1.54)	0.183	0.668	0.53 (0.15-1.92)	0.336	0.934
TK1	1.16 (0.74-1.84)	0.515	0.946	1.47 (0.78-2.74)	0.229	0.739	0.80 (0.28-2.28)	0.672	0.975
TLR1	1.25 (0.48-3.24)	0.648	0.971	2.61 (0.53-12.95)	0.239	0.742	0.97 (0.17-5.63)	0.975	0.994
TLR2	1.38 (0.46-4.20)	0.567	0.956	2.79 (0.45-17.18)	0.270	0.779	0.67 (0.08-5.62)	0.708	0.976
TLR3	1.16 (0.79-1.71)	0.453	0.916	0.86 (0.45-1.64)	0.652	0.992	0.96 (0.49-1.85)	0.897	0.992
TLR4	0.73 (0.28-1.92)	0.523	0.948	3.96 (0.88-17.88)	0.074	0.464	0.23 (0.04-1.47)	0.120	0.783

TMCO5A	0.94 (0.58-1.53)	0.798	0.994	1.29 (0.67-2.50)	0.444	0.914	0.21 (0.06-0.71)	0.012	0.433
TMED1	0.87 (0.54-1.42)	0.581	0.957	0.94 (0.40-2.23)	0.893	0.997	1.84 (0.98-3.47)	0.058	0.698
TMED10	1.14 (0.67-1.94)	0.626	0.966	0.53 (0.12-2.40)	0.413	0.903	0.55 (0.13-2.35)	0.423	0.942
TMED4	1.47 (1.08-2.00)	0.014	0.286	1.69 (1.10-2.60)	0.017	0.235	0.61 (0.18-2.14)	0.444	0.942
TMED8	0.97 (0.77-1.22)	0.780	0.992	1.03 (0.68-1.55)	0.902	0.997	0.87 (0.57-1.33)	0.519	0.948
TMEM106A	0.89 (0.61-1.29)	0.541	0.948	1.14 (0.62-2.11)	0.677	0.993	0.53 (0.24-1.13)	0.100	0.762
TMEM132A	1.12 (0.76-1.64)	0.578	0.956	0.74 (0.36-1.56)	0.432	0.908	1.49 (0.90-2.47)	0.119	0.783
TMEM25	0.95 (0.59-1.54)	0.849	0.998	0.79 (0.31-2.06)	0.633	0.986	1.11 (0.48-2.56)	0.808	0.991
TMOD4	1.33 (0.80-2.21)	0.268	0.796	1.51 (0.66-3.43)	0.329	0.832	2.17 (0.89-5.27)	0.089	0.745
TMPRSS11B	1.06 (0.80-1.40)	0.668	0.971	1.11 (0.67-1.84)	0.673	0.993	0.83 (0.44-1.57)	0.569	0.960
TMPRSS11D	1.72 (1.01-2.92)	0.044	0.425	1.33 (0.49-3.61)	0.570	0.963	1.88 (0.75-4.73)	0.179	0.853
TMPRSS15	1.12 (0.88-1.44)	0.351	0.856	1.11 (0.71-1.74)	0.639	0.988	1.60 (1.00-2.55)	0.050	0.670
TMPRSS5	1.60 (0.87-2.92)	0.128	0.626	0.94 (0.33-2.69)	0.908	0.997	0.88 (0.31-2.53)	0.814	0.991
TMSB10	1.03 (0.75-1.41)	0.867	0.998	1.41 (0.79-2.50)	0.248	0.753	0.87 (0.47-1.60)	0.652	0.975
TNC	1.17 (0.76-1.80)	0.484	0.933	0.83 (0.38-1.82)	0.639	0.989	1.61 (0.75-3.44)	0.222	0.874
TNF	1.22 (0.71-2.08)	0.472	0.924	1.89 (0.89-3.99)	0.098	0.520	0.67 (0.18-2.43)	0.537	0.952
TNFAIP2	1.10 (0.67-1.82)	0.701	0.975	1.63 (0.70-3.78)	0.260	0.772	1.33 (0.58-3.06)	0.496	0.947
TNFAIP8	0.88 (0.48-1.61)	0.670	0.971	1.60 (0.69-3.73)	0.273	0.783	0.68 (0.22-2.12)	0.510	0.947
TNFAIP8L2	0.75 (0.49-1.14)	0.179	0.706	0.98 (0.46-2.06)	0.952	0.997	0.53 (0.22-1.24)	0.144	0.808
TNFRSF10A	2.65 (1.53-4.58)	<0.001	0.048	3.11 (1.19-8.13)	0.020	0.256	4.13 (1.60-10.66)	0.003	0.306
TNFRSF10B	1.71 (1.27-2.31)	<0.001	0.046	1.74 (0.98-3.08)	0.057	0.404	2.04 (1.24-3.37)	0.005	0.343
TNFRSF10C	1.39 (0.86-2.25)	0.184	0.708	1.71 (0.70-4.20)	0.240	0.742	1.11 (0.47-2.63)	0.815	0.991
TNFRSF11A	1.58 (1.01-2.47)	0.047	0.428	1.57 (0.71-3.50)	0.267	0.777	1.32 (0.57-3.10)	0.518	0.948
TNFRSF11B	1.65 (0.83-3.32)	0.156	0.676	8.16 (2.53-26.32)	<0.001	0.050	1.17 (0.32-4.27)	0.813	0.991
TNFRSF12A	2.67 (1.69-4.22)	<0.001	0.009	2.46 (1.08-5.63)	0.032	0.314	3.22 (1.54-6.73)	0.002	0.228

TNFRSF13B	2.23 (1.23-4.05)	0.009	0.220	3.65 (1.24-10.75)	0.019	0.247	1.21 (0.38-3.83)	0.748	0.985
TNFRSF13C	1.12 (0.67-1.85)	0.668	0.971	1.19 (0.46-3.05)	0.722	0.995	0.70 (0.25-1.91)	0.484	0.944
TNFRSF14	1.54 (0.82-2.89)	0.180	0.706	1.84 (0.64-5.33)	0.260	0.772	1.23 (0.38-4.03)	0.731	0.978
TNFRSF17	1.57 (1.01-2.44)	0.046	0.428	2.35 (1.08-5.13)	0.032	0.314	1.39 (0.61-3.19)	0.438	0.942
TNFRSF19	1.92 (1.22-3.01)	0.005	0.165	2.02 (0.96-4.25)	0.064	0.428	2.19 (1.03-4.63)	0.041	0.630
TNFRSF1A	1.97 (1.16-3.35)	0.012	0.260	2.51 (1.07-5.88)	0.035	0.323	2.21 (0.89-5.48)	0.086	0.745
TNFRSF1B	1.35 (0.89-2.06)	0.159	0.679	1.86 (1.05-3.30)	0.034	0.319	1.23 (0.49-3.08)	0.653	0.975
TNFRSF21	1.69 (0.85-3.38)	0.134	0.639	2.66 (0.88-8.10)	0.084	0.486	1.27 (0.34-4.73)	0.725	0.978
TNFRSF4	1.83 (1.15-2.94)	0.012	0.259	3.95 (1.90-8.20)	<0.001	0.042	1.69 (0.70-4.07)	0.246	0.897
TNFRSF6B	1.56 (1.13-2.17)	0.007	0.195	2.02 (1.19-3.41)	0.009	0.187	1.28 (0.69-2.35)	0.435	0.942
TNFRSF8	1.26 (0.78-2.04)	0.341	0.854	1.90 (0.85-4.23)	0.117	0.564	0.48 (0.18-1.29)	0.147	0.813
TNFRSF9	1.59 (1.02-2.48)	0.041	0.416	2.53 (1.33-4.82)	0.005	0.145	1.09 (0.45-2.64)	0.845	0.992
TNFSF10	1.24 (0.53-2.89)	0.619	0.966	4.00 (0.86-18.61)	0.078	0.471	0.27 (0.06-1.26)	0.096	0.762
TNFSF11	0.95 (0.66-1.37)	0.788	0.994	1.38 (0.70-2.72)	0.346	0.854	0.44 (0.23-0.87)	0.018	0.512
TNFSF12	0.42 (0.19-0.92)	0.031	0.372	1.06 (0.25-4.50)	0.941	0.997	0.19 (0.04-0.89)	0.035	0.623
TNFSF13	2.08 (0.96-4.54)	0.065	0.473	2.12 (0.51-8.79)	0.301	0.809	3.66 (0.97-13.85)	0.056	0.698
TNFSF13B	2.07 (1.05-4.09)	0.036	0.400	2.49 (0.77-8.11)	0.130	0.590	2.42 (0.74-7.87)	0.142	0.808
TNFSF14	1.01 (0.67-1.51)	0.969	0.999	0.85 (0.40-1.81)	0.676	0.993	0.86 (0.39-1.88)	0.704	0.975
TNFSF8	1.38 (0.82-2.33)	0.225	0.757	1.81 (0.86-3.78)	0.116	0.563	0.75 (0.23-2.52)	0.646	0.973
TNIP1	0.94 (0.71-1.24)	0.664	0.971	0.94 (0.56-1.57)	0.802	0.997	0.86 (0.50-1.48)	0.593	0.965
TNN	1.58 (0.88-2.85)	0.124	0.623	2.63 (0.92-7.49)	0.070	0.454	0.97 (0.33-2.80)	0.948	0.992
TNNI3	1.13 (0.85-1.52)	0.399	0.894	1.19 (0.68-2.09)	0.539	0.955	0.96 (0.60-1.53)	0.862	0.992
TNPO1	1.14 (0.59-2.21)	0.693	0.975	1.43 (0.43-4.82)	0.562	0.962	0.36 (0.11-1.22)	0.101	0.762
TNR	1.67 (0.98-2.86)	0.061	0.471	3.39 (1.21-9.47)	0.020	0.254	0.90 (0.33-2.46)	0.843	0.992
TNXB	1.06 (0.48-2.35)	0.889	0.999	21.41 (4.58-100.00)	<0.001	0.024	0.37 (0.09-1.47)	0.157	0.825

TOMM20	0.81 (0.56-1.16)	0.250	0.775	1.06 (0.57-1.98)	0.858	0.997	0.58 (0.28-1.23)	0.155	0.822
TOP1	0.96 (0.76-1.22)	0.755	0.986	1.21 (0.78-1.86)	0.394	0.896	0.94 (0.62-1.44)	0.787	0.989
TOP1MT	0.86 (0.49-1.52)	0.603	0.962	1.55 (0.83-2.91)	0.172	0.657	0.74 (0.26-2.13)	0.581	0.964
TOP2B	0.90 (0.64-1.28)	0.562	0.956	1.29 (0.73-2.28)	0.388	0.891	0.62 (0.31-1.25)	0.181	0.853
TOR1AIP1	0.92 (0.52-1.63)	0.783	0.994	1.42 (0.58-3.45)	0.444	0.914	0.69 (0.23-2.09)	0.516	0.947
TP53	0.83 (0.47-1.45)	0.506	0.943	0.73 (0.25-2.10)	0.558	0.962	0.54 (0.19-1.59)	0.264	0.910
TP53BP1	0.96 (0.71-1.31)	0.797	0.994	0.75 (0.41-1.39)	0.363	0.865	1.06 (0.61-1.86)	0.836	0.991
TP53I3	0.90 (0.56-1.43)	0.650	0.971	1.23 (0.55-2.77)	0.610	0.978	0.73 (0.31-1.71)	0.468	0.942
TP53INP1	1.29 (0.64-2.58)	0.476	0.928	0.59 (0.10-3.35)	0.550	0.960	1.84 (0.62-5.48)	0.273	0.910
TP73	1.34 (0.66-2.70)	0.414	0.894	0.97 (0.27-3.45)	0.968	0.997	0.99 (0.28-3.55)	0.987	0.997
TPBGL	0.79 (0.43-1.45)	0.440	0.909	0.28 (0.07-1.13)	0.074	0.465	0.67 (0.20-2.23)	0.519	0.948
TPD52L2	0.89 (0.70-1.13)	0.333	0.848	0.92 (0.60-1.41)	0.695	0.994	0.65 (0.41-1.03)	0.065	0.710
TPK1	0.75 (0.31-1.85)	0.535	0.948	3.70 (0.83-16.51)	0.086	0.491	1.88 (0.39-9.10)	0.434	0.942
TPM3	1.13 (0.66-1.93)	0.655	0.971	2.59 (1.15-5.83)	0.021	0.261	0.49 (0.17-1.46)	0.203	0.867
TPMT	0.79 (0.59-1.06)	0.123	0.620	0.85 (0.49-1.46)	0.555	0.962	0.81 (0.47-1.40)	0.457	0.942
TPP1	1.05 (0.65-1.68)	0.852	0.998	1.18 (0.54-2.60)	0.673	0.993	0.92 (0.37-2.31)	0.863	0.992
TPPP2	0.73 (0.52-1.03)	0.075	0.512	0.72 (0.38-1.36)	0.316	0.819	0.27 (0.11-0.63)	0.003	0.281
TPPP3	1.01 (0.59-1.73)	0.976	0.999	1.40 (0.71-2.78)	0.334	0.839	1.17 (0.45-3.05)	0.753	0.985
TPR	1.06 (0.61-1.84)	0.832	0.997	1.69 (0.86-3.29)	0.126	0.587	0.89 (0.30-2.59)	0.825	0.991
TPRKB	0.99 (0.66-1.48)	0.955	0.999	1.26 (0.80-1.98)	0.311	0.816	0.40 (0.13-1.27)	0.120	0.783
TPSAB1	0.63 (0.41-0.97)	0.036	0.400	0.54 (0.26-1.12)	0.099	0.524	0.86 (0.38-1.91)	0.705	0.975
TPSD1	1.02 (0.48-2.17)	0.958	0.999	1.30 (0.38-4.44)	0.673	0.993	1.87 (0.69-5.05)	0.218	0.869
TPSG1	1.09 (0.48-2.46)	0.835	0.997	1.51 (0.36-6.36)	0.578	0.966	0.37 (0.07-1.79)	0.214	0.867
TPT1	0.68 (0.43-1.07)	0.097	0.569	0.90 (0.44-1.84)	0.766	0.997	1.08 (0.59-1.97)	0.811	0.991
TRAF2	1.14 (0.84-1.55)	0.408	0.894	0.92 (0.52-1.62)	0.773	0.997	1.30 (0.75-2.25)	0.345	0.936

TRAF3	0.85 (0.40-1.82)	0.681	0.975	1.03 (0.28-3.71)	0.968	0.997	0.70 (0.18-2.73)	0.611	0.966
TRAF3IP2	0.64 (0.32-1.28)	0.208	0.740	0.87 (0.32-2.38)	0.793	0.997	0.58 (0.16-2.11)	0.407	0.942
TRDMT1	1.01 (0.74-1.38)	0.946	0.999	0.95 (0.54-1.68)	0.862	0.997	1.11 (0.65-1.89)	0.714	0.976
TREH	0.97 (0.72-1.32)	0.850	0.998	1.06 (0.61-1.86)	0.838	0.997	0.81 (0.47-1.40)	0.449	0.942
TREM2	1.01 (0.67-1.51)	0.980	0.999	0.66 (0.31-1.40)	0.279	0.789	1.06 (0.50-2.21)	0.884	0.992
TREML1	0.81 (0.48-1.36)	0.420	0.899	1.26 (0.53-3.03)	0.602	0.973	0.52 (0.18-1.51)	0.229	0.879
TREML2	1.41 (0.73-2.72)	0.305	0.822	2.16 (0.67-6.99)	0.200	0.707	0.93 (0.28-3.14)	0.910	0.992
TRIAP1	1.01 (0.70-1.45)	0.957	0.999	1.09 (0.57-2.10)	0.794	0.997	0.71 (0.34-1.46)	0.347	0.936
TRIM21	1.09 (0.81-1.47)	0.570	0.956	1.05 (0.60-1.83)	0.868	0.997	1.12 (0.64-1.95)	0.692	0.975
TRIM24	1.06 (0.73-1.55)	0.756	0.986	1.42 (0.76-2.66)	0.269	0.778	1.54 (0.78-3.02)	0.210	0.867
TRIM25	0.99 (0.79-1.25)	0.924	0.999	1.17 (0.77-1.78)	0.469	0.927	0.90 (0.58-1.39)	0.633	0.973
TRIM26	1.19 (0.89-1.60)	0.241	0.769	1.31 (0.82-2.08)	0.257	0.771	0.95 (0.50-1.79)	0.867	0.992
TRIM40	0.97 (0.69-1.38)	0.872	0.998	0.98 (0.53-1.83)	0.955	0.997	0.76 (0.38-1.51)	0.434	0.942
TRIM5	1.03 (0.79-1.34)	0.844	0.998	1.11 (0.68-1.82)	0.672	0.993	0.95 (0.57-1.59)	0.857	0.992
TRIM58	0.93 (0.69-1.25)	0.630	0.966	0.84 (0.49-1.42)	0.504	0.942	0.89 (0.52-1.53)	0.686	0.975
TRPV3	0.99 (0.59-1.63)	0.956	0.999	1.28 (0.62-2.66)	0.500	0.941	1.40 (0.64-3.08)	0.396	0.942
TSC1	1.18 (0.86-1.61)	0.303	0.821	1.20 (0.68-2.12)	0.538	0.954	1.28 (0.69-2.36)	0.428	0.942
TSC22D1	0.88 (0.60-1.31)	0.538	0.948	1.19 (0.62-2.31)	0.598	0.973	0.54 (0.23-1.29)	0.166	0.848
TSHB	1.01 (0.79-1.29)	0.920	0.999	0.84 (0.53-1.33)	0.456	0.923	1.23 (0.81-1.85)	0.328	0.932
TSLP	1.19 (0.89-1.58)	0.237	0.767	1.24 (0.78-1.98)	0.359	0.862	1.22 (0.73-2.04)	0.445	0.942
TSNAX	1.17 (0.63-2.16)	0.618	0.966	1.54 (0.55-4.32)	0.408	0.903	1.69 (0.62-4.58)	0.306	0.923
TSPAN1	0.81 (0.54-1.21)	0.298	0.816	1.12 (0.63-2.00)	0.697	0.994	0.69 (0.32-1.46)	0.328	0.932
TSPAN15	1.19 (0.82-1.72)	0.358	0.863	1.30 (0.72-2.37)	0.385	0.888	1.00 (0.46-2.17)	0.993	0.998
TSPAN7	0.97 (0.69-1.36)	0.856	0.998	1.08 (0.63-1.87)	0.781	0.997	0.83 (0.41-1.68)	0.613	0.966
TSPAN8	0.84 (0.65-1.07)	0.161	0.680	1.25 (0.81-1.93)	0.311	0.816	0.63 (0.39-1.02)	0.059	0.698

TSPYL1	1.16 (0.64-2.11)	0.623	0.966	2.00 (0.73-5.47)	0.177	0.662	1.29 (0.42-3.92)	0.658	0.975
TST	0.88 (0.65-1.21)	0.439	0.909	0.96 (0.54-1.69)	0.881	0.997	0.78 (0.43-1.43)	0.429	0.942
TTF2	0.89 (0.49-1.64)	0.719	0.975	1.02 (0.34-3.03)	0.970	0.997	0.53 (0.16-1.74)	0.297	0.917
TTN	1.30 (0.94-1.79)	0.116	0.607	1.36 (0.77-2.43)	0.294	0.803	1.27 (0.65-2.48)	0.480	0.944
TTR	0.80 (0.32-1.95)	0.618	0.966	1.03 (0.20-5.32)	0.976	0.997	1.05 (0.20-5.63)	0.953	0.992
TUBB3	1.40 (0.51-3.85)	0.516	0.946	3.87 (0.62-24.00)	0.147	0.617	0.47 (0.08-2.88)	0.415	0.942
TWF2	0.82 (0.61-1.10)	0.179	0.706	1.27 (0.77-2.09)	0.346	0.853	0.51 (0.27-0.95)	0.033	0.613
TXK	1.24 (0.97-1.60)	0.088	0.546	1.46 (1.02-2.08)	0.038	0.337	1.48 (0.99-2.22)	0.057	0.698
TXLNA	1.05 (0.81-1.35)	0.707	0.975	1.13 (0.71-1.80)	0.602	0.973	1.03 (0.64-1.68)	0.895	0.992
TXN	1.14 (0.70-1.86)	0.604	0.962	1.30 (0.61-2.77)	0.498	0.941	1.05 (0.43-2.54)	0.919	0.992
TXNDC15	1.74 (0.80-3.79)	0.163	0.683	13.98 (3.09-63.25)	<0.001	0.059	0.83 (0.18-3.76)	0.811	0.991
TXNDC5	1.01 (0.72-1.42)	0.950	0.999	1.08 (0.58-2.00)	0.810	0.997	0.84 (0.43-1.66)	0.617	0.967
TXNDC9	0.80 (0.48-1.34)	0.404	0.894	1.08 (0.46-2.54)	0.854	0.997	0.54 (0.18-1.63)	0.277	0.910
TXNL1	1.04 (0.78-1.37)	0.792	0.994	1.28 (0.86-1.91)	0.228	0.738	0.63 (0.28-1.43)	0.269	0.910
TXNRD1	0.84 (0.51-1.40)	0.506	0.943	1.05 (0.43-2.59)	0.910	0.997	1.04 (0.41-2.60)	0.935	0.992
TYMP	0.95 (0.66-1.36)	0.766	0.987	1.46 (0.76-2.80)	0.260	0.772	0.72 (0.36-1.45)	0.361	0.939
TYRO3	1.70 (0.78-3.73)	0.181	0.708	3.72 (0.93-14.86)	0.063	0.428	0.40 (0.09-1.77)	0.228	0.878
TYRP1	1.53 (1.00-2.34)	0.052	0.443	1.95 (0.94-4.06)	0.073	0.460	2.19 (1.04-4.61)	0.039	0.625
UBAC1	1.21 (0.64-2.28)	0.567	0.956	0.66 (0.20-2.17)	0.491	0.939	1.15 (0.38-3.53)	0.801	0.991
UBE2B	1.68 (0.74-3.83)	0.218	0.746	1.84 (0.43-7.85)	0.410	0.903	1.22 (0.27-5.59)	0.797	0.990
UBE2L6	0.99 (0.65-1.51)	0.969	0.999	2.03 (0.99-4.17)	0.053	0.392	0.94 (0.43-2.08)	0.881	0.992
UBE2Z	0.90 (0.56-1.45)	0.671	0.971	1.08 (0.49-2.37)	0.857	0.997	0.73 (0.27-1.98)	0.532	0.951
UBQLN3	0.92 (0.59-1.45)	0.728	0.978	1.55 (0.96-2.52)	0.075	0.466	1.01 (0.48-2.13)	0.972	0.994
UBXN1	0.90 (0.64-1.29)	0.574	0.956	1.15 (0.63-2.12)	0.649	0.992	0.88 (0.47-1.66)	0.690	0.975
UFD1	0.92 (0.77-1.10)	0.368	0.871	1.10 (0.80-1.53)	0.546	0.959	0.79 (0.56-1.10)	0.165	0.848

UGDH	1.12 (0.91-1.38)	0.291	0.809	1.08 (0.72-1.62)	0.723	0.995	1.23 (0.85-1.78)	0.274	0.910
UHRF2	0.59 (0.24-1.46)	0.255	0.782	0.84 (0.21-3.38)	0.806	0.997	0.58 (0.11-3.17)	0.531	0.951
ULBP2	2.08 (1.28-3.40)	0.003	0.133	4.26 (1.92-9.44)	<0.001	0.047	2.56 (1.09-5.99)	0.031	0.603
UMOD	0.80 (0.55-1.16)	0.234	0.765	0.75 (0.39-1.44)	0.382	0.886	0.62 (0.33-1.16)	0.138	0.802
UNC5D	2.86 (1.22-6.74)	0.016	0.294	6.72 (1.93-23.40)	0.003	0.110	0.90 (0.14-5.75)	0.910	0.992
UNC79	0.84 (0.50-1.39)	0.492	0.936	1.49 (0.71-3.15)	0.295	0.803	0.48 (0.16-1.44)	0.190	0.862
UNG	0.33 (0.13-0.81)	0.016	0.294	0.42 (0.08-2.24)	0.307	0.816	0.13 (0.02-0.78)	0.026	0.582
UPB1	0.83 (0.63-1.09)	0.172	0.697	0.92 (0.58-1.47)	0.741	0.997	0.73 (0.44-1.22)	0.233	0.884
UPK3A	1.28 (0.83-1.98)	0.261	0.788	1.56 (0.82-2.95)	0.172	0.657	1.54 (0.77-3.09)	0.220	0.869
UPK3BL1	0.81 (0.48-1.39)	0.449	0.916	0.78 (0.28-2.14)	0.630	0.984	1.14 (0.62-2.09)	0.667	0.975
UROD	0.90 (0.62-1.30)	0.569	0.956	0.89 (0.45-1.73)	0.726	0.996	0.88 (0.46-1.71)	0.712	0.976
UROS	0.88 (0.64-1.21)	0.424	0.902	0.89 (0.50-1.57)	0.683	0.993	1.13 (0.67-1.90)	0.652	0.975
USO1	1.00 (0.78-1.28)	0.974	0.999	1.01 (0.65-1.58)	0.955	0.997	1.02 (0.63-1.65)	0.929	0.992
USP25	0.92 (0.65-1.30)	0.635	0.968	1.25 (0.68-2.30)	0.466	0.926	0.95 (0.51-1.77)	0.884	0.992
USP28	1.31 (0.92-1.87)	0.137	0.641	1.31 (0.69-2.49)	0.415	0.903	1.40 (0.73-2.68)	0.309	0.923
USP47	1.18 (0.88-1.56)	0.267	0.796	1.57 (1.07-2.32)	0.022	0.261	0.60 (0.27-1.37)	0.227	0.878
USP8	0.98 (0.76-1.25)	0.845	0.998	0.98 (0.62-1.55)	0.941	0.997	0.84 (0.52-1.38)	0.499	0.947
UXS1	0.77 (0.50-1.20)	0.246	0.771	0.54 (0.23-1.28)	0.161	0.639	0.69 (0.32-1.49)	0.339	0.935
VAMP5	1.25 (0.90-1.75)	0.185	0.710	1.45 (0.89-2.34)	0.133	0.598	0.96 (0.38-2.43)	0.939	0.992
VAMP8	0.93 (0.68-1.28)	0.668	0.971	1.07 (0.61-1.86)	0.815	0.997	0.77 (0.42-1.42)	0.404	0.942
VASH1	0.91 (0.69-1.20)	0.499	0.939	0.97 (0.59-1.59)	0.896	0.997	0.92 (0.54-1.56)	0.760	0.987
VASN	1.01 (0.47-2.17)	0.982	0.999	2.15 (0.58-7.98)	0.253	0.761	0.57 (0.14-2.35)	0.439	0.942
VASP	0.88 (0.63-1.24)	0.467	0.923	1.13 (0.63-2.02)	0.684	0.993	0.68 (0.34-1.32)	0.252	0.901
VAT1	0.73 (0.24-2.18)	0.571	0.956	1.28 (0.17-9.53)	0.812	0.997	1.15 (0.15-8.72)	0.894	0.992
VAV3	1.04 (0.81-1.34)	0.737	0.982	1.19 (0.75-1.87)	0.463	0.926	0.93 (0.58-1.50)	0.780	0.987

VCAM1	1.27 (0.60-2.70)	0.534	0.948	2.61 (0.77-8.82)	0.122	0.579	0.89 (0.22-3.66)	0.871	0.992
VCAN	1.72 (0.83-3.56)	0.143	0.661	0.73 (0.18-2.87)	0.647	0.992	2.67 (0.77-9.32)	0.123	0.791
VCPKMT	1.07 (0.86-1.35)	0.542	0.948	0.88 (0.53-1.49)	0.642	0.990	0.85 (0.50-1.44)	0.543	0.952
VEGFA	1.25 (0.85-1.83)	0.259	0.787	1.54 (0.77-3.09)	0.220	0.729	1.17 (0.57-2.40)	0.673	0.975
VEGFB	1.98 (0.81-4.79)	0.132	0.632	5.50 (1.43-21.12)	0.013	0.213	1.12 (0.21-6.01)	0.893	0.992
VEGFC	0.91 (0.61-1.35)	0.642	0.970	1.18 (0.57-2.45)	0.661	0.992	0.61 (0.29-1.30)	0.199	0.867
VEGFD	1.00 (0.56-1.79)	0.992	0.999	1.53 (0.52-4.52)	0.439	0.912	1.18 (0.42-3.29)	0.752	0.985
VGF	1.00 (0.47-2.09)	0.990	0.999	1.20 (0.33-4.41)	0.779	0.997	2.18 (0.64-7.42)	0.212	0.867
VIM	1.09 (0.90-1.32)	0.374	0.874	0.94 (0.67-1.32)	0.720	0.995	1.38 (0.95-1.98)	0.087	0.745
VIPR1	1.34 (0.56-3.20)	0.504	0.943	1.35 (0.30-5.95)	0.694	0.994	1.28 (0.23-7.20)	0.777	0.987
VIT	1.53 (0.78-2.98)	0.216	0.746	3.61 (1.16-11.25)	0.027	0.284	1.11 (0.31-3.99)	0.870	0.992
VMO1	1.24 (0.88-1.74)	0.218	0.746	1.51 (0.79-2.89)	0.208	0.712	1.42 (0.74-2.71)	0.289	0.915
VNN1	0.97 (0.69-1.37)	0.870	0.998	1.25 (0.64-2.43)	0.518	0.945	1.02 (0.54-1.95)	0.950	0.992
VNN2	1.35 (0.81-2.24)	0.252	0.777	1.08 (0.45-2.63)	0.860	0.997	1.82 (0.72-4.64)	0.208	0.867
VPS28	0.82 (0.55-1.22)	0.329	0.846	1.04 (0.52-2.07)	0.917	0.997	0.61 (0.27-1.37)	0.229	0.879
VPS37A	0.98 (0.74-1.30)	0.902	0.999	1.02 (0.61-1.70)	0.952	0.997	0.93 (0.54-1.59)	0.788	0.989
VPS4B	0.83 (0.63-1.11)	0.215	0.746	0.97 (0.58-1.61)	0.901	0.997	0.62 (0.36-1.06)	0.083	0.743
VPS53	0.96 (0.72-1.28)	0.780	0.992	1.09 (0.65-1.83)	0.746	0.997	0.81 (0.46-1.44)	0.479	0.944
VSIG10	0.92 (0.43-1.97)	0.833	0.997	1.24 (0.34-4.48)	0.747	0.997	0.86 (0.20-3.67)	0.840	0.991
VSIG10L	1.67 (1.05-2.66)	0.030	0.372	2.16 (1.12-4.20)	0.022	0.264	1.78 (0.80-3.98)	0.158	0.826
VSIG2	1.48 (1.12-1.97)	0.006	0.186	1.62 (1.00-2.64)	0.051	0.391	1.97 (1.24-3.14)	0.004	0.316
VSIG4	1.31 (0.85-1.99)	0.217	0.746	1.16 (0.53-2.52)	0.705	0.994	1.35 (0.63-2.91)	0.442	0.942
VSIR	1.08 (0.76-1.54)	0.652	0.971	1.24 (0.68-2.28)	0.482	0.934	0.76 (0.37-1.56)	0.450	0.942
VSNL1	0.95 (0.51-1.78)	0.882	0.999	1.55 (0.54-4.46)	0.419	0.903	1.00 (0.32-3.14)	0.995	0.998
VSTM1	0.76 (0.47-1.21)	0.247	0.771	0.78 (0.34-1.81)	0.565	0.963	0.61 (0.25-1.48)	0.279	0.910

VSTM2B	1.93 (1.24-3.00)	0.004	0.139	1.81 (0.76-4.30)	0.181	0.667	2.47 (1.23-4.96)	0.011	0.429
VSTM2L	1.11 (0.78-1.59)	0.570	0.956	1.51 (0.96-2.38)	0.072	0.459	0.83 (0.38-1.82)	0.649	0.973
VTA1	1.08 (0.78-1.50)	0.623	0.966	1.22 (0.67-2.22)	0.509	0.942	1.02 (0.55-1.90)	0.953	0.992
VTCN1	1.07 (0.51-2.25)	0.848	0.998	1.51 (0.40-5.72)	0.548	0.960	0.94 (0.23-3.86)	0.932	0.992
VTI1A	1.08 (0.68-1.72)	0.735	0.981	1.15 (0.49-2.68)	0.755	0.997	1.28 (0.56-2.94)	0.554	0.955
VWA1	0.98 (0.58-1.65)	0.935	0.999	2.18 (0.89-5.32)	0.087	0.491	1.13 (0.46-2.79)	0.788	0.989
VWA5A	1.09 (0.82-1.45)	0.538	0.948	1.13 (0.68-1.88)	0.646	0.991	1.08 (0.66-1.76)	0.764	0.987
VWC2	1.47 (0.89-2.44)	0.132	0.632	2.11 (0.86-5.16)	0.102	0.527	2.24 (0.92-5.44)	0.075	0.718
VWC2L	0.93 (0.68-1.28)	0.652	0.971	0.45 (0.15-1.35)	0.154	0.627	1.15 (0.73-1.82)	0.536	0.952
VWF	1.03 (0.76-1.38)	0.863	0.998	0.68 (0.39-1.18)	0.171	0.656	1.57 (0.96-2.58)	0.071	0.718
WARS	1.26 (0.70-2.27)	0.434	0.906	1.99 (0.68-5.85)	0.210	0.713	2.08 (0.69-6.25)	0.195	0.866
WAS	0.95 (0.68-1.31)	0.739	0.982	0.99 (0.55-1.78)	0.984	0.998	0.84 (0.44-1.63)	0.613	0.966
WASF1	1.01 (0.79-1.30)	0.927	0.999	1.08 (0.68-1.69)	0.754	0.997	1.05 (0.65-1.68)	0.841	0.992
WASF3	0.94 (0.72-1.24)	0.680	0.975	0.97 (0.59-1.60)	0.904	0.997	0.92 (0.55-1.52)	0.739	0.983
WASHC3	0.97 (0.69-1.36)	0.861	0.998	1.12 (0.61-2.05)	0.723	0.995	1.05 (0.56-1.97)	0.876	0.992
WASL	1.09 (0.90-1.31)	0.390	0.884	0.89 (0.57-1.39)	0.615	0.979	1.10 (0.78-1.54)	0.593	0.965
WDR46	0.77 (0.36-1.67)	0.512	0.945	1.16 (0.36-3.70)	0.800	0.997	0.31 (0.06-1.44)	0.133	0.796
WFDC1	1.26 (0.89-1.79)	0.192	0.718	1.53 (0.94-2.50)	0.089	0.494	1.29 (0.65-2.60)	0.467	0.942
WFDC12	1.27 (0.92-1.76)	0.151	0.673	1.64 (0.94-2.86)	0.080	0.479	1.79 (1.02-3.14)	0.041	0.630
WFDC2	1.33 (0.86-2.06)	0.197	0.722	1.27 (0.57-2.79)	0.559	0.962	1.87 (0.98-3.56)	0.057	0.698
WFIKKN1	0.46 (0.26-0.82)	0.008	0.220	0.54 (0.19-1.57)	0.259	0.772	0.69 (0.25-1.88)	0.469	0.942
WFIKKN2	0.81 (0.42-1.54)	0.514	0.946	1.16 (0.36-3.77)	0.803	0.997	1.05 (0.30-3.63)	0.943	0.992
WIF1	1.01 (0.55-1.86)	0.980	0.999	2.50 (0.83-7.52)	0.103	0.528	0.73 (0.23-2.32)	0.589	0.965
WNT9A	1.78 (1.00-3.16)	0.048	0.430	2.10 (0.73-5.99)	0.167	0.649	2.42 (1.08-5.41)	0.031	0.605
WWP2	1.12 (0.74-1.70)	0.597	0.962	1.34 (0.63-2.86)	0.452	0.920	1.08 (0.50-2.37)	0.838	0.991

XCL1	1.98 (1.50-2.60)	<0.001	0.002	1.77 (1.07-2.92)	0.027	0.284	1.76 (1.00-3.11)	0.050	0.670
XG	2.64 (1.63-4.28)	<0.001	0.021	3.38 (1.46-7.85)	0.005	0.140	2.30 (0.97-5.44)	0.059	0.698
XIAP	0.98 (0.62-1.54)	0.917	0.999	1.12 (0.50-2.52)	0.783	0.997	0.66 (0.26-1.72)	0.398	0.942
XPNPEP2	0.94 (0.74-1.18)	0.570	0.956	0.90 (0.59-1.37)	0.625	0.983	0.86 (0.57-1.28)	0.449	0.942
XRCC4	1.14 (0.68-1.91)	0.621	0.966	1.39 (0.62-3.11)	0.417	0.903	1.27 (0.47-3.42)	0.631	0.973
YAP1	2.21 (1.31-3.72)	0.003	0.129	2.59 (1.15-5.84)	0.022	0.261	3.19 (1.59-6.41)	0.001	0.185
YARS1	1.03 (0.87-1.21)	0.750	0.986	1.17 (0.85-1.60)	0.327	0.831	1.00 (0.73-1.37)	0.993	0.998
YES1	1.11 (0.92-1.35)	0.284	0.806	1.13 (0.79-1.62)	0.500	0.941	1.14 (0.79-1.67)	0.482	0.944
YJU2	0.41 (0.17-0.99)	0.048	0.430	1.09 (0.26-4.49)	0.907	0.997	0.10 (0.02-0.55)	0.008	0.402
YOD1	0.90 (0.61-1.33)	0.604	0.962	1.13 (0.58-2.19)	0.715	0.995	0.95 (0.48-1.87)	0.886	0.992
YTHDF3	0.87 (0.67-1.12)	0.278	0.804	0.88 (0.54-1.42)	0.595	0.972	0.80 (0.49-1.30)	0.366	0.939
YWHAQ	0.97 (0.71-1.34)	0.876	0.998	1.26 (0.71-2.24)	0.420	0.903	0.82 (0.45-1.50)	0.523	0.948
YY1	1.24 (0.48-3.19)	0.657	0.971	1.22 (0.21-7.09)	0.821	0.997	1.12 (0.19-6.50)	0.899	0.992
ZBP1	1.21 (0.67-2.21)	0.531	0.948	2.25 (1.02-4.93)	0.044	0.363	0.52 (0.12-2.18)	0.370	0.939
ZBTB16	1.12 (0.83-1.50)	0.461	0.920	0.94 (0.53-1.66)	0.833	0.997	0.89 (0.49-1.61)	0.696	0.975
ZBTB17	1.35 (0.85-2.14)	0.203	0.732	1.71 (0.84-3.50)	0.139	0.604	1.33 (0.58-3.07)	0.500	0.947
ZCCHC8	0.99 (0.63-1.57)	0.974	0.999	1.35 (0.75-2.41)	0.319	0.823	0.72 (0.26-2.00)	0.534	0.952
ZFYVE19	0.94 (0.72-1.23)	0.652	0.971	1.16 (0.72-1.87)	0.538	0.954	0.82 (0.50-1.32)	0.412	0.942
ZHX2	0.77 (0.32-1.84)	0.550	0.949	0.71 (0.14-3.65)	0.679	0.993	0.99 (0.29-3.41)	0.992	0.998
ZNF174	0.88 (0.36-2.16)	0.785	0.994	1.19 (0.25-5.60)	0.822	0.997	1.13 (0.23-5.62)	0.877	0.992
ZNF75D	0.99 (0.67-1.48)	0.978	0.999	0.96 (0.46-2.01)	0.924	0.997	1.02 (0.54-1.92)	0.951	0.992
ZNF830	0.79 (0.52-1.21)	0.279	0.804	0.98 (0.50-1.91)	0.942	0.997	0.32 (0.10-1.00)	0.049	0.670
ZNRD2	1.12 (0.79-1.58)	0.519	0.947	1.28 (0.70-2.36)	0.425	0.905	1.06 (0.56-2.01)	0.855	0.992
ZNRF4	1.04 (0.66-1.65)	0.866	0.998	1.65 (0.89-3.04)	0.109	0.545	0.60 (0.21-1.68)	0.327	0.932
ZP3	1.07 (0.96-1.20)	0.216	0.746	1.01 (0.84-1.22)	0.920	0.997	1.28 (0.98-1.69)	0.071	0.718

ZP4	1.52 (0.66-3.50)	0.330	0.846	1.74 (0.41-7.31)	0.452	0.920	0.67 (0.09-4.80)	0.690	0.975
ZPR1	0.88 (0.65-1.18)	0.385	0.878	0.65 (0.33-1.27)	0.203	0.710	0.41 (0.18-0.94)	0.035	0.623

Cox model adjusted for age, sex, smoking status, alcohol consumption, body mass index, education level, ethnic, and Townsend deprivation index.

Table S7. C-index for plasma proteins

Proteins	Outcome	C_index	Lower_CI	Upper_CI
CD109	ACD_status	0.648458	0.57688	0.720035
COL4A1	ACD_status	0.660885	0.586075	0.735695
CHRDL1	ACD_status	0.673977	0.60188	0.746075
CD276	ACD_status	0.652617	0.586647	0.718587
RNASE1	ACD_status	0.624217	0.55045	0.697985
IL18R1	ACD_status	0.64209	0.573102	0.711079
GFAP	ACD_status	0.708258	0.641386	0.775131
SCARA5	ACD_status	0.622874	0.549003	0.696745
PTK7	ACD_status	0.633601	0.567351	0.699851
ELN	ACD_status	0.674385	0.59412	0.754649
TNFRSF12A	ACD_status	0.662072	0.591548	0.732596
TNFRSF10A	ACD_status	0.655175	0.582356	0.727995
XG	ACD_status	0.653074	0.576058	0.730091
CCN5	ACD_status	0.626133	0.548489	0.703776
COL6A3	ACD_status	0.635116	0.555962	0.714269
EDA2R	ACD_status	0.68524	0.616963	0.753516
NEFL	ACD_status	0.69373	0.618214	0.769246
ICAM5	ACD_status	0.638832	0.571015	0.706648
RNASE6	ACD_status	0.657798	0.5852	0.730396
XCL1	ACD_status	0.641962	0.569707	0.714217
LECT2	ACD_status	0.696503	0.625306	0.7677
GDF15	ACD_status	0.672941	0.60439	0.741492
ATRAID	ACD_status	0.605716	0.534822	0.676609

TNFRSF10B	ACD_status	0.646929	0.571454	0.722403
SLC39A14	ACD_status	0.619351	0.547761	0.690941
BST2	ACD_status	0.632078	0.556348	0.707809
FGF23	ACD_status	0.62356	0.543162	0.703958
GUCY2C	ACD_status	0.580561	0.503311	0.65781
KLK3	ACD_status	0.66195	0.589348	0.734552
TNXB	AD_status	0.716389	0.595691	0.837088
NTRK2	AD_status	0.709466	0.597526	0.821405
LAMP1	AD_status	0.690473	0.554542	0.826403
CTHRC1	AD_status	0.715512	0.576645	0.854379
CPA4	AD_status	0.756035	0.659668	0.852401
RNASET2	AD_status	0.707023	0.580611	0.833435
DIPK2B	AD_status	0.788448	0.710042	0.866854
SFRP4	AD_status	0.730545	0.625065	0.836025
CCDC80	AD_status	0.746669	0.627481	0.865856
CHRDL1	AD_status	0.769147	0.66944	0.868854
FBLN2	AD_status	0.688434	0.551779	0.825088
SMOC2	AD_status	0.724285	0.606648	0.841922
GFAP	AD_status	0.708825	0.566145	0.851506
PTPRH	AD_status	0.743278	0.653257	0.833299
ULBP2	AD_status	0.738204	0.60991	0.866498
COL6A3	AD_status	0.719401	0.600674	0.838127
TNFRSF4	AD_status	0.760563	0.647289	0.873837
PRSS22	AD_status	0.72336	0.629254	0.817467
TIMP2	AD_status	0.752383	0.656191	0.848575

SDC1	AD_status	0.703348	0.589554	0.817142
CSPG5	AD_status	0.649025	0.530767	0.767284
CNGB3	AD_status	0.658249	0.525378	0.791121
SLC39A14	AD_status	0.639328	0.516699	0.761956
GUCY2C	AD_status	0.703158	0.577876	0.828441
NT5C1A	VaD_status	0.711123	0.612095	0.810151
KLK3	VaD_status	0.731596	0.607548	0.855644

ACD, all-cause dementia; AD, Alzheimer's disease; VaD, vascular dementia.

Table S8. Associations of plasma proteins with dementia risk among male and female sleep apnea patients

	Female			Male		
	**Case/Total**	**HR (95%CI)**	***P***	**Case/Total**	**HR (95%CI)**	***P***
**All-cause dementia**						
GFAP	29/413	2.35 (1.19-4.65)	0.014	34/869	3.35 (1.79-6.26)	<0.001
**Alzheimer's disease**						
DIPK2B	9/413	19.56 (1.76-217.07)	0.015	10/869	15.06 (2.69-84.26)	0.002
**Vascular dementia**						
KLK3	6/413	0.21 (0.05-0.80)	0.022	13/869	0.43 (0.29-0.65)	<0.001

Cox model adjusted for age, sex, smoking status, alcohol consumption, body mass index, education level, ethnic, and Townsend deprivation index.

Table S9. Associations of plasma proteins with dementia risk with additional adjustment for diagnosis-to-detection time interval

	HR (95%CI)	*P*
**All-cause dementia**		
GFAP	2.94 (1.85-4.66)	<0.001
**Alzheimer's disease**		
DIPK2B	9.28 (3.22-26.76)	<0.001
**Vascular dementia**		
KLK3	0.42 (0.29-0.62)	<0.001

Cox model adjusted for age, sex, smoking status, alcohol consumption, body mass index, education level, ethnic, Townsend deprivation index and diagnosis-to-detection time interval.
